# Novel insights into the pleiotropic health effects of growth differentiation factor 11 gained from genome-wide association studies in population biobanks

**DOI:** 10.1186/s12864-024-10710-7

**Published:** 2024-09-06

**Authors:** Jessica Strosahl, Kaixiong Ye, Robert Pazdro

**Affiliations:** 1 Department of Nutritional Sciences, University of Georgia, 305 Sanford Drive, Athens, GA 30602 USA; 2 Department of Genetics, University of Georgia, Athens, GA 30602 USA; 3 Institute of Bioinformatics, University of Georgia, Athens, GA 30602 USA

**Keywords:** Growth differentiation factor 11, PheWAS, Asthma

## Abstract

**Background:**

Growth differentiation factor 11 (GDF11) is a member of the transforming growth factor-β (TGF-β) superfamily that has gained considerable attention over the last decade for its observed ability to reverse age-related deterioration of multiple tissues, including the heart. Yet as many researchers have struggled to confirm the cardioprotective and anti-aging effects of GDF11, the topic has grown increasingly controversial, and the field has reached an impasse. We postulated that a clearer understanding of GDF11 could be gained by investigating its health effects at the population level.

**Methods and results:**

We employed a comprehensive strategy to interrogate results from genome-wide association studies in population Biobanks. Interestingly, phenome-wide association studies (PheWAS) of *GDF11* tissue-specific *cis*-eQTLs revealed associations with asthma, immune function, lung function, and thyroid phenotypes. Furthermore, PheWAS of *GDF11* genetic variants confirmed these results, revealing similar associations with asthma, immune function, lung function, and thyroid health. To complement these findings, we mined results from transcriptome-wide association studies, which uncovered associations between predicted tissue-specific *GDF11* expression and the same health effects identified from PheWAS analyses.

**Conclusions:**

In this study, we report novel relationships between GDF11 and disease, namely asthma and hypothyroidism, in contrast to its formerly assumed role as a rejuvenating factor in basic aging and cardiovascular health. We propose that these associations are mediated through the involvement of GDF11 in inflammatory signaling pathways. Taken together, these findings provide new insights into the health effects of GDF11 at the population level and warrant future studies investigating the role of GDF11 in these specific health conditions.

**Supplementary Information:**

The online version contains supplementary material available at 10.1186/s12864-024-10710-7.

## Introduction

Growth Differentiation Factor 11 (GDF11) is a circulating member of the transforming growth factor β (TGF-β) superfamily that has essential roles in mammalian development. Genetic knockout studies have revealed the far-reaching effects of GDF11 on the developing embryo. Most dramatically, global, constitutive deletion of the *Gdf11* gene in mice caused early mortality, with *Gdf11*^−/−^ mice dying within 24 h after birth [1, 2]. Those mice also exhibited anterior homeotic transformations of the axial skeleton [1, 2], an effect mediated by Smad signaling pathways [3–5]. Other studies showed that *Gdf11*^−/−^ mice exhibit renal agenesis, cleft palate [6] and greater numbers of pancreatic NGN3 + islet progenitor cells [7, 8], retinal ganglion cells [9], and olfactory epithelium progenitor cells [10], resulting from the loss of negative feedback of *Gdf11* on progenitor cell number. In adult mice and humans, GDF11 has been implicated in cell differentiation and tissue repair processes, including erythropoiesis [11–13], angiogenesis [14–16], bone homeostasis [17, 18] and myogenesis [19, 20]. In humans, individuals with heterozygous loss-of-function mutations in the *GDF11* gene present severe craniofacial and vertebral abnormalities, in addition to other multisystemic phenotypes such as visual and hearing disorders, cardiac abnormalities, and connective tissue dysfunction [21].

Beyond its defined roles in development, research over the past several years has indicated that GDF11 may have a powerful role in rejuvenating aged tissues, leading to a surge of interest in – and controversies surrounding – this factor. In a landmark study using heterochronic parabiosis, Loffredo et al*.* discovered that blood from young mice reversed age-related cardiac hypertrophy, and ultimately, GDF11 was identified as the blood-borne factor behind this effect [22]. According to the study, systemic GDF11 levels decreased with age, and treating old mice with recombinant GDF11 reversed histopathological and molecular markers of cardiac hypertrophy. Similar findings were recapitulated in other tissues, including skeletal muscle [23], brain [24–26], and skin [27–29]; meanwhile, other studies in mice and humans reported conflicting findings [30–36], claiming no effect of GDF11 on cardiac structure or function [30] or even suggesting that it is a risk factor for comorbidity and frailty in older adults with cardiovascular disease [31]. These contradictions were attributed to multiple issues – most prominent among them was the inability of various antibody-based assays to distinguish between GDF11 and its homolog, myostatin (MSTN) [22, 33, 37], which share 90% amino acid sequence identity in their mature domains [38], leading to confusion around the independent cardiac impact of each protein. But addressing these issues has not fully resolved the discrepancies in the field, as more recent studies have still presented contradictory results. Several studies reported adverse effects of GDF11 on cardiovascular and overall health [39, 40], demonstrating that GDF11 can induce cardiac dysfunction and pathologic atrophy [39] and even cause severe cachexia and death at high doses [40]. Yet most studies still support a cardioprotective role for GDF11, showing that it ameliorates pathological remodeling [41, 42], mitigates ischemia–reperfusion injury [43–45], improves outcomes in myocardial infarction [46, 47], and is associated with lower risk of cardiovascular events and death [37]. Despite a greater understanding of the factors contributing to the discrepancies across GDF11 studies, the precise health effects of this circulating protein remain unresolved.

We posited that a new understanding of GDF11 and the heart could be gained by bridging the work previously done in inbred mouse strains and studies of human participants; for this purpose, we selected an outbred mouse population [48–50] and identified a suggestive peak underlying natural variation in serum GDF11 on murine chromosome 3 [51]. The peak lies in close proximity to the protein-coding gene *Hes Related Family BHLH Transcription Factor with YRPW Motif 1* (*Hey1*), a transcriptional repressor involved in the regulation of cardiac atrioventricular canal and vasculature development through Notch-dependent signaling pathways [52–55]. Moreover, genetic mapping of serum MSTN levels revealed a significant locus on murine chromosome 3 near the protein-coding gene *Forkhead Box O1* (*FoxO1)*. Surprisingly, statistical analyses only revealed weak, inconsistent relationships between serum GDF11 and cardiac health, whereas MSTN exhibited significant negative associations with heart weight, heart weight standardized to tibial length, and left ventricular heart wall thickness [51]. These results did not support an anti-hypertrophic effect for GDF11 but instead added to the large body of evidence suggesting that GDF11 is not a major predictor of cardiovascular health. At this point, a new approach is needed to resolve the true health effects of GDF11 in adulthood and aging.

In the present study, we sought to define the unique health effects of GDF11 at the population level – by interrogating the impact of *GDF11* variants on health outcomes using published GWAS results and biobank data from large cohorts, including the UK Biobank – and contrasting the results against those from MSTN. Here, we employed a comprehensive set of analyses that utilizes the deep genetic and phenotypic data housed in public databases. We began by identifying tissue-specific *cis*-eQTLs [56–59], taking into account the wide range of tissues with detectable *GDF11* expression in humans [60, 61] and mice [62, 63]. We then leveraged data from phenome-wide association studies (PheWAS) to identify pleiotropic effects, where a single locus affects multiple distinct phenotypes [64–66]. Finally, to evaluate the connection between the tissue-specific expression of *GDF11* and human disease, we explored integrative platforms housing transcriptome-wide association study (TWAS) results and functional genomics data [67–70]. Analytical processes were performed in parallel for both GDF11 and MSTN, and we report the unique connections between traits associated with *GDF11* variants and its tissue-specific expression level that expand our knowledge of the health impacts of this gene.

## Methods

UK Biobank is a globally accessible, large-scale longitudinal population cohort containing genetic and disease information from over 500,000 British individuals [71]. Participants ranging from 40 to 69 years of age were recruited between the years 2006 and 2010 [71]. The North West Multi-Centre Research Ethics Committee (11/NW/ 0382) approved the UK Biobank project. Informed consent was obtained from each participant prior to collection of biological and anthropometric measurements, lifestyle indicators, blood and urine biomarkers, and information from their medical records [71].

The Genotype-Tissue Expression (GTEx) project collected 15,201 RNA-sequencing samples from 54 tissues of 838 postmortem donors. Biospecimen Source Sites (BSS) were required to submit a GTEx research protocol and undergo IRB review or forwent further review on account of deceased donors not constituting as human subjects [72, 73]. However, GTEx required explicit next-of-kin or legal representative authorization for study participation, given the public availability of the data [72]. Specific training regulating how BSS obtained consent can be found at http://gtextraining.org/. Only de-identified data according to HIPPAA policy is distributed to GTEx project collaborators [72].

### Single-tissue expression-quantitative trait locus analysis

We utilized the GTEx portal to identify variants that were significantly associated with *GDF11* and *MSTN* expression levels. Specifically, we searched for *cis*-eQTLs of *GDF11* and *MSTN* across tissues. The GTEx project is an open-access database with data including gene expression, QTLs, and histology images from 54 non-diseased tissue sites across nearly 1000 individuals [74, 75]. Briefly, quality control (QC) was performed as follows: RNA-seq expression outliers were excluded based on previously described methods [76], read counts for samples were normalized and log-transformed with an offset of 1, and the read count matrix was hierarchically clustered [72]. In addition, samples with < 10 million mapped reads were removed, and if replicate samples were present, the replicate with the greatest number of reads was selected [72]. The data used for the analyses described in this manuscript were obtained from the GTEx Portal on 11/08/2022.

### Transcriptome-wide association study

We used TWAS results from the TWAS Hub to identify traits associated with the tissue-specific expression of *GDF11* and *MSTN*, as well as their putative genetic regulators, *HEY1* and *FOXO1*, respectively. TWAS leverage gene expression measurements with summary statistics from large-scale GWAS to identify significant expression-trait associations [67]. GWAS and functional data for hundreds of traits and over 100,000 expression models were integrated within TWAS Hub [67, 77]. Summary association statistics came from 30 large-scale GWAS studies, and SNPs with minor allele frequencies of less than 1% were removed [70]. RNA sequencing data originated from CommonMind Consortium (brain, *n* = 613) [78], GTEx (41 tissues) [60], and the Metabolic Syndrome in Men study (adipose, *N* = 563) [79, 80], and expression microarray data from the Young Finns Study (blood, *N* = 1,264) [81, 82] and the Netherlands Twins Registry (*N* = 1,247) [70, 76]. Associations were considered significant if they reached the tissue-specific threshold determined by Bonferroni correction at an experimental α of 0.05, as a conservative measure.

The PhenomeXcan database was used to supplement results from the TWAS hub. PhenomeXcan is a gene-based program which houses 22,255 gene associations and 4,091 traits with transcriptome regulation data from 49 tissues in GTEx v8 using an adaptation of the PrediXcan method [83–85]. Colocalization analysis was performed via fastENLOC, a novel Bayesian hierarchical colocalization method [83, 84]. We utilized the ‘PhenomeXcan_SingleTissue' function, including 4,091 traits and 49 tissues. We only included associations that met the significance threshold *p* < 1e-08.

### Variant-centric analysis

To investigate associations between health outcomes and variants of *GDF11*, *HEY1*, *MSTN*, and *FOXO1*, we employed the Open Target Genetics, PhenoScanner, GeneATLAS, and GWAS Catalog databases. Open Target Genetics is an open-access integrative database that combines human GWAS and functional genomics data to allow researchers to conduct systematic identification and prioritization of plausible causal variants and genes [86–88]. GWAS with and without summary statistics were sourced from the NHGRI-EBI GWAS Catalog summary statistics repository (number of studies = 300; only included associations with *p* ≤ 5*e* − 8 and removed redundant associations via distance-based clustering ± 500 kb) [89]. GWAS with full summary statistics were sourced from two GWAS analyses using UK Biobank data: the SAIGE study of binary phenotypes (number of studies = 2,139) [90], and the Neale Lab study (number of studies = 1,283) [91]. Full GWAS summary statistics were only included from those studies of predominantly European ancestries due to limited reference genotypes from other populations [87, 89]. Additionally, 92 tissue- and cell-type-specific molecular QTL (molQTL) datasets were integrated from GTEx [60], eQTLGen [92], the eQTL Catalogue, and pQTLs [93], and systematic disease-molecular trait colocalization tests were performed [87]. Variants are sorted by their locus to gene (L2G) pipeline score on a scale from 0–1 based on evidence including molecular phenotype quantitative trait loci data, chromatin interaction data, in silico functional predictions, and distance from the canonical transcript start site [86, 87]. Associations from all studies were only included if *p* ≤ 5e − 8 [86, 87].

PhenoScanner is a database that contains over 65 billion association results, including eQTL, pQTL, methylation QTL (mQTL), and upwards of 150 million genetic variants to enable researchers to conduct “phenome-wide scans” [94–96]. Variants with minor allele frequencies < 0.5%, multiallelic variants, and large indels (⁠ ≥ 5 bases) were removed from analyses [94, 95]. Variants were positionally annotated utilizing the Variant Effect Predictor, and traits were mapped to Experimental Factor Ontology Terms [94, 95]. The significance cut-off *p* < 1e–5 was used for all genes, genomic regions, and phenotypes [94, 95].

GeneATLAS utilizes the UK Biobank cohort (*N* = 452,264) of British individuals to systematically catalog associations between 778 traits and over 30 million variants [97, 98]. The associations were computed by use of Mixed Linear Models in a large supercomputer using the DISSECT software (freely available at https://www.dissect.ed.ac.uk under GNU Lesser General Public License v3). We utilized the GeneATLAS “region PheWAS” function to extract PheWAS associations located ± 1000 kb of our genes of interest, and significance was determined at *p* ≤ 1e-8. Additionally, we searched the GWAS Catalog for significant associations with the genes of interest. We only included associations that met the significance threshold *p* ≤ 5e − 8.

## Results

### Tissue-specific *cis*-eQTLs for GDF11 are associated with asthma, immune function, lung function, and thyroid phenotypes

**Fig. 1:**
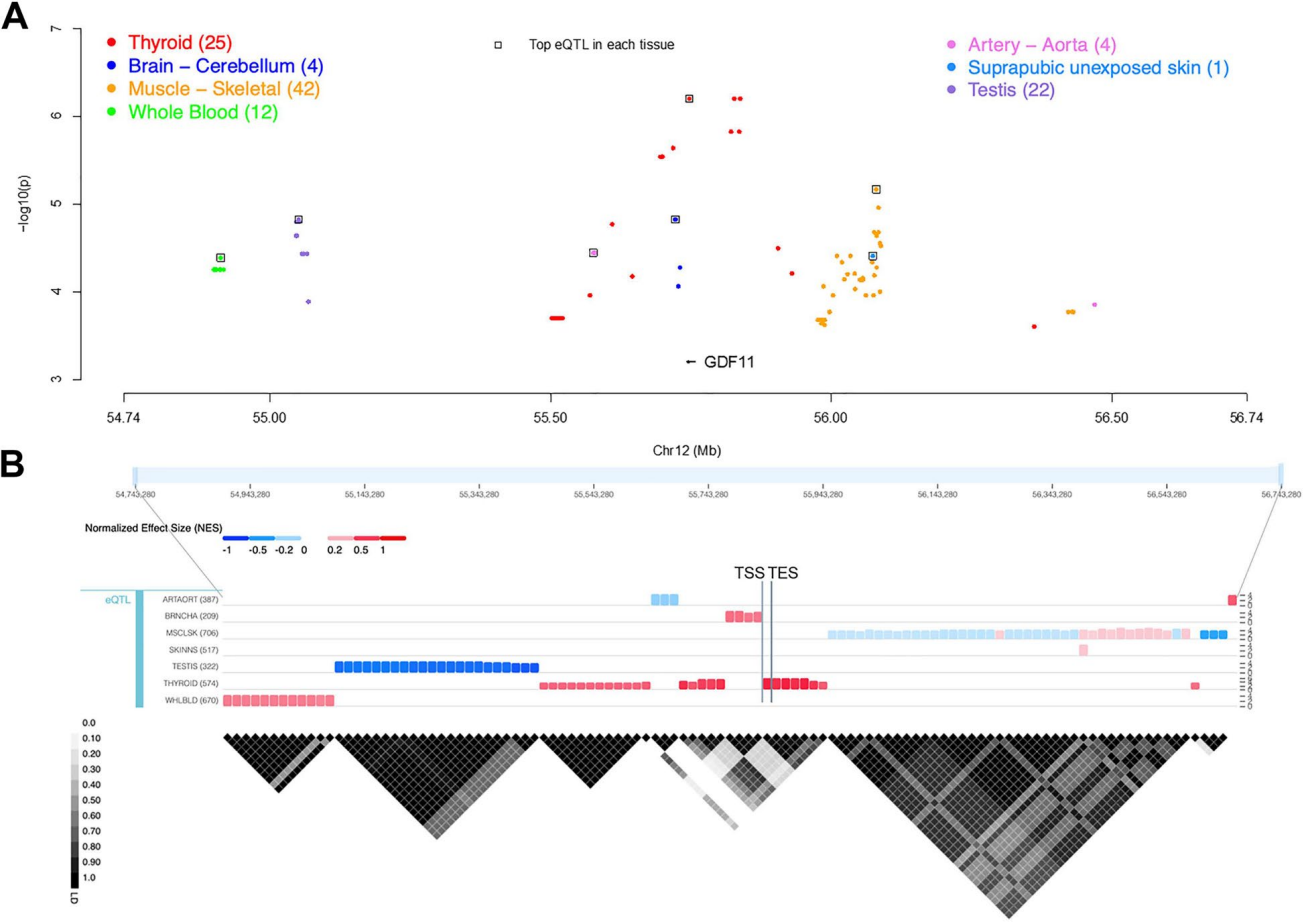
Genetic variants significantly associated with *GDF11* expression as detected in the GTEx project. **A** *GDF11 cis*-eQTLs (± 1 Mb of the transcription start site) separated by tissue. Specific tissues are denoted by colors. The most significant eQTL in each tissue is denoted by a black square. **B** Linkage disequilibrium (LD) blocks for *GDF11 cis*-eQTLs from the GTEx project. The LD heatmap reports pairwise LD values (R^2^) of the QTL variants. The corresponding eQTL normalized effect size (NES) bar chart heatmaps are located above the LD heatmap. Row labels (to the left of each chart) denote the tissue type and number of samples. The y-axis (to the right of each chart) is the -log_10_(*p*-value). Tissue abbreviations: ARTAORT = Artery – Aorta; BRNCHA = Brain – Cerebellum; MSCLSK = Muscle – Skeletal; SKINNS = Skin – Not Sun Exposed (Suprapubic); WHLBLD = Whole Blood. TSS = transcription start site; TES = transcription end site

To comprehensively define the health effects of *GDF11* variation, we started by identifying variants that (1) lie in close proximity to the *GDF11* gene and (2) are associated with its expression levels in at least one tissue. We compiled a total of 110 variants located from within ± 1 Mb of the transcription start site (TSS). Those *cis*-eQTLs spanned the genomic region of chr12:54,899,536–56,468,936 base pairs (bp; Supplementary Table S1), and a visual depiction of all *cis*-eQTLs associated with *GDF11* expression is shown in Fig. 1A, which highlights a tissue-specific association pattern (Fig. 1B). From there, we narrowed the list of *cis*-eQTLs for further examination by selecting the most significant variant for each tissue as a representative of the haplotype block. Notably, the most significant *GDF11 cis*-eQTL, rs117385153, was associated with *GDF11* expression in thyroid tissue (*p* = 6.30E-07; Table 1). A visual of all genes located in the *GDF11 cis*-window can be found in the Supplementary file (Supplementary Fig. S1).

**Table 1:** Tissue-specific *GDF11 cis*-eQTLs are significantly associated with asthma, immune function, lung function, and hypothyroidism in PhenoScanner. The most significant *cis*-eQTL from each tissue identified in the GTEx project is listed in the table. Trait associations were identified in PhenoScanner. A *p*-value less than 1e-5 was considered statistically significant

*Cis-*eQTL	eQTL *p*-value	*cis*-eQTL tissue	Trait	*P*-value	GWAS source	Beta	N
rs117385153	6.30E-07	Thyroid	Self-reported pulmonary fibrosis	2.76E-05	UKBB	0.0005422	337,159
			Cause of death: asthma, unspecified	6.54E-05	UKBB	0.007471	7637
rs7297175	0.0000068	Skeletal muscle	No blood clot, bronchitis, emphysema, asthma, rhinitis, eczema or allergy diagnosed by doctor	8.03E-19	UKBB	0.01017	336,782
			Eosinophil percentage of white cells	6.52E-14	PMID: 27863252	-0.02689	173,480
			Eosinophil count	5.02E-13	PMID: 27863252	-0.02593	173,480
			Self-reported asthma	6.16E-13	UKBB	-0.0056633	337,159
			Asthma	1.91E-12	UKBB	-0.00553	336,782
			Sum eosinophil basophil counts	5.70E-12	PMID: 27863252	-0.02474	173,480
			Eosinophil percentage of granulocytes	1.93E-11	PMID: 27863252	-0.02417	173,480
			Neutrophil percentage of granulocytes	1.30E-10	PMID: 27863252	0.02315	173,480
			Forced expiratory volume in 1-s, best measure	7.98E-07	UKBB	0.01138	255,492
			Doctor diagnosed asthma	6.60E-06	UKBB	-0.007408	83,529
			Self-reported hypothyroidism or myxoedema	1.17E-10	UKBB	-0.003373	337,159
rs3138140	0.000015	Cerebellum	NA	NA	NA	NA	NA
rs879920435	0.000015	Testis	NA	NA	NA	NA	NA
rs59980219	0.000036	Aorta	NA	NA	NA	NA	NA
rs7312770	0.000039	Unexposed suprapubic skin	No blood clot, bronchitis, emphysema, asthma, rhinitis, eczema or allergy diagnosed by doctor	3.85E-15	UKBB	-0.009001	336,782
			Self-reported asthma	2.38E-12	UKBB	0.005501	337,159
			Asthma	8.07E-12	UKBB	0.005355	336,782
			Eosinophil percentage of white cells	8.26E-11	PMID: 27863252	0.02326	173,480
			Eosinophil count	1.23E-10	PMID: 27863252	0.02307	173,480
			Sum eosinophil basophil counts	2.72E-10	PMID: 27863252	0.02265	173,480
			Eosinophil percentage of granulocytes	1.51E-08	PMID: 27863252	0.02036	173,480
			Forced expiratory volume in 1-s, best measure	1.52E-08	UKBB	-0.01301	255,492
			Neutrophil percentage of granulocytes	1.87E-08	PMID: 27863252	-0.02023	173,480
			Doctor diagnosed asthma	2.35E-06	UKBB	-0.01172	317,756
			Forced expiratory volume in 1-s	2.48E-06	UKBB	-0.009861	307,638
			Self-reported hypothyroidism or myxoedema	7.93E-09	UKBB	0.003012	337,159
			Hypothyroidism	8.00E-08	PMID: 27182965	NA	NA
rs76779798	0.000041	Whole blood	NA	NA	NA	NA	NA

Next, we aimed to identify the health effects of the most significant *GDF11 cis-*eQTL in each tissue. We began by performing a PheWAS in PhenoScanner for rs117385153, the most significant variant in thyroid tissue, and the results pointed to associations with “self-reported pulmonary fibrosis” (*p* = 2.76E-05) and “cause of death: asthma, unspecified” (*p* = 6.54E-05). Then, we performed a PheWAS for the *cis-*eQTLs with the highest statistical significance from other tissues. The most significant *cis-*eQTL in skeletal muscle tissue was rs7297175 (*p* = 6.8E-6), and PheWAS results for this SNP revealed associations with 11 traits related to asthma, immune function, lung function, and thyroid health (Table 1). We identified rs7312770 as the most significant *cis-*eQTL in unexposed suprapubic skin (*p* = 3.9E-5), and PheWAS analysis of this SNP indicated associations with 13 traits related to asthma, immune function, lung function, and thyroid health (Table 1). More information on sample data and procedures in unexposed suprapubic skin can be found in Supplementary Table S2. Both rs7297175 and rs7312770 exhibited the most significant association with the entry “no blood clot, bronchitis, emphysema, asthma, rhinitis, eczema or allergy diagnosed by a doctor." Lastly, the most significant *cis*-eQTLs in the cerebellum, testis, aorta, and whole blood were rs3138140 (*p* = 1.5E-5), rs879920435 *(p* = 1.5E-5), rs59980219 (*p* = 3.6E-5), and rs76779798 (*p* = 4.1E-5), respectively. PheWAS of these *cis*-eQTLs did not return any significant associations in PhenoScanner.

To contrast the health impacts of *GDF11* against those of its homolog, *MSTN*, we searched for all *MSTN cis*-eQTLs in the GTEx portal and identified 771 significant variants (Supplementary Table S3). A visual depiction of all variants associated with *MSTN* expression can be found in Supplementary Fig. S2A. We observed a similar tissue-specific association pattern among *MSTN cis*-eQTLs as we previously did for those associated with *GDF11* (Supplementary Fig. S2B). In contrast to *GDF11*, PheWAS of the most significant *MSTN cis*-eQTL in each tissue did not reveal consistent associations with any particular health conditions (Supplementary Table S4). As a result, we were unable to find any overlap between *GDF11* and *MSTN cis*-eQTL PheWAS data.

Importantly, we included a positive control in the form of a benchmark gene to evaluate the reliability of our inquiry. We used a SNP (rs174546), whose PheWAS results have been previously reported [99]. We confirmed that our PheWAS of this SNP reproduced all the previously reported associations (Supplementary Table S5).

### *GDF11* variants demonstrate links to asthma, immune function, lung function, and thyroid health

**Fig. 2:**
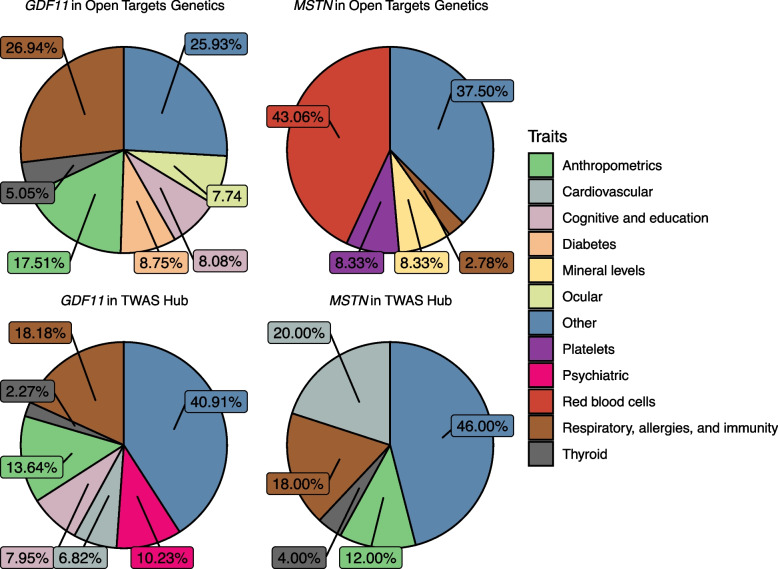
Traits associated with variants and predicted tissue-specific expression of *GDF11* and *MSTN* in Open Targets Genetics and the TWAS Hub. Traits were sorted by disease category to identify the most prevalent associations in each database. Disease categories are denoted by colors in the legend. Traits that did not fit a category were placed into the “other” category

The Open Targets Genetics Portal integrates GWAS and functional genomics data to allow for variant-centric analysis across thousands of traits. Variants were assigned to genes based on predicted functional effects, distance from the transcript start site, molecular phenotype quantitative trait loci experiments, and chromatin interaction experiments [86, 87]. To further analyze the health effects of *GDF11* variants, we employed the Open Targets Genetics Portal and identified 297 associations with *GDF11* variants (Supplementary Table S6). We sorted the total number of associations by disease category to identify the most prevalent health impact of *GDF11* variation (Fig. 2A). Associations related to a specific disease were grouped together. For example, asthma and eosinophil counts were grouped with respiratory health, allergies, and immunity, and hypothyroidism was grouped with thyroid traits. Associations that did not easily fit a disease classification were placed in the “other” category.

**Fig. 3:**
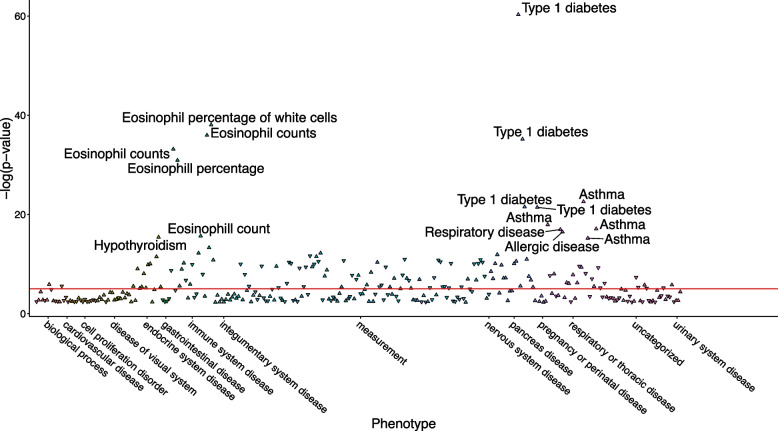
PheWAS results from Open Target Genetics reveal associations between the SNP rs1689510 and asthma, immune function, lung function, and hypothyroidism. The x-axis represents categories of disease traits, and the y-axis represents the -log_10_ (*p*-value) of each association. The red significance line represents *p* = 1e-5. Associations that met the threshold *p* < 1e-14 are labeled in the figure. Results are sourced from FinnGen, UK Biobank, and GWAS Catalog

Among the associations we identified with *GDF11* variants, the largest portion (26.94%) were traits related to respiratory health, allergies, and immunity. The association with the highest statistical significance in this category was observed between rs705702 and “eosinophil counts” (*p* = 1.6E-41; Table 2), whereas the most significant association overall was between rs61134397 and “refractive error” (*p* = 6.0e-174). Asthma emerged as the most prevalent trait, accounting for 32.86% of all respiratory health, allergies, and immunity associations. Intriguingly, we found another *GDF11* variant, rs1689510, exhibited significant associations with asthma, immune function, and lung function across multiple studies (Table 2). To further investigate this SNP, we searched for all associations with rs1689510 in the Open Targets Genetics Portal and presented those data in Fig. 3. The most statistically significant association with rs1689510 was “type 1 diabetes” (*p* = 5.0e-61), followed by “eosinophil percentage of white cells” (*p* = 8.9e-39). rs1689510 was also associated with “asthma” (*p* = 2.7e-23), and “hypothyroidism or myxedema” (*p* = 3.9e-16) (Supplementary Table S7).

**Table 2:** *GDF11* variants are associated with asthma, immune function, lung function, and hypothyroidism in Open Target Genetics. Respiratory, immune function, and lung function associations are listed together and sorted by significance. Thyroid associations are listed separately and sorted by significance. A *p*-value equal to or less than 5*e* − 8 was considered statistically significant. L2G score = “locus-to-gene” score calculated by Open Targets Genetics to weigh each association and prioritize likely candidate genes using a machine-learning model. All associations with *GDF11* variants in Open Target Genetics can be found in Supplementary Table S6

Trait	Variant	N initital	*P*-value	GWAS Source	Reference allele	Effect allele	Beta	Odds ratio	L2G score
Eosinophil counts	rs705702	440,275	1.60E-41	Barton AR (2021) PMID: 34226706	A	G	0.027	NA	0.022
Eosinophil percentage of white cells	rs1689510	408,112	8.90E-39	Vuckovic D (2020) PMID: 32888494	G	C	0.029	NA	0.058
Eosinophil counts	rs1689510	474,237	9.00E-37	Chen MH (2020) PMID: 32888493	G	C	0.027	NA	0.025
Eosinophil counts	rs1689510	583,850	9.00E-37	Chen MH (2020) PMID: 32888493	G	C	NA	NA	0.057
Eosinophill counts	rs1689510	408,112	6.70E-34	Vuckovic D (2020) PMID: 32888494	G	C	0.027	NA	0.048
Eosinophill percentage	rs1689510	349,861	1.20E-31	UKB Neale v2 (2018)	G	C	0.054	NA	0.052
Asthma	rs1689510	536,345	2.00E-31	Han Y (2020) PMID: 32296059	G	C	NA	NA	0.038
Eosinophil counts	rs773110	440,000	4.00E-31	Kichaev G (2018) PMID: 30595370	C	G	NA	NA	0.011
Asthma	rs7302200	341,215	8.00E-25	Ferreira MAR (2019) PMID: 30929738	G	A	NA	1.1	0.04
Asthma (childhood onset)	rs705700	314,633	3.00E-24	Ferreira MAR (2019) PMID: 30929738	T	C	NA	NA	0.01
Asthma	rs1689510	484,598	2.70E-23	Donertas HM (2021) PMID: 33959723	G	C	NA	1	0.05
Asthma [conditional]	rs1689510	281,699	3.00E-20	Johansson A (2019) PMID: 31361310	G	C	NA	NA	0.036
Lung function (FEV1/FVC)	rs1701704	371,898	6.10E-20	Barton AR (2021) PMID: 34226706	T	G	− 0.020	NA	0.053
Lung function (FEV1/FVC)	rs1701704	370,000	1.00E-19	Kichaev G (2018) PMID: 30595370	T	G	NA	NA	0.036
Respiratory diseases	rs1689510	459,000	2.00E-19	Kichaev G (2018) PMID: 30595370	G	C	NA	NA	0.035
Asthma | non-cancer illness code, self-reported	rs1689510	361,141	1.20E-18	UKB Neale v2 (2018)	G	C	NA	1.1	0.054
Eosinophil counts	rs10876864	442,919	2.00E-18	Sakaue S (2021) PMID: 34594039	G	A	-0.015		0.011
Asthma	rs1702877	625,448	3.00E-18	Sakaue S (2021) PMID: 34594039	C	T	0.066	NA	0.036
Respiratory or ear nose throat disease	rs7302200	484,598	6.30E-18	Donertas HM (2021) PMID: 33959723	G	A	NA	1	0.057
Asthma | blood clot, dvt, bronchitis, emphysema, asthma, rhinitis, eczema, allergy diagnosed by doctor	rs1689510	360,527	8.30E-18	UKB Neale v2 (2018)	G	C	NA	1.1	0.053
Asthma (childhood onset)	rs705699	406,621	9.00E-17	Zhu Z (2019) PMID: 31669095	G	A	NA	NA	0.011
Allergic disease (asthma, hay fever or eczema)	rs1689510	360,838	3.40E-17	Ferreira MA (2017) PMID: 29083406	G	C	NA	1.1	0.05
Asthma	rs705700	394,283	1.00E-16	Zhu Z (2019) PMID: 31669095	T	C	NA	1.1	0.011
Eosinophil count	rs1689510	349,856	2.20E-16	UKB Neale v2 (2018)	G	C	0.015	NA	0.05
Atopic asthma	rs705699	417,151	4.00E-16	Zhu Z (2019) PMID: 31669095	G	A	NA	NA	0.011
Asthma	rs1689510	408,442	6.10E-16	Valette K (2021) PMID: 34103634	G	C	NA	1.1	0.054
Asthma	rs10876866	787,635	1.00E-15	Olafsdottir TA (2020) PMID: 31959851	G	A	NA	1.1	0.036
Asthma (adult onset)	rs7302200	327,253	4.00E-15	Ferreira MAR (2019) PMID: 30929738	G	A	NA	NA	0.037
Asthma	rs1701704	305,945	2.00E-14	Salinas YD (2020) PMID: 32700739	T	G	NA	1.1	0.035
Eosinophil percentage of white cells	rs1689510	172,378	5.00E-14	Astle WJ (2016) PMID: 27863252	G	C	0.028	NA	0.054
Asthma or allergic disease (pleiotropy)	rs10876864	116,538	1.00E-13	Zhu Z (2018) PMID: 29785011	G	A	NA	NA	0.016
Asthma	rs1701704	4,836	2.00E-13	Hirota T (2011) PMID: 21804548	T	G	NA	1.2	0.035
Eosinophil counts	rs7302200	172,275	3.00E-13	Astle WJ (2016) PMID: 27863252	G	A	0.027	NA	0.052
Forced vital capacity (fvc), best measure	rs59822547	272,338	6.20E-13	UKB Neale v2 (2018)	A	G	0.027	NA	0.013
Lymphocyte counts	rs1131017	524,923	9.80E-13	Chen MH (2020) PMID: 32888493	C	G	0.014	NA	0.015
Sum eosinophil basophil counts	rs772920	171,771	5.30E-12	Astle WJ (2016) PMID: 27863252	C	G	0.026	NA	0.021
Asthma (childhood onset)	rs705699	327,670	1.00E-11	Pividori M (2019) PMID: 31036433	G	A	NA	1.1	0.012
Asthma	rs1702877	175,948	1.00E-11	Sakaue S (2021) PMID: 34594039	C	T	0.12	NA	0.031
Eosinophil percentage of granulocytes	rs10876864	170,536	1.30E-11	Astle WJ (2016) PMID: 27863252	G	A	-0.024	NA	0.014
Lymphocyte counts	rs1131017	408,112	3.60E-11	Vuckovic D (2020) PMID: 32888494	C	G	0.014	NA	0.02
Neutrophil percentage of granulocytes	rs10876864	170,672	7.10E-11	Astle WJ (2016) PMID: 27863252	G	A	0.024	NA	0.015
Forced expiratory volume in 1-s (fev1), best measure	rs1689510	272,338	3.00E-10	UKB Neale v2 (2018)	G	C	− 0.010	NA	0.069
Asthma	rs1702877	401,837	3.40E-10	UKB SAIGE (2018)	C	T	NA	1.1	0.052
FEV1	rs705704	321,047	3.90E-10	Shrine N (2019) PMID: 30804560	G	A	− 0.015	NA	0.053
Lymphocyte counts	rs1131017	643,370	4.00E-10	Chen MH (2020) PMID: 32888493	C	G	NA	NA	0.011
Allergic disease (asthma, hay fever or eczema)	rs11171739	102,453	4.00E-10	Zhu Z (2019) PMID: 31669095	C	T	NA	NA	0.012
Lymophocyte percentage	12_56041720_G_C (no rsID)	349,861	4.50E-10	UKB Neale v2 (2018)	G	C	-0.11	NA	0.047
Pediatric asthma	rs1702877	601,193	6.00E-10	Sakaue S (2021) PMID: 34594039	C	T	0.06	NA	0.032
Asthma	rs705704	209,808	7.00E-10	Ishigaki K (2020) PMID: 32514122	G	A	NA	1.1	0.028
Asthma (adult onset)	rs705700	426,604	1.00E-09	Zhu Z (2019) PMID: 31669095	T	C	NA	NA	0.012
Asthma (moderate or severe)	rs7305461	30,810	1.00E-09	Shrine N (2018) PMID: 30552067	A	C	NA	0.91	0.013
Nonatopic asthma	rs705700	450,910	2.00E-09	Zhu Z (2019) PMID: 31669095	T	C	NA	NA	0.013
Lymphocyte percentage of white cells	rs1131017	408,112	2.70E-09	Vuckovic D (2020) PMID: 32888494	C	G	0.013	NA	0.022
Lymphocyte counts	rs1131017	443,762	3.80E-09	Barton AR (2021) PMID: 34226706	C	G	0.012	NA	0.02
Allergic disease (asthma, hay fever and/or eczema) (age of onset)	rs705699	117,130	4.00E-09	Ferreira MAR (2019) PMID: 30929738	G	A	-0.025	NA	0.013
Allergic disease (asthma, hay fever and/or eczema) (multivariate analysis)	rs705699	477,968	6.00E-09	Ferreira MAR (2019) PMID: 30929738	G	A	-0.024	NA	0.013
Forced expiratory volume in 1-s (fev1)	rs772920	329,404	6.60E-09	UKB Neale v2 (2018)	C	G	-0.0087	NA	0.022
Lung function (FEV1/FVC)	rs61938962	321,047	7.00E-09	Shrine N (2019) PMID: 30804560	C	T	-0.015	NA	0.08
Lung function (FEV1)	rs772920	90,715	2.00E-08	Wyss AB (2018) PMID: 30061609	C	G	NA	NA	0.017
Hypothyroidism or myxedema	rs772920	484,598	9.50E-17	Donertas HM (2021) PMID: 33959723	C	G	NA	1	0.026
Hypothyroidism	rs772920	459,000	7.00E-15	Kichaev G (2018) PMID: 30595370	C	G	NA	NA	0.017
Thyroid problem (not cancer)	rs7302200	484,598	1.30E-12	Donertas HM (2021) PMID: 33959723	G	A	NA	1	0.052
Autoimmune thyroid disease	rs2271194	755,406	2.00E-11	Saevarsdottir S (2020) PMID: 32581359	A	T	NA	NA	0.011
Hypothyroidism, other/unspecified	rs61938963	254,846	3.10E-09	FINNGEN_R6 (2022)	C	T	NA	1.1	0.047
Hypothyroidism	rs1131017	405,600	3.70E-08	UKB SAIGE (2018)	C	G	NA	0.93	0.017
Hashimoto thyroiditis	rs11611029	568,833	1.00E-08	Sakaue S (2021) PMID: 34594039	C	T	-0.096	NA	0.009
Hypothyroidism/myxoedema | non-cancer illness code, self-reported	rs1398310988	361,141	1.50E-11	UKB Neale v2 (2018)	CA	C	NA	0.92	0.015
Hypothyroidism, strict autoimmune, 3 medication purchases required	rs705702	260,405	2.80E-10	FINNGEN_R6 (2022)	A	G	NA	1.1	0.026
Hypothyroidism, strict autoimmune	rs61938963	235,230	3.00E-11	FINNGEN_R6 (2022)	C	T	NA	1.1	0.055
Hypothyroidism	rs11171710	583,911	3.00E-12	Sakaue S (2021) PMID: 34594039	G	A	-0.07	NA	0.009

To complement discoveries made with Open Targets Genetics, we employed PhenoScanner to screen for associations with *GDF11* variants. We identified 34 total associations with *GDF11* variants, and the most significant association overall was between rs61134397 and “cause of death: pharynx, unspecified” (*p* = 1.763e-21; Supplementary Table S8). The most significant respiratory association was between rs139996303 and “self-reported respiratory infection” (*p* = 5.46E-06; Supplementary Table S8). The investigation also revealed two *GDF11* variants associated with “self-reported pulmonary fibrosis”: rs12304296 and rs7297523 (*p* = 8.69E-06 and *p* = 8.99E-06, respectively; Supplementary Table S8). Additionally, we searched for associations with *GDF11* variants in GeneATLAS and identified associations with respiratory disease, asthma, and hypothyroidism, validating our previous findings (Supplementary Table S9).

Once again, we sought to contrast the health effects of *GDF11* and *MSTN* variants, so we conducted a reciprocal analysis of *MSTN* in the Open Target Genetics Portal and identified 72 significant health associations (Supplementary Table S10). The results indicated that the largest number of associations with *MSTN* variants were red blood cell traits (43.06%), and the most significant association overall was between rs291444 and “serum levels of protein HIBCH” (*p* = 2.0e-258; Supplementary Table S10). The search did not reveal a robust relationship between *MSTN* variants and respiratory health, allergies, and immunity that was found for *GDF11* (Fig. 2B). Lastly, we investigated the health effects of *MSTN* variants in PhenoScanner and GeneATLAS but did not identify consistent associations with a particular disease category (Supplementary Tables S11 and S12).

### Predicted *GDF11* expression is associated with asthma, immune function, lung function, and thyroid health

TWAS Hub is an open-access database that contains genomics data on hundreds of traits and over 100,000 expression models [67]. We identified 91 associations between tissue-specific *GDF11* expression and health outcomes (Fig. 2C; Supplementary Table S13). Predictive models for the expression of *GDF11* were only available for one tissue, unexposed suprapubic skin*.* Associations between *GDF11* expression in the skin and various health conditions were compiled and sorted into disease categories; 18.18% were traits related to respiratory health, allergies, and immunity. "Blood eosinophil count" demonstrated the highest statistical significance within this category (*p* = 4.79E-07, Z-score = -4.9; Table 3), whereas the most significant association with *GDF11* expression overall was “smoking status” (*p* = 1.70e-07, Z-score = 5.1). Other traits associated with *GDF11* expression in this category included “respiratory disease” (*p* = 1.30E-06, Z-score = -4.7), “self-reported asthma” (*p* = 3.17E-05, Z-score = -4.0), and “lung FEV1/FVC ratio” (*p* = 4.81E-05, Z-score = 3.9). Furthermore, the search revealed thyroid traits associated with *GDF11* expression, such as “self-reported hypothyroidism” (*p* = 8.54E-06, Z-score = -4.3) and “hypothyroidism/myxodema” (*p* = 2.07E-05, Z-score = -4.1). All associations remained significant after a tissue-specific Bonferroni correction at an experiment-wide α of 0.05 was applied. In tandem, we mined *MSTN* TWAS results in the TWAS hub and identified 50 significant associations. Predictive models for *MSTN* expression were only available for one tissue, hypothalamus. The most significant association with *MSTN* expression in the hypothalamus was “diastolic blood pressure, automated reading” (*p* = 2.60e-12, Z-score = -6.9; Supplementary Table S14), and the disease category most frequently associated with *MSTN* expression was cardiovascular health (20.00%; Fig. 2D).

**Table 3:** Predicted tissue-specific *GDF11* expression is associated with asthma, immune function, lung function, and thyroid traits in the TWAS Hub. Associations were identified in suprapubic unexposed skin. All associations remained significant after a tissue-specific Bonferroni correction at an experiment-wide α of 0.05 was applied. All associations with predicted tissue-specific *GDF11* expression in the TWAS Hub can be found in Supplementary Table S13

Tissue	Trait	*P*-value	Z-score
Suprapubic unexposed skin	Blood eosinophil count	4.79E-07	-4.9
	Respiratory disease	1.30E-06	-4.7
	Hypothyroidism (self-reported)	8.54E-06	-4.3
	Hypothyroidism/ myxodema	2.07E-05	-4.1
	Asthma (self-reported)	3.17E-05	-4
	Lung FEV1/FVC ratio	4.81E-05	3.9
	Asthma	7.23E-05	-3.8
	Lung FVC	4.66E-03	2.6

### *HEY1*, a candidate genetic regulator of* GDF11 *expression, is associated with respiratory, immune function, and thyroid health

**Fig. 4:**
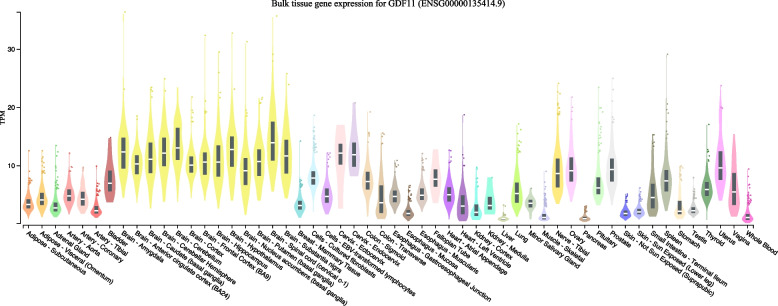
Bulk tissue gene expression for *GDF11* from the GTEx dataset. The x-axis represents separate tissues, and the y-axis represents expression values. Expression values are shown in TPM (transcripts per million), calculated from a gene model with isoforms collapsed to a single gene

In the next step of evaluating the respective effects of *GDF11* and *MSTN*, we contrasted their putative genetic regulators, *HEY1* and *FOXO1,* respectively, which we identified previously. We utilized the GTEx dataset (Supplementary Fig. S3) to contrast *GDF11* and *HEY1* expression across many tissues. *GDF11* appeared to exhibit a similar expression pattern as *HEY1* across tissues. These results indicated low *MSTN* expression levels across tissues relative to other genes in the query, and *MSTN* and *FOXO1* did not appear to follow similar expression profiles across tissues. Moreover, bulk tissue expression data from the GTEx project revealed distinct expression profiles between *GDF11* and *MSTN*. These expression data indicated the highest level of *GDF11* expression in various areas of the brain, including the cervical spinal cord, cerebellum, hypothalamus, amygdala, and spleen (Fig. 4). *GDF11*’s expression profile provided further evidence to support the health associations we uncovered; *GDF11* showed high levels of expression in the lungs and thyroid, with median bulk tissue expression levels of approximately 7 and 8 TPM, respectively. In contrast, *MSTN* exhibited the highest expression in cultured fibroblasts and skeletal muscle but had relatively low levels of expression across tissues compared to *GDF11* (Supplementary Fig. S4).

Lastly, we sought to define the extent to which health effects of *GDF11* overlapped with those of its own potential genetic regulator, *HEY1*. We conducted a PheWAS analysis of *HEY1* in GeneATLAS and identified a total of 27 health associations (Supplementary Table S15)*.* Interestingly, PheWAS results revealed associations between *HEY1* variants and respiratory and immune function traits (Table 4). The association with the highest statistical significance was between rs3888020 and “lymphocyte count” (*p* = 2.43E-18). Two other *HEY1* variants, rs4739738 and rs13263709, were associated with “asthma” (*p* = 6.40E-15 and* p* = 5.06E-12, respectively). Furthermore, we searched PhenomeXcan for TWAS results and identified associations between tissue-specific *HEY1* expression and respiratory and thyroid phenotypes (Table 5). The strongest respiratory-related association with *HEY1* expression across all tissues with available data was “diagnoses—main ICD10: J39 Other diseases of upper respiratory tract” (*p* = 2.35E-4) and was identified in the minor salivary gland tissue. Thyroid traits associated with *HEY1* expression in PhenomeXcan included “non-cancer illness code, self-reported: thyroid problem (not cancer)” identified in the minor salivary gland (*p* = 9.06E-3) and hypothalamus (*p* = 1.53E-2), and “hypothyroidism (congenital or acquired)” identified in the testis (*p* = 9.89E-3). All associations with tissue-specific *HEY1* expression identified in PhenomeXcan can be found in Supplementary Table S16. Using the GWAS Catalog, we uncovered associations between *HEY1* variants and various health outcomes, including educational attainment and colorectal cancer survival, though none of which overlapped with respiratory, lung function, immune function, or thyroid traits (Supplementary Table S17). Of note, we investigated the other *HEY* family genes and the results were consistent with those for *HEY1* (Supplementary Table S18).

**Table 4:** *HEY1* variants are associated with respiratory and immune function traits in GeneATLAS. Associations were identified using the “Region PheWAS” function in GeneATLAS. A *p*-value less than 0.05 was considered statistically significant. All associations with *HEY1* variants in GeneATLAS can be found in Supplementary Table S15

Trait	Variant	*P*-value
Lymphocyte count	rs3888020	2.43E-18
Lymphocyte percentage	rs3888020	1.87E-17
Asthma	rs4739738	6.40E-15
Neutrophil percentage	rs3888020	9.12E-15
Eosinophil percentage	rs13263709	2.73E-12
J45 Asthma	rs13263709	5.06E-12
Eosinophil count	rs13263709	5.47E-12
J40-J47 Chronic lower respiratory diseases	rs4739738	6.63E-11
J40-J47 Chronic lower respiratory diseases	rs1702877	6.63E-11
White blood cell (leukocyte) count	rs12677936	7.92E-11
Monocyte percentage	rs3888020	2.08E-10

**Table 5:** Predicted tissue-specific *HEY1* expression is associated with respiratory and thyroid traits in PhenomeXcan. The respective tissue where each association was detected is listed in the table. A *p*-value less than 0.05 was considered statistically significant. All associations with predicted tissue-specific *HEY1* expression can be found in Supplementary Table S16

Trait	Tissue	*P*-value	Z-score
Diagnoses—main ICD10: J39 Other diseases of upper respiratory tract	Minor salivary gland	2.35E-4	-3.68
Diagnoses—main ICD10: J39 Other diseases of upper respiratory tract	Pituitary; Brain_Nucleus_accumbens_basal_ganglia; Brain_Caudate_basal_ganglia; Brain_Putamen_basal_ganglia	9.08E-4	3.32
Underlying (primary) cause of death: ICD10: C34.9 Bronchus or lung, unspecified	Skeletal muscle	3.43E-3	2.93
Recent medication for asthma	Skeletal muscle	5.79E-3	2.76
Non-cancer illness code, self-reported: thyroid problem (not cancer)	Minor salivary gland	9.06E-3	-2.61
Hypothyroidism (congenital or acquired)	Testis	9.89E-3	2.58
Non-cancer illness code, self-reported: thyroid problem (not cancer)	Hypothalamus	1.53E-2	-2.42

**Fig. 5:**
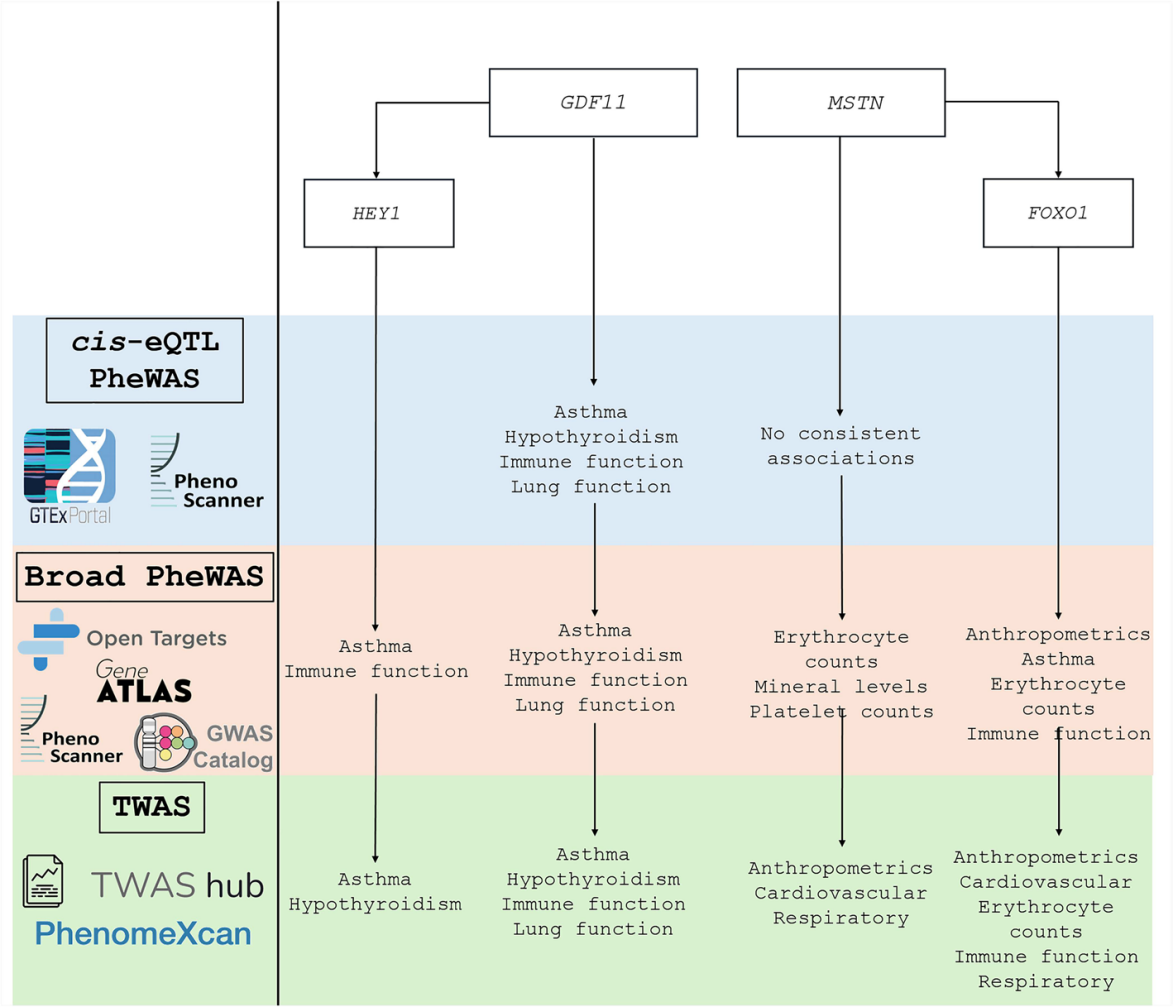
Overarching study design and results flow chart for *GDF11*, *MSTN*, *HEY1*, and *FOXO1*. Each step of the study design and corresponding databases are depicted to the left of the vertical black line. Overarching results for each gene identified from each step are depicted to the right of the vertical black line

Finally, to be as comprehensive as possible, we searched for overlap between *MSTN* and its potential genetic regulator, *FOXO1*, and identified shared associations between these genes in several databases (Supplementary Tables S19-S22). A flow chart depicting the study design and overarching results for each gene included in this study can be found in Fig. 5.

## Discussion

In the present study, we sought to define the unique health effects of GDF11 at the population level, versus that of its homolog MSTN, using repositories of published GWAS results in population biobanks. Prior studies had demonstrated roles for GDF11 in rejuvenating the aged heart [22, 100], brain [24–26], and skeletal muscle [23], so we predicted that *GDF11* variants would be associated with cardiovascular, cognitive, and muscular diseases and phenotypes. Yet our results revealed consistent relationships between *GDF11* and respiratory, immune function, and thyroid health instead. PheWAS of *GDF11 cis*-eQTLs revealed associations with asthma, lung function, immune function, and thyroid health, and PheWAS of broader *GDF11* variants and TWAS of its predicted tissue-specific expression confirmed those findings. Through our comprehensive approach, we documented novel associations with *GDF11*, expanding our knowledge of the pleiotropic health effects of this gene.

Our analysis revealed that *GDF11* variants and its predicted tissue-specific expression levels were consistently associated with asthma across multiple databases. Asthma is a chronic inflammatory lung disease characterized by airflow obstruction in which airway smooth muscle constricts due to a variety of triggers, such as allergens, tobacco smoke, air pollution, and infections [101, 102]. Asthma affects over 300 million individuals worldwide and claimed over 455,000 deaths in 2019, according to the Global Burden of Disease study [103, 104]. Asthma, like many other chronic conditions, is polygenic – driven by complex interactions among many genes and variants. Human GWAS consistently implicate the 17q21 locus with asthma, and variants of four genes within this locus have been linked to the development of the disease [105]. Mutations in these genes, including *ORMDL3*, *GSDMB*, *ZPBP2*, and *IKZF3*, result in reduced protein folding in the endoplasmic reticulum leading to an overall pro-inflammatory effect in asthma patients [106]. However, as with other complex genetic diseases, much of its heritability remains undefined [106]. In addition to the presence of asthma, in our study, *GDF11* variants and transcript levels were also associated with blood eosinophil counts. These data are supportive of a potential relationship between GDF11 and asthma due to the major role eosinophils play in asthma pathogenesis. In T2 high asthma, distinguished by eosinophilic inflammation in the airways [107], eosinophils are recruited to the inflammatory site and release inflammatory mediators such as cytokines and chemokines [108]. Furthermore, sputum and blood eosinophil counts have been used as clinical biomarkers for disease exacerbation [109–111]. In this study, we identified high *GDF11* expression levels in the spleen, consistent with findings from past studies [112, 113]. The spleen has a broad range of immunological functions and contains several types of resident immune cells, including T and B cells, dendritic cells, and macrophages [114–116]. These results suggest *GDF11* may influence the development or progression of asthma through its relationship with the immune system. Future research will expand our understanding of asthma genetics, including the relationship between *GDF11*, asthma, and the immune system.

Furthermore, our results pointed to a relationship between *GDF11* and thyroid phenotypes, particularly hypothyroidism. Hypothyroidism is a chronic disease characterized by a deficiency in thyroxine (T4) and triiodothyronine (T3) [117], with an estimated worldwide prevalence of 5% [118]. While environmental iodine deficiency is the most common cause of hypothyroidism globally, autoimmune thyroiditis (Hashimoto’s disease) is the leading cause of primary hypothyroidism in regions of iodine sufficiency [119]. Hypothyroidism results from pathological processes within the thyroid gland (primary hypothyroidism) but can also develop from hypothalamus or pituitary disorders (central hypothyroidism) or disorders of the peripheries [119, 120]. The genetic basis of hypothyroidism has yet to be well-defined [119], although GWAS have identified common loci associated with thyroid hormone regulation [121–123]. Interestingly, a recent study by Añón-Hidalgo et al. identified a positive association between GDF11 and TSH levels in humans [124], supporting the relationship we found between *GDF11* and hypothyroidism. Importantly, the top *cis-*eQTL of *GDF11*, rs117385153, was identified in the thyroid tissue, and bulk tissue expression from the GTEx dataset supports this finding, revealing moderate *GDF11* expression levels in the thyroid tissue. Collectively, these data highlight a consistency between the genetic regulation of *GDF11* and its associations with hypothyroidism, supporting the potential role of *GDF11* in the development or progression of thyroid disease.

We propose that the associations identified within this study, linking *GDF11* variants and transcript levels to respiratory and thyroid phenotypes—specifically asthma and hypothyroidism—are mediated through the involvement of GDF11 in inflammatory signaling pathways. The relationship between GDF11 and inflammation has been reported in prior studies, particularly by its attenuation of inflammatory factor expression by impeding nuclear factor kappa-light-chain-enhancer of activated B cells (NF-kB) and JNK signaling pathways through TGF-β/Smad2/3 activation [27, 124–128]. Through its anti-inflammatory actions, GDF11 has been shown to be beneficial by relieving acute lung injury [127], the development of inflammatory arthritis [125], endothelial injury and atherosclerotic lesion formation [126], and aging of the skin [27]. Moreover, prior studies in humans with chronic obstructive pulmonary disease (COPD) report decreases in circulating GDF11 levels [128, 129] and reduced *GDF11* expression in the serum and cells of these patients [130]. Asthma and hypothyroidism are complex, chronic conditions whose pathogeneses and progression are largely dictated by inflammation [131–133]. Both conditions are often diagnosed in individuals with autoimmune disorders, such as Type 1 diabetes [117, 134]. Asthma and thyroid diseases have been correlated with each other in several studies [135–146], but the research is mostly limited to association data. These reports include case studies that identify patients afflicted by both diseases [143, 142], epidemiological evidence from the Oxford Record Linkage Study (ORLS) supporting a possible association between the two conditions [137], and a population-based cohort study suggesting maternal hypothyroidism may increase the risk of childhood asthma [144]. Several studies report associations between hypothyroidism and milder asthma symptoms [140, 145, 146], potentially due to reduced thyroid hormone levels which cause decreased oxygen consumption [147]. We posit that *GDF11* influences respiratory and thyroid health, particularly asthma and hypothyroidism, through its anti-inflammatory effects. To our knowledge, no group has reported a connection between *GDF11*, asthma, and hypothyroidism simultaneously, warranting future research on the potential role of GDF11 in the pathogenesis of these conditions.

One SNP identified in Open Target Genetics, rs1689510, was found to be associated with asthma, immune function, lung function, and thyroid traits across multiple studies. PheWAS results from FinnGen, UK Biobank, and GWAS Catalog for this SNP indicate significant associations with type 1 diabetes, asthma, immune function, and hypothyroidism. These findings highlight a major advantage of PheWAS – the ability to identify a single locus that affects multiple distinct phenotypes – and provide pleiotropic variants to inform the next steps in this research. For instance, mechanistic investigations are now needed to test and validate rs1689510 as a key player in asthma pathogenesis. rs1689510 is located 259,862 bp away from *GDF11’s* canonical transcription start site (TSS), and upon further investigation, we identified this variant as a *cis-*eQTL of *GDF11* in the eQTL Gen database [92]. The nearest gene to rs1689510 is IKAROS family zinc finger 4 (*IKZF4;* 4,675 bp to canonical TSS), which encodes a protein that binds to the 5'GGGAATRCC-3' Ikaros-binding sequence and serves as a transcriptional repressor [148]. Results from the GTEx portal indicate that rs1689510 is a *cis*-eQTL of *IKZF4* in adipose tissue (*p* = 6.6E-08), lymphocytes (*p* = 1.2E-06), and unexposed suprapubic skin (*p* = 3.7E-05). Intriguingly, *IKZF4* has been shown to be necessary for the inhibitory role of T-regulatory cells [149, 150], suggesting rs1689510 may regulate the predicted expression levels of several genes involved in immune function, including *IKZF4* and *GDF11*. Overall, these results encourage future studies on the role rs1689510 plays in the immune system and disease progression and development, particularly asthma.

In our prior rodent work, we provided evidence of members of the hairy and enhancer of split-related (HESR) family of basic helix-loop-helix (bHLH) transcriptional repressors [151], specifically *Hey1*, as genetic regulators of GDF11 [51]. In the present study, gene expression results from the GTEx dataset indicate similar expression patterns for *GDF11* and *HEY1* across tissues*.* These relationships remain, although less consistently, between *GDF11* and other *HEY* family genes. These human expression data mirror those of mice, wherein *Gdf11* and *Hey* family genes are positively correlated with each other [152]. Moreover, we noted general associations between *HEY1* variants and its predicted tissue-specific expression levels with respiratory and thyroid conditions, as we did for GDF11. These results are consistent with *HEY1* as a potential genetic regulator of *GDF11,* specifically in the context of inflammatory disease. We also investigated the homolog of *GDF11*, *MSTN,* to parse out their distinct biological roles. Results mirror MSTN’s known role in the heart [153–156], as many associations were related to cardiovascular health. However, *MSTN* did not share the same robust relationship with respiratory, lung function, immune function, and thyroid traits as *GDF11* did. This suggests that *GDF11* has a separate, distinct role in the development of inflammatory diseases that is not shared with *MSTN*. Future work should continue to delineate the shared and distinct functions of these homologs.

It must be noted that there are several limitations to our study. First, we applied Bonferroni correction on many transcriptome-wide associations to establish a conservative significance threshold, but not all of our variables were truly independent [157]. TWAS results were based on genetically predicted gene expression, not direct measurements of gene expression – TWAS signals rely on the predictive power of the genetic model to compute gene expression and GWAS to source variant-level trait associations. Furthermore, our stratified approach used multiple databases to gather evidence; however, these databases were not completely independent from one another. Some of the consistent observations we identified between databases were due to the same underlying GWAS results housed in several platforms. Importantly, these databases primarily include British individuals, which affords them limited ancestral diversity. It is imperative that future studies investigate these genotype–phenotype relationships in populations with greater genetic diversity. Lastly, due to incomplete linkage disequilibrium (LD) between SNPs and the availability of SNPs in the databases used in this study, we did observe trait associations for some SNPs within LD blocks, but not others. In the interest of communicating the novel relationships identified between *GDF11* and health, we included all reported significant associations to be comprehensive.

In this study, we provide evidence of *GDF11* in the involvement of inflammatory diseases, namely asthma and hypothyroidism. PheWAS revealed robust associations between *GDF11 cis*-eQTLs and asthma, immune function, lung function, and thyroid health. Associations identified from PheWAS on *GDF11* variants and TWAS on predicted *GDF11* tissue-specific expression confirmed our findings. Secondarily, we found similar health associations with *HEY1* as we did with *GDF11*, supporting our previous work which identified *HEY1* as a candidate genetic regulator of *GDF11* in the DO mouse stock. Moreover, gene expression data strengthen our hypotheses that *GDF11* is involved in respiratory and thyroid disease pathogenesis and that *HEY1* regulates *GDF11*. Through these efforts, we report novel relationships between *GDF11* and disease, suggesting GDF11 may exert its effects by acting on inflammatory pathways, in contrast to its formerly assumed role as a rejuvenating factor in basic aging. These data provide novel insights into the health impacts of *GDF11* and lend further support for future mechanistic studies to illuminate the precise role of GDF11 in inflammatory disease pathogenesis.

## Supplementary Information

Supplementary Material 1.

## Data Availability

All data analyzed during this study are included in this published article and its supplementary information files. All data was downloaded from open-access databases, and details with links can be found in references [ 79, 80, 89, 92, 100, 102].

## Abbreviations

DO: Diversity Outbred
FoxO1: Forkhead Box O1
GDF11: Growth differentiation factor 11
Hey1: Hes Related Family BHLH Transcription Factor with YRPW Motif 1
MSTN: Myostatin
PheWAS: Phenome-wide association study
SNP: Single nucleotide polymorphism
TWAS: Transcriptome-wide association study
TGF-β: Transforming growth factor-β
