# Supplementary material for: Novel insights into the pleiotropic health effects of growth differentiation factor 11 gained from genome-wide association studies in population biobanks
Source: BMC Genomics. 2024 Sep 6;25:837. doi: 10.1186/s12864-024-10710-7 (PMC11378601; doi:10.1186/s12864-024-10710-7)
Supplement: Supplementary file 1 — Supplementary Material 1. [file 12864_2024_10710_MOESM1_ESM.docx]

SUPPLEMENTARY INFORMATION

| **Gencode Id** | **Gene Symbol** | **Variant Id** | **SNP Id** | **P-Value** | **NES** | **Tissue** |
| --- | --- | --- | --- | --- | --- | --- |
| ENSG00000135414.9 | GDF11 | chr12_55746802_T_A_b38 | rs117385153 | 6.3E-07 | 0.56 | Thyroid |
| ENSG00000135414.9 | GDF11 | chr12_55826821_C_G_b38 | rs138611520 | 6.3E-07 | 0.56 | Thyroid |
| ENSG00000135414.9 | GDF11 | chr12_55837573_T_C_b38 | rs17844804 | 6.3E-07 | 0.56 | Thyroid |
| ENSG00000135414.9 | GDF11 | chr12_55835907_G_GC_b38 | rs17844808 | 0.0000015 | 0.53 | Thyroid |
| ENSG00000135414.9 | GDF11 | chr12_55821332_G_A_b38 | rs79015557 | 0.0000015 | 0.52 | Thyroid |
| ENSG00000135414.9 | GDF11 | chr12_55718000_TC_T_b38 | rs145322257 | 0.0000023 | 0.52 | Thyroid |
| ENSG00000135414.9 | GDF11 | chr12_55695560_A_G_b38 | rs7971022 | 0.0000029 | 0.48 | Thyroid |
| ENSG00000135414.9 | GDF11 | chr12_55699080_C_G_b38 | rs118129867 | 0.0000029 | 0.48 | Thyroid |
| ENSG00000135414.9 | GDF11 | chr12_56080024_T_C_b38 | rs7297175 | 0.0000068 | 0.14 | Muscle - Skeletal |
| ENSG00000135414.9 | GDF11 | chr12_56083910_A_T_b38 | rs2271194 | 0.000011 | 0.14 | Muscle - Skeletal |
| ENSG00000135414.9 | GDF11 | chr12_55722001_C_T_b38 | rs3138140 | 0.000015 | 0.37 | Brain - Cerebellum |
| ENSG00000135414.9 | GDF11 | chr12_55722099_T_C_b38 | rs3138139 | 0.000015 | 0.37 | Brain - Cerebellum |
| ENSG00000135414.9 | GDF11 | chr12_55050814_C_CTGTGT_b38 | rs879920435 | 0.000015 | -0.73 | Testis |
| ENSG00000135414.9 | GDF11 | chr12_55046394_A_G_b38 | rs114387148 | 0.000016 | -0.73 | Testis |
| ENSG00000135414.9 | GDF11 | chr12_55048626_A_G_b38 | rs77543972 | 0.000016 | -0.73 | Testis |
| ENSG00000135414.9 | GDF11 | chr12_55048675_G_A_b38 | rs77755167 | 0.000016 | -0.73 | Testis |
| ENSG00000135414.9 | GDF11 | chr12_55048879_C_CA_b38 | rs149004057 | 0.000016 | -0.73 | Testis |
| ENSG00000135414.9 | GDF11 | chr12_55049092_G_C_b38 | rs76519671 | 0.000016 | -0.73 | Testis |
| ENSG00000135414.9 | GDF11 | chr12_55049312_C_T_b38 | rs75332788 | 0.000016 | -0.73 | Testis |
| ENSG00000135414.9 | GDF11 | chr12_55050365_T_G_b38 | rs17753819 | 0.000016 | -0.73 | Testis |
| ENSG00000135414.9 | GDF11 | chr12_55052159_C_T_b38 | rs114065808 | 0.000016 | -0.73 | Testis |
| ENSG00000135414.9 | GDF11 | chr12_55609562_T_A_b38 | rs191460468 | 0.000017 | 0.52 | Thyroid |
| ENSG00000135414.9 | GDF11 | chr12_56076696_C_T_b38 | rs12230102 | 0.000021 | 0.14 | Muscle - Skeletal |
| ENSG00000135414.9 | GDF11 | chr12_56084218_A_C_b38 | rs10876870 | 0.000021 | 0.13 | Muscle - Skeletal |
| ENSG00000135414.9 | GDF11 | chr12_55046395_A_G_b38 | rs77505923 | 0.000023 | -0.68 | Testis |
| ENSG00000135414.9 | GDF11 | chr12_55046446_C_T_b38 | rs77065430 | 0.000023 | -0.68 | Testis |
| ENSG00000135414.9 | GDF11 | chr12_55046454_A_G_b38 | rs76657475 | 0.000023 | -0.68 | Testis |
| ENSG00000135414.9 | GDF11 | chr12_55046508_G_A_b38 | rs79491138 | 0.000023 | -0.68 | Testis |
| ENSG00000135414.9 | GDF11 | chr12_55047186_G_T_b38 | rs117051050 | 0.000023 | -0.68 | Testis |
| ENSG00000135414.9 | GDF11 | chr12_55047225_C_T_b38 | rs150441588 | 0.000023 | -0.68 | Testis |
| ENSG00000135414.9 | GDF11 | chr12_55047623_C_T_b38 | rs145480060 | 0.000023 | -0.68 | Testis |
| ENSG00000135414.9 | GDF11 | chr12_56080696_A_G_b38 | rs4759229 | 0.000023 | 0.14 | Muscle - Skeletal |
| ENSG00000135414.9 | GDF11 | chr12_56086864_G_A_b38 | rs705696 | 0.000028 | -0.14 | Muscle - Skeletal |
| ENSG00000135414.9 | GDF11 | chr12_56088396_T_G_b38 | rs2292239 | 0.000030 | 0.13 | Muscle - Skeletal |
| ENSG00000135414.9 | GDF11 | chr12_55905111_C_G_b38 | rs561294154 | 0.000032 | 0.50 | Thyroid |
| ENSG00000135414.9 | GDF11 | chr12_55575881_AG_A_b38 | rs59980219 | 0.000036 | -0.23 | Artery - Aorta |
| ENSG00000135414.9 | GDF11 | chr12_55578135_C_T_b38 | rs55685136 | 0.000036 | -0.23 | Artery - Aorta |
| ENSG00000135414.9 | GDF11 | chr12_55578787_T_C_b38 | rs56315406 | 0.000036 | -0.23 | Artery - Aorta |
| ENSG00000135414.9 | GDF11 | chr12_55056934_C_A_b38 | rs114417122 | 0.000037 | -0.73 | Testis |
| ENSG00000135414.9 | GDF11 | chr12_55060015_G_A_b38 | rs11610945 | 0.000037 | -0.73 | Testis |
| ENSG00000135414.9 | GDF11 | chr12_55065931_A_C_b38 | rs17754152 | 0.000037 | -0.73 | Testis |
| ENSG00000135414.9 | GDF11 | chr12_56073803_C_T_b38 | rs7312770 | 0.000039 | 0.12 | Muscle - Skeletal |
| ENSG00000135414.9 | GDF11 | chr12_56009793_C_T_b38 | rs772921 | 0.000039 | -0.14 | Muscle - Skeletal |
| ENSG00000135414.9 | GDF11 | chr12_56034024_C_T_b38 | rs1702877 | 0.000039 | -0.14 | Muscle - Skeletal |
| ENSG00000135414.9 | GDF11 | chr12_56073803_C_T_b38 | rs7312770 | 0.000039 | 0.14 | Skin - Not Sun Exposed (Suprapubic) |
| ENSG00000135414.9 | GDF11 | chr12_54912034_C_T_b38 | rs76779798 | 0.000041 | 0.31 | Whole Blood |
| ENSG00000135414.9 | GDF11 | chr12_56018703_T_G_b38 | rs1701704 | 0.000046 | -0.14 | Muscle - Skeletal |
| ENSG00000135414.9 | GDF11 | chr12_56072689_C_T_b38 | rs2640562 | 0.000046 | -0.13 | Muscle - Skeletal |
| ENSG00000135414.9 | GDF11 | chr12_56080595_T_C_b38 | rs3741499 | 0.000053 | 0.13 | Muscle - Skeletal |
| ENSG00000135414.9 | GDF11 | chr12_55730556_G_GA_b38 | rs59962788 | 0.000053 | 0.34 | Brain - Cerebellum |
| ENSG00000135414.9 | GDF11 | chr12_54899536_A_G_b38 | rs79947170 | 0.000056 | 0.33 | Whole Blood |
| ENSG00000135414.9 | GDF11 | chr12_54900612_T_A_b38 | rs144822395 | 0.000056 | 0.33 | Whole Blood |
| ENSG00000135414.9 | GDF11 | chr12_54900684_T_A_b38 | rs148517445 | 0.000056 | 0.33 | Whole Blood |
| ENSG00000135414.9 | GDF11 | chr12_54900781_C_T_b38 | rs142440779 | 0.000056 | 0.33 | Whole Blood |
| ENSG00000135414.9 | GDF11 | chr12_54901647_C_T_b38 | rs145231839 | 0.000056 | 0.33 | Whole Blood |
| ENSG00000135414.9 | GDF11 | chr12_54903294_T_C_b38 | rs145752677 | 0.000056 | 0.33 | Whole Blood |
| ENSG00000135414.9 | GDF11 | chr12_54904626_C_T_b38 | rs148126933 | 0.000056 | 0.33 | Whole Blood |
| ENSG00000135414.9 | GDF11 | chr12_54909864_G_T_b38 | rs145470854 | 0.000056 | 0.33 | Whole Blood |
| ENSG00000135414.9 | GDF11 | chr12_54911368_C_T_b38 | rs75249636 | 0.000056 | 0.33 | Whole Blood |
| ENSG00000135414.9 | GDF11 | chr12_54911556_T_A_b38 | rs75237836 | 0.000056 | 0.33 | Whole Blood |
| ENSG00000135414.9 | GDF11 | chr12_54917039_C_A_b38 | rs78872239 | 0.000056 | 0.33 | Whole Blood |
| ENSG00000135414.9 | GDF11 | chr12_56041628_G_A_b38 | rs705704 | 0.000062 | -0.13 | Muscle - Skeletal |
| ENSG00000135414.9 | GDF11 | chr12_55929798_T_A_b38 | rs7306585 | 0.000062 | 0.41 | Thyroid |
| ENSG00000135414.9 | GDF11 | chr12_56028855_CGTG_C_b38 | rs34813703 | 0.000063 | -0.13 | Muscle - Skeletal |
| ENSG00000135414.9 | GDF11 | chr12_56076841_C_T_b38 | rs11171739 | 0.000065 | 0.12 | Muscle - Skeletal |
| ENSG00000135414.9 | GDF11 | chr12_55645543_T_G_b38 | rs74388028 | 0.000067 | 0.42 | Thyroid |
| ENSG00000135414.9 | GDF11 | chr12_56055651_G_A_b38 | rs7302200 | 0.000070 | -0.13 | Muscle - Skeletal |
| ENSG00000135414.9 | GDF11 | chr12_56023144_A_C_b38 | rs2456973 | 0.000072 | -0.13 | Muscle - Skeletal |
| ENSG00000135414.9 | GDF11 | chr12_56050848_C_T_b38 | rs34415530 | 0.000073 | -0.13 | Muscle - Skeletal |
| ENSG00000135414.9 | GDF11 | chr12_56051582_G_A_b38 | rs10876866 | 0.000073 | -0.13 | Muscle - Skeletal |
| ENSG00000135414.9 | GDF11 | chr12_56052982_C_T_b38 | rs61938962 | 0.000073 | -0.13 | Muscle - Skeletal |
| ENSG00000135414.9 | GDF11 | chr12_56053020_C_T_b38 | rs61938963 | 0.000073 | -0.13 | Muscle - Skeletal |
| ENSG00000135414.9 | GDF11 | chr12_56056323_GA_G_b38 | rs141490969 | 0.000073 | -0.13 | Muscle - Skeletal |
| ENSG00000135414.9 | GDF11 | chr12_55985643_G_C_b38 | rs1873914 | 0.000087 | -0.12 | Muscle - Skeletal |
| ENSG00000135414.9 | GDF11 | chr12_55727665_G_C_b38 | rs3138132 | 0.000087 | 0.34 | Brain - Cerebellum |
| ENSG00000135414.9 | GDF11 | chr12_56042145_C_G_b38 | rs1131017 | 0.000093 | 0.12 | Muscle - Skeletal |
| ENSG00000135414.9 | GDF11 | chr12_56086799_G_A_b38 | rs877636 | 0.00010 | 0.12 | Muscle - Skeletal |
| ENSG00000135414.9 | GDF11 | chr12_56061510_C_T_b38 | rs2640564 | 0.00011 | -0.13 | Muscle - Skeletal |
| ENSG00000135414.9 | GDF11 | chr12_55569944_C_T_b38 | rs117718204 | 0.00011 | 0.41 | Thyroid |
| ENSG00000135414.9 | GDF11 | chr12_56074922_A_T_b38 | rs7955865 | 0.00011 | 0.12 | Muscle - Skeletal |
| ENSG00000135414.9 | GDF11 | chr12_56002984_G_C_b38 | rs1689510 | 0.00011 | -0.13 | Muscle - Skeletal |
| ENSG00000135414.9 | GDF11 | chr12_55067921_A_C_b38 | rs115858907 | 0.00013 | -0.83 | Testis |
| ENSG00000135414.9 | GDF11 | chr12_55068687_G_A_b38 | rs76490331 | 0.00013 | -0.83 | Testis |
| ENSG00000135414.9 | GDF11 | chr12_55068830_G_A_b38 | rs115392269 | 0.00013 | -0.83 | Testis |
| ENSG00000135414.9 | GDF11 | chr12_56468936_C_G_b38 | rs138855722 | 0.00014 | 0.42 | Artery - Aorta |
| ENSG00000135414.9 | GDF11 | chr12_55996580_C_G_b38 | rs772920 | 0.00017 | -0.13 | Muscle - Skeletal |
| ENSG00000135414.9 | GDF11 | chr12_55996852_A_G_b38 | rs705702 | 0.00017 | -0.13 | Muscle - Skeletal |
| ENSG00000135414.9 | GDF11 | chr12_56421933_T_C_b38 | rs61733875 | 0.00017 | -0.45 | Muscle - Skeletal |
| ENSG00000135414.9 | GDF11 | chr12_56428906_C_T_b38 | rs11832580 | 0.00017 | -0.45 | Muscle - Skeletal |
| ENSG00000135414.9 | GDF11 | chr12_56431004_T_C_b38 | rs6581093 | 0.00017 | -0.45 | Muscle - Skeletal |
| ENSG00000135414.9 | GDF11 | chr12_55502607_T_C_b38 | rs73341456 | 0.00020 | 0.39 | Thyroid |
| ENSG00000135414.9 | GDF11 | chr12_55502786_T_A_b38 | rs74092350 | 0.00020 | 0.39 | Thyroid |
| ENSG00000135414.9 | GDF11 | chr12_55504916_G_T_b38 | rs58459351 | 0.00020 | 0.39 | Thyroid |
| ENSG00000135414.9 | GDF11 | chr12_55508368_T_C_b38 | rs57085131 | 0.00020 | 0.39 | Thyroid |
| ENSG00000135414.9 | GDF11 | chr12_55509091_G_T_b38 | rs4286462 | 0.00020 | 0.39 | Thyroid |
| ENSG00000135414.9 | GDF11 | chr12_55509353_T_C_b38 | rs138809713 | 0.00020 | 0.39 | Thyroid |
| ENSG00000135414.9 | GDF11 | chr12_55510677_C_T_b38 | rs73324262 | 0.00020 | 0.39 | Thyroid |
| ENSG00000135414.9 | GDF11 | chr12_55516582_A_G_b38 | rs4597757 | 0.00020 | 0.39 | Thyroid |
| ENSG00000135414.9 | GDF11 | chr12_55519029_A_C_b38 | rs139063414 | 0.00020 | 0.39 | Thyroid |
| ENSG00000135414.9 | GDF11 | chr12_55520496_C_A_b38 | rs563559475 | 0.00020 | 0.39 | Thyroid |
| ENSG00000135414.9 | GDF11 | chr12_55520669_A_G_b38 | rs569485563 | 0.00020 | 0.39 | Thyroid |
| ENSG00000135414.9 | GDF11 | chr12_55976127_A_G_b38 | rs773108 | 0.00021 | -0.12 | Muscle - Skeletal |
| ENSG00000135414.9 | GDF11 | chr12_55981353_C_G_b38 | rs773110 | 0.00021 | -0.12 | Muscle - Skeletal |
| ENSG00000135414.9 | GDF11 | chr12_55981956_C_A_b38 | rs773111 | 0.00021 | -0.12 | Muscle - Skeletal |
| ENSG00000135414.9 | GDF11 | chr12_55986335_T_A_b38 | rs2048036 | 0.00021 | -0.12 | Muscle - Skeletal |
| ENSG00000135414.9 | GDF11 | chr12_55987700_A_C_b38 | rs61937247 | 0.00021 | -0.12 | Muscle - Skeletal |
| ENSG00000135414.9 | GDF11 | chr12_55988132_A_G_b38 | rs61937249 | 0.00021 | -0.12 | Muscle - Skeletal |
| ENSG00000135414.9 | GDF11 | chr12_55982097_C_T_b38 | rs773112 | 0.00023 | -0.12 | Muscle - Skeletal |
| ENSG00000135414.9 | GDF11 | chr12_55987921_C_T_b38 | rs61937248 | 0.00024 | -0.12 | Muscle - Skeletal |
| ENSG00000135414.9 | GDF11 | chr12_56361274_A_G_b38 | rs4301822 | 0.00025 | 0.36 | Thyroid |

**Supplementary Table S1. Significant single-tissue *cis*-eQTLs of *GDF11* in the GTEx project.** A p-value less than 1.0 x 10^-5^ was considered statistically significant.

| **Samples** | **All** | **Female** | **Male** |
| --- | --- | --- | --- |
| **Total** | 604 | 193 | 411 |
| **With Genotype** | 517 | 169 | 348 |

**Supplementary Table S2. Sample count in Skin – Not Sun Exposed (Suprapubic) tissue in the GTEx project.** The sample was taken from the extension of the abdominal incision to the suprapubic area avoiding pubic hair.

| **Gencode Id** | **Gene Symbol** | **Variant Id** | **SNP Id** | **P-Value** | **NES** | **Tissue** |
| --- | --- | --- | --- | --- | --- | --- |
| ENSG00000138379.4 | MSTN | chr2_190204504_A_G_b38 | rs12618060 | 2.4E-07 | -0.32 | Skin - Sun Exposed (Lower leg) |
| ENSG00000138379.4 | MSTN | chr2_190310913_GT_G_b38 | rs36074132 | 3.1E-07 | 0.28 | Thyroid |
| ENSG00000138379.4 | MSTN | chr2_190067353_T_C_b38 | rs7571669 | 3.6E-07 | -0.36 | Brain - Cerebellum |
| ENSG00000138379.4 | MSTN | chr2_190070148_C_T_b38 | rs13420500 | 3.6E-07 | -0.36 | Brain - Cerebellum |
| ENSG00000138379.4 | MSTN | chr2_190072692_A_C_b38 | rs7603078 | 3.6E-07 | -0.36 | Brain - Cerebellum |
| ENSG00000138379.4 | MSTN | chr2_190369464_C_A_b38 | rs1108939 | 4.2E-07 | -0.31 | Skin - Sun Exposed (Lower leg) |
| ENSG00000138379.4 | MSTN | chr2_190329955_C_T_b38 | rs12615897 | 6.6E-07 | -0.30 | Thyroid |
| ENSG00000138379.4 | MSTN | chr2_190334828_C_T_b38 | rs62182001 | 6.6E-07 | -0.30 | Thyroid |
| ENSG00000138379.4 | MSTN | chr2_190315381_A_C_b38 | rs1547460 | 7.7E-07 | -0.30 | Thyroid |
| ENSG00000138379.4 | MSTN | chr2_190320999_G_T_b38 | rs1372053 | 7.7E-07 | -0.30 | Thyroid |
| ENSG00000138379.4 | MSTN | chr2_190251712_C_CAT_b38 | rs3838547 | 8.2E-07 | 0.23 | Skin - Sun Exposed (Lower leg) |
| ENSG00000138379.4 | MSTN | chr2_190300671_T_C_b38 | rs4853691 | 9.7E-07 | -0.29 | Thyroid |
| ENSG00000138379.4 | MSTN | chr2_190301433_A_G_b38 | rs12467835 | 9.7E-07 | -0.29 | Thyroid |
| ENSG00000138379.4 | MSTN | chr2_190324913_T_C_b38 | rs62181998 | 0.0000010 | -0.29 | Thyroid |
| ENSG00000138379.4 | MSTN | chr2_190326122_C_T_b38 | rs16832572 | 0.0000010 | -0.29 | Thyroid |
| ENSG00000138379.4 | MSTN | chr2_190326187_G_A_b38 | rs62181999 | 0.0000010 | -0.29 | Thyroid |
| ENSG00000138379.4 | MSTN | chr2_190331137_A_G_b38 | rs59696237 | 0.0000010 | -0.29 | Thyroid |
| ENSG00000138379.4 | MSTN | chr2_190333185_G_A_b38 | rs76736746 | 0.0000010 | -0.29 | Thyroid |
| ENSG00000138379.4 | MSTN | chr2_190333246_G_A_b38 | rs77225909 | 0.0000010 | -0.29 | Thyroid |
| ENSG00000138379.4 | MSTN | chr2_190071862_G_A_b38 | rs10804031 | 0.0000010 | 0.35 | Brain - Cerebellum |
| ENSG00000138379.4 | MSTN | chr2_190280481_C_T_b38 | rs1876876 | 0.0000011 | -0.29 | Thyroid |
| ENSG00000138379.4 | MSTN | chr2_190286130_C_T_b38 | rs12464840 | 0.0000011 | -0.29 | Thyroid |
| ENSG00000138379.4 | MSTN | chr2_190286626_G_A_b38 | rs62184406 | 0.0000011 | -0.29 | Thyroid |
| ENSG00000138379.4 | MSTN | chr2_190291627_T_C_b38 | rs62184432 | 0.0000011 | -0.29 | Thyroid |
| ENSG00000138379.4 | MSTN | chr2_190291698_T_C_b38 | rs35334255 | 0.0000011 | -0.29 | Thyroid |
| ENSG00000138379.4 | MSTN | chr2_190292456_C_T_b38 | rs62184435 | 0.0000011 | -0.29 | Thyroid |
| ENSG00000138379.4 | MSTN | chr2_190313593_C_T_b38 | rs10931449 | 0.0000011 | -0.29 | Thyroid |
| ENSG00000138379.4 | MSTN | chr2_190007690_G_T_b38 | rs72907385 | 0.0000011 | -0.40 | Brain - Cerebellum |
| ENSG00000138379.4 | MSTN | chr2_190300671_T_C_b38 | rs4853691 | 0.0000011 | -0.30 | Skin - Sun Exposed (Lower leg) |
| ENSG00000138379.4 | MSTN | chr2_190301433_A_G_b38 | rs12467835 | 0.0000011 | -0.30 | Skin - Sun Exposed (Lower leg) |
| ENSG00000138379.4 | MSTN | chr2_190045833_T_C_b38 | rs13401889 | 0.0000012 | -0.36 | Brain - Cerebellum |
| ENSG00000138379.4 | MSTN | chr2_190222714_G_A_b38 | rs12472258 | 0.0000012 | -0.29 | Thyroid |
| ENSG00000138379.4 | MSTN | chr2_190268043_A_G_b38 | rs3791799 | 0.0000012 | -0.30 | Skin - Sun Exposed (Lower leg) |
| ENSG00000138379.4 | MSTN | chr2_190275282_T_C_b38 | rs76451356 | 0.0000012 | -0.30 | Skin - Sun Exposed (Lower leg) |
| ENSG00000138379.4 | MSTN | chr2_190282369_G_A_b38 | rs59090097 | 0.0000012 | -0.30 | Skin - Sun Exposed (Lower leg) |
| ENSG00000138379.4 | MSTN | chr2_190283149_T_C_b38 | rs10931447 | 0.0000012 | -0.30 | Skin - Sun Exposed (Lower leg) |
| ENSG00000138379.4 | MSTN | chr2_190303188_T_G_b38 | rs12466290 | 0.0000012 | -0.30 | Skin - Sun Exposed (Lower leg) |
| ENSG00000138379.4 | MSTN | chr2_190222714_G_A_b38 | rs12472258 | 0.0000012 | -0.30 | Skin - Sun Exposed (Lower leg) |
| ENSG00000138379.4 | MSTN | chr2_190235233_A_G_b38 | rs4853503 | 0.0000013 | 0.23 | Skin - Sun Exposed (Lower leg) |
| ENSG00000138379.4 | MSTN | chr2_190244722_G_A_b38 | rs7582209 | 0.0000013 | 0.23 | Skin - Sun Exposed (Lower leg) |
| ENSG00000138379.4 | MSTN | chr2_190298534_G_A_b38 | rs62184436 | 0.0000013 | -0.29 | Thyroid |
| ENSG00000138379.4 | MSTN | chr2_190233602_A_G_b38 | rs6737514 | 0.0000014 | 0.23 | Skin - Sun Exposed (Lower leg) |
| ENSG00000138379.4 | MSTN | chr2_190016108_G_C_b38 | rs55838368 | 0.0000014 | -0.38 | Brain - Cerebellum |
| ENSG00000138379.4 | MSTN | chr2_190058686_A_G_b38 | rs7570532 | 0.0000014 | -0.35 | Brain - Cerebellum |
| ENSG00000138379.4 | MSTN | chr2_190316371_G_A_b38 | rs2055825 | 0.0000014 | -0.29 | Thyroid |
| ENSG00000138379.4 | MSTN | chr2_190268043_A_G_b38 | rs3791799 | 0.0000014 | -0.29 | Thyroid |
| ENSG00000138379.4 | MSTN | chr2_190275282_T_C_b38 | rs76451356 | 0.0000014 | -0.29 | Thyroid |
| ENSG00000138379.4 | MSTN | chr2_190282369_G_A_b38 | rs59090097 | 0.0000014 | -0.29 | Thyroid |
| ENSG00000138379.4 | MSTN | chr2_190283149_T_C_b38 | rs10931447 | 0.0000014 | -0.29 | Thyroid |
| ENSG00000138379.4 | MSTN | chr2_190303188_T_G_b38 | rs12466290 | 0.0000014 | -0.29 | Thyroid |
| ENSG00000138379.4 | MSTN | chr2_190364474_G_A_b38 | rs62182028 | 0.0000014 | -0.30 | Skin - Sun Exposed (Lower leg) |
| ENSG00000138379.4 | MSTN | chr2_189945336_A_C_b38 | rs1454301 | 0.0000015 | -0.38 | Brain - Cerebellum |
| ENSG00000138379.4 | MSTN | chr2_190292176_T_G_b38 | rs12621421 | 0.0000016 | -0.29 | Thyroid |
| ENSG00000138379.4 | MSTN | chr2_190324913_T_C_b38 | rs62181998 | 0.0000016 | -0.29 | Skin - Sun Exposed (Lower leg) |
| ENSG00000138379.4 | MSTN | chr2_190326122_C_T_b38 | rs16832572 | 0.0000016 | -0.29 | Skin - Sun Exposed (Lower leg) |
| ENSG00000138379.4 | MSTN | chr2_190326187_G_A_b38 | rs62181999 | 0.0000016 | -0.29 | Skin - Sun Exposed (Lower leg) |
| ENSG00000138379.4 | MSTN | chr2_190331137_A_G_b38 | rs59696237 | 0.0000016 | -0.29 | Skin - Sun Exposed (Lower leg) |
| ENSG00000138379.4 | MSTN | chr2_190333185_G_A_b38 | rs76736746 | 0.0000016 | -0.29 | Skin - Sun Exposed (Lower leg) |
| ENSG00000138379.4 | MSTN | chr2_190333246_G_A_b38 | rs77225909 | 0.0000016 | -0.29 | Skin - Sun Exposed (Lower leg) |
| ENSG00000138379.4 | MSTN | chr2_190251121_T_C_b38 | rs713427 | 0.0000016 | -0.28 | Thyroid |
| ENSG00000138379.4 | MSTN | chr2_190202187_T_C_b38 | rs3749015 | 0.0000017 | 0.23 | Skin - Sun Exposed (Lower leg) |
| ENSG00000138379.4 | MSTN | chr2_190204708_G_A_b38 | rs1058230 | 0.0000017 | 0.23 | Skin - Sun Exposed (Lower leg) |
| ENSG00000138379.4 | MSTN | chr2_190204750_C_T_b38 | rs1058227 | 0.0000017 | 0.23 | Skin - Sun Exposed (Lower leg) |
| ENSG00000138379.4 | MSTN | chr2_190352531_A_G_b38 | rs2016037 | 0.0000017 | -0.23 | Skin - Sun Exposed (Lower leg) |
| ENSG00000138379.4 | MSTN | chr2_190343621_G_A_b38 | rs1372054 | 0.0000019 | -0.22 | Skin - Sun Exposed (Lower leg) |
| ENSG00000138379.4 | MSTN | chr2_190251121_T_C_b38 | rs713427 | 0.0000022 | -0.29 | Skin - Sun Exposed (Lower leg) |
| ENSG00000138379.4 | MSTN | chr2_190066710_T_C_b38 | rs4853626 | 0.0000022 | -0.34 | Brain - Cerebellum |
| ENSG00000138379.4 | MSTN | chr2_190219148_C_CA_b38 | rs34447671 | 0.0000023 | 0.22 | Skin - Sun Exposed (Lower leg) |
| ENSG00000138379.4 | MSTN | chr2_190064356_C_G_b38 | rs3762546 | 0.0000023 | -0.33 | Brain - Cerebellum |
| ENSG00000138379.4 | MSTN | chr2_190304977_TCTAA_T_b38 | rs138777105 | 0.0000024 | -0.23 | Skin - Sun Exposed (Lower leg) |
| ENSG00000138379.4 | MSTN | chr2_190299502_G_GAT_b38 | rs74420213 | 0.0000028 | -0.23 | Skin - Sun Exposed (Lower leg) |
| ENSG00000138379.4 | MSTN | chr2_190354389_T_TTCA_b38 | rs3064189 | 0.0000028 | 0.23 | Skin - Sun Exposed (Lower leg) |
| ENSG00000138379.4 | MSTN | chr2_190200869_G_T_b38 | rs2271270 | 0.0000029 | 0.22 | Skin - Sun Exposed (Lower leg) |
| ENSG00000138379.4 | MSTN | chr2_190284343_A_C_b38 | rs3791800 | 0.0000030 | 0.24 | Skin - Sun Exposed (Lower leg) |
| ENSG00000138379.4 | MSTN | chr2_190288825_A_C_b38 | rs2664267 | 0.0000030 | -0.23 | Skin - Sun Exposed (Lower leg) |
| ENSG00000138379.4 | MSTN | chr2_190315381_A_C_b38 | rs1547460 | 0.0000030 | -0.29 | Skin - Sun Exposed (Lower leg) |
| ENSG00000138379.4 | MSTN | chr2_190320999_G_T_b38 | rs1372053 | 0.0000030 | -0.29 | Skin - Sun Exposed (Lower leg) |
| ENSG00000138379.4 | MSTN | chr2_189940874_G_C_b38 | rs144238103 | 0.0000030 | -0.38 | Brain - Cerebellum |
| ENSG00000138379.4 | MSTN | chr2_189946601_T_C_b38 | rs72907308 | 0.0000030 | -0.38 | Brain - Cerebellum |
| ENSG00000138379.4 | MSTN | chr2_189949739_C_G_b38 | rs112138500 | 0.0000030 | -0.38 | Brain - Cerebellum |
| ENSG00000138379.4 | MSTN | chr2_189983602_A_T_b38 | rs16832209 | 0.0000030 | -0.38 | Brain - Cerebellum |
| ENSG00000138379.4 | MSTN | chr2_189984486_T_C_b38 | rs11688447 | 0.0000030 | -0.38 | Brain - Cerebellum |
| ENSG00000138379.4 | MSTN | chr2_190370015_G_A_b38 | rs4853695 | 0.0000030 | -0.28 | Skin - Sun Exposed (Lower leg) |
| ENSG00000138379.4 | MSTN | chr2_190224215_C_T_b38 | rs13416673 | 0.0000030 | 0.22 | Skin - Sun Exposed (Lower leg) |
| ENSG00000138379.4 | MSTN | chr2_190305222_G_A_b38 | rs291436 | 0.0000031 | -0.22 | Skin - Sun Exposed (Lower leg) |
| ENSG00000138379.4 | MSTN | chr2_190204504_A_G_b38 | rs12618060 | 0.0000031 | -0.27 | Thyroid |
| ENSG00000138379.4 | MSTN | chr2_190280481_C_T_b38 | rs1876876 | 0.0000032 | -0.29 | Skin - Sun Exposed (Lower leg) |
| ENSG00000138379.4 | MSTN | chr2_190286130_C_T_b38 | rs12464840 | 0.0000032 | -0.29 | Skin - Sun Exposed (Lower leg) |
| ENSG00000138379.4 | MSTN | chr2_190286626_G_A_b38 | rs62184406 | 0.0000032 | -0.29 | Skin - Sun Exposed (Lower leg) |
| ENSG00000138379.4 | MSTN | chr2_190291627_T_C_b38 | rs62184432 | 0.0000032 | -0.29 | Skin - Sun Exposed (Lower leg) |
| ENSG00000138379.4 | MSTN | chr2_190291698_T_C_b38 | rs35334255 | 0.0000032 | -0.29 | Skin - Sun Exposed (Lower leg) |
| ENSG00000138379.4 | MSTN | chr2_190292456_C_T_b38 | rs62184435 | 0.0000032 | -0.29 | Skin - Sun Exposed (Lower leg) |
| ENSG00000138379.4 | MSTN | chr2_190313593_C_T_b38 | rs10931449 | 0.0000032 | -0.29 | Skin - Sun Exposed (Lower leg) |
| ENSG00000138379.4 | MSTN | chr2_190344069_C_T_b38 | rs760307 | 0.0000032 | 0.24 | Skin - Sun Exposed (Lower leg) |
| ENSG00000138379.4 | MSTN | chr2_190354888_G_A_b38 | rs3791815 | 0.0000032 | 0.23 | Skin - Sun Exposed (Lower leg) |
| ENSG00000138379.4 | MSTN | chr2_190355612_C_A_b38 | rs72627906 | 0.0000032 | 0.23 | Skin - Sun Exposed (Lower leg) |
| ENSG00000138379.4 | MSTN | chr2_190360026_T_G_b38 | rs2067402 | 0.0000032 | 0.23 | Skin - Sun Exposed (Lower leg) |
| ENSG00000138379.4 | MSTN | chr2_190369464_C_A_b38 | rs1108939 | 0.0000034 | -0.28 | Thyroid |
| ENSG00000138379.4 | MSTN | chr2_189963288_A_G_b38 | rs113532711 | 0.0000035 | -0.37 | Brain - Cerebellum |
| ENSG00000138379.4 | MSTN | chr2_190246661_G_A_b38 | rs61038868 | 0.0000037 | 0.23 | Skin - Sun Exposed (Lower leg) |
| ENSG00000138379.4 | MSTN | chr2_190288830_A_G_b38 | rs2664266 | 0.0000037 | -0.22 | Skin - Sun Exposed (Lower leg) |
| ENSG00000138379.4 | MSTN | chr2_190258603_A_G_b38 | rs291413 | 0.0000039 | 0.22 | Skin - Sun Exposed (Lower leg) |
| ENSG00000138379.4 | MSTN | chr2_190262846_C_T_b38 | rs2664271 | 0.0000039 | 0.22 | Skin - Sun Exposed (Lower leg) |
| ENSG00000138379.4 | MSTN | chr2_190329955_C_T_b38 | rs12615897 | 0.0000039 | -0.28 | Skin - Sun Exposed (Lower leg) |
| ENSG00000138379.4 | MSTN | chr2_190334828_C_T_b38 | rs62182001 | 0.0000039 | -0.28 | Skin - Sun Exposed (Lower leg) |
| ENSG00000138379.4 | MSTN | chr2_190237523_G_T_b38 | rs11684995 | 0.0000039 | 0.24 | Skin - Sun Exposed (Lower leg) |
| ENSG00000138379.4 | MSTN | chr2_190238070_A_C_b38 | rs2099127 | 0.0000039 | 0.24 | Skin - Sun Exposed (Lower leg) |
| ENSG00000138379.4 | MSTN | chr2_190323487_A_G_b38 | rs7567973 | 0.0000039 | 0.24 | Skin - Sun Exposed (Lower leg) |
| ENSG00000138379.4 | MSTN | chr2_190298534_G_A_b38 | rs62184436 | 0.0000041 | -0.28 | Skin - Sun Exposed (Lower leg) |
| ENSG00000138379.4 | MSTN | chr2_190268352_C_T_b38 | rs2664264 | 0.0000041 | -0.22 | Skin - Sun Exposed (Lower leg) |
| ENSG00000138379.4 | MSTN | chr2_190272435_C_T_b38 | rs2664242 | 0.0000041 | -0.22 | Skin - Sun Exposed (Lower leg) |
| ENSG00000138379.4 | MSTN | chr2_190302602_G_A_b38 | rs2136568 | 0.0000041 | -0.22 | Skin - Sun Exposed (Lower leg) |
| ENSG00000138379.4 | MSTN | chr2_190316371_G_A_b38 | rs2055825 | 0.0000041 | -0.28 | Skin - Sun Exposed (Lower leg) |
| ENSG00000138379.4 | MSTN | chr2_190276153_C_T_b38 | rs1583878 | 0.0000042 | -0.22 | Skin - Sun Exposed (Lower leg) |
| ENSG00000138379.4 | MSTN | chr2_190343615_C_T_b38 | rs2028431 | 0.0000042 | 0.24 | Skin - Sun Exposed (Lower leg) |
| ENSG00000138379.4 | MSTN | chr2_190275143_T_C_b38 | rs7599085 | 0.0000043 | 0.24 | Skin - Sun Exposed (Lower leg) |
| ENSG00000138379.4 | MSTN | chr2_190276772_T_C_b38 | rs72627905 | 0.0000043 | 0.24 | Skin - Sun Exposed (Lower leg) |
| ENSG00000138379.4 | MSTN | chr2_190288288_A_C_b38 | rs4853689 | 0.0000043 | 0.24 | Skin - Sun Exposed (Lower leg) |
| ENSG00000138379.4 | MSTN | chr2_190300243_G_A_b38 | rs6752703 | 0.0000043 | 0.24 | Skin - Sun Exposed (Lower leg) |
| ENSG00000138379.4 | MSTN | chr2_190305031_A_C_b38 | rs291435 | 0.0000044 | -0.22 | Skin - Sun Exposed (Lower leg) |
| ENSG00000138379.4 | MSTN | chr2_190306288_G_C_b38 | rs291437 | 0.0000044 | -0.22 | Skin - Sun Exposed (Lower leg) |
| ENSG00000138379.4 | MSTN | chr2_190306377_T_C_b38 | rs291438 | 0.0000044 | -0.22 | Skin - Sun Exposed (Lower leg) |
| ENSG00000138379.4 | MSTN | chr2_190306710_G_A_b38 | rs291439 | 0.0000044 | -0.22 | Skin - Sun Exposed (Lower leg) |
| ENSG00000138379.4 | MSTN | chr2_190006617_C_T_b38 | rs28651568 | 0.0000048 | -0.38 | Brain - Cerebellum |
| ENSG00000138379.4 | MSTN | chr2_190366777_G_A_b38 | rs4940 | 0.0000048 | 0.22 | Skin - Sun Exposed (Lower leg) |
| ENSG00000138379.4 | MSTN | chr2_190275561_C_T_b38 | rs2664244 | 0.0000048 | -0.22 | Skin - Sun Exposed (Lower leg) |
| ENSG00000138379.4 | MSTN | chr2_190276867_T_C_b38 | rs2136567 | 0.0000048 | -0.22 | Skin - Sun Exposed (Lower leg) |
| ENSG00000138379.4 | MSTN | chr2_190262711_T_C_b38 | rs291419 | 0.0000048 | 0.22 | Skin - Sun Exposed (Lower leg) |
| ENSG00000138379.4 | MSTN | chr2_190262847_A_T_b38 | rs2562799 | 0.0000048 | 0.22 | Skin - Sun Exposed (Lower leg) |
| ENSG00000138379.4 | MSTN | chr2_190262984_C_G_b38 | rs291420 | 0.0000048 | 0.22 | Skin - Sun Exposed (Lower leg) |
| ENSG00000138379.4 | MSTN | chr2_190263111_CA_C_b38 | rs34646136 | 0.0000048 | 0.22 | Skin - Sun Exposed (Lower leg) |
| ENSG00000138379.4 | MSTN | chr2_190264116_G_A_b38 | rs2582762 | 0.0000048 | 0.22 | Skin - Sun Exposed (Lower leg) |
| ENSG00000138379.4 | MSTN | chr2_190264249_C_T_b38 | rs2582763 | 0.0000048 | 0.22 | Skin - Sun Exposed (Lower leg) |
| ENSG00000138379.4 | MSTN | chr2_190265311_TATTTCACTGA_T_b38 | rs112829808 | 0.0000048 | 0.22 | Skin - Sun Exposed (Lower leg) |
| ENSG00000138379.4 | MSTN | chr2_190317563_A_C_b38 | rs291463 | 0.0000049 | -0.22 | Skin - Sun Exposed (Lower leg) |
| ENSG00000138379.4 | MSTN | chr2_190267842_A_AG_b38 | rs3838548 | 0.0000051 | -0.22 | Skin - Sun Exposed (Lower leg) |
| ENSG00000138379.4 | MSTN | chr2_190268789_A_G_b38 | rs291425 | 0.0000051 | -0.22 | Skin - Sun Exposed (Lower leg) |
| ENSG00000138379.4 | MSTN | chr2_190272774_C_G_b38 | rs2582771 | 0.0000051 | -0.22 | Skin - Sun Exposed (Lower leg) |
| ENSG00000138379.4 | MSTN | chr2_190274614_C_T_b38 | rs2582772 | 0.0000051 | -0.22 | Skin - Sun Exposed (Lower leg) |
| ENSG00000138379.4 | MSTN | chr2_190258124_A_G_b38 | rs77315392 | 0.0000051 | 0.24 | Skin - Sun Exposed (Lower leg) |
| ENSG00000138379.4 | MSTN | chr2_190265022_C_T_b38 | rs74661763 | 0.0000051 | 0.24 | Skin - Sun Exposed (Lower leg) |
| ENSG00000138379.4 | MSTN | chr2_190360961_C_T_b38 | rs2067411 | 0.0000052 | 0.23 | Skin - Sun Exposed (Lower leg) |
| ENSG00000138379.4 | MSTN | chr2_189940427_C_CG_b38 | rs578146022 | 0.0000052 | -0.39 | Brain - Cerebellum |
| ENSG00000138379.4 | MSTN | chr2_190256574_G_A_b38 | rs291410 | 0.0000054 | 0.22 | Skin - Sun Exposed (Lower leg) |
| ENSG00000138379.4 | MSTN | chr2_190226544_G_A_b38 | rs6434382 | 0.0000055 | 0.24 | Skin - Sun Exposed (Lower leg) |
| ENSG00000138379.4 | MSTN | chr2_190258242_C_T_b38 | rs291412 | 0.0000055 | 0.22 | Skin - Sun Exposed (Lower leg) |
| ENSG00000138379.4 | MSTN | chr2_190260142_G_A_b38 | rs291415 | 0.0000055 | 0.22 | Skin - Sun Exposed (Lower leg) |
| ENSG00000138379.4 | MSTN | chr2_190261520_C_A_b38 | rs291418 | 0.0000055 | 0.22 | Skin - Sun Exposed (Lower leg) |
| ENSG00000138379.4 | MSTN | chr2_190306932_T_G_b38 | rs291440 | 0.0000055 | -0.22 | Skin - Sun Exposed (Lower leg) |
| ENSG00000138379.4 | MSTN | chr2_190307538_T_C_b38 | rs291441 | 0.0000055 | -0.22 | Skin - Sun Exposed (Lower leg) |
| ENSG00000138379.4 | MSTN | chr2_190308874_G_A_b38 | rs291442 | 0.0000055 | -0.22 | Skin - Sun Exposed (Lower leg) |
| ENSG00000138379.4 | MSTN | chr2_190241970_C_T_b38 | rs7573689 | 0.0000055 | 0.22 | Skin - Sun Exposed (Lower leg) |
| ENSG00000138379.4 | MSTN | chr2_190286932_T_C_b38 | rs16832512 | 0.0000055 | 0.24 | Skin - Sun Exposed (Lower leg) |
| ENSG00000138379.4 | MSTN | chr2_190269317_C_T_b38 | rs291426 | 0.0000056 | -0.22 | Skin - Sun Exposed (Lower leg) |
| ENSG00000138379.4 | MSTN | chr2_190370015_G_A_b38 | rs4853695 | 0.0000057 | -0.27 | Thyroid |
| ENSG00000138379.4 | MSTN | chr2_190359482_G_A_b38 | rs2067398 | 0.0000057 | 0.23 | Skin - Sun Exposed (Lower leg) |
| ENSG00000138379.4 | MSTN | chr2_190362206_T_C_b38 | rs2067418 | 0.0000057 | 0.23 | Skin - Sun Exposed (Lower leg) |
| ENSG00000138379.4 | MSTN | chr2_190292176_T_G_b38 | rs12621421 | 0.0000058 | -0.28 | Skin - Sun Exposed (Lower leg) |
| ENSG00000138379.4 | MSTN | chr2_190273292_C_G_b38 | rs2664243 | 0.0000058 | -0.22 | Skin - Sun Exposed (Lower leg) |
| ENSG00000138379.4 | MSTN | chr2_190274992_T_C_b38 | rs2582773 | 0.0000058 | -0.22 | Skin - Sun Exposed (Lower leg) |
| ENSG00000138379.4 | MSTN | chr2_190278383_T_C_b38 | rs2941091 | 0.0000058 | -0.22 | Skin - Sun Exposed (Lower leg) |
| ENSG00000138379.4 | MSTN | chr2_190279283_T_C_b38 | rs2664246 | 0.0000058 | -0.22 | Skin - Sun Exposed (Lower leg) |
| ENSG00000138379.4 | MSTN | chr2_190280103_G_T_b38 | rs2664247 | 0.0000058 | -0.22 | Skin - Sun Exposed (Lower leg) |
| ENSG00000138379.4 | MSTN | chr2_190281457_C_A_b38 | rs1114863 | 0.0000058 | -0.22 | Skin - Sun Exposed (Lower leg) |
| ENSG00000138379.4 | MSTN | chr2_190285410_T_G_b38 | rs1866849 | 0.0000058 | -0.22 | Skin - Sun Exposed (Lower leg) |
| ENSG00000138379.4 | MSTN | chr2_190285617_T_C_b38 | rs1866850 | 0.0000058 | -0.22 | Skin - Sun Exposed (Lower leg) |
| ENSG00000138379.4 | MSTN | chr2_190293050_A_G_b38 | rs10931448 | 0.0000058 | -0.22 | Skin - Sun Exposed (Lower leg) |
| ENSG00000138379.4 | MSTN | chr2_190295165_C_T_b38 | rs291451 | 0.0000058 | -0.22 | Skin - Sun Exposed (Lower leg) |
| ENSG00000138379.4 | MSTN | chr2_190297119_C_T_b38 | rs2582743 | 0.0000058 | -0.22 | Skin - Sun Exposed (Lower leg) |
| ENSG00000138379.4 | MSTN | chr2_190297356_T_C_b38 | rs2582744 | 0.0000058 | -0.22 | Skin - Sun Exposed (Lower leg) |
| ENSG00000138379.4 | MSTN | chr2_190298554_G_T_b38 | rs2664258 | 0.0000058 | -0.22 | Skin - Sun Exposed (Lower leg) |
| ENSG00000138379.4 | MSTN | chr2_190300509_T_C_b38 | rs2582749 | 0.0000058 | -0.22 | Skin - Sun Exposed (Lower leg) |
| ENSG00000138379.4 | MSTN | chr2_190282296_T_C_b38 | rs2664249 | 0.0000060 | -0.22 | Skin - Sun Exposed (Lower leg) |
| ENSG00000138379.4 | MSTN | chr2_190312256_G_T_b38 | rs291446 | 0.0000060 | -0.22 | Skin - Sun Exposed (Lower leg) |
| ENSG00000138379.4 | MSTN | chr2_190237806_G_A_b38 | rs11685119 | 0.0000062 | 0.23 | Skin - Sun Exposed (Lower leg) |
| ENSG00000138379.4 | MSTN | chr2_190247515_G_A_b38 | rs11692448 | 0.0000062 | 0.23 | Skin - Sun Exposed (Lower leg) |
| ENSG00000138379.4 | MSTN | chr2_190248633_G_A_b38 | rs55742982 | 0.0000062 | 0.23 | Skin - Sun Exposed (Lower leg) |
| ENSG00000138379.4 | MSTN | chr2_190251629_A_G_b38 | rs3791792 | 0.0000062 | 0.23 | Skin - Sun Exposed (Lower leg) |
| ENSG00000138379.4 | MSTN | chr2_190262845_G_C_b38 | rs3791795 | 0.0000062 | 0.23 | Skin - Sun Exposed (Lower leg) |
| ENSG00000138379.4 | MSTN | chr2_190289810_C_T_b38 | rs2582742 | 0.0000062 | -0.22 | Skin - Sun Exposed (Lower leg) |
| ENSG00000138379.4 | MSTN | chr2_190290962_C_G_b38 | rs2966470 | 0.0000062 | -0.22 | Skin - Sun Exposed (Lower leg) |
| ENSG00000138379.4 | MSTN | chr2_190340576_C_T_b38 | rs11689196 | 0.0000062 | 0.24 | Skin - Sun Exposed (Lower leg) |
| ENSG00000138379.4 | MSTN | chr2_190040310_A_G_b38 | rs7595306 | 0.0000063 | -0.33 | Brain - Cerebellum |
| ENSG00000138379.4 | MSTN | chr2_190351383_T_A_b38 | rs2582753 | 0.0000063 | -0.22 | Skin - Sun Exposed (Lower leg) |
| ENSG00000138379.4 | MSTN | chr2_190232266_C_A_b38 | rs55684836 | 0.0000064 | 0.23 | Skin - Sun Exposed (Lower leg) |
| ENSG00000138379.4 | MSTN | chr2_190259372_T_G_b38 | rs2582759 | 0.0000065 | 0.22 | Skin - Sun Exposed (Lower leg) |
| ENSG00000138379.4 | MSTN | chr2_190235527_A_C_b38 | rs11888626 | 0.0000066 | 0.21 | Skin - Sun Exposed (Lower leg) |
| ENSG00000138379.4 | MSTN | chr2_190236361_T_TTAAA_b38 | rs112469972 | 0.0000066 | 0.21 | Skin - Sun Exposed (Lower leg) |
| ENSG00000138379.4 | MSTN | chr2_190243710_G_A_b38 | rs2099129 | 0.0000066 | 0.21 | Skin - Sun Exposed (Lower leg) |
| ENSG00000138379.4 | MSTN | chr2_190245144_C_A_b38 | rs2303827 | 0.0000066 | 0.21 | Skin - Sun Exposed (Lower leg) |
| ENSG00000138379.4 | MSTN | chr2_190247858_C_T_b38 | rs2664276 | 0.0000066 | 0.21 | Skin - Sun Exposed (Lower leg) |
| ENSG00000138379.4 | MSTN | chr2_190248071_G_C_b38 | rs2664275 | 0.0000066 | 0.21 | Skin - Sun Exposed (Lower leg) |
| ENSG00000138379.4 | MSTN | chr2_190252927_A_G_b38 | rs291404 | 0.0000066 | 0.21 | Skin - Sun Exposed (Lower leg) |
| ENSG00000138379.4 | MSTN | chr2_190253854_T_C_b38 | rs291405 | 0.0000066 | 0.21 | Skin - Sun Exposed (Lower leg) |
| ENSG00000138379.4 | MSTN | chr2_190254573_A_G_b38 | rs291407 | 0.0000066 | 0.21 | Skin - Sun Exposed (Lower leg) |
| ENSG00000138379.4 | MSTN | chr2_190362347_A_G_b38 | rs2067419 | 0.0000066 | 0.23 | Skin - Sun Exposed (Lower leg) |
| ENSG00000138379.4 | MSTN | chr2_190211930_G_A_b38 | rs72627903 | 0.0000067 | 0.23 | Skin - Sun Exposed (Lower leg) |
| ENSG00000138379.4 | MSTN | chr2_190215164_T_C_b38 | rs72627904 | 0.0000067 | 0.23 | Skin - Sun Exposed (Lower leg) |
| ENSG00000138379.4 | MSTN | chr2_190222778_C_T_b38 | rs6434381 | 0.0000067 | 0.23 | Skin - Sun Exposed (Lower leg) |
| ENSG00000138379.4 | MSTN | chr2_190227877_C_T_b38 | rs75520894 | 0.0000067 | 0.23 | Skin - Sun Exposed (Lower leg) |
| ENSG00000138379.4 | MSTN | chr2_190332009_T_A_b38 | rs58400804 | 0.0000067 | 0.23 | Skin - Sun Exposed (Lower leg) |
| ENSG00000138379.4 | MSTN | chr2_190336838_A_C_b38 | rs67791002 | 0.0000067 | 0.23 | Skin - Sun Exposed (Lower leg) |
| ENSG00000138379.4 | MSTN | chr2_190259556_G_A_b38 | rs176712 | 0.0000068 | 0.22 | Skin - Sun Exposed (Lower leg) |
| ENSG00000138379.4 | MSTN | chr2_190259705_T_C_b38 | rs291414 | 0.0000068 | 0.22 | Skin - Sun Exposed (Lower leg) |
| ENSG00000138379.4 | MSTN | chr2_190264529_C_T_b38 | rs2664269 | 0.0000068 | 0.22 | Skin - Sun Exposed (Lower leg) |
| ENSG00000138379.4 | MSTN | chr2_189991368_G_T_b38 | rs4449169 | 0.0000068 | -0.38 | Brain - Cerebellum |
| ENSG00000138379.4 | MSTN | chr2_189995644_T_C_b38 | rs72907373 | 0.0000068 | -0.38 | Brain - Cerebellum |
| ENSG00000138379.4 | MSTN | chr2_190314782_T_G_b38 | rs4853693 | 0.0000070 | 0.23 | Skin - Sun Exposed (Lower leg) |
| ENSG00000138379.4 | MSTN | chr2_190223888_G_A_b38 | rs60068799 | 0.0000072 | 0.23 | Skin - Sun Exposed (Lower leg) |
| ENSG00000138379.4 | MSTN | chr2_190233286_T_G_b38 | rs6723792 | 0.0000072 | 0.23 | Skin - Sun Exposed (Lower leg) |
| ENSG00000138379.4 | MSTN | chr2_190266958_C_T_b38 | rs291421 | 0.0000072 | -0.22 | Skin - Sun Exposed (Lower leg) |
| ENSG00000138379.4 | MSTN | chr2_190266965_T_C_b38 | rs291422 | 0.0000072 | -0.22 | Skin - Sun Exposed (Lower leg) |
| ENSG00000138379.4 | MSTN | chr2_190268120_G_A_b38 | rs291424 | 0.0000072 | -0.22 | Skin - Sun Exposed (Lower leg) |
| ENSG00000138379.4 | MSTN | chr2_190271657_A_T_b38 | rs2582769 | 0.0000072 | -0.22 | Skin - Sun Exposed (Lower leg) |
| ENSG00000138379.4 | MSTN | chr2_190272005_C_T_b38 | rs2664241 | 0.0000072 | -0.22 | Skin - Sun Exposed (Lower leg) |
| ENSG00000138379.4 | MSTN | chr2_190278392_G_T_b38 | rs2919675 | 0.0000072 | -0.22 | Skin - Sun Exposed (Lower leg) |
| ENSG00000138379.4 | MSTN | chr2_190280639_GA_G_b38 | rs34748012 | 0.0000072 | -0.22 | Skin - Sun Exposed (Lower leg) |
| ENSG00000138379.4 | MSTN | chr2_190281831_T_C_b38 | rs2582738 | 0.0000072 | -0.22 | Skin - Sun Exposed (Lower leg) |
| ENSG00000138379.4 | MSTN | chr2_190282905_A_G_b38 | rs2582740 | 0.0000072 | -0.22 | Skin - Sun Exposed (Lower leg) |
| ENSG00000138379.4 | MSTN | chr2_190284596_G_A_b38 | rs2664255 | 0.0000072 | -0.22 | Skin - Sun Exposed (Lower leg) |
| ENSG00000138379.4 | MSTN | chr2_190288864_T_C_b38 | rs2941092 | 0.0000072 | -0.22 | Skin - Sun Exposed (Lower leg) |
| ENSG00000138379.4 | MSTN | chr2_190294506_A_G_b38 | rs188339 | 0.0000072 | -0.22 | Skin - Sun Exposed (Lower leg) |
| ENSG00000138379.4 | MSTN | chr2_190295728_T_C_b38 | rs291455 | 0.0000072 | -0.22 | Skin - Sun Exposed (Lower leg) |
| ENSG00000138379.4 | MSTN | chr2_190296337_A_G_b38 | rs291456 | 0.0000072 | -0.22 | Skin - Sun Exposed (Lower leg) |
| ENSG00000138379.4 | MSTN | chr2_190296896_T_C_b38 | rs1058180 | 0.0000072 | -0.22 | Skin - Sun Exposed (Lower leg) |
| ENSG00000138379.4 | MSTN | chr2_190297563_T_G_b38 | rs2664256 | 0.0000072 | -0.22 | Skin - Sun Exposed (Lower leg) |
| ENSG00000138379.4 | MSTN | chr2_190298250_C_T_b38 | rs113630156 | 0.0000072 | -0.22 | Skin - Sun Exposed (Lower leg) |
| ENSG00000138379.4 | MSTN | chr2_190299097_A_G_b38 | rs2664261 | 0.0000072 | -0.22 | Skin - Sun Exposed (Lower leg) |
| ENSG00000138379.4 | MSTN | chr2_190299433_A_G_b38 | rs2582747 | 0.0000072 | -0.22 | Skin - Sun Exposed (Lower leg) |
| ENSG00000138379.4 | MSTN | chr2_190302915_A_G_b38 | rs2136570 | 0.0000072 | -0.22 | Skin - Sun Exposed (Lower leg) |
| ENSG00000138379.4 | MSTN | chr2_190302952_A_G_b38 | rs2136571 | 0.0000072 | -0.22 | Skin - Sun Exposed (Lower leg) |
| ENSG00000138379.4 | MSTN | chr2_190201957_C_T_b38 | rs4853685 | 0.0000072 | 0.21 | Skin - Sun Exposed (Lower leg) |
| ENSG00000138379.4 | MSTN | chr2_190200510_TTTTG_T_b38 | rs149132415 | 0.0000072 | 0.22 | Skin - Sun Exposed (Lower leg) |
| ENSG00000138379.4 | MSTN | chr2_190294130_A_C_b38 | rs291450 | 0.0000073 | -0.22 | Skin - Sun Exposed (Lower leg) |
| ENSG00000138379.4 | MSTN | chr2_190300703_C_T_b38 | rs60469543 | 0.0000075 | 0.23 | Skin - Sun Exposed (Lower leg) |
| ENSG00000138379.4 | MSTN | chr2_189980420_C_A_b38 | rs113722942 | 0.0000076 | -0.38 | Brain - Cerebellum |
| ENSG00000138379.4 | MSTN | chr2_190321471_CAT_C_b38 | rs79576023 | 0.0000076 | 0.23 | Skin - Sun Exposed (Lower leg) |
| ENSG00000138379.4 | MSTN | chr2_190325327_C_G_b38 | rs2354871 | 0.0000076 | 0.23 | Skin - Sun Exposed (Lower leg) |
| ENSG00000138379.4 | MSTN | chr2_190328370_AC_A_b38 | rs79455790 | 0.0000076 | 0.23 | Skin - Sun Exposed (Lower leg) |
| ENSG00000138379.4 | MSTN | chr2_190300939_C_T_b38 | rs2251867 | 0.0000078 | -0.22 | Skin - Sun Exposed (Lower leg) |
| ENSG00000138379.4 | MSTN | chr2_190330756_T_C_b38 | rs57199157 | 0.0000078 | 0.23 | Skin - Sun Exposed (Lower leg) |
| ENSG00000138379.4 | MSTN | chr2_189947246_T_C_b38 | rs7564968 | 0.0000079 | -0.37 | Brain - Cerebellum |
| ENSG00000138379.4 | MSTN | chr2_190235997_T_C_b38 | rs16832456 | 0.0000083 | 0.21 | Skin - Sun Exposed (Lower leg) |
| ENSG00000138379.4 | MSTN | chr2_190237141_C_G_b38 | rs10188044 | 0.0000083 | 0.21 | Skin - Sun Exposed (Lower leg) |
| ENSG00000138379.4 | MSTN | chr2_190237202_T_C_b38 | rs10200496 | 0.0000083 | 0.21 | Skin - Sun Exposed (Lower leg) |
| ENSG00000138379.4 | MSTN | chr2_190238091_T_C_b38 | rs2099128 | 0.0000083 | 0.21 | Skin - Sun Exposed (Lower leg) |
| ENSG00000138379.4 | MSTN | chr2_190244474_A_G_b38 | rs3791791 | 0.0000083 | 0.21 | Skin - Sun Exposed (Lower leg) |
| ENSG00000138379.4 | MSTN | chr2_190244807_C_T_b38 | rs11883568 | 0.0000083 | 0.21 | Skin - Sun Exposed (Lower leg) |
| ENSG00000138379.4 | MSTN | chr2_190252914_C_T_b38 | rs291403 | 0.0000083 | 0.21 | Skin - Sun Exposed (Lower leg) |
| ENSG00000138379.4 | MSTN | chr2_190202244_C_T_b38 | rs3749016 | 0.0000084 | 0.21 | Skin - Sun Exposed (Lower leg) |
| ENSG00000138379.4 | MSTN | chr2_190204963_T_A_b38 | rs11542 | 0.0000084 | 0.21 | Skin - Sun Exposed (Lower leg) |
| ENSG00000138379.4 | MSTN | chr2_190317355_C_T_b38 | rs3791806 | 0.0000084 | 0.23 | Skin - Sun Exposed (Lower leg) |
| ENSG00000138379.4 | MSTN | chr2_190000767_A_G_b38 | rs6728421 | 0.0000086 | -0.36 | Brain - Cerebellum |
| ENSG00000138379.4 | MSTN | chr2_190216961_G_A_b38 | rs56742312 | 0.0000086 | 0.23 | Skin - Sun Exposed (Lower leg) |
| ENSG00000138379.4 | MSTN | chr2_190231246_C_T_b38 | rs11683636 | 0.0000086 | 0.23 | Skin - Sun Exposed (Lower leg) |
| ENSG00000138379.4 | MSTN | chr2_189984396_G_C_b38 | rs11682589 | 0.0000087 | -0.37 | Brain - Cerebellum |
| ENSG00000138379.4 | MSTN | chr2_190288194_T_C_b38 | rs2256301 | 0.0000089 | -0.21 | Skin - Sun Exposed (Lower leg) |
| ENSG00000138379.4 | MSTN | chr2_190197305_C_T_b38 | rs11894667 | 0.0000089 | 0.21 | Skin - Sun Exposed (Lower leg) |
| ENSG00000138379.4 | MSTN | chr2_190234769_C_G_b38 | rs10196351 | 0.0000092 | 0.21 | Skin - Sun Exposed (Lower leg) |
| ENSG00000138379.4 | MSTN | chr2_190242262_C_T_b38 | rs7574028 | 0.0000092 | 0.21 | Skin - Sun Exposed (Lower leg) |
| ENSG00000138379.4 | MSTN | chr2_190249093_C_T_b38 | rs2582757 | 0.0000092 | 0.21 | Skin - Sun Exposed (Lower leg) |
| ENSG00000138379.4 | MSTN | chr2_190251105_C_T_b38 | rs713426 | 0.0000092 | 0.21 | Skin - Sun Exposed (Lower leg) |
| ENSG00000138379.4 | MSTN | chr2_190252480_G_A_b38 | rs291402 | 0.0000092 | 0.21 | Skin - Sun Exposed (Lower leg) |
| ENSG00000138379.4 | MSTN | chr2_190308058_C_G_b38 | rs3791805 | 0.0000095 | 0.23 | Skin - Sun Exposed (Lower leg) |
| ENSG00000138379.4 | MSTN | chr2_190202507_T_C_b38 | rs55815425 | 0.000010 | 0.23 | Skin - Sun Exposed (Lower leg) |
| ENSG00000138379.4 | MSTN | chr2_190258114_T_A_b38 | rs291411 | 0.000010 | 0.21 | Skin - Sun Exposed (Lower leg) |
| ENSG00000138379.4 | MSTN | chr2_190216149_G_C_b38 | rs7569366 | 0.000010 | 0.22 | Skin - Sun Exposed (Lower leg) |
| ENSG00000138379.4 | MSTN | chr2_190222032_C_T_b38 | rs66867328 | 0.000010 | 0.22 | Skin - Sun Exposed (Lower leg) |
| ENSG00000138379.4 | MSTN | chr2_190228379_C_T_b38 | rs35214387 | 0.000010 | 0.22 | Skin - Sun Exposed (Lower leg) |
| ENSG00000138379.4 | MSTN | chr2_190304198_C_T_b38 | rs291434 | 0.000011 | -0.21 | Skin - Sun Exposed (Lower leg) |
| ENSG00000138379.4 | MSTN | chr2_189440722_G_C_b38 | rs10184474 | 0.000011 | -0.35 | Artery - Tibial |
| ENSG00000138379.4 | MSTN | chr2_190511849_C_T_b38 | rs75579212 | 0.000011 | 0.42 | Spleen |
| ENSG00000138379.4 | MSTN | chr2_190217468_A_G_b38 | rs6751238 | 0.000011 | 0.21 | Skin - Sun Exposed (Lower leg) |
| ENSG00000138379.4 | MSTN | chr2_190217755_A_T_b38 | rs6751588 | 0.000011 | 0.21 | Skin - Sun Exposed (Lower leg) |
| ENSG00000138379.4 | MSTN | chr2_190220095_T_C_b38 | rs3791789 | 0.000011 | 0.21 | Skin - Sun Exposed (Lower leg) |
| ENSG00000138379.4 | MSTN | chr2_190220384_A_G_b38 | rs3828269 | 0.000011 | 0.21 | Skin - Sun Exposed (Lower leg) |
| ENSG00000138379.4 | MSTN | chr2_190225111_C_T_b38 | rs7371399 | 0.000011 | 0.21 | Skin - Sun Exposed (Lower leg) |
| ENSG00000138379.4 | MSTN | chr2_190226285_A_AG_b38 | rs35318181 | 0.000011 | 0.21 | Skin - Sun Exposed (Lower leg) |
| ENSG00000138379.4 | MSTN | chr2_190228513_G_C_b38 | rs13011138 | 0.000011 | 0.21 | Skin - Sun Exposed (Lower leg) |
| ENSG00000138379.4 | MSTN | chr2_190228928_T_C_b38 | rs1366672 | 0.000011 | 0.21 | Skin - Sun Exposed (Lower leg) |
| ENSG00000138379.4 | MSTN | chr2_190229219_C_T_b38 | rs6749512 | 0.000011 | 0.21 | Skin - Sun Exposed (Lower leg) |
| ENSG00000138379.4 | MSTN | chr2_190231200_C_T_b38 | rs13405019 | 0.000011 | 0.21 | Skin - Sun Exposed (Lower leg) |
| ENSG00000138379.4 | MSTN | chr2_190231331_G_C_b38 | rs11683763 | 0.000011 | 0.21 | Skin - Sun Exposed (Lower leg) |
| ENSG00000138379.4 | MSTN | chr2_190232442_T_C_b38 | rs6720060 | 0.000011 | 0.21 | Skin - Sun Exposed (Lower leg) |
| ENSG00000138379.4 | MSTN | chr2_190200027_C_T_b38 | rs6753459 | 0.000011 | 0.21 | Skin - Sun Exposed (Lower leg) |
| ENSG00000138379.4 | MSTN | chr2_190243365_T_G_b38 | rs10173593 | 0.000012 | 0.21 | Skin - Sun Exposed (Lower leg) |
| ENSG00000138379.4 | MSTN | chr2_190247229_G_A_b38 | rs2664277 | 0.000012 | 0.21 | Skin - Sun Exposed (Lower leg) |
| ENSG00000138379.4 | MSTN | chr2_190248283_T_G_b38 | rs2562796 | 0.000012 | 0.21 | Skin - Sun Exposed (Lower leg) |
| ENSG00000138379.4 | MSTN | chr2_190248929_C_A_b38 | rs2664274 | 0.000012 | 0.21 | Skin - Sun Exposed (Lower leg) |
| ENSG00000138379.4 | MSTN | chr2_190249090_T_C_b38 | rs2664273 | 0.000012 | 0.21 | Skin - Sun Exposed (Lower leg) |
| ENSG00000138379.4 | MSTN | chr2_190249336_T_C_b38 | rs2664272 | 0.000012 | 0.21 | Skin - Sun Exposed (Lower leg) |
| ENSG00000138379.4 | MSTN | chr2_190249503_T_C_b38 | rs2244076 | 0.000012 | 0.21 | Skin - Sun Exposed (Lower leg) |
| ENSG00000138379.4 | MSTN | chr2_190249509_G_A_b38 | rs2244075 | 0.000012 | 0.21 | Skin - Sun Exposed (Lower leg) |
| ENSG00000138379.4 | MSTN | chr2_190251082_C_A_b38 | rs713425 | 0.000012 | 0.21 | Skin - Sun Exposed (Lower leg) |
| ENSG00000138379.4 | MSTN | chr2_190199330_G_A_b38 | rs13415995 | 0.000013 | 0.21 | Skin - Sun Exposed (Lower leg) |
| ENSG00000138379.4 | MSTN | chr2_190210721_G_A_b38 | rs72625302 | 0.000013 | 0.23 | Skin - Sun Exposed (Lower leg) |
| ENSG00000138379.4 | MSTN | chr2_190278401_G_A_b38 | rs2919676 | 0.000014 | -0.22 | Skin - Sun Exposed (Lower leg) |
| ENSG00000138379.4 | MSTN | chr2_190223854_T_C_b38 | rs12693564 | 0.000014 | 0.21 | Skin - Sun Exposed (Lower leg) |
| ENSG00000138379.4 | MSTN | chr2_190353645_A_G_b38 | rs3791813 | 0.000014 | 0.22 | Skin - Sun Exposed (Lower leg) |
| ENSG00000138379.4 | MSTN | chr2_190211137_T_C_b38 | rs10183133 | 0.000014 | 0.21 | Skin - Sun Exposed (Lower leg) |
| ENSG00000138379.4 | MSTN | chr2_190213309_A_C_b38 | rs3815961 | 0.000014 | 0.21 | Skin - Sun Exposed (Lower leg) |
| ENSG00000138379.4 | MSTN | chr2_190221615_T_C_b38 | rs34760043 | 0.000014 | 0.21 | Skin - Sun Exposed (Lower leg) |
| ENSG00000138379.4 | MSTN | chr2_190224632_A_G_b38 | rs56853355 | 0.000014 | 0.21 | Skin - Sun Exposed (Lower leg) |
| ENSG00000138379.4 | MSTN | chr2_190226549_T_C_b38 | rs6434383 | 0.000014 | 0.21 | Skin - Sun Exposed (Lower leg) |
| ENSG00000138379.4 | MSTN | chr2_190227308_A_G_b38 | rs78187364 | 0.000014 | 0.21 | Skin - Sun Exposed (Lower leg) |
| ENSG00000138379.4 | MSTN | chr2_190228047_C_T_b38 | rs4853688 | 0.000014 | 0.21 | Skin - Sun Exposed (Lower leg) |
| ENSG00000138379.4 | MSTN | chr2_190228539_A_C_b38 | rs13010589 | 0.000014 | 0.21 | Skin - Sun Exposed (Lower leg) |
| ENSG00000138379.4 | MSTN | chr2_190231348_T_C_b38 | rs11689666 | 0.000014 | 0.21 | Skin - Sun Exposed (Lower leg) |
| ENSG00000138379.4 | MSTN | chr2_190231678_A_G_b38 | rs11695166 | 0.000014 | 0.21 | Skin - Sun Exposed (Lower leg) |
| ENSG00000138379.4 | MSTN | chr2_190207562_T_C_b38 | rs72625301 | 0.000014 | 0.21 | Skin - Sun Exposed (Lower leg) |
| ENSG00000138379.4 | MSTN | chr2_190208473_A_G_b38 | rs3762548 | 0.000014 | 0.21 | Skin - Sun Exposed (Lower leg) |
| ENSG00000138379.4 | MSTN | chr2_190209198_C_G_b38 | rs7562807 | 0.000014 | 0.21 | Skin - Sun Exposed (Lower leg) |
| ENSG00000138379.4 | MSTN | chr2_190213437_G_A_b38 | rs3791788 | 0.000014 | 0.21 | Skin - Sun Exposed (Lower leg) |
| ENSG00000138379.4 | MSTN | chr2_190196907_G_T_b38 | rs10188870 | 0.000014 | 0.21 | Skin - Sun Exposed (Lower leg) |
| ENSG00000138379.4 | MSTN | chr2_189988448_G_T_b38 | rs72907363 | 0.000015 | -0.44 | Brain - Cerebellum |
| ENSG00000138379.4 | MSTN | chr2_190490237_C_A_b38 | rs62181034 | 0.000015 | 0.41 | Spleen |
| ENSG00000138379.4 | MSTN | chr2_190492123_A_G_b38 | rs75488522 | 0.000015 | 0.41 | Spleen |
| ENSG00000138379.4 | MSTN | chr2_190501981_A_G_b38 | rs1128723 | 0.000015 | 0.41 | Spleen |
| ENSG00000138379.4 | MSTN | chr2_190518622_A_G_b38 | rs12053190 | 0.000015 | 0.41 | Spleen |
| ENSG00000138379.4 | MSTN | chr2_190522353_T_G_b38 | rs10432434 | 0.000015 | 0.41 | Spleen |
| ENSG00000138379.4 | MSTN | chr2_190530301_C_T_b38 | rs4258824 | 0.000015 | 0.41 | Spleen |
| ENSG00000138379.4 | MSTN | chr2_190533627_A_C_b38 | rs4439990 | 0.000015 | 0.41 | Spleen |
| ENSG00000138379.4 | MSTN | chr2_190203040_T_TTC_b38 | rs143044502 | 0.000016 | 0.21 | Skin - Sun Exposed (Lower leg) |
| ENSG00000138379.4 | MSTN | chr2_190203739_A_G_b38 | rs6737846 | 0.000016 | 0.21 | Skin - Sun Exposed (Lower leg) |
| ENSG00000138379.4 | MSTN | chr2_190210010_G_T_b38 | rs2353890 | 0.000016 | 0.21 | Skin - Sun Exposed (Lower leg) |
| ENSG00000138379.4 | MSTN | chr2_190212538_C_T_b38 | rs7574495 | 0.000016 | 0.21 | Skin - Sun Exposed (Lower leg) |
| ENSG00000138379.4 | MSTN | chr2_190215024_A_G_b38 | rs1972288 | 0.000016 | 0.21 | Skin - Sun Exposed (Lower leg) |
| ENSG00000138379.4 | MSTN | chr2_190221815_A_T_b38 | rs66738092 | 0.000016 | 0.21 | Skin - Sun Exposed (Lower leg) |
| ENSG00000138379.4 | MSTN | chr2_190222457_A_G_b38 | rs7565423 | 0.000016 | 0.21 | Skin - Sun Exposed (Lower leg) |
| ENSG00000138379.4 | MSTN | chr2_190220259_T_A_b38 | rs3791790 | 0.000017 | 0.20 | Skin - Sun Exposed (Lower leg) |
| ENSG00000138379.4 | MSTN | chr2_190482672_CT_C_b38 | rs59305081 | 0.000018 | 0.41 | Spleen |
| ENSG00000138379.4 | MSTN | chr2_190198345_G_C_b38 | rs16832420 | 0.000018 | 0.22 | Skin - Sun Exposed (Lower leg) |
| ENSG00000138379.4 | MSTN | chr2_190348938_A_G_b38 | rs1882892 | 0.000018 | -0.20 | Skin - Sun Exposed (Lower leg) |
| ENSG00000138379.4 | MSTN | chr2_190513444_C_T_b38 | rs12052514 | 0.000018 | 0.41 | Spleen |
| ENSG00000138379.4 | MSTN | chr2_190196682_A_G_b38 | rs10209516 | 0.000018 | 0.21 | Skin - Sun Exposed (Lower leg) |
| ENSG00000138379.4 | MSTN | chr2_190562973_GGT_G_b38 | rs200611893 | 0.000018 | 0.34 | Stomach |
| ENSG00000138379.4 | MSTN | chr2_190562978_T_A_b38 | rs543299239 | 0.000018 | 0.34 | Stomach |
| ENSG00000138379.4 | MSTN | chr2_190562980_C_A_b38 | rs80094409 | 0.000018 | 0.34 | Stomach |
| ENSG00000138379.4 | MSTN | chr2_190562981_T_TTTATAG_b38 | rs377730611 | 0.000018 | 0.34 | Stomach |
| ENSG00000138379.4 | MSTN | chr2_190569454_T_C_b38 | rs62182865 | 0.000018 | 0.34 | Stomach |
| ENSG00000138379.4 | MSTN | chr2_189939024_G_A_b38 | rs72905399 | 0.000018 | -0.36 | Brain - Cerebellum |
| ENSG00000138379.4 | MSTN | chr2_189939772_G_T_b38 | rs72905401 | 0.000018 | -0.36 | Brain - Cerebellum |
| ENSG00000138379.4 | MSTN | chr2_189961697_G_T_b38 | rs11685436 | 0.000018 | -0.36 | Brain - Cerebellum |
| ENSG00000138379.4 | MSTN | chr2_190006510_C_T_b38 | rs28377311 | 0.000018 | -0.35 | Brain - Cerebellum |
| ENSG00000138379.4 | MSTN | chr2_190015735_A_C_b38 | rs56014369 | 0.000018 | -0.35 | Brain - Cerebellum |
| ENSG00000138379.4 | MSTN | chr2_190540554_T_A_b38 | rs10931461 | 0.000019 | -0.41 | Spleen |
| ENSG00000138379.4 | MSTN | chr2_190566015_A_G_b38 | rs4371384 | 0.000019 | -0.41 | Spleen |
| ENSG00000138379.4 | MSTN | chr2_190018634_T_C_b38 | rs10931440 | 0.000019 | 0.30 | Brain - Cerebellum |
| ENSG00000138379.4 | MSTN | chr2_189440723_A_G_b38 | rs10207812 | 0.000019 | -0.35 | Artery - Tibial |
| ENSG00000138379.4 | MSTN | chr2_190210294_A_G_b38 | rs10191068 | 0.000020 | 0.20 | Skin - Sun Exposed (Lower leg) |
| ENSG00000138379.4 | MSTN | chr2_190216151_T_C_b38 | rs7583250 | 0.000020 | 0.20 | Skin - Sun Exposed (Lower leg) |
| ENSG00000138379.4 | MSTN | chr2_190216292_C_T_b38 | rs7569296 | 0.000020 | 0.20 | Skin - Sun Exposed (Lower leg) |
| ENSG00000138379.4 | MSTN | chr2_190220472_G_A_b38 | rs4853687 | 0.000020 | 0.20 | Skin - Sun Exposed (Lower leg) |
| ENSG00000138379.4 | MSTN | chr2_190221294_A_C_b38 | rs10209984 | 0.000020 | 0.20 | Skin - Sun Exposed (Lower leg) |
| ENSG00000138379.4 | MSTN | chr2_190230780_C_T_b38 | rs7602400 | 0.000020 | 0.20 | Skin - Sun Exposed (Lower leg) |
| ENSG00000138379.4 | MSTN | chr2_190231012_T_A_b38 | rs7566380 | 0.000020 | 0.20 | Skin - Sun Exposed (Lower leg) |
| ENSG00000138379.4 | MSTN | chr2_190487209_C_G_b38 | rs11885992 | 0.000020 | 0.41 | Spleen |
| ENSG00000138379.4 | MSTN | chr2_190015208_G_A_b38 | rs59270595 | 0.000020 | -0.36 | Brain - Cerebellum |
| ENSG00000138379.4 | MSTN | chr2_190218339_T_C_b38 | rs7590942 | 0.000020 | 0.20 | Skin - Sun Exposed (Lower leg) |
| ENSG00000138379.4 | MSTN | chr2_189970747_G_A_b38 | rs6749643 | 0.000021 | -0.34 | Brain - Cerebellum |
| ENSG00000138379.4 | MSTN | chr2_189972414_A_G_b38 | rs7578546 | 0.000021 | -0.34 | Brain - Cerebellum |
| ENSG00000138379.4 | MSTN | chr2_189982022_A_G_b38 | rs72907349 | 0.000021 | -0.34 | Brain - Cerebellum |
| ENSG00000138379.4 | MSTN | chr2_190197469_C_A_b38 | rs11894766 | 0.000021 | 0.20 | Skin - Sun Exposed (Lower leg) |
| ENSG00000138379.4 | MSTN | chr2_190059437_T_C_b38 | rs3791783 | 0.000021 | -0.31 | Brain - Cerebellum |
| ENSG00000138379.4 | MSTN | chr2_190062133_GT_G_b38 | rs11333758 | 0.000021 | -0.31 | Brain - Cerebellum |
| ENSG00000138379.4 | MSTN | chr2_190198225_A_G_b38 | rs4853684 | 0.000021 | 0.22 | Skin - Sun Exposed (Lower leg) |
| ENSG00000138379.4 | MSTN | chr2_189401053_C_CTTTTT_b38 | rs56397395 | 0.000022 | -0.35 | Artery - Tibial |
| ENSG00000138379.4 | MSTN | chr2_189404407_T_C_b38 | rs10210634 | 0.000022 | -0.35 | Artery - Tibial |
| ENSG00000138379.4 | MSTN | chr2_189420502_A_T_b38 | rs1356976 | 0.000022 | -0.35 | Artery - Tibial |
| ENSG00000138379.4 | MSTN | chr2_189432613_A_T_b38 | rs2882805 | 0.000022 | -0.35 | Artery - Tibial |
| ENSG00000138379.4 | MSTN | chr2_189434705_C_T_b38 | rs6434342 | 0.000022 | -0.35 | Artery - Tibial |
| ENSG00000138379.4 | MSTN | chr2_190579100_TAGAA_T_b38 | rs148640504 | 0.000022 | 0.34 | Stomach |
| ENSG00000138379.4 | MSTN | chr2_190590898_A_C_b38 | rs12477369 | 0.000022 | 0.34 | Stomach |
| ENSG00000138379.4 | MSTN | chr2_190604036_T_C_b38 | rs16832765 | 0.000022 | 0.34 | Stomach |
| ENSG00000138379.4 | MSTN | chr2_190267633_G_A_b38 | rs291423 | 0.000022 | -0.20 | Skin - Sun Exposed (Lower leg) |
| ENSG00000138379.4 | MSTN | chr2_190466578_G_C_b38 | rs60915953 | 0.000023 | 0.33 | Stomach |
| ENSG00000138379.4 | MSTN | chr2_190474407_A_G_b38 | rs62181029 | 0.000023 | 0.33 | Stomach |
| ENSG00000138379.4 | MSTN | chr2_190475169_C_G_b38 | rs62181030 | 0.000023 | 0.33 | Stomach |
| ENSG00000138379.4 | MSTN | chr2_190483619_G_C_b38 | rs113513615 | 0.000023 | 0.33 | Stomach |
| ENSG00000138379.4 | MSTN | chr2_190523132_A_G_b38 | rs62181052 | 0.000023 | 0.33 | Stomach |
| ENSG00000138379.4 | MSTN | chr2_190533819_G_A_b38 | rs12472081 | 0.000023 | 0.33 | Stomach |
| ENSG00000138379.4 | MSTN | chr2_189463207_T_C_b38 | rs4666775 | 0.000023 | -0.34 | Artery - Tibial |
| ENSG00000138379.4 | MSTN | chr2_189466035_G_A_b38 | rs1520866 | 0.000023 | -0.34 | Artery - Tibial |
| ENSG00000138379.4 | MSTN | chr2_189487826_G_T_b38 | rs12622720 | 0.000024 | -0.35 | Artery - Tibial |
| ENSG00000138379.4 | MSTN | chr2_189476347_C_T_b38 | rs8179732 | 0.000025 | -0.34 | Artery - Tibial |
| ENSG00000138379.4 | MSTN | chr2_189476357_A_C_b38 | rs8179541 | 0.000025 | -0.34 | Artery - Tibial |
| ENSG00000138379.4 | MSTN | chr2_190003441_G_T_b38 | rs11695980 | 0.000025 | -0.35 | Brain - Cerebellum |
| ENSG00000138379.4 | MSTN | chr2_190581965_C_A_b38 | rs7569128 | 0.000025 | -0.40 | Spleen |
| ENSG00000138379.4 | MSTN | chr2_190597407_C_T_b38 | rs2192010 | 0.000025 | 0.40 | Spleen |
| ENSG00000138379.4 | MSTN | chr2_190604260_G_C_b38 | rs61117285 | 0.000025 | 0.40 | Spleen |
| ENSG00000138379.4 | MSTN | chr2_190470903_G_GTA_b38 | rs148619709 | 0.000026 | 0.36 | Stomach |
| ENSG00000138379.4 | MSTN | chr2_189396558_G_C_b38 | rs11677167 | 0.000026 | -0.34 | Artery - Tibial |
| ENSG00000138379.4 | MSTN | chr2_190138823_C_T_b38 | rs1366675 | 0.000027 | 0.49 | Heart - Atrial Appendage |
| ENSG00000138379.4 | MSTN | chr2_190138867_C_T_b38 | rs1366674 | 0.000027 | 0.49 | Heart - Atrial Appendage |
| ENSG00000138379.4 | MSTN | chr2_190138869_T_C_b38 | rs1366673 | 0.000027 | 0.49 | Heart - Atrial Appendage |
| ENSG00000138379.4 | MSTN | chr2_190139025_AG_A_b38 | rs11359759 | 0.000027 | 0.49 | Heart - Atrial Appendage |
| ENSG00000138379.4 | MSTN | chr2_190139070_A_C_b38 | rs2562803 | 0.000027 | 0.49 | Heart - Atrial Appendage |
| ENSG00000138379.4 | MSTN | chr2_190139688_T_C_b38 | rs1072068 | 0.000027 | 0.49 | Heart - Atrial Appendage |
| ENSG00000138379.4 | MSTN | chr2_190139788_T_A_b38 | rs1806355 | 0.000027 | 0.49 | Heart - Atrial Appendage |
| ENSG00000138379.4 | MSTN | chr2_190140126_G_T_b38 | rs2562807 | 0.000027 | 0.49 | Heart - Atrial Appendage |
| ENSG00000138379.4 | MSTN | chr2_190140205_T_G_b38 | rs2562808 | 0.000027 | 0.49 | Heart - Atrial Appendage |
| ENSG00000138379.4 | MSTN | chr2_190140280_C_A_b38 | rs2562809 | 0.000027 | 0.49 | Heart - Atrial Appendage |
| ENSG00000138379.4 | MSTN | chr2_190140539_G_A_b38 | rs2562810 | 0.000027 | 0.49 | Heart - Atrial Appendage |
| ENSG00000138379.4 | MSTN | chr2_190140741_T_C_b38 | rs12693562 | 0.000027 | 0.49 | Heart - Atrial Appendage |
| ENSG00000138379.4 | MSTN | chr2_190140778_A_G_b38 | rs12693563 | 0.000027 | 0.49 | Heart - Atrial Appendage |
| ENSG00000138379.4 | MSTN | chr2_190141664_T_A_b38 | rs2562811 | 0.000027 | 0.49 | Heart - Atrial Appendage |
| ENSG00000138379.4 | MSTN | chr2_190141775_C_T_b38 | rs2562812 | 0.000027 | 0.49 | Heart - Atrial Appendage |
| ENSG00000138379.4 | MSTN | chr2_190142034_A_G_b38 | rs2562813 | 0.000027 | 0.49 | Heart - Atrial Appendage |
| ENSG00000138379.4 | MSTN | chr2_190142095_T_C_b38 | rs2690727 | 0.000027 | 0.49 | Heart - Atrial Appendage |
| ENSG00000138379.4 | MSTN | chr2_190142705_C_T_b38 | rs2562814 | 0.000027 | 0.49 | Heart - Atrial Appendage |
| ENSG00000138379.4 | MSTN | chr2_190142725_A_G_b38 | rs2562815 | 0.000027 | 0.49 | Heart - Atrial Appendage |
| ENSG00000138379.4 | MSTN | chr2_190143241_T_C_b38 | rs2690729 | 0.000027 | 0.49 | Heart - Atrial Appendage |
| ENSG00000138379.4 | MSTN | chr2_190143355_G_A_b38 | rs2562816 | 0.000027 | 0.49 | Heart - Atrial Appendage |
| ENSG00000138379.4 | MSTN | chr2_190143399_T_C_b38 | rs2562817 | 0.000027 | 0.49 | Heart - Atrial Appendage |
| ENSG00000138379.4 | MSTN | chr2_190143575_A_G_b38 | rs2562818 | 0.000027 | 0.49 | Heart - Atrial Appendage |
| ENSG00000138379.4 | MSTN | chr2_190544890_C_G_b38 | rs35818159 | 0.000027 | 0.33 | Stomach |
| ENSG00000138379.4 | MSTN | chr2_189214177_A_G_b38 | rs13411992 | 0.000028 | 0.33 | Artery - Tibial |
| ENSG00000138379.4 | MSTN | chr2_189215637_T_A_b38 | rs10184895 | 0.000028 | 0.33 | Artery - Tibial |
| ENSG00000138379.4 | MSTN | chr2_189215769_C_T_b38 | rs10172607 | 0.000028 | 0.33 | Artery - Tibial |
| ENSG00000138379.4 | MSTN | chr2_189216917_T_C_b38 | rs10173224 | 0.000028 | 0.33 | Artery - Tibial |
| ENSG00000138379.4 | MSTN | chr2_190604261_G_A_b38 | rs58568707 | 0.000028 | 0.40 | Spleen |
| ENSG00000138379.4 | MSTN | chr2_190605526_C_G_b38 | rs112979515 | 0.000028 | 0.40 | Spleen |
| ENSG00000138379.4 | MSTN | chr2_190606669_C_T_b38 | rs62182897 | 0.000028 | 0.40 | Spleen |
| ENSG00000138379.4 | MSTN | chr2_190371653_A_G_b38 | rs2067454 | 0.000029 | 0.20 | Skin - Sun Exposed (Lower leg) |
| ENSG00000138379.4 | MSTN | chr2_189191856_A_T_b38 | rs12474942 | 0.000029 | 0.33 | Artery - Tibial |
| ENSG00000138379.4 | MSTN | chr2_189197180_T_C_b38 | rs13408928 | 0.000029 | 0.33 | Artery - Tibial |
| ENSG00000138379.4 | MSTN | chr2_189199061_T_TTA_b38 | rs10699846 | 0.000029 | 0.33 | Artery - Tibial |
| ENSG00000138379.4 | MSTN | chr2_189199441_C_A_b38 | rs4667270 | 0.000029 | 0.33 | Artery - Tibial |
| ENSG00000138379.4 | MSTN | chr2_189201202_A_G_b38 | rs10166385 | 0.000029 | 0.33 | Artery - Tibial |
| ENSG00000138379.4 | MSTN | chr2_189202116_G_A_b38 | rs13413518 | 0.000029 | 0.33 | Artery - Tibial |
| ENSG00000138379.4 | MSTN | chr2_190572298_A_G_b38 | rs62182885 | 0.000030 | 0.33 | Stomach |
| ENSG00000138379.4 | MSTN | chr2_190310913_GT_G_b38 | rs36074132 | 0.000030 | 0.24 | Skin - Sun Exposed (Lower leg) |
| ENSG00000138379.4 | MSTN | chr2_190463126_A_G_b38 | rs59929070 | 0.000030 | 0.37 | Spleen |
| ENSG00000138379.4 | MSTN | chr2_189250168_G_A_b38 | rs6434334 | 0.000034 | 0.33 | Artery - Tibial |
| ENSG00000138379.4 | MSTN | chr2_189268066_C_T_b38 | rs1474000 | 0.000034 | 0.33 | Artery - Tibial |
| ENSG00000138379.4 | MSTN | chr2_189271206_C_T_b38 | rs6761868 | 0.000034 | 0.33 | Artery - Tibial |
| ENSG00000138379.4 | MSTN | chr2_189271331_C_T_b38 | rs6704568 | 0.000034 | 0.33 | Artery - Tibial |
| ENSG00000138379.4 | MSTN | chr2_189278387_C_T_b38 | rs6434337 | 0.000034 | -0.33 | Artery - Tibial |
| ENSG00000138379.4 | MSTN | chr2_189279002_A_T_b38 | rs7574281 | 0.000034 | -0.33 | Artery - Tibial |
| ENSG00000138379.4 | MSTN | chr2_189280576_G_A_b38 | rs12621657 | 0.000034 | -0.33 | Artery - Tibial |
| ENSG00000138379.4 | MSTN | chr2_189286491_A_G_b38 | rs939158 | 0.000034 | -0.33 | Artery - Tibial |
| ENSG00000138379.4 | MSTN | chr2_189287123_A_C_b38 | rs2351635 | 0.000034 | -0.33 | Artery - Tibial |
| ENSG00000138379.4 | MSTN | chr2_190047775_C_G_b38 | rs11693603 | 0.000035 | -0.35 | Brain - Cerebellum |
| ENSG00000138379.4 | MSTN | chr2_190256459_T_C_b38 | rs291409 | 0.000035 | 0.22 | Skin - Sun Exposed (Lower leg) |
| ENSG00000138379.4 | MSTN | chr2_190029027_T_G_b38 | rs1435139 | 0.000035 | 0.29 | Brain - Cerebellum |
| ENSG00000138379.4 | MSTN | chr2_190398003_G_A_b38 | rs9288173 | 0.000036 | 0.18 | Artery - Tibial |
| ENSG00000138379.4 | MSTN | chr2_189323004_A_AG_b38 | rs59246807 | 0.000037 | -0.34 | Artery - Tibial |
| ENSG00000138379.4 | MSTN | chr2_189324609_CA_C_b38 | rs139135494 | 0.000037 | -0.34 | Artery - Tibial |
| ENSG00000138379.4 | MSTN | chr2_189325989_T_C_b38 | rs2683018 | 0.000037 | -0.34 | Artery - Tibial |
| ENSG00000138379.4 | MSTN | chr2_189325990_G_A_b38 | rs2680631 | 0.000037 | -0.34 | Artery - Tibial |
| ENSG00000138379.4 | MSTN | chr2_189331183_A_G_b38 | rs2351932 | 0.000037 | -0.34 | Artery - Tibial |
| ENSG00000138379.4 | MSTN | chr2_189332460_G_T_b38 | rs2683012 | 0.000037 | -0.34 | Artery - Tibial |
| ENSG00000138379.4 | MSTN | chr2_189339387_A_G_b38 | rs10804029 | 0.000037 | -0.34 | Artery - Tibial |
| ENSG00000138379.4 | MSTN | chr2_189361623_G_T_b38 | rs4580411 | 0.000037 | -0.34 | Artery - Tibial |
| ENSG00000138379.4 | MSTN | chr2_189367236_G_C_b38 | rs4667280 | 0.000037 | -0.34 | Artery - Tibial |
| ENSG00000138379.4 | MSTN | chr2_189379860_G_A_b38 | rs6752545 | 0.000037 | -0.34 | Artery - Tibial |
| ENSG00000138379.4 | MSTN | chr2_189384578_C_T_b38 | rs7566093 | 0.000037 | -0.34 | Artery - Tibial |
| ENSG00000138379.4 | MSTN | chr2_189225629_T_C_b38 | rs7603704 | 0.000037 | 0.33 | Artery - Tibial |
| ENSG00000138379.4 | MSTN | chr2_190511849_C_T_b38 | rs75579212 | 0.000037 | 0.32 | Stomach |
| ENSG00000138379.4 | MSTN | chr2_190164699_T_C_b38 | rs12478752 | 0.000037 | -0.18 | Nerve - Tibial |
| ENSG00000138379.4 | MSTN | chr2_190302759_T_C_b38 | rs13388583 | 0.000037 | 0.87 | Esophagus - Muscularis |
| ENSG00000138379.4 | MSTN | chr2_190004330_G_C_b38 | rs72907382 | 0.000038 | -0.35 | Brain - Cerebellum |
| ENSG00000138379.4 | MSTN | chr2_189940845_C_T_b38 | rs7598182 | 0.000038 | -0.34 | Brain - Cerebellum |
| ENSG00000138379.4 | MSTN | chr2_189250614_T_C_b38 | rs10167242 | 0.000039 | 0.33 | Artery - Tibial |
| ENSG00000138379.4 | MSTN | chr2_190158630_G_T_b38 | rs6747241 | 0.000039 | -0.26 | Heart - Left Ventricle |
| ENSG00000138379.4 | MSTN | chr2_190319369_C_G_b38 | rs4146044 | 0.000041 | 0.21 | Skin - Sun Exposed (Lower leg) |
| ENSG00000138379.4 | MSTN | chr2_190445861_A_AT_b38 | rs138381334 | 0.000041 | 0.37 | Stomach |
| ENSG00000138379.4 | MSTN | chr2_190455452_G_A_b38 | rs62181016 | 0.000041 | 0.37 | Stomach |
| ENSG00000138379.4 | MSTN | chr2_190048134_C_CTTGATTT_b38 | rs149112043 | 0.000041 | -0.34 | Brain - Cerebellum |
| ENSG00000138379.4 | MSTN | chr2_190052338_T_G_b38 | rs72909328 | 0.000041 | -0.34 | Brain - Cerebellum |
| ENSG00000138379.4 | MSTN | chr2_190052349_A_G_b38 | rs72909330 | 0.000041 | -0.34 | Brain - Cerebellum |
| ENSG00000138379.4 | MSTN | chr2_190069949_T_C_b38 | rs57566211 | 0.000041 | -0.34 | Brain - Cerebellum |
| ENSG00000138379.4 | MSTN | chr2_190604260_G_C_b38 | rs61117285 | 0.000042 | 0.32 | Stomach |
| ENSG00000138379.4 | MSTN | chr2_190604261_G_A_b38 | rs58568707 | 0.000042 | 0.32 | Stomach |
| ENSG00000138379.4 | MSTN | chr2_190605526_C_G_b38 | rs112979515 | 0.000042 | 0.32 | Stomach |
| ENSG00000138379.4 | MSTN | chr2_190606669_C_T_b38 | rs62182897 | 0.000042 | 0.32 | Stomach |
| ENSG00000138379.4 | MSTN | chr2_190490237_C_A_b38 | rs62181034 | 0.000042 | 0.32 | Stomach |
| ENSG00000138379.4 | MSTN | chr2_190145426_G_A_b38 | rs2562821 | 0.000043 | 0.50 | Heart - Atrial Appendage |
| ENSG00000138379.4 | MSTN | chr2_190145493_T_C_b38 | rs2690730 | 0.000043 | 0.50 | Heart - Atrial Appendage |
| ENSG00000138379.4 | MSTN | chr2_190146521_A_G_b38 | rs785271 | 0.000043 | 0.50 | Heart - Atrial Appendage |
| ENSG00000138379.4 | MSTN | chr2_190146747_T_C_b38 | rs785270 | 0.000043 | 0.50 | Heart - Atrial Appendage |
| ENSG00000138379.4 | MSTN | chr2_190147349_G_A_b38 | rs1085203 | 0.000043 | 0.50 | Heart - Atrial Appendage |
| ENSG00000138379.4 | MSTN | chr2_190147678_CAG_C_b38 | rs71023798 | 0.000043 | 0.50 | Heart - Atrial Appendage |
| ENSG00000138379.4 | MSTN | chr2_190148148_A_T_b38 | rs1085202 | 0.000043 | 0.50 | Heart - Atrial Appendage |
| ENSG00000138379.4 | MSTN | chr2_190148167_C_T_b38 | rs1085201 | 0.000043 | 0.50 | Heart - Atrial Appendage |
| ENSG00000138379.4 | MSTN | chr2_190148319_C_T_b38 | rs1085199 | 0.000043 | 0.50 | Heart - Atrial Appendage |
| ENSG00000138379.4 | MSTN | chr2_190148660_T_C_b38 | rs1085196 | 0.000043 | 0.50 | Heart - Atrial Appendage |
| ENSG00000138379.4 | MSTN | chr2_190148928_A_G_b38 | rs1085194 | 0.000043 | 0.50 | Heart - Atrial Appendage |
| ENSG00000138379.4 | MSTN | chr2_190467443_T_A_b38 | rs7567059 | 0.000043 | 0.39 | Spleen |
| ENSG00000138379.4 | MSTN | chr2_190540554_T_A_b38 | rs10931461 | 0.000043 | -0.31 | Stomach |
| ENSG00000138379.4 | MSTN | chr2_190566015_A_G_b38 | rs4371384 | 0.000043 | -0.31 | Stomach |
| ENSG00000138379.4 | MSTN | chr2_189940936_A_G_b38 | rs7571613 | 0.000043 | -0.33 | Brain - Cerebellum |
| ENSG00000138379.4 | MSTN | chr2_189963353_CCT_C_b38 | rs112072247 | 0.000043 | -0.33 | Brain - Cerebellum |
| ENSG00000138379.4 | MSTN | chr2_189964271_T_G_b38 | rs72907337 | 0.000043 | -0.33 | Brain - Cerebellum |
| ENSG00000138379.4 | MSTN | chr2_189984164_C_A_b38 | rs2196289 | 0.000043 | -0.33 | Brain - Cerebellum |
| ENSG00000138379.4 | MSTN | chr2_190424107_A_G_b38 | rs4577309 | 0.000044 | 0.18 | Artery - Tibial |
| ENSG00000138379.4 | MSTN | chr2_190354338_C_A_b38 | rs1984626 | 0.000044 | 0.19 | Skin - Sun Exposed (Lower leg) |
| ENSG00000138379.4 | MSTN | chr2_190354547_A_G_b38 | rs4853694 | 0.000044 | 0.19 | Skin - Sun Exposed (Lower leg) |
| ENSG00000138379.4 | MSTN | chr2_190355987_T_TA_b38 | rs11412132 | 0.000044 | 0.19 | Skin - Sun Exposed (Lower leg) |
| ENSG00000138379.4 | MSTN | chr2_190356640_A_C_b38 | rs3791816 | 0.000044 | 0.19 | Skin - Sun Exposed (Lower leg) |
| ENSG00000138379.4 | MSTN | chr2_190357784_G_A_b38 | rs2067394 | 0.000044 | 0.19 | Skin - Sun Exposed (Lower leg) |
| ENSG00000138379.4 | MSTN | chr2_190360512_G_C_b38 | rs2067406 | 0.000044 | 0.19 | Skin - Sun Exposed (Lower leg) |
| ENSG00000138379.4 | MSTN | chr2_190362016_A_G_b38 | rs2067416 | 0.000044 | 0.19 | Skin - Sun Exposed (Lower leg) |
| ENSG00000138379.4 | MSTN | chr2_190222714_G_A_b38 | rs12472258 | 0.000044 | -0.24 | Artery - Tibial |
| ENSG00000138379.4 | MSTN | chr2_190006107_C_T_b38 | rs16832244 | 0.000047 | -0.35 | Brain - Cerebellum |
| ENSG00000138379.4 | MSTN | chr2_189219458_A_G_b38 | rs2351632 | 0.000047 | 0.32 | Artery - Tibial |
| ENSG00000138379.4 | MSTN | chr2_190487209_C_G_b38 | rs11885992 | 0.000047 | 0.32 | Stomach |
| ENSG00000138379.4 | MSTN | chr2_189971652_C_T_b38 | rs186955980 | 0.000050 | -0.35 | Brain - Cerebellum |
| ENSG00000138379.4 | MSTN | chr2_189396781_G_T_b38 | rs10931417 | 0.000050 | -0.32 | Artery - Tibial |
| ENSG00000138379.4 | MSTN | chr2_189397626_C_CATT_b38 | rs35736992 | 0.000050 | -0.32 | Artery - Tibial |
| ENSG00000138379.4 | MSTN | chr2_189403545_A_G_b38 | rs9677436 | 0.000050 | -0.32 | Artery - Tibial |
| ENSG00000138379.4 | MSTN | chr2_189407469_T_C_b38 | rs13018252 | 0.000050 | -0.32 | Artery - Tibial |
| ENSG00000138379.4 | MSTN | chr2_189355516_T_TC_b38 | rs140032114 | 0.000050 | -0.33 | Artery - Tibial |
| ENSG00000138379.4 | MSTN | chr2_189355617_G_C_b38 | rs6434340 | 0.000050 | -0.33 | Artery - Tibial |
| ENSG00000138379.4 | MSTN | chr2_190169524_G_A_b38 | rs34280054 | 0.000051 | -0.18 | Nerve - Tibial |
| ENSG00000138379.4 | MSTN | chr2_190170117_G_A_b38 | rs35927363 | 0.000051 | -0.18 | Nerve - Tibial |
| ENSG00000138379.4 | MSTN | chr2_190463714_G_T_b38 | rs56342910 | 0.000051 | 0.36 | Spleen |
| ENSG00000138379.4 | MSTN | chr2_190159073_G_A_b38 | rs6750723 | 0.000052 | -0.25 | Heart - Left Ventricle |
| ENSG00000138379.4 | MSTN | chr2_190422957_A_G_b38 | rs59109438 | 0.000053 | 0.36 | Spleen |
| ENSG00000138379.4 | MSTN | chr2_190422959_A_C_b38 | rs61709084 | 0.000053 | 0.36 | Spleen |
| ENSG00000138379.4 | MSTN | chr2_190256459_T_C_b38 | rs291409 | 0.000054 | 0.25 | Skin - Not Sun Exposed (Suprapubic) |
| ENSG00000138379.4 | MSTN | chr2_190492123_A_G_b38 | rs75488522 | 0.000055 | 0.31 | Stomach |
| ENSG00000138379.4 | MSTN | chr2_190501981_A_G_b38 | rs1128723 | 0.000055 | 0.31 | Stomach |
| ENSG00000138379.4 | MSTN | chr2_190518622_A_G_b38 | rs12053190 | 0.000055 | 0.31 | Stomach |
| ENSG00000138379.4 | MSTN | chr2_190522353_T_G_b38 | rs10432434 | 0.000055 | 0.31 | Stomach |
| ENSG00000138379.4 | MSTN | chr2_190530301_C_T_b38 | rs4258824 | 0.000055 | 0.31 | Stomach |
| ENSG00000138379.4 | MSTN | chr2_190533627_A_C_b38 | rs4439990 | 0.000055 | 0.31 | Stomach |
| ENSG00000138379.4 | MSTN | chr2_190149104_C_A_b38 | rs1085192 | 0.000059 | 0.53 | Heart - Atrial Appendage |
| ENSG00000138379.4 | MSTN | chr2_190014216_A_T_b38 | rs72907391 | 0.000061 | -0.33 | Brain - Cerebellum |
| ENSG00000138379.4 | MSTN | chr2_191014336_T_A_b38 | rs41452847 | 0.000064 | -0.79 | Artery - Tibial |
| ENSG00000138379.4 | MSTN | chr2_190608154_G_A_b38 | rs12476782 | 0.000064 | 0.28 | Stomach |
| ENSG00000138379.4 | MSTN | chr2_190362347_A_G_b38 | rs2067419 | 0.000064 | 0.22 | Skin - Not Sun Exposed (Suprapubic) |
| ENSG00000138379.4 | MSTN | chr2_190581965_C_A_b38 | rs7569128 | 0.000065 | -0.31 | Stomach |
| ENSG00000138379.4 | MSTN | chr2_190597407_C_T_b38 | rs2192010 | 0.000065 | 0.31 | Stomach |
| ENSG00000138379.4 | MSTN | chr2_190467443_T_A_b38 | rs7567059 | 0.000067 | 0.30 | Stomach |
| ENSG00000138379.4 | MSTN | chr2_190354338_C_A_b38 | rs1984626 | 0.000068 | 0.18 | Artery - Tibial |
| ENSG00000138379.4 | MSTN | chr2_190354547_A_G_b38 | rs4853694 | 0.000068 | 0.18 | Artery - Tibial |
| ENSG00000138379.4 | MSTN | chr2_190355987_T_TA_b38 | rs11412132 | 0.000068 | 0.18 | Artery - Tibial |
| ENSG00000138379.4 | MSTN | chr2_190356640_A_C_b38 | rs3791816 | 0.000068 | 0.18 | Artery - Tibial |
| ENSG00000138379.4 | MSTN | chr2_190357784_G_A_b38 | rs2067394 | 0.000068 | 0.18 | Artery - Tibial |
| ENSG00000138379.4 | MSTN | chr2_190360512_G_C_b38 | rs2067406 | 0.000068 | 0.18 | Artery - Tibial |
| ENSG00000138379.4 | MSTN | chr2_190362016_A_G_b38 | rs2067416 | 0.000068 | 0.18 | Artery - Tibial |
| ENSG00000138379.4 | MSTN | chr2_189239171_T_G_b38 | rs10931405 | 0.000070 | 0.32 | Artery - Tibial |
| ENSG00000138379.4 | MSTN | chr2_190359482_G_A_b38 | rs2067398 | 0.000071 | 0.22 | Skin - Not Sun Exposed (Suprapubic) |
| ENSG00000138379.4 | MSTN | chr2_190362206_T_C_b38 | rs2067418 | 0.000071 | 0.22 | Skin - Not Sun Exposed (Suprapubic) |
| ENSG00000138379.4 | MSTN | chr2_190347505_C_T_b38 | rs6721431 | 0.000073 | -0.18 | Skin - Sun Exposed (Lower leg) |
| ENSG00000138379.4 | MSTN | chr2_190348263_A_T_b38 | rs2736621 | 0.000073 | -0.18 | Skin - Sun Exposed (Lower leg) |
| ENSG00000138379.4 | MSTN | chr2_190349249_C_T_b38 | rs2736622 | 0.000073 | -0.18 | Skin - Sun Exposed (Lower leg) |
| ENSG00000138379.4 | MSTN | chr2_189936232_C_T_b38 | rs11684699 | 0.000075 | -0.33 | Brain - Cerebellum |
| ENSG00000138379.4 | MSTN | chr2_190482672_CT_C_b38 | rs59305081 | 0.000075 | 0.30 | Stomach |
| ENSG00000138379.4 | MSTN | chr2_190074739_A_C_b38 | rs12472193 | 0.000079 | 0.27 | Brain - Cerebellum |
| ENSG00000138379.4 | MSTN | chr2_190031057_G_A_b38 | rs6760268 | 0.000079 | -0.33 | Brain - Cerebellum |
| ENSG00000138379.4 | MSTN | chr2_190187167_T_TAG_b38 | rs57586613 | 0.000080 | 0.76 | Esophagus - Muscularis |
| ENSG00000138379.4 | MSTN | chr2_190628440_C_T_b38 | rs887695 | 0.000082 | 0.18 | Artery - Tibial |
| ENSG00000138379.4 | MSTN | chr2_189553964_C_T_b38 | rs12693541 | 0.000083 | -0.24 | Skin - Sun Exposed (Lower leg) |
| ENSG00000138379.4 | MSTN | chr2_190346887_G_A_b38 | rs979329 | 0.000085 | -0.18 | Skin - Sun Exposed (Lower leg) |
| ENSG00000138379.4 | MSTN | chr2_189554593_G_C_b38 | rs10165059 | 0.000087 | 0.46 | Skin - Sun Exposed (Lower leg) |
| ENSG00000138379.4 | MSTN | chr2_190204504_A_G_b38 | rs12618060 | 0.000091 | -0.23 | Artery - Tibial |
| ENSG00000138379.4 | MSTN | chr2_190348938_A_G_b38 | rs1882892 | 0.000091 | -0.18 | Artery - Tibial |
| ENSG00000138379.4 | MSTN | chr2_189995337_G_A_b38 | rs11900658 | 0.000093 | -0.32 | Brain - Cerebellum |
| ENSG00000138379.4 | MSTN | chr2_190019764_G_T_b38 | rs72907398 | 0.000093 | -0.32 | Brain - Cerebellum |
| ENSG00000138379.4 | MSTN | chr2_190022193_C_T_b38 | rs72907401 | 0.000093 | -0.32 | Brain - Cerebellum |
| ENSG00000138379.4 | MSTN | chr2_190025336_C_T_b38 | rs11900530 | 0.000093 | -0.32 | Brain - Cerebellum |
| ENSG00000138379.4 | MSTN | chr2_190031119_G_T_b38 | rs6760285 | 0.000093 | -0.32 | Brain - Cerebellum |
| ENSG00000138379.4 | MSTN | chr2_190068280_T_C_b38 | rs1474359 | 0.000093 | 0.27 | Brain - Cerebellum |
| ENSG00000138379.4 | MSTN | chr2_190068577_C_T_b38 | rs1967606 | 0.000093 | 0.27 | Brain - Cerebellum |
| ENSG00000138379.4 | MSTN | chr2_190458569_C_T_b38 | rs62181018 | 0.000097 | 0.35 | Spleen |
| ENSG00000138379.4 | MSTN | chr2_190460875_A_C_b38 | rs62181020 | 0.000097 | 0.35 | Spleen |
| ENSG00000138379.4 | MSTN | chr2_190346887_G_A_b38 | rs979329 | 0.000098 | -0.18 | Artery - Tibial |
| ENSG00000138379.4 | MSTN | chr2_190347505_C_T_b38 | rs6721431 | 0.000098 | -0.18 | Artery - Tibial |
| ENSG00000138379.4 | MSTN | chr2_190348263_A_T_b38 | rs2736621 | 0.000098 | -0.18 | Artery - Tibial |
| ENSG00000138379.4 | MSTN | chr2_190349249_C_T_b38 | rs2736622 | 0.000098 | -0.18 | Artery - Tibial |
| ENSG00000138379.4 | MSTN | chr2_190354191_T_C_b38 | rs2015744 | 0.00010 | -0.18 | Skin - Sun Exposed (Lower leg) |
| ENSG00000138379.4 | MSTN | chr2_190156124_G_A_b38 | rs6738692 | 0.00010 | -0.25 | Heart - Left Ventricle |
| ENSG00000138379.4 | MSTN | chr2_190251121_T_C_b38 | rs713427 | 0.00010 | -0.23 | Artery - Tibial |
| ENSG00000138379.4 | MSTN | chr2_190151766_C_A_b38 | rs57856620 | 0.00010 | -0.25 | Heart - Left Ventricle |
| ENSG00000138379.4 | MSTN | chr2_190275561_C_T_b38 | rs2664244 | 0.00011 | -0.21 | Skin - Not Sun Exposed (Suprapubic) |
| ENSG00000138379.4 | MSTN | chr2_190276867_T_C_b38 | rs2136567 | 0.00011 | -0.21 | Skin - Not Sun Exposed (Suprapubic) |
| ENSG00000138379.4 | MSTN | chr2_190038380_T_A_b38 | rs11693478 | 0.00011 | -0.32 | Brain - Cerebellum |
| ENSG00000138379.4 | MSTN | chr2_190460688_A_G_b38 | rs62181019 | 0.00011 | 0.34 | Spleen |
| ENSG00000138379.4 | MSTN | chr2_190462421_T_C_b38 | rs4287794 | 0.00011 | 0.34 | Spleen |
| ENSG00000138379.4 | MSTN | chr2_190463126_A_G_b38 | rs59929070 | 0.00011 | 0.29 | Stomach |
| ENSG00000138379.4 | MSTN | chr2_190621355_C_T_b38 | rs72917118 | 0.00011 | -0.23 | Thyroid |
| ENSG00000138379.4 | MSTN | chr2_190156171_A_T_b38 | rs6709775 | 0.00011 | -0.24 | Heart - Left Ventricle |
| ENSG00000138379.4 | MSTN | chr2_190163572_A_G_b38 | rs6738688 | 0.00011 | -0.24 | Heart - Left Ventricle |
| ENSG00000138379.4 | MSTN | chr2_190233286_T_G_b38 | rs6723792 | 0.00011 | 0.21 | Skin - Not Sun Exposed (Suprapubic) |
| ENSG00000138379.4 | MSTN | chr2_190463714_G_T_b38 | rs56342910 | 0.00011 | 0.29 | Stomach |
| ENSG00000138379.4 | MSTN | chr2_190354360_T_G_b38 | rs13411353 | 0.00012 | 0.77 | Esophagus - Muscularis |
| ENSG00000138379.4 | MSTN | chr2_190364474_G_A_b38 | rs62182028 | 0.00012 | -0.23 | Thyroid |
| ENSG00000138379.4 | MSTN | chr2_190354389_T_TTCA_b38 | rs3064189 | 0.00012 | 0.20 | Skin - Not Sun Exposed (Suprapubic) |
| ENSG00000138379.4 | MSTN | chr2_190315381_A_C_b38 | rs1547460 | 0.00012 | -0.23 | Artery - Tibial |
| ENSG00000138379.4 | MSTN | chr2_190320999_G_T_b38 | rs1372053 | 0.00012 | -0.23 | Artery - Tibial |
| ENSG00000138379.4 | MSTN | chr2_190330756_T_C_b38 | rs57199157 | 0.00012 | 0.22 | Skin - Not Sun Exposed (Suprapubic) |
| ENSG00000138379.4 | MSTN | chr2_190271278_C_A_b38 | rs2582766 | 0.00012 | 0.17 | Thyroid |
| ENSG00000138379.4 | MSTN | chr2_189304080_C_T_b38 | rs7425383 | 0.00012 | -0.31 | Artery - Tibial |
| ENSG00000138379.4 | MSTN | chr2_189306282_C_T_b38 | rs10188246 | 0.00012 | -0.31 | Artery - Tibial |
| ENSG00000138379.4 | MSTN | chr2_189307673_C_T_b38 | rs12994482 | 0.00012 | -0.31 | Artery - Tibial |
| ENSG00000138379.4 | MSTN | chr2_189308496_A_C_b38 | rs11676905 | 0.00012 | -0.31 | Artery - Tibial |
| ENSG00000138379.4 | MSTN | chr2_190357387_T_C_b38 | rs2067392 | 0.00013 | 0.18 | Skin - Sun Exposed (Lower leg) |
| ENSG00000138379.4 | MSTN | chr2_190357605_C_A_b38 | rs2067393 | 0.00013 | 0.18 | Skin - Sun Exposed (Lower leg) |
| ENSG00000138379.4 | MSTN | chr2_190358021_T_C_b38 | rs2067395 | 0.00013 | 0.18 | Skin - Sun Exposed (Lower leg) |
| ENSG00000138379.4 | MSTN | chr2_190359324_G_C_b38 | rs2067397 | 0.00013 | 0.18 | Skin - Sun Exposed (Lower leg) |
| ENSG00000138379.4 | MSTN | chr2_190359671_T_C_b38 | rs2067400 | 0.00013 | 0.18 | Skin - Sun Exposed (Lower leg) |
| ENSG00000138379.4 | MSTN | chr2_190359682_C_CCT_b38 | rs113646908 | 0.00013 | 0.18 | Skin - Sun Exposed (Lower leg) |
| ENSG00000138379.4 | MSTN | chr2_190359685_A_G_b38 | rs71217541 | 0.00013 | 0.18 | Skin - Sun Exposed (Lower leg) |
| ENSG00000138379.4 | MSTN | chr2_190360255_G_T_b38 | rs4656 | 0.00013 | 0.18 | Skin - Sun Exposed (Lower leg) |
| ENSG00000138379.4 | MSTN | chr2_190360471_A_G_b38 | rs2067405 | 0.00013 | 0.18 | Skin - Sun Exposed (Lower leg) |
| ENSG00000138379.4 | MSTN | chr2_190360551_A_G_b38 | rs2067407 | 0.00013 | 0.18 | Skin - Sun Exposed (Lower leg) |
| ENSG00000138379.4 | MSTN | chr2_190360580_C_T_b38 | rs2067408 | 0.00013 | 0.18 | Skin - Sun Exposed (Lower leg) |
| ENSG00000138379.4 | MSTN | chr2_190360676_C_G_b38 | rs2067409 | 0.00013 | 0.18 | Skin - Sun Exposed (Lower leg) |
| ENSG00000138379.4 | MSTN | chr2_190361056_A_G_b38 | rs2067412 | 0.00013 | 0.18 | Skin - Sun Exposed (Lower leg) |
| ENSG00000138379.4 | MSTN | chr2_190361140_AG_A_b38 | rs11321941 | 0.00013 | 0.18 | Skin - Sun Exposed (Lower leg) |
| ENSG00000138379.4 | MSTN | chr2_190361214_C_T_b38 | rs2067414 | 0.00013 | 0.18 | Skin - Sun Exposed (Lower leg) |
| ENSG00000138379.4 | MSTN | chr2_190361886_C_T_b38 | rs2067415 | 0.00013 | 0.18 | Skin - Sun Exposed (Lower leg) |
| ENSG00000138379.4 | MSTN | chr2_190362160_A_G_b38 | rs2067417 | 0.00013 | 0.18 | Skin - Sun Exposed (Lower leg) |
| ENSG00000138379.4 | MSTN | chr2_190362509_T_G_b38 | rs2067421 | 0.00013 | 0.18 | Skin - Sun Exposed (Lower leg) |
| ENSG00000138379.4 | MSTN | chr2_190362766_A_G_b38 | rs972689 | 0.00013 | 0.18 | Skin - Sun Exposed (Lower leg) |
| ENSG00000138379.4 | MSTN | chr2_190363029_A_G_b38 | rs972691 | 0.00013 | 0.18 | Skin - Sun Exposed (Lower leg) |
| ENSG00000138379.4 | MSTN | chr2_190363152_T_C_b38 | rs972692 | 0.00013 | 0.18 | Skin - Sun Exposed (Lower leg) |
| ENSG00000138379.4 | MSTN | chr2_190363154_A_C_b38 | rs2067423 | 0.00013 | 0.18 | Skin - Sun Exposed (Lower leg) |
| ENSG00000138379.4 | MSTN | chr2_190363350_A_G_b38 | rs2067424 | 0.00013 | 0.18 | Skin - Sun Exposed (Lower leg) |
| ENSG00000138379.4 | MSTN | chr2_190363613_G_A_b38 | rs2067425 | 0.00013 | 0.18 | Skin - Sun Exposed (Lower leg) |
| ENSG00000138379.4 | MSTN | chr2_190363694_C_A_b38 | rs2067427 | 0.00013 | 0.18 | Skin - Sun Exposed (Lower leg) |
| ENSG00000138379.4 | MSTN | chr2_190363701_G_T_b38 | rs6707681 | 0.00013 | 0.18 | Skin - Sun Exposed (Lower leg) |
| ENSG00000138379.4 | MSTN | chr2_190363799_C_T_b38 | rs6707602 | 0.00013 | 0.18 | Skin - Sun Exposed (Lower leg) |
| ENSG00000138379.4 | MSTN | chr2_190258124_A_G_b38 | rs77315392 | 0.00013 | 0.22 | Skin - Not Sun Exposed (Suprapubic) |
| ENSG00000138379.4 | MSTN | chr2_190265022_C_T_b38 | rs74661763 | 0.00013 | 0.22 | Skin - Not Sun Exposed (Suprapubic) |
| ENSG00000138379.4 | MSTN | chr2_190286932_T_C_b38 | rs16832512 | 0.00013 | 0.22 | Skin - Not Sun Exposed (Suprapubic) |
| ENSG00000138379.4 | MSTN | chr2_190343615_C_T_b38 | rs2028431 | 0.00013 | 0.22 | Skin - Not Sun Exposed (Suprapubic) |
| ENSG00000138379.4 | MSTN | chr2_190314782_T_G_b38 | rs4853693 | 0.00013 | 0.22 | Skin - Not Sun Exposed (Suprapubic) |
| ENSG00000138379.4 | MSTN | chr2_190319798_C_T_b38 | rs3749022 | 0.00013 | 0.19 | Skin - Sun Exposed (Lower leg) |
| ENSG00000138379.4 | MSTN | chr2_190370269_C_T_b38 | rs4853696 | 0.00013 | -0.19 | Skin - Sun Exposed (Lower leg) |
| ENSG00000138379.4 | MSTN | chr2_190360961_C_T_b38 | rs2067411 | 0.00014 | 0.21 | Skin - Not Sun Exposed (Suprapubic) |
| ENSG00000138379.4 | MSTN | chr2_189299235_C_T_b38 | rs7422875 | 0.00014 | -0.31 | Artery - Tibial |
| ENSG00000138379.4 | MSTN | chr2_190231246_C_T_b38 | rs11683636 | 0.00014 | 0.22 | Skin - Not Sun Exposed (Suprapubic) |
| ENSG00000138379.4 | MSTN | chr2_190366777_G_A_b38 | rs4940 | 0.00014 | 0.20 | Skin - Not Sun Exposed (Suprapubic) |
| ENSG00000138379.4 | MSTN | chr2_190340576_C_T_b38 | rs11689196 | 0.00014 | 0.22 | Skin - Not Sun Exposed (Suprapubic) |
| ENSG00000138379.4 | MSTN | chr2_190140584_C_T_b38 | rs11690233 | 0.00015 | 0.18 | Skin - Not Sun Exposed (Suprapubic) |
| ENSG00000138379.4 | MSTN | chr2_190346887_G_A_b38 | rs979329 | 0.00015 | -0.14 | Nerve - Tibial |
| ENSG00000138379.4 | MSTN | chr2_190276153_C_T_b38 | rs1583878 | 0.00015 | -0.20 | Skin - Not Sun Exposed (Suprapubic) |
| ENSG00000138379.4 | MSTN | chr2_190354888_G_A_b38 | rs3791815 | 0.00016 | 0.20 | Skin - Not Sun Exposed (Suprapubic) |
| ENSG00000138379.4 | MSTN | chr2_190355612_C_A_b38 | rs72627906 | 0.00016 | 0.20 | Skin - Not Sun Exposed (Suprapubic) |
| ENSG00000138379.4 | MSTN | chr2_190360026_T_G_b38 | rs2067402 | 0.00016 | 0.20 | Skin - Not Sun Exposed (Suprapubic) |
| ENSG00000138379.4 | MSTN | chr2_190357387_T_C_b38 | rs2067392 | 0.00016 | 0.17 | Artery - Tibial |
| ENSG00000138379.4 | MSTN | chr2_190357605_C_A_b38 | rs2067393 | 0.00016 | 0.17 | Artery - Tibial |
| ENSG00000138379.4 | MSTN | chr2_190358021_T_C_b38 | rs2067395 | 0.00016 | 0.17 | Artery - Tibial |
| ENSG00000138379.4 | MSTN | chr2_190359324_G_C_b38 | rs2067397 | 0.00016 | 0.17 | Artery - Tibial |
| ENSG00000138379.4 | MSTN | chr2_190359671_T_C_b38 | rs2067400 | 0.00016 | 0.17 | Artery - Tibial |
| ENSG00000138379.4 | MSTN | chr2_190359682_C_CCT_b38 | rs113646908 | 0.00016 | 0.17 | Artery - Tibial |
| ENSG00000138379.4 | MSTN | chr2_190359685_A_G_b38 | rs71217541 | 0.00016 | 0.17 | Artery - Tibial |
| ENSG00000138379.4 | MSTN | chr2_190360255_G_T_b38 | rs4656 | 0.00016 | 0.17 | Artery - Tibial |
| ENSG00000138379.4 | MSTN | chr2_190360471_A_G_b38 | rs2067405 | 0.00016 | 0.17 | Artery - Tibial |
| ENSG00000138379.4 | MSTN | chr2_190360551_A_G_b38 | rs2067407 | 0.00016 | 0.17 | Artery - Tibial |
| ENSG00000138379.4 | MSTN | chr2_190360580_C_T_b38 | rs2067408 | 0.00016 | 0.17 | Artery - Tibial |
| ENSG00000138379.4 | MSTN | chr2_190360676_C_G_b38 | rs2067409 | 0.00016 | 0.17 | Artery - Tibial |
| ENSG00000138379.4 | MSTN | chr2_190361056_A_G_b38 | rs2067412 | 0.00016 | 0.17 | Artery - Tibial |
| ENSG00000138379.4 | MSTN | chr2_190361140_AG_A_b38 | rs11321941 | 0.00016 | 0.17 | Artery - Tibial |
| ENSG00000138379.4 | MSTN | chr2_190361214_C_T_b38 | rs2067414 | 0.00016 | 0.17 | Artery - Tibial |
| ENSG00000138379.4 | MSTN | chr2_190361886_C_T_b38 | rs2067415 | 0.00016 | 0.17 | Artery - Tibial |
| ENSG00000138379.4 | MSTN | chr2_190362160_A_G_b38 | rs2067417 | 0.00016 | 0.17 | Artery - Tibial |
| ENSG00000138379.4 | MSTN | chr2_190362509_T_G_b38 | rs2067421 | 0.00016 | 0.17 | Artery - Tibial |
| ENSG00000138379.4 | MSTN | chr2_190362766_A_G_b38 | rs972689 | 0.00016 | 0.17 | Artery - Tibial |
| ENSG00000138379.4 | MSTN | chr2_190363029_A_G_b38 | rs972691 | 0.00016 | 0.17 | Artery - Tibial |
| ENSG00000138379.4 | MSTN | chr2_190363152_T_C_b38 | rs972692 | 0.00016 | 0.17 | Artery - Tibial |
| ENSG00000138379.4 | MSTN | chr2_190363154_A_C_b38 | rs2067423 | 0.00016 | 0.17 | Artery - Tibial |
| ENSG00000138379.4 | MSTN | chr2_190363350_A_G_b38 | rs2067424 | 0.00016 | 0.17 | Artery - Tibial |
| ENSG00000138379.4 | MSTN | chr2_190363613_G_A_b38 | rs2067425 | 0.00016 | 0.17 | Artery - Tibial |
| ENSG00000138379.4 | MSTN | chr2_190363694_C_A_b38 | rs2067427 | 0.00016 | 0.17 | Artery - Tibial |
| ENSG00000138379.4 | MSTN | chr2_190363701_G_T_b38 | rs6707681 | 0.00016 | 0.17 | Artery - Tibial |
| ENSG00000138379.4 | MSTN | chr2_190363799_C_T_b38 | rs6707602 | 0.00016 | 0.17 | Artery - Tibial |
| ENSG00000138379.4 | MSTN | chr2_189985770_G_A_b38 | rs79770098 | 0.00016 | -0.37 | Nerve - Tibial |
| ENSG00000138379.4 | MSTN | chr2_190357202_C_T_b38 | rs2067391 | 0.00016 | 0.17 | Skin - Sun Exposed (Lower leg) |
| ENSG00000138379.4 | MSTN | chr2_190362471_G_A_b38 | rs2067420 | 0.00016 | 0.17 | Skin - Sun Exposed (Lower leg) |
| ENSG00000138379.4 | MSTN | chr2_190363008_A_G_b38 | rs972690 | 0.00016 | 0.17 | Skin - Sun Exposed (Lower leg) |
| ENSG00000138379.4 | MSTN | chr2_190280481_C_T_b38 | rs1876876 | 0.00017 | -0.22 | Artery - Tibial |
| ENSG00000138379.4 | MSTN | chr2_190286130_C_T_b38 | rs12464840 | 0.00017 | -0.22 | Artery - Tibial |
| ENSG00000138379.4 | MSTN | chr2_190286626_G_A_b38 | rs62184406 | 0.00017 | -0.22 | Artery - Tibial |
| ENSG00000138379.4 | MSTN | chr2_190291627_T_C_b38 | rs62184432 | 0.00017 | -0.22 | Artery - Tibial |
| ENSG00000138379.4 | MSTN | chr2_190291698_T_C_b38 | rs35334255 | 0.00017 | -0.22 | Artery - Tibial |
| ENSG00000138379.4 | MSTN | chr2_190292456_C_T_b38 | rs62184435 | 0.00017 | -0.22 | Artery - Tibial |
| ENSG00000138379.4 | MSTN | chr2_190313593_C_T_b38 | rs10931449 | 0.00017 | -0.22 | Artery - Tibial |
| ENSG00000138379.4 | MSTN | chr2_190319749_A_G_b38 | rs291466 | 0.00017 | 0.16 | Thyroid |
| ENSG00000138379.4 | MSTN | chr2_190329333_C_T_b38 | rs291427 | 0.00017 | 0.16 | Thyroid |
| ENSG00000138379.4 | MSTN | chr2_190340251_TA_T_b38 | rs34767025 | 0.00017 | 0.16 | Thyroid |
| ENSG00000138379.4 | MSTN | chr2_190348938_A_G_b38 | rs1882892 | 0.00017 | -0.14 | Nerve - Tibial |
| ENSG00000138379.4 | MSTN | chr2_190135181_T_C_b38 | rs12988305 | 0.00018 | 0.18 | Skin - Not Sun Exposed (Suprapubic) |
| ENSG00000138379.4 | MSTN | chr2_190334001_A_C_b38 | rs291430 | 0.00018 | 0.16 | Thyroid |
| ENSG00000138379.4 | MSTN | chr2_190272068_A_C_b38 | rs2582770 | 0.00018 | 0.16 | Thyroid |
| ENSG00000138379.4 | MSTN | chr2_190247515_G_A_b38 | rs11692448 | 0.00018 | 0.21 | Skin - Not Sun Exposed (Suprapubic) |
| ENSG00000138379.4 | MSTN | chr2_190248633_G_A_b38 | rs55742982 | 0.00018 | 0.21 | Skin - Not Sun Exposed (Suprapubic) |
| ENSG00000138379.4 | MSTN | chr2_190251629_A_G_b38 | rs3791792 | 0.00018 | 0.21 | Skin - Not Sun Exposed (Suprapubic) |
| ENSG00000138379.4 | MSTN | chr2_190216961_G_A_b38 | rs56742312 | 0.00019 | 0.21 | Skin - Not Sun Exposed (Suprapubic) |
| ENSG00000138379.4 | MSTN | chr2_190347505_C_T_b38 | rs6721431 | 0.00019 | -0.14 | Nerve - Tibial |
| ENSG00000138379.4 | MSTN | chr2_190348263_A_T_b38 | rs2736621 | 0.00019 | -0.14 | Nerve - Tibial |
| ENSG00000138379.4 | MSTN | chr2_190349249_C_T_b38 | rs2736622 | 0.00019 | -0.14 | Nerve - Tibial |
| ENSG00000138379.4 | MSTN | chr2_190329955_C_T_b38 | rs12615897 | 0.00019 | -0.22 | Artery - Tibial |
| ENSG00000138379.4 | MSTN | chr2_190283832_G_A_b38 | rs2664253 | 0.00019 | 0.16 | Thyroid |
| ENSG00000138379.4 | MSTN | chr2_190299502_G_GAT_b38 | rs74420213 | 0.00019 | -0.20 | Skin - Not Sun Exposed (Suprapubic) |
| ENSG00000138379.4 | MSTN | chr2_189159234_A_G_b38 | rs10191420 | 0.00020 | 0.36 | Artery - Tibial |
| ENSG00000138379.4 | MSTN | chr2_189160936_T_C_b38 | rs60251658 | 0.00020 | 0.36 | Artery - Tibial |
| ENSG00000138379.4 | MSTN | chr2_190292176_T_G_b38 | rs12621421 | 0.00020 | -0.22 | Artery - Tibial |
| ENSG00000138379.4 | MSTN | chr2_190352531_A_G_b38 | rs2016037 | 0.00020 | -0.18 | Artery - Tibial |
| ENSG00000138379.4 | MSTN | chr2_190237523_G_T_b38 | rs11684995 | 0.00021 | 0.21 | Skin - Not Sun Exposed (Suprapubic) |
| ENSG00000138379.4 | MSTN | chr2_190238070_A_C_b38 | rs2099127 | 0.00021 | 0.21 | Skin - Not Sun Exposed (Suprapubic) |
| ENSG00000138379.4 | MSTN | chr2_190284343_A_C_b38 | rs3791800 | 0.00021 | 0.21 | Skin - Not Sun Exposed (Suprapubic) |
| ENSG00000138379.4 | MSTN | chr2_190237806_G_A_b38 | rs11685119 | 0.00021 | 0.21 | Skin - Not Sun Exposed (Suprapubic) |
| ENSG00000138379.4 | MSTN | chr2_190351383_T_A_b38 | rs2582753 | 0.00021 | -0.20 | Skin - Not Sun Exposed (Suprapubic) |
| ENSG00000138379.4 | MSTN | chr2_190299502_G_GAT_b38 | rs74420213 | 0.00021 | -0.15 | Nerve - Tibial |
| ENSG00000138379.4 | MSTN | chr2_190321471_CAT_C_b38 | rs79576023 | 0.00021 | 0.21 | Skin - Not Sun Exposed (Suprapubic) |
| ENSG00000138379.4 | MSTN | chr2_190325327_C_G_b38 | rs2354871 | 0.00021 | 0.21 | Skin - Not Sun Exposed (Suprapubic) |
| ENSG00000138379.4 | MSTN | chr2_190328370_AC_A_b38 | rs79455790 | 0.00021 | 0.21 | Skin - Not Sun Exposed (Suprapubic) |
| ENSG00000138379.4 | MSTN | chr2_190332009_T_A_b38 | rs58400804 | 0.00021 | 0.21 | Skin - Not Sun Exposed (Suprapubic) |
| ENSG00000138379.4 | MSTN | chr2_190336838_A_C_b38 | rs67791002 | 0.00021 | 0.21 | Skin - Not Sun Exposed (Suprapubic) |
| ENSG00000138379.4 | MSTN | chr2_190410852_T_TC_b38 | rs200461307 | 0.00022 | -0.23 | Thyroid |
| ENSG00000138379.4 | MSTN | chr2_190411009_C_T_b38 | rs112699106 | 0.00022 | -0.23 | Thyroid |
| ENSG00000138379.4 | MSTN | chr2_190424785_A_G_b38 | rs72905203 | 0.00022 | -0.23 | Thyroid |
| ENSG00000138379.4 | MSTN | chr2_190301486_G_A_b38 | rs906916 | 0.00023 | 0.16 | Thyroid |
| ENSG00000138379.4 | MSTN | chr2_189133613_C_T_b38 | rs6752781 | 0.00023 | -0.26 | Artery - Tibial |
| ENSG00000138379.4 | MSTN | chr2_189161292_G_C_b38 | rs1016622 | 0.00023 | -0.26 | Artery - Tibial |
| ENSG00000138379.4 | MSTN | chr2_190142664_G_A_b38 | rs4853675 | 0.00023 | 0.17 | Skin - Not Sun Exposed (Suprapubic) |
| ENSG00000138379.4 | MSTN | chr2_190143478_C_T_b38 | rs12478659 | 0.00023 | 0.17 | Skin - Not Sun Exposed (Suprapubic) |
| ENSG00000138379.4 | MSTN | chr2_189584697_C_T_b38 | rs77284543 | 0.00024 | -0.36 | Nerve - Tibial |
| ENSG00000138379.4 | MSTN | chr2_190262845_G_C_b38 | rs3791795 | 0.00024 | 0.20 | Skin - Not Sun Exposed (Suprapubic) |
| ENSG00000138379.4 | MSTN | chr2_190319798_C_T_b38 | rs3749022 | 0.00024 | 0.17 | Thyroid |
| ENSG00000138379.4 | MSTN | chr2_189185054_T_C_b38 | rs13028226 | 0.00024 | -0.22 | Artery - Tibial |
| ENSG00000138379.4 | MSTN | chr2_189188285_G_A_b38 | rs1356166 | 0.00024 | -0.22 | Artery - Tibial |
| ENSG00000138379.4 | MSTN | chr2_189188971_A_T_b38 | rs1983318 | 0.00024 | -0.22 | Artery - Tibial |
| ENSG00000138379.4 | MSTN | chr2_189192751_C_T_b38 | rs6728877 | 0.00024 | -0.22 | Artery - Tibial |
| ENSG00000138379.4 | MSTN | chr2_189193735_T_C_b38 | rs12999125 | 0.00024 | -0.22 | Artery - Tibial |
| ENSG00000138379.4 | MSTN | chr2_189193768_G_T_b38 | rs1546617 | 0.00024 | -0.22 | Artery - Tibial |
| ENSG00000138379.4 | MSTN | chr2_189197907_C_T_b38 | rs1973666 | 0.00024 | -0.22 | Artery - Tibial |
| ENSG00000138379.4 | MSTN | chr2_189198735_T_TAA_b38 | rs34159739 | 0.00024 | -0.22 | Artery - Tibial |
| ENSG00000138379.4 | MSTN | chr2_190299058_T_C_b38 | rs2582746 | 0.00024 | 0.16 | Thyroid |
| ENSG00000138379.4 | MSTN | chr2_190357202_C_T_b38 | rs2067391 | 0.00024 | 0.17 | Artery - Tibial |
| ENSG00000138379.4 | MSTN | chr2_190362471_G_A_b38 | rs2067420 | 0.00024 | 0.17 | Artery - Tibial |
| ENSG00000138379.4 | MSTN | chr2_190363008_A_G_b38 | rs972690 | 0.00024 | 0.17 | Artery - Tibial |
| ENSG00000138379.4 | MSTN | chr2_190333215_G_A_b38 | rs291429 | 0.00025 | 0.16 | Thyroid |
| ENSG00000138379.4 | MSTN | chr2_190300703_C_T_b38 | rs60469543 | 0.00025 | 0.20 | Skin - Not Sun Exposed (Suprapubic) |
| ENSG00000138379.4 | MSTN | chr2_190308058_C_G_b38 | rs3791805 | 0.00026 | 0.20 | Skin - Not Sun Exposed (Suprapubic) |
| ENSG00000138379.4 | MSTN | chr2_190317355_C_T_b38 | rs3791806 | 0.00026 | 0.20 | Skin - Not Sun Exposed (Suprapubic) |
| ENSG00000138379.4 | MSTN | chr2_190222032_C_T_b38 | rs66867328 | 0.00026 | 0.20 | Skin - Not Sun Exposed (Suprapubic) |
| ENSG00000138379.4 | MSTN | chr2_190228379_C_T_b38 | rs35214387 | 0.00026 | 0.20 | Skin - Not Sun Exposed (Suprapubic) |
| ENSG00000138379.4 | MSTN | chr2_189148420_G_A_b38 | rs9288163 | 0.00026 | 0.25 | Artery - Tibial |
| ENSG00000138379.4 | MSTN | chr2_190294130_A_C_b38 | rs291450 | 0.00027 | -0.15 | Nerve - Tibial |
| ENSG00000138379.4 | MSTN | chr2_190394308_A_G_b38 | rs55966183 | 0.00027 | -0.22 | Artery - Tibial |
| ENSG00000138379.4 | MSTN | chr2_190283621_G_A_b38 | rs2664252 | 0.00027 | 0.16 | Thyroid |
| ENSG00000138379.4 | MSTN | chr2_190327505_G_T_b38 | rs17800251 | 0.00027 | 0.42 | Skin - Sun Exposed (Lower leg) |
| ENSG00000138379.4 | MSTN | chr2_190342721_T_C_b38 | rs2067385 | 0.00027 | 0.42 | Skin - Sun Exposed (Lower leg) |
| ENSG00000138379.4 | MSTN | chr2_190353645_A_G_b38 | rs3791813 | 0.00027 | 0.20 | Skin - Not Sun Exposed (Suprapubic) |
| ENSG00000138379.4 | MSTN | chr2_190275143_T_C_b38 | rs7599085 | 0.00027 | 0.21 | Skin - Not Sun Exposed (Suprapubic) |
| ENSG00000138379.4 | MSTN | chr2_190276772_T_C_b38 | rs72627905 | 0.00027 | 0.21 | Skin - Not Sun Exposed (Suprapubic) |
| ENSG00000138379.4 | MSTN | chr2_190288288_A_C_b38 | rs4853689 | 0.00027 | 0.21 | Skin - Not Sun Exposed (Suprapubic) |
| ENSG00000138379.4 | MSTN | chr2_190300243_G_A_b38 | rs6752703 | 0.00027 | 0.21 | Skin - Not Sun Exposed (Suprapubic) |
| ENSG00000138379.4 | MSTN | chr2_190157718_A_G_b38 | rs12474543 | 0.00027 | -0.17 | Nerve - Tibial |
| ENSG00000138379.4 | MSTN | chr2_190304977_TCTAA_T_b38 | rs138777105 | 0.00027 | -0.19 | Skin - Not Sun Exposed (Suprapubic) |
| ENSG00000138379.4 | MSTN | chr2_189122343_G_A_b38 | rs4667263 | 0.00028 | -0.25 | Artery - Tibial |
| ENSG00000138379.4 | MSTN | chr2_190280166_A_T_b38 | rs2582736 | 0.00029 | 0.16 | Thyroid |
| ENSG00000138379.4 | MSTN | chr2_190280548_G_A_b38 | rs1876877 | 0.00029 | 0.16 | Thyroid |
| ENSG00000138379.4 | MSTN | chr2_190366777_G_A_b38 | rs4940 | 0.00029 | 0.18 | Artery - Tibial |
| ENSG00000138379.4 | MSTN | chr2_189539788_T_C_b38 | rs11896326 | 0.00029 | 0.22 | Skin - Sun Exposed (Lower leg) |
| ENSG00000138379.4 | MSTN | chr2_189143466_C_T_b38 | rs13005834 | 0.00029 | -0.25 | Artery - Tibial |
| ENSG00000138379.4 | MSTN | chr2_189146692_G_A_b38 | rs4667266 | 0.00029 | -0.25 | Artery - Tibial |
| ENSG00000138379.4 | MSTN | chr2_190352531_A_G_b38 | rs2016037 | 0.00030 | -0.14 | Nerve - Tibial |
| ENSG00000138379.4 | MSTN | chr2_190268043_A_G_b38 | rs3791799 | 0.00030 | -0.21 | Artery - Tibial |
| ENSG00000138379.4 | MSTN | chr2_190275282_T_C_b38 | rs76451356 | 0.00030 | -0.21 | Artery - Tibial |
| ENSG00000138379.4 | MSTN | chr2_190282369_G_A_b38 | rs59090097 | 0.00030 | -0.21 | Artery - Tibial |
| ENSG00000138379.4 | MSTN | chr2_190283149_T_C_b38 | rs10931447 | 0.00030 | -0.21 | Artery - Tibial |
| ENSG00000138379.4 | MSTN | chr2_190303188_T_G_b38 | rs12466290 | 0.00030 | -0.21 | Artery - Tibial |
| ENSG00000138379.4 | MSTN | chr2_190354191_T_C_b38 | rs2015744 | 0.00030 | -0.16 | Artery - Tibial |
| ENSG00000138379.4 | MSTN | chr2_189163763_A_G_b38 | rs11901914 | 0.00030 | 0.25 | Artery - Tibial |
| ENSG00000138379.4 | MSTN | chr2_190382090_T_TA_b38 | rs35236491 | 0.00033 | -0.19 | Skin - Sun Exposed (Lower leg) |

**Supplementary Table S3. Significant single-tissue *cis*-eQTLs of *MSTN* in the GTEx project.** A p-value less than 1.0 x 10^-5^ was considered statistically significant.

| ***cis-*eQTL** | **eQTL p-value** | ***cis*-eQTL tissue** |
| --- | --- | --- |
| rs12618060 | 2.40E-07 | Sun exposed skin (lower leg) |
| rs36074132 | 3.10E-07 | Thyroid |
| rs7571669 | 3.60E-07 | Cerebellum |
| rs10184474 | 0.000011 | Tibial artery |
| rs75579212 | 0.000011 | Spleen |
| rs200611893 | 0.000018 | Stomach |
| rs1366675 | 0.000027 | Atrial appendage (heart) |
| rs12478752 | 0.000037 | Tibial nerve |
| rs13388583 | 0.000037 | Esophagus muscularis |
| rs291409 | 0.000054 | Unexposed skin (suprapubic) |
| rs6747241 | 0.000039 | Left ventricle (heart) |

**Supplementary Table S4. The most significant tissue-specific *MSTN cis*-eQTL in the GTEx project.** Tissues with available expression data are listed in the table.

| **Trait** | **P-value** | **Beta** | **Odds Ratio** | **PMID** | **Author (Year)** | **N Cases** | **N Overall** |
| --- | --- | --- | --- | --- | --- | --- | --- |
| Mean corpuscular volume | 7.85E-30 | -0.021667 |  | PMID:32888493 | Chen MH |  | 544127 |
| Mean corpuscular hemoglobin concentration | 7.8E-17 | 0.017542 |  | PMID:32888493 | Chen MH |  | 491553 |
| Eosinophil counts | 9.5E-26 | -0.021909 |  | PMID:32888493 | Chen MH |  | 474237 |
| Mean platelet volume | 9.73E-90 | -0.040507 |  | PMID:32888493 | Chen MH |  | 460935 |
| Mean spheric corpuscular volume | 4.7E-36 | -0.0273573 |  | PMID:32888494 | Vuckovic D |  | 408112 |
| Lymphocyte percentage of white cells | 0.000005 | 0.0103317 |  | PMID:32888494 | Vuckovic D |  | 408112 |
| Monocyte percentage of white cells | 8.2E-30 | 0.0253324 |  | PMID:32888494 | Vuckovic D |  | 408112 |
| Hematocrit | 1.3E-27 | 0.0244156 |  | PMID:32888494 | Vuckovic D |  | 408112 |
| Valvular heart disease including rheumatic fever | 0.00201 | -0.026 | 0.97433509 |  | FINNGEN_R6 | 47003 | 229974 |
| Chronic sinusitis | 0.000353 | -0.05417 | 0.947271057 |  | FINNGEN_R6 | 10435 | 209643 |
| Venous thromboembolism | 0.00000914 | -0.06507 | 0.937001871 |  | FINNGEN_R6 | 11288 | 260405 |
| Benign neoplasm: Colon | 5.05E-07 | -0.07998 | 0.923134809 |  | FINNGEN_R6 | 9208 | 260405 |
| Disorders of adult personality and behaviour | 0.00068 | 0.06184 | 1.063792124 |  | FINNGEN_R6 | 7528 | 260405 |
| Ohter specific/unspecified arthritis | 0.00114 | -0.06524 | 0.936842594 |  | FINNGEN_R6 | 5498 | 178912 |
| Personality disorders | 0.0018 | 0.05807 | 1.059789178 |  | FINNGEN_R6 | 7121 | 260405 |
| Lost weight | weight change during worst episode of depression | 0.0046684 | 0.03650242 | 1.037176814 |  | UKB Neale v2 | 20313 | 56571 |
| Decaffeinated coffee (any type) | coffee type | 0.00014135 | -0.02663713 | 0.973714506 |  | UKB Neale v2 | 55310 | 283449 |
| Sum basophil neutrophil counts | 2.201E-10 | -0.0238862 |  | PMID:27863252 | Astle WJ |  | 170143 |
| Blonde | hair colour (natural, before greying) | 2.72928E-06 | 0.036429087 | 1.037100757 |  | UKB Neale v2 | 41178 | 360270 |
| Platelet distribution width | 0.001459 | 0.01200052 |  | PMID:27863252 | Astle WJ |  | 164433 |
| None of the above | blood clot, dvt, bronchitis, emphysema, asthma, rhinitis, eczema, allergy diagnosed by doctor | 1.4947E-06 | 0.025444857 | 1.02577134 |  | UKB Neale v2 | 116005 | 360527 |
| Cholelithiasis/gall stones | non-cancer illness code, self-reported | 0.00181024 | 0.060536162 | 1.062406015 |  | UKB Neale v2 | 5941 | 361141 |
| Hypothyroidism/myxoedema | non-cancer illness code, self-reported | 3.33796E-07 | -0.05799254 | 0.943656985 |  | UKB Neale v2 | 17574 | 361141 |
| Bowel cancer | illnesses of siblings | 0.00306101 | -0.05304239 | 0.948339814 |  | UKB Neale v2 | 6727 | 279242 |
| Taking other prescription medications | 0.000109262 | -0.01878456 | 0.981390767 |  | UKB Neale v2 | 168993 | 360027 |
| Had other major operations | 0.00288518 | 0.021373723 | 1.021603777 |  | UKB Neale v2 | 63035 | 192470 |
| Ulcerative rectosigmoiditis | 0.00441 | 0.10765 | 1.113657897 |  | FINNGEN_R6 | 1494 | 251199 |
| Facial ageing | 4.88715E-10 | -0.00788302 |  |  | UKB Neale v2 |  | 330409 |
| Monocyte percentage | 1.01816E-13 | 0.0496902 |  |  | UKB Neale v2 |  | 349861 |
| Nitrogen dioxide air pollution; 2005 | 0.00290638 | 0.0700073 |  |  | UKB Neale v2 |  | 356078 |
| Mean sphered cell volume | 2.86595E-16 | -0.108419 |  |  | UKB Neale v2 |  | 344729 |
| Lymphocyte percentage | 0.000575673 | 0.0626131 |  |  | UKB Neale v2 |  | 349861 |
| Red blood cell (erythrocyte) distribution width | 9.05821E-63 | -0.0396852 |  |  | UKB Neale v2 |  | 350473 |
| Heel quantitative ultrasound index (qui), direct entry | 7.21917E-05 | 0.271471 |  |  | UKB Neale v2 |  | 206589 |
| Haematocrit percentage | 2.2272E-19 | 0.0650302 |  |  | UKB Neale v2 |  | 350475 |
| Dried fruit intake | 0.00205531 | -0.0060927 |  |  | UKB Neale v2 |  | 329134 |
| Frequency of walking for pleasure in last 4 weeks | 0.000211499 | -0.0160965 |  |  | UKB Neale v2 |  | 257478 |
| Total testosterone levels | 0.00002 | -0.00568245 |  | PMID:32042192 | Ruth KS |  | 425097 |
| Total testosterone levels | 2.6E-07 | -0.0163735 |  | PMID:32042192 | Ruth KS |  | 194453 |
| Sex hormone-binding globulin levels | 5.5E-09 | -0.00743462 |  | PMID:32042192 | Ruth KS |  | 180726 |
| Time spent driving | 0.000198693 | -0.00816957 |  |  | UKB Neale v2 |  | 241357 |
| Dermatologic disease | 0.00025 | 0.00154981 | 1.001551012 | PMID:33959723 | Donertas HM | 20101 | 484598 |
| Nasal polyps | 0.0000341 | -0.109 | 0.896730417 |  | UKB SAIGE | 3311 | 393356 |
| Intelligence | 0.00001595 | 0.012461735 |  | PMID:29942086 | Savage JE |  | 269867 |
| Crohn's disease | 8.132E-07 | 0.0826 | 1.086107279 | PMID:28067908 | de Lange KM | 12194 | 40266 |
| Basal cell carcinoma | 0.00093 | -0.03798207 | 0.962730207 | PMID:33549134 | Adolphe C | 17416 | 392871 |
| Appendicular lean mass | 6.25E-10 | -0.0123 |  | PMID:33097823 | Pei YF |  | 450243 |
| Other allied disorders of spine | 0.000677 | -0.27 | 0.763379494 |  | UKB SAIGE | 351 | 392268 |
| Prostaglandin E synthase 2 measurement | 0.0008189 | 0.0457 |  | PMID:33328453 | Pietzner M |  | 10708 |
| Type 2 diabetes | 0.001888 | -0.0236 | 0.976676302 | PMID:30054458 | Xue A | 62892 | 659316 |
| Bipolar disorder vs autism spectrum disorder (ordinary least squares (OLS)) | 0.00003 | 0.013 | 1.013084867 | PMID:33686288 | Peyrot WJ | 38733 | 38733 |
| Bipolar disorder vs anorexia nervosa (ordinary least squares (OLS)) | 0.000055 | 0.015 | 1.015113065 | PMID:33686288 | Peyrot WJ | 37344 | 37344 |
| Urate levels | 1.2E-13 | 0.014926 |  | PMID:34226706 | Barton AR |  | 437354 |
| Red blood cell count | 2E-63 | 0.0333855 |  | PMID:34226706 | Barton AR |  | 445305 |
| Platelet count | 1.8E-91 | 0.0382625 |  | PMID:34226706 | Barton AR |  | 444866 |
| Heel bone mineral density T score | 4E-16 | 0.0158178 |  | PMID:34226706 | Barton AR |  | 445855 |
| Neutrophil count | 4.3E-30 | -0.0234663 |  | PMID:34226706 | Barton AR |  | 443782 |
| Mean corpuscular hemoglobin concentration | 3.8E-08 | -0.0104506 |  | PMID:34226706 | Barton AR |  | 443081 |
| Mean corpuscular volume | 1E-31 | -0.0220919 |  | PMID:34226706 | Barton AR |  | 444035 |
| Apolipoprotein B levels | 7.4E-123 | -0.047789 |  | PMID:34226706 | Barton AR |  | 435744 |
| Bilirubin levels | 9.8E-34 | -0.0221522 |  | PMID:34226706 | Barton AR |  | 436055 |
| Calcium levels | 5.2E-08 | -0.011887 |  | PMID:34226706 | Barton AR |  | 400792 |
| Triglycerides | 5.413E-24 | 0.0484 |  | PMID:20686565 | Teslovich TM |  | 96598 |
| Corrected insulin response | 0.00396 | 0.06 |  | PMID:24699409 | Prokopenko I |  | 5372 |
| Cholesteryl ester_20:5_[M+NH4]1+ levels | 2.6E-147 | -0.306302 |  | PMID:34503513 | Harshfield EL |  | 13814 |
| Phosphatidylserine-P_42:1_[M+H]1+/Phosphatidylglycerol-P_42:6_[M+NH4]1+ levels | 1.2E-19 | -0.119898 |  | PMID:34503513 | Harshfield EL |  | 13814 |
| Phosphatidylcholine_40:8_[M+OAc]1-/Phosphatidylserine_44:7_[M-H]1- levels | 3.3E-216 | 0.336895 |  | PMID:34503513 | Harshfield EL |  | 13814 |
| Fatty acid(20:4)_[M-H]1- levels | 4.8E-121 | -0.187764 |  | PMID:34503513 | Harshfield EL |  | 13814 |
| Phosphatidylcholine-O_36:3_[M+OAc]1-/Phosphatidylcholine-P_36:2_[M+OAc]1-/Phosphatidylserine-O_40:2_[M-H]1- levels | 1.2E-71 | 0.190076 |  | PMID:34503513 | Harshfield EL |  | 13814 |
| Phosphatidylcholine_34:2_[M+OAc]1-/Phosphatidylserine_38:1_[M-H]1- levels | 3.7E-68 | 0.173549 |  | PMID:34503513 | Harshfield EL |  | 13814 |
| Phosphatidylethanolamine_34:2_[M-H]1- levels | 6.7E-71 | 0.213577 |  | PMID:34503513 | Harshfield EL |  | 13814 |
| Phosphatidylinositol-O_36:0_[M-H]1- levels | 3.5E-30 | -0.122608 |  | PMID:34503513 | Harshfield EL |  | 13814 |
| Fatty acid(22:5)_[M-H]1- levels | 8.8E-42 | -0.166356 |  | PMID:34503513 | Harshfield EL |  | 13814 |
| Phosphatidylcholine_40:9_[M+OAc]1-/Phosphatidylserine_44:8_[M-H]1- levels | 2E-31 | -0.115413 |  | PMID:34503513 | Harshfield EL |  | 13814 |
| Fatty acid(18:4)_[M-H]1- levels | 6E-16 | -0.08033 |  | PMID:34503513 | Harshfield EL |  | 13814 |
| Phosphatidylcholine-O_34:2_[M+OAc]1-/Phosphatidylcholine-P_34:1_[M+OAc]1-/Phosphatidylserine-O_38:1_[M-H]1- levels | 8.7E-11 | 0.0718932 |  | PMID:34503513 | Harshfield EL |  | 13814 |
| Phosphatidylinositol_36:0_[M-H]1- levels | 6.9E-19 | -0.0965219 |  | PMID:34503513 | Harshfield EL |  | 13814 |
| Fatty acid(20:3)_[M-H]1- levels | 1.8E-10 | 0.0503661 |  | PMID:34503513 | Harshfield EL |  | 13814 |
| Phosphatidylcholine_32:0_[M+OAc]1- levels | 1.3E-24 | -0.107547 |  | PMID:34503513 | Harshfield EL |  | 13814 |
| Phosphatidylcholine-O_32:1_[M+H]1+/Phosphatidylcholine-P_32:0_[M+H]1+/Phosphatidylethanolamine-O_35:1_[M+H]1+ levels | 1.2E-08 | -0.070694 |  | PMID:34503513 | Harshfield EL |  | 13814 |
| Phosphatidylinositol_35:0_[M-H]1- levels | 2.7E-10 | -0.0513618 |  | PMID:34503513 | Harshfield EL |  | 13814 |
| Phosphatidylcholine_35:5_[M+OAc]1-/Phosphatidylserine_39:4_[M-H]1- levels | 1.3E-24 | -0.100071 |  | PMID:34503513 | Harshfield EL |  | 13814 |
| Phosphatidylserine_38:3_[M+H]1+/Phosphatidylglycerol_38:5_[M+NH4]1+ levels | 0.0036 | 0.0326123 |  | PMID:34503513 | Harshfield EL |  | 13814 |
| Diacylglycerol_33:1_[M+H-H2O]1+ levels | 0.0016 | 0.0373316 |  | PMID:34503513 | Harshfield EL |  | 13814 |
| Phosphatidylserine-O_38:4_[M+H]1+/Phosphatidylglycerol-O_38:6_[M+NH4]1+ levels | 0.000074 | -0.0408088 |  | PMID:34503513 | Harshfield EL |  | 13814 |
| Diacylglycerol_42:7_[M+H-H2O]1+ levels | 0.00044 | -0.0409408 |  | PMID:34503513 | Harshfield EL |  | 13814 |
| Diacylglycerol_34:0_[M+H-H2O]1+ levels | 0.0011 | 0.0392929 |  | PMID:34503513 | Harshfield EL |  | 13814 |
| Phosphatidylethanolamine_42:9_[M+H]1+/Phosphatidate_44:10_[M+NH4]1+ levels | 0.00005 | 0.0602858 |  | PMID:34503513 | Harshfield EL |  | 13814 |
| Lysophosphatidylcholine_20:4_[M+H]1+ levels | 3E-261 | -0.41056 |  | PMID:34503513 | Harshfield EL |  | 13814 |
| Phosphatidylcholine_35:3_[M+H]1+/Phosphatidylethanolamine_38:3_[M+H]1+/Phosphatidate_40:4_[M+NH4]1+ levels | 1.7E-95 | 0.217406 |  | PMID:34503513 | Harshfield EL |  | 13814 |
| Phosphatidylcholine-O_38:5_[M+H]1+/Phosphatidylcholine-P_38:4_[M+H]1+ levels | 8E-54 | -0.230413 |  | PMID:34503513 | Harshfield EL |  | 13814 |
| Phosphatidylcholine-O_18:0_[M+H]1+/Lysophosphatidylethanolamine_21:0_[M+H]1+ levels | 2.1E-08 | -0.0626124 |  | PMID:34503513 | Harshfield EL |  | 13814 |
| Phosphatidylcholine_40:7_[M+H]1+ levels | 1.2E-35 | -0.177847 |  | PMID:34503513 | Harshfield EL |  | 13814 |
| Cholesterol_[M+H-H2O]1+ levels | 4.8E-08 | -0.0502817 |  | PMID:34503513 | Harshfield EL |  | 13814 |
| Phosphatidylcholine-O_33:3_[M+H]1+/Phosphatidylcholine-P_33:2_[M+H]1+/Phosphatidylethanolamine-O_36:3_[M+H]1+ levels | 2.6E-12 | 0.0890567 |  | PMID:34503513 | Harshfield EL |  | 13814 |
| Phosphatidylethanolamine-O_40:6_[M+H]1+/Phosphatidylethanolamine-P_40:5_[M+H]1+ levels | 5.7E-26 | -0.135503 |  | PMID:34503513 | Harshfield EL |  | 13814 |
| Triacylglycerol_50:5_[M+NH4]1+ levels | 4.1E-07 | -0.0772385 |  | PMID:34503513 | Harshfield EL |  | 13814 |
| Phosphatidylcholine-O_40:5_[M+H]1+/Phosphatidylcholine-P_40:4_[M+H]1+ levels | 7.3E-39 | -0.195699 |  | PMID:34503513 | Harshfield EL |  | 13814 |
| Phosphatidylinositol_38:4_[M+H]1+ levels | 5.9E-20 | -0.120204 |  | PMID:34503513 | Harshfield EL |  | 13814 |
| Triacylglycerol_50:0_[M+NH4]1+ levels | 0.0038 | 0.0451787 |  | PMID:34503513 | Harshfield EL |  | 13814 |
| Triacylglycerol_50:3_[M+NH4]1+ levels | 0.0033 | 0.0454155 |  | PMID:34503513 | Harshfield EL |  | 13814 |
| Phosphatidylethanolamine-O_36:5_[M+H]1+/Phosphatidylethanolamine-P_36:4_[M+H]1+ levels | 1E-39 | -0.174087 |  | PMID:34503513 | Harshfield EL |  | 13814 |
| Lymphocyte counts | 2.43E-07 | -0.010274 |  | PMID:32888493 | Chen MH |  | 524923 |
| White blood cell count | 2.8E-31 | -0.022516 |  | PMID:32888493 | Chen MH |  | 562243 |
| Red cell distribution width | 6.32E-171 | -0.055089 |  | PMID:32888493 | Chen MH |  | 531774 |
| Red cell distribution width | 9.2E-158 | -0.0598222 |  | PMID:32888494 | Vuckovic D |  | 408112 |
| Mean corpuscular volume | 6.2E-23 | -0.0213579 |  | PMID:32888494 | Vuckovic D |  | 408112 |
| Single spontaneous delivery | 0.0000544 | -0.03237 | 0.968148301 |  | FINNGEN_R6 | 71075 | 140316 |
| Benign neoplasms | 0.000717 | -0.02472 | 0.975583037 |  | FINNGEN_R6 | 62983 | 260405 |
| Mood [affective] disorders | 0.000587 | 0.03343 | 1.033995062 |  | FINNGEN_R6 | 31588 | 260405 |
| Peripheral atherosclerosis | 0.0000971 | -0.07214 | 0.930400631 |  | FINNGEN_R6 | 8393 | 199229 |
| Symptoms and signs involving the digestive system and abdomen | 0.00205 | 0.02135 | 1.021579542 |  | FINNGEN_R6 | 69391 | 260405 |
| Disorders of lipoprotein metabolism and other lipidaemias | 0.00000105 | -0.05658 | 0.944990882 |  | FINNGEN_R6 | 20148 | 251596 |
| Ulcerative colitis | 0.0000188 | 0.08835 | 1.092370385 |  | FINNGEN_R6 | 5349 | 255054 |
| Colorectal cancer | 0.00427 | -0.06459 | 0.93745174 |  | FINNGEN_R6 | 4401 | 260405 |
| Atopic dermatitis | 0.00207 | 0.0521 | 1.053481085 |  | FINNGEN_R6 | 8383 | 244544 |
| Malignant neoplasm of respiratory system and intrathoracic organs (controls excluding all cancers) | 0.00459 | -0.07172 | 0.930791481 |  | FINNGEN_R6 | 3679 | 207676 |
| Benign neoplasm: Ascending colon | 0.00216 | -0.11248 | 0.893615219 |  | FINNGEN_R6 | 1605 | 260405 |
| Monocyte percentage of white cells | 2.715E-14 | 0.02845103 |  | PMID:27863252 | Astle WJ |  | 170494 |
| High light scatter reticulocyte count | 0.0002679 | 0.01371288 |  | PMID:27863252 | Astle WJ |  | 170761 |
| Eosinophil counts | 9.221E-11 | -0.02423451 |  | PMID:27863252 | Astle WJ |  | 172275 |
| Mean corpuscular volume | 1.208E-10 | -0.02384161 |  | PMID:27863252 | Astle WJ |  | 172433 |
| Ground coffee (include espresso, filter etc) | coffee type | 4.16068E-05 | 0.027092348 | 1.027462682 |  | UKB Neale v2 | 64962 | 283449 |
| Acne | 0.0000765 | 0.14748 | 1.158910106 |  | FINNGEN_R6 | 1558 | 252546 |
| Car/motor vehicle | transport type for commuting to job workplace | 0.000837408 | -0.02827099 | 0.972124899 |  | UKB Neale v2 | 38351 | 190832 |
| Benign neoplasm: Sigmoid colon (controls excluding all cancers) | 0.00255 | -0.08535 | 0.918190861 |  | FINNGEN_R6 | 2695 | 210214 |
| High cholesterol | non-cancer illness code, self-reported | 3.04078E-06 | -0.0344583 | 0.966128629 |  | UKB Neale v2 | 43957 | 361141 |
| No | breathing problems responsible for leaving job | 6.40917E-06 | -0.09396511 | 0.910314526 |  | UKB Neale v2 | 5412 | 91149 |
| Seen a psychiatrist for nerves, anxiety, tension or depression | 1.93608E-05 | 0.033241615 | 1.033800291 |  | UKB Neale v2 | 41233 | 359535 |
| Ever had same-sex intercourse | 0.00107553 | 0.046776143 | 1.047887406 |  | UKB Neale v2 | 11109 | 326849 |
| Ulcerative ileocolitis | 0.000375 | 0.18086 | 1.198247413 |  | FINNGEN_R6 | 803 | 250508 |
| Number of pregnancy terminations | 0.000121813 | 0.0143394 |  |  | UKB Neale v2 |  | 60099 |
| Ecg, heart rate | 2.12584E-05 | 0.387202 |  |  | UKB Neale v2 |  | 53777 |
| Sleep duration | 4.03306E-06 | 0.00874005 |  |  | UKB Neale v2 |  | 359020 |
| Spherical power (right) | 6.72583E-05 | -0.0592621 |  |  | UKB Neale v2 |  | 77983 |
| High light scatter reticulocyte count | 4.77639E-09 | 0.000151164 |  |  | UKB Neale v2 |  | 344729 |
| Falls in the last year | 0.000412808 | -0.00495476 |  |  | UKB Neale v2 |  | 360344 |
| Nitrogen dioxide air pollution; 2006 | 0.00498713 | 0.0602891 |  |  | UKB Neale v2 |  | 356078 |
| Eosinophill count | 5.41232E-20 | -0.0169223 |  |  | UKB Neale v2 |  | 349856 |
| Mean platelet (thrombocyte) volume | 1.56384E-56 | -0.0428248 |  |  | UKB Neale v2 |  | 350470 |
| Place of birth in uk - north co-ordinate | 0.00126613 | -967.969 |  |  | UKB Neale v2 |  | 338701 |
| Heel bone mineral density | 4.8E-13 | 0.0154482 |  | PMID:30598549 | Morris JA |  | 426824 |
| Daytime nap | 7.2E-14 | 0.00951441 |  | PMID:33568662 | Dashti HS |  | 452633 |
| Eprosartan | treatment/medication code | 0.00479981 | 0.277361844 | 1.31964379 |  | UKB Neale v2 | 229 | 361141 |
| Cardiovascular disease | 0.0028 | -0.00287114 | 0.997132978 | PMID:33959723 | Donertas HM | 177923 | 484598 |
| High cholesterol | 3.8E-09 | -0.0038775 | 0.996130008 | PMID:33959723 | Donertas HM | 59853 | 484598 |
| Respiratory or ear-nose-throat disease | 3E-11 | -0.00541455 | 0.994600082 | PMID:33959723 | Donertas HM | 86427 | 484598 |
| Malignant neoplasm, other | 0.0043 | -0.0342 | 0.96637821 |  | UKB SAIGE | 15979 | 386583 |
| Sebaceous cyst | 0.000113 | -0.0623 | 0.939600964 |  | UKB SAIGE | 8876 | 408131 |
| Triglyceride levels | 0.000108187 | 0.06 |  | PMID:28887542 | Prins BP |  | 9745 |
| Other non-epithelial cancer of skin | 0.000627 | -0.0501 | 0.951134306 |  | UKB SAIGE | 11149 | 406220 |
| LDL cholesterol | 0.000311741 | -0.06 |  | PMID:28887542 | Prins BP |  | 9961 |
| Arterial embolism and thrombosis of lower extremity artery | 0.00369 | -0.183 | 0.832768156 |  | UKB SAIGE | 557 | 401152 |
| Sialoadenitis | 0.00283 | -0.232 | 0.792946123 |  | UKB SAIGE | 366 | 403689 |
| Apolipoprotein A-I measurement | 0.0001057 | 0.0539 |  | PMID:33328453 | Pietzner M |  | 10708 |
| Tumor necrosis factor ligand superfamily member 14 levels | 0.003317 | 0.0343 |  | PMID:33067605 | Folkersen L |  | 21758 |
| TNF-related activation-induced cytokine levels | 0.001774 | -0.0354 |  | PMID:33067605 | Folkersen L |  | 21758 |
| Crohn's disease vs rheumatoid arthritis (ordinary least squares (OLS)) | 9.2E-09 | 0.051 | 1.052322893 | PMID:33686288 | Peyrot WJ | 14831 | 14831 |
| Ovarian cancer-related tumor marker CA 125 levels | 0.001579 | 0.0483 |  | PMID:33067605 | Folkersen L |  | 21758 |
| Galanin peptide levels | 0.003903 | 0.0428 |  | PMID:33067605 | Folkersen L |  | 21758 |
| Haemorrhoidal disease | 0.001242 | -0.0134 | 0.98668938 | PMID:33888516 | Zheng T | 218920 | 944133 |
| Birth weight | 0.0036 | 0.0112 |  | PMID:27680694 | Horikoshi M |  | 133903 |
| Gamma glutamyl transferase levels | 0.000039 | 0.0086497 |  | PMID:34226706 | Barton AR |  | 437651 |
| Mean reticulocyte volume | 3.2E-24 | -0.0201767 |  | PMID:34226706 | Barton AR |  | 436944 |
| Cognitive performance | 0.00000331 | 0.01394 |  | PMID:30038396 | Lee JJ |  | 257841 |
| HDL cholesterol levels | 8E-278 | -0.0705865 |  | PMID:34226706 | Barton AR |  | 400754 |
| Serum total protein level | 1.6E-53 | 0.0339053 |  | PMID:34226706 | Barton AR |  | 400482 |
| Cholesterol, total | 2.851E-22 | -0.0506 |  | PMID:20686565 | Teslovich TM |  | 100184 |
| Cholesterol, total | 2.619E-37 | -0.0479 |  | PMID:24097068 | Willer CJ |  | 94595 |
| Fasting blood glucose | 4.615E-10 | -0.02 |  | PMID:22581228 | Manning AK |  | 58074 |
| Lysophosphatidylcholine-O_18:2_[M+H]1+/Lysophosphatidylcholine-P_18:1_[M+H]1+ levels | 4.4E-08 | -0.0539117 |  | PMID:34503513 | Harshfield EL |  | 13814 |
| Ceramide_40:1_[M-H]1- levels | 6.2E-08 | 0.0599294 |  | PMID:34503513 | Harshfield EL |  | 13814 |
| Phosphatidylcholine_35:3_[M+OAc]1-/Phosphatidylserine_39:2_[M-H]1- levels | 6.6E-17 | 0.0746555 |  | PMID:34503513 | Harshfield EL |  | 13814 |
| Phosphatidylethanolamine-O_40:6_[M-H]1-/Phosphatidylethanolamine-P_40:5_[M-H]1- levels | 1.4E-22 | -0.0989972 |  | PMID:34503513 | Harshfield EL |  | 13814 |
| Phosphatidylglycerol_36:0_[M-H]1- levels | 1.9E-17 | -0.0862587 |  | PMID:34503513 | Harshfield EL |  | 13814 |
| Sphingomyelin_34:2_[M+OAc]1- levels | 4.1E-09 | -0.0588196 |  | PMID:34503513 | Harshfield EL |  | 13814 |
| Lysophosphatidylethanolamine-O_18:1_[M-H]1-/Lysophosphatidylethanolamine-P_18:0_[M-H]1- levels | 1.2E-18 | -0.0908632 |  | PMID:34503513 | Harshfield EL |  | 13814 |
| Phosphatidylcholine_38:6_[M+OAc]1-/Phosphatidylserine_42:5_[M-H]1- levels | 2.7E-19 | -0.0980497 |  | PMID:34503513 | Harshfield EL |  | 13814 |
| Lysophosphatidylcholine_20:3_[M+OAc]1- levels | 1.5E-12 | 0.0717838 |  | PMID:34503513 | Harshfield EL |  | 13814 |
| Phosphatidylethanolamine-O_40:4_[M-H]1-/Phosphatidylethanolamine-P_40:3_[M-H]1- levels | 0.0000017 | -0.0451539 |  | PMID:34503513 | Harshfield EL |  | 13814 |
| Phosphatidylinositol_35:2_[M-H]1- levels | 5.5E-12 | 0.0729059 |  | PMID:34503513 | Harshfield EL |  | 13814 |
| Diacylglycerol_44:6_[M+H-H2O]1+ levels | 7.1E-09 | -0.0706272 |  | PMID:34503513 | Harshfield EL |  | 13814 |
| Phosphatidylcholine-O_31:0_[M+H]1+/Phosphatidylethanolamine-O_34:0_[M+H]1+ levels | 0.0000017 | -0.0578009 |  | PMID:34503513 | Harshfield EL |  | 13814 |
| Diacylglycerol_38:3_[M+H-H2O]1+ levels | 9.3E-99 | 0.174701 |  | PMID:34503513 | Harshfield EL |  | 13814 |
| Phosphatidylcholine-2O_32:0_[M+H]1+ levels | 0.000043 | -0.0509465 |  | PMID:34503513 | Harshfield EL |  | 13814 |
| Phosphatidylcholine_32:0_[M+H]1+/Phosphatidylethanolamine_35:0_[M+H]1+/Phosphatidate_37:1_[M+NH4]1+ levels | 3.1E-40 | -0.160491 |  | PMID:34503513 | Harshfield EL |  | 13814 |
| Lysophosphatidylethanolamine_18:2_[M+H]1+ levels | 8E-39 | 0.160014 |  | PMID:34503513 | Harshfield EL |  | 13814 |
| Lysophosphatidylcholine-O_16:1_[M+H]1+/Lysophosphatidylcholine-P_16:0_[M+H]1+ levels | 6.7E-25 | -0.0923873 |  | PMID:34503513 | Harshfield EL |  | 13814 |
| Phosphatidylcholine-O_37:2_[M+H]1+/Phosphatidylcholine-P_37:1_[M+H]1+/Phosphatidylethanolamine-O_40:2_[M+H]1+ levels | 0.00016 | -0.0513867 |  | PMID:34503513 | Harshfield EL |  | 13814 |
| Phosphatidylcholine_37:5_[M+H]1+/Phosphatidylethanolamine_40:5_[M+H]1+/Phosphatidate_42:6_[M+NH4]1+ levels | 1.1E-34 | -0.185174 |  | PMID:34503513 | Harshfield EL |  | 13814 |
| Phosphatidylcholine_40:6_[M+H]1+/Phosphatidylethanolamine_43:6_[M+H]1+ levels | 3.3E-29 | -0.174014 |  | PMID:34503513 | Harshfield EL |  | 13814 |
| Phosphatidylcholine-P_38:6_[M+H]1+ levels | 4.3E-09 | -0.0764781 |  | PMID:34503513 | Harshfield EL |  | 13814 |
| Diacylglycerol_36:3_[M+NH4]1+ levels | 8E-10 | 0.0672855 |  | PMID:34503513 | Harshfield EL |  | 13814 |
| Sphingomyelin_40:2_[M+H]1+ levels | 0.000055 | -0.0578681 |  | PMID:34503513 | Harshfield EL |  | 13814 |
| Sphingomyelin_41:2_[M+H]1+ levels | 0.00013 | -0.0550847 |  | PMID:34503513 | Harshfield EL |  | 13814 |
| Pulse pressure | 0.000009548 | -0.00732229 |  | PMID:33230300 | Surendran P |  | 810865 |
| Type 2 diabetes (adjusted for BMI) | 0.00002507 | -0.0302 | 0.970251464 | PMID:29632382 | Mahajan A | 48286 | 298957 |
| Reticulocyte fraction of red cells | 4.7E-09 | 0.0132442 |  | PMID:32888494 | Vuckovic D |  | 408112 |
| Neutrophil percentage of white cells | 1.4E-07 | -0.0119497 |  | PMID:32888494 | Vuckovic D |  | 408112 |
| Hemoglobin | 1.1E-40 | 0.0299385 |  | PMID:32888494 | Vuckovic D |  | 408112 |
| Acute appendicitis | 0.00312 | 0.03343 | 1.033995062 |  | FINNGEN_R6 | 18798 | 258873 |
| Delivery | 0.000052 | -0.03167 | 0.968826242 |  | FINNGEN_R6 | 77820 | 147061 |
| Chronic lower respiratory diseases | 0.00477 | -0.02472 | 0.975583037 |  | FINNGEN_R6 | 39947 | 260405 |
| Non-rheumatic valve diseases | 0.00145 | -0.04704 | 0.954049235 |  | FINNGEN_R6 | 12375 | 195346 |
| Personality disorders (more control exclusions) | 0.000232 | 0.07969 | 1.082951301 |  | FINNGEN_R6 | 7121 | 205222 |
| Specific personality disorders | 0.00279 | 0.05979 | 1.061613584 |  | FINNGEN_R6 | 6142 | 259019 |
| DVT of lower extremities | 0.0000425 | -0.0831 | 0.920259117 |  | FINNGEN_R6 | 5632 | 231367 |
| Type 2 diabetes with coma | 0.0046 | -0.07886 | 0.924169299 |  | FINNGEN_R6 | 3011 | 218171 |
| Emotionally unstable personality disorder | 0.00211 | 0.08647 | 1.090318658 |  | FINNGEN_R6 | 2975 | 255852 |
| Acne vulgaris | 0.000204 | 0.15051 | 1.162426929 |  | FINNGEN_R6 | 1315 | 252303 |
| Talking therapies, such as psychotherapy, counselling, group therapy or cbt | activities undertaken to treat depression | 0.000729067 | 0.034731558 | 1.035341743 |  | UKB Neale v2 | 25960 | 117763 |
| Basophil percentage of white cells | 0.00151 | 0.01155656 |  | PMID:27863252 | Astle WJ |  | 171996 |
| Neutrophil percentage of white cells | 0.0001976 | -0.01393076 |  | PMID:27863252 | Astle WJ |  | 171542 |
| Neutrophil count | 3.107E-10 | -0.02364545 |  | PMID:27863252 | Astle WJ |  | 170702 |
| Basophil percentage of granulocytes | 0.00004375 | 0.01501741 |  | PMID:27863252 | Astle WJ |  | 170223 |
| Eosinophil percentage of granulocytes | 0.0006011 | -0.01287851 |  | PMID:27863252 | Astle WJ |  | 170536 |
| White blood cell count | 6.285E-10 | -0.02324855 |  | PMID:27863252 | Astle WJ |  | 172435 |
| Granulocyte count | 4.364E-12 | -0.02608207 |  | PMID:27863252 | Astle WJ |  | 169822 |
| Public transport | transport type for commuting to job workplace | 0.0028627 | 0.024936657 | 1.025250176 |  | UKB Neale v2 | 39655 | 190832 |
| Yes | breathing problems during period of job | 4.89242E-05 | -0.07656107 | 0.926296345 |  | UKB Neale v2 | 6697 | 91149 |
| Cholesterol lowering medication | medication for cholesterol, blood pressure, diabetes, or take exogenous hormones | 5.82968E-05 | -0.03976366 | 0.961016536 |  | UKB Neale v2 | 24247 | 193148 |
| Benign neoplasm: Sigmoid colon | 0.0031 | -0.08319 | 0.920176297 |  | FINNGEN_R6 | 2695 | 260405 |
| Heel bone mineral density | 7.7E-08 | 0.0185461 |  | PMID:28869591 | Kemp JP |  | 142487 |
| Mean corpuscular haemoglobin | 0.00345638 | -0.0133065 |  |  | UKB Neale v2 |  | 350472 |
| Heel bone mineral density (bmd) t-score, automated | 7.22471E-05 | 0.0153253 |  |  | UKB Neale v2 |  | 206589 |
| Pulse rate | 7.51779E-14 | 0.370835 |  |  | UKB Neale v2 |  | 118850 |
| Nap during day | 1.89412E-10 | 0.00917836 |  |  | UKB Neale v2 |  | 360855 |
| Length of mobile phone use | 0.00415809 | -0.00947683 |  |  | UKB Neale v2 |  | 356618 |
| Recent easy annoyance or irritability | 0.000565428 | 0.00872668 |  |  | UKB Neale v2 |  | 117483 |
| Astigmatism angle (left) | 0.0034527 | 0.790747 |  |  | UKB Neale v2 |  | 77739 |
| Lifetime number of sexual partners | 0.00127523 | 0.00680017 |  |  | UKB Neale v2 |  | 296609 |
| Mean corpuscular volume | 2.27971E-13 | -0.0800813 |  |  | UKB Neale v2 |  | 350473 |
| Appendicular lean mass | 0.0000034 | -0.0117608 |  | PMID:33097823 | Pei YF |  | 244730 |
| Fractures | 0.000099 | 4.980154587 | 145.4968718 | PMID:30598549 | Morris JA | 53184 | 426795 |
| Sex hormone-binding globulin levels | 7.6E-26 | -0.0102326 |  | PMID:32042192 | Ruth KS |  | 370125 |
| Sex hormone-binding globulin levels adjusted for BMI | 6.8E-28 | -0.00953896 |  | PMID:32042192 | Ruth KS |  | 368929 |
| Sex hormone-binding globulin levels adjusted for BMI | 3E-20 | -0.0120791 |  | PMID:32042192 | Ruth KS |  | 188908 |
| Circulatory disease nec | 0.00249 | -0.0366 | 0.964061683 |  | UKB SAIGE | 16366 | 404271 |
| Skin cancer | 0.00132 | -0.0424 | 0.958486309 |  | UKB SAIGE | 13752 | 408823 |
| Inguinal hernia | 0.000753 | -0.0424 | 0.958486309 |  | UKB SAIGE | 15995 | 377612 |
| Asthma | 8E-16 | -0.00542137 | 0.994593299 | PMID:33959723 | Donertas HM | 56087 | 484598 |
| Oesophageal cancer | cancer code, self-reported | 0.0027409 | -0.3501852 | 0.704557595 |  | UKB Neale v2 | 162 | 361141 |
| Anal and rectal polyp | 0.00182 | -0.0547 | 0.946769136 |  | UKB SAIGE | 7408 | 394746 |
| Scavenger receptor cysteine-rich domain-containing group B protein levels | 0.000131987 | -0.1818996 |  | PMID:33303764 | Gilly A |  | 1272 |
| Ankylosing spondylitis | 0.00439 | -0.171 | 0.842821573 |  | UKB SAIGE | 620 | 365705 |
| Fibrinogen gamma chain measurement | 0.003707 | 0.0408 |  | PMID:33328453 | Pietzner M |  | 10708 |
| Phospholipase D3 measurement | 0.0000819 | 0.0528 |  | PMID:33328453 | Pietzner M |  | 10708 |
| KDEL motif-containing protein 2 measurement | 0.00004712 | 0.0549 |  | PMID:33328453 | Pietzner M |  | 10708 |
| Bipolar disorder | 0.0006632 | 0.0795 | 1.08274556 | PMID:27329760 | Hou L | 7647 | 34950 |
| Ulcerative colitis vs rheumatoid arthritis (ordinary least squares (OLS)) | 0.00058 | 0.029 | 1.029424594 | PMID:33686288 | Peyrot WJ | 15843 | 15843 |
| Bipolar disorder vs major depressive disorder (ordinary least squares (OLS)) | 0.000093 | 0.011 | 1.011060722 | PMID:33686288 | Peyrot WJ | 191108 | 191108 |
| White blood cell count | 3.7E-27 | -0.0224204 |  | PMID:34226706 | Barton AR |  | 444734 |
| Creatinine levels | 2.5E-07 | -0.0113465 |  | PMID:34226706 | Barton AR |  | 437660 |
| High light scatter reticulocyte count | 7.4E-12 | 0.0131564 |  | PMID:34226706 | Barton AR |  | 437723 |
| Aspartate aminotransferase levels | 0.0024 | 0.00617044 |  | PMID:34226706 | Barton AR |  | 436275 |
| Apolipoprotein A1 levels | 1.6E-97 | -0.0436536 |  | PMID:34226706 | Barton AR |  | 398508 |
| Frailty index | 0.00006907 | -0.0138 |  | PMID:34431594 | Atkins JL |  | 175226 |
| Lipoprotein (a) levels | 0.0025 | 0.00379739 |  | PMID:34226706 | Barton AR |  | 348806 |
| Circulating plasma alpha-Klotho levels | 0.0043 | 0.065 |  | PMID:34542150 | Gergei I |  | 4376 |
| Ribonate levels | 0.002725 | 0.0354 |  | PMID:33437055 | Panyard DJ |  | 291 |
| LDL cholesterol | 1.762E-21 | -0.05 |  | PMID:20686565 | Teslovich TM |  | 95454 |
| Phosphatidylcholine_35:2_[M+H]1+/Phosphatidylethanolamine_38:2_[M+H]1+/Phosphatidate_40:3_[M+NH4]1+ levels | 2.1E-40 | 0.16424 |  | PMID:34503513 | Harshfield EL |  | 13814 |
| Phosphatidylcholine_37:4_[M+H]1+/Phosphatidylethanolamine_40:4_[M+H]1+/Phosphatidate_42:5_[M+NH4]1+ levels | 2.3E-130 | -0.376112 |  | PMID:34503513 | Harshfield EL |  | 13814 |
| Phosphatidylcholine_38:8_[M+OAc]1-/Phosphatidylserine_42:7_[M-H]1- levels | 1.7E-81 | 0.206448 |  | PMID:34503513 | Harshfield EL |  | 13814 |
| Lysophosphatidylethanolamine_20:4_[M-H]1- levels | 2.5E-69 | -0.206053 |  | PMID:34503513 | Harshfield EL |  | 13814 |
| Phosphatidylinositol_36:3_[M-H]1- levels | 1.4E-93 | 0.22292 |  | PMID:34503513 | Harshfield EL |  | 13814 |
| Phosphatidylethanolamine_39:4_[M-H]1- levels | 1.3E-80 | -0.187081 |  | PMID:34503513 | Harshfield EL |  | 13814 |
| Phosphatidylinositol_34:2_[M-H]1- levels | 1.2E-29 | 0.125716 |  | PMID:34503513 | Harshfield EL |  | 13814 |
| Phosphatidylcholine_35:4_[M+OAc]1-/Phosphatidylserine_39:3_[M-H]1- levels | 1.6E-67 | -0.154956 |  | PMID:34503513 | Harshfield EL |  | 13814 |
| Phosphatidylinositol_36:4_[M-H]1- levels | 2E-22 | -0.105799 |  | PMID:34503513 | Harshfield EL |  | 13814 |
| Phosphatidylethanolamine-O_38:6_[M-H]1-/Phosphatidylethanolamine-P_38:5_[M-H]1- levels | 5.1E-23 | -0.114666 |  | PMID:34503513 | Harshfield EL |  | 13814 |
| Phosphatidylcholine-O_38:4_[M+OAc]1-/Phosphatidylcholine-P_38:3_[M+OAc]1-/Phosphatidylserine-P_42:0_[M-H]1- levels | 1.3E-14 | -0.079379 |  | PMID:34503513 | Harshfield EL |  | 13814 |
| Phosphatidylcholine_52:4_[M+OAc]1- levels | 2.9E-22 | -0.0971759 |  | PMID:34503513 | Harshfield EL |  | 13814 |
| Sphingomyelin_36:2_[M+OAc]1- levels | 9.5E-12 | -0.0706308 |  | PMID:34503513 | Harshfield EL |  | 13814 |
| Ceramide_42:1_[M+OAc]1- levels | 4E-07 | 0.0554539 |  | PMID:34503513 | Harshfield EL |  | 13814 |
| Lysophosphatidylethanolamine_18:1_[M-H]1- levels | 7.5E-07 | 0.0555305 |  | PMID:34503513 | Harshfield EL |  | 13814 |
| Phosphatidylglycerol_36:1_[M-H]1- levels | 0.0049 | -0.0315298 |  | PMID:34503513 | Harshfield EL |  | 13814 |
| Phosphatidylinositol_38:1_[M-H]1- levels | 0.0018 | 0.0360314 |  | PMID:34503513 | Harshfield EL |  | 13814 |
| Triacylglycerol_56:8_[M+NH4]1+ levels | 2.1E-27 | -0.16823 |  | PMID:34503513 | Harshfield EL |  | 13814 |
| Phosphatidylinositol_38:4_[M+NH4]1+ levels | 6.3E-55 | -0.190599 |  | PMID:34503513 | Harshfield EL |  | 13814 |
| Phosphatidylcholine_35:4_[M+H]1+/Phosphatidylethanolamine_38:4_[M+H]1+/Phosphatidate_40:5_[M+NH4]1+ levels | 6.7E-77 | -0.223853 |  | PMID:34503513 | Harshfield EL |  | 13814 |
| Phosphatidylcholine-O_36:4_[M+H]1+/Phosphatidylcholine-P_36:3_[M+H]1+ levels | 3.7E-48 | -0.184008 |  | PMID:34503513 | Harshfield EL |  | 13814 |
| Phosphatidylinositol_36:2_[M+H]1+ levels | 5.4E-15 | 0.105629 |  | PMID:34503513 | Harshfield EL |  | 13814 |
| Cholesteryl ester_22:5_[M+NH4]1+ levels | 2E-13 | -0.0786722 |  | PMID:34503513 | Harshfield EL |  | 13814 |
| Phosphatidylcholine_31:2_[M+H]1+/Phosphatidylethanolamine_34:2_[M+H]1+/Phosphatidate_36:3_[M+NH4]1+ levels | 6.4E-76 | 0.233054 |  | PMID:34503513 | Harshfield EL |  | 13814 |
| Lysophosphatidylcholine_22:6_[M+H]1+ levels | 5.2E-18 | -0.103596 |  | PMID:34503513 | Harshfield EL |  | 13814 |
| Sphingomyelin_34:0_[M+H]1+ levels | 0.00039 | -0.0429829 |  | PMID:34503513 | Harshfield EL |  | 13814 |
| Diacylglycerol_34:2_[M+H-H2O]1+ levels | 2.3E-09 | 0.0652214 |  | PMID:34503513 | Harshfield EL |  | 13814 |
| Phosphatidylcholine_38:3_[M+H]1+/Phosphatidylethanolamine_41:3_[M+H]1+/Phosphatidate_43:4_[M+NH4]1+ levels | 1.3E-61 | 0.259393 |  | PMID:34503513 | Harshfield EL |  | 13814 |
| Phosphatidylcholine_40:4_[M+H]1+/Phosphatidylethanolamine_43:4_[M+H]1+ levels | 1.4E-51 | -0.224185 |  | PMID:34503513 | Harshfield EL |  | 13814 |
| Sphingomyelin_42:3_[M+H]1+ levels | 0.0000021 | -0.0682522 |  | PMID:34503513 | Harshfield EL |  | 13814 |
| Triacylglycerol_51:3_[M+NH4]1+ levels | 0.00014 | 0.0576785 |  | PMID:34503513 | Harshfield EL |  | 13814 |
| Triacylglycerol_53:2_[M+NH4]1+ levels | 0.0000082 | 0.0681385 |  | PMID:34503513 | Harshfield EL |  | 13814 |
| Phosphatidylcholine-O_44:5_[M+H]1+/Phosphatidylcholine-P_44:4_[M+H]1+ levels | 1.4E-31 | -0.180151 |  | PMID:34503513 | Harshfield EL |  | 13814 |
| Triacylglycerol_54:2_[M+NH4]1+ levels | 0.0018 | 0.0485057 |  | PMID:34503513 | Harshfield EL |  | 13814 |
| Phosphatidylcholine-O_34:2_[M+H]1+/Phosphatidylcholine-P_34:1_[M+H]1+/Phosphatidylethanolamine-O_37:2_[M+H]1+ levels | 7.7E-07 | 0.0625801 |  | PMID:34503513 | Harshfield EL |  | 13814 |
| Phosphatidylcholine_37:6_[M+H]1+/Phosphatidylethanolamine_40:6_[M+H]1+/Phosphatidate_42:7_[M+NH4]1+ levels | 1.6E-07 | -0.0791895 |  | PMID:34503513 | Harshfield EL |  | 13814 |
| Type 2 diabetes | 0.000001778 | -0.0366 | 0.964061683 | PMID:29632382 | Mahajan A | 48286 | 298957 |
| Mean platelet volume | 4.7E-84 | -0.0418079 |  | PMID:32888494 | Vuckovic D |  | 408112 |
| Reticulocyte count | 1.3E-18 | 0.0198701 |  | PMID:32888494 | Vuckovic D |  | 408112 |
| Platelet count | 2.6E-63 | 0.0371827 |  | PMID:32888494 | Vuckovic D |  | 408112 |
| Platelet distribution width | 2.8E-19 | 0.0203219 |  | PMID:32888494 | Vuckovic D |  | 408112 |
| White blood cell count | 5.3E-26 | -0.0235638 |  | PMID:32888494 | Vuckovic D |  | 408112 |
| Cholelithiasis | 2.05E-10 | 0.07096 | 1.073538283 |  | FINNGEN_R6 | 23089 | 254733 |
| Mood disorders | 0.000627 | 0.03327 | 1.033829636 |  | FINNGEN_R6 | 31552 | 260405 |
| Metabolic disorders | 0.000115 | -0.03733 | 0.963358175 |  | FINNGEN_R6 | 28957 | 260405 |
| Noninfective enteritis and colitis | 0.0000295 | 0.06194 | 1.063898509 |  | FINNGEN_R6 | 10700 | 260405 |
| Peripheral artery disease | 0.00000179 | -0.07947 | 0.923605728 |  | FINNGEN_R6 | 9021 | 253928 |
| Benign neoplasm: Colon (controls excluding all cancers) | 4.39E-07 | -0.0818 | 0.921456232 |  | FINNGEN_R6 | 9208 | 212634 |
| Diabetes mellitus | 0.00017 | -0.03448 | 0.966107662 |  | FINNGEN_R6 | 45245 | 260405 |
| Bipolar affective disorders | 0.0000158 | 0.09851 | 1.10352544 |  | FINNGEN_R6 | 5091 | 233908 |
| Granulocyte percentage of myeloid white cells | 5.033E-16 | -0.03039748 |  | PMID:27863252 | Astle WJ |  | 169545 |
| None of the above | vitamin and mineral supplements | 0.00309509 | 0.015753249 | 1.015877986 |  | UKB Neale v2 | 112464 | 359245 |
| Reticulocyte count | 9.645E-09 | 0.02162245 |  | PMID:27863252 | Astle WJ |  | 170641 |
| Reticulocyte fraction of red cells | 0.000138 | 0.01435848 |  | PMID:27863252 | Astle WJ |  | 170690 |
| Pattern 1 | hair/balding pattern | 3.29528E-13 | 0.056719614 | 1.05835902 |  | UKB Neale v2 | 53076 | 165649 |
| Pattern 4 | hair/balding pattern | 1.17564E-08 | -0.05373767 | 0.947680681 |  | UKB Neale v2 | 30225 | 165649 |
| Sequelae of injuries of head | 0.00306 | -0.14464 | 0.865333757 |  | FINNGEN_R6 | 880 | 256536 |
| Basal cell carcinoma | cancer code, self-reported | 0.00338989 | -0.07460909 | 0.928106224 |  | UKB Neale v2 | 3441 | 361141 |
| Comparative height size at age 10 | 0.00190306 | -0.00523437 |  |  | UKB Neale v2 |  | 355331 |
| Normal-pressure hydrocephalus | 0.00319 | 0.21707 | 1.242431069 |  | FINNGEN_R6 | 384 | 259903 |
| Number of self-reported non-cancer illnesses | 0.000353393 | -0.00707074 |  |  | UKB Neale v2 |  | 361141 |
| Standing height | 6.27944E-12 | -0.107484 |  |  | UKB Neale v2 |  | 360388 |
| High light scatter reticulocyte percentage | 0.00148301 | 0.00272441 |  |  | UKB Neale v2 |  | 344729 |
| Heel broadband ultrasound attenuation (right) | 0.000214099 | 0.299629 |  |  | UKB Neale v2 |  | 114609 |
| Number of diet questionnaires completed | 0.00178935 | 0.0138636 |  |  | UKB Neale v2 |  | 156401 |
| Home location - north co-ordinate (rounded) | 0.00237296 | -981.603 |  |  | UKB Neale v2 |  | 361187 |
| Secondary malignant neoplasm of other and unspecified sites | 0.00114 | -0.30954 | 0.733784419 |  | FINNGEN_R6 | 230 | 260405 |
| Estradiol levels | 0.000028 | -0.00367644 |  | PMID:32042192 | Ruth KS |  | 206927 |
| Hypothyroidism | 0.0000049 | -0.0592 | 0.942518247 |  | UKB SAIGE | 14871 | 406300 |
| Diabetes or endocrine disease | 1.2E-10 | -0.0041431 | 0.995865471 | PMID:33959723 | Donertas HM | 51949 | 484598 |
| Venous thromboembolism | 0.000025 | -0.00138868 | 0.998612284 | PMID:33959723 | Donertas HM | 12240 | 484598 |
| Diseases of sebaceous glands | 0.000129 | -0.0615 | 0.940352946 |  | UKB SAIGE | 8948 | 408203 |
| Irritable mood | 3.576E-08 | 0.013629483 |  | PMID:29500382 | Nagel M |  | 366726 |
| Cholesterol, total | 0.002152814 | -0.05 |  | PMID:28887542 | Prins BP |  | 9817 |
| Inflammation of eyelids | 0.000527 | -0.105 | 0.900324523 |  | UKB SAIGE | 2396 | 401702 |
| Pancreas fat | 0.0015 | -0.0282722 |  | PMID:34128465 | Liu Y |  | 25617 |
| Atopic dermatitis | 0.0009338 | 0.0344 | 1.034998523 | PMID:34454985 | Sliz E | 22474 | 796661 |
| Pathologic fracture | 0.000306 | -0.237 | 0.788991288 |  | UKB SAIGE | 514 | 401793 |
| Protein FAM162A measurement | 0.001043 | 0.0433 |  | PMID:33328453 | Pietzner M |  | 10708 |
| GrpE protein homolog 1, mitochondrial measurement | 0.0007439 | 0.0451 |  | PMID:33328453 | Pietzner M |  | 10708 |
| Panniculitis | 0.00342 | 0.431 | 1.53879555 |  | UKB SAIGE | 103 | 378814 |
| Mean platelet volume | 4.5E-143 | -0.043928 |  | PMID:34226706 | Barton AR |  | 445364 |
| Height | 6E-18 | -0.0137667 |  | PMID:34226706 | Barton AR |  | 458235 |
| Monocyte count | 0.00011 | 0.00706914 |  | PMID:34226706 | Barton AR |  | 443529 |
| Triglyceride levels | 3.3E-148 | 0.0520023 |  | PMID:34226706 | Barton AR |  | 437532 |
| Eosinophil counts | 3E-32 | -0.0247483 |  | PMID:34226706 | Barton AR |  | 440275 |
| Direct low density lipoprotein cholesterol levels | 9.9E-59 | -0.0338932 |  | PMID:34226706 | Barton AR |  | 437068 |
| Blood glucose levels | 1.7E-20 | -0.0210616 |  | PMID:34226706 | Barton AR |  | 400458 |
| Beta-citrylglutamate levels | 0.002119 | 0.0377 |  | PMID:33437055 | Panyard DJ |  | 291 |
| Asthma | 0.0000106 | -0.05909597 | 0.942616302 | PMID:29273806 | Demenais F | 19954 | 127669 |
| HDL cholesterol | 2.622E-22 | -0.0476 |  | PMID:20686565 | Teslovich TM |  | 99900 |
| Phosphatidylcholine_36:4_[M+OAc]1-/Phosphatidylserine_40:3_[M-H]1- levels | 3.4E-241 | -0.338647 |  | PMID:34503513 | Harshfield EL |  | 13814 |
| Phosphatidylcholine_38:4_[M+OAc]1-/Phosphatidylserine_42:3_[M-H]1- levels | 1.7E-263 | -0.343882 |  | PMID:34503513 | Harshfield EL |  | 13814 |
| Sphingomyelin_38:2_[M+H]1+ levels | 1E-10 | -0.0736886 |  | PMID:34503513 | Harshfield EL |  | 13814 |
| Lysophosphatidylethanolamine_22:4_[M-H]1- levels | 4.9E-284 | -0.352894 |  | PMID:34503513 | Harshfield EL |  | 13814 |
| Phosphatidylethanolamine_38:4_[M-H]1- levels | 2.4E-148 | -0.224436 |  | PMID:34503513 | Harshfield EL |  | 13814 |
| Phosphatidylcholine-O_36:5_[M+OAc]1-/Phosphatidylcholine-P_36:4_[M+OAc]1-/Phosphatidylserine-O_40:4_[M-H]1- levels | 2.4E-131 | -0.26691 |  | PMID:34503513 | Harshfield EL |  | 13814 |
| Phosphatidylethanolamine-O_38:6_[M+H]1+/Phosphatidylethanolamine-P_38:5_[M+H]1+ levels | 4.6E-20 | -0.111992 |  | PMID:34503513 | Harshfield EL |  | 13814 |
| Phosphatidylethanolamine-O_34:3_[M-H]1-/Phosphatidylethanolamine-P_34:2_[M-H]1- levels | 2.2E-32 | 0.139659 |  | PMID:34503513 | Harshfield EL |  | 13814 |
| Phosphatidylethanolamine_37:3_[M-H]1- levels | 5.2E-20 | 0.0899816 |  | PMID:34503513 | Harshfield EL |  | 13814 |
| Phosphatidylcholine_34:4_[M+OAc]1-/Phosphatidylserine_38:3_[M-H]1- levels | 2.2E-32 | -0.132109 |  | PMID:34503513 | Harshfield EL |  | 13814 |
| Phosphatidylcholine-O_42:6_[M+OAc]1-/Phosphatidylcholine-P_42:5_[M+OAc]1- levels | 5.7E-31 | -0.125586 |  | PMID:34503513 | Harshfield EL |  | 13814 |
| Phosphatidylcholine_42:4_[M+OAc]1- levels | 2E-50 | -0.154252 |  | PMID:34503513 | Harshfield EL |  | 13814 |
| Phosphatidylcholine_30:0_[M+H]1+/Phosphatidylethanolamine_33:0_[M+H]1+/Phosphatidate_35:1_[M+NH4]1+ levels | 8.1E-07 | -0.0619632 |  | PMID:34503513 | Harshfield EL |  | 13814 |
| Phosphatidylcholine_38:9_[M+OAc]1-/Phosphatidylserine_42:8_[M-H]1- levels | 9E-22 | -0.103695 |  | PMID:34503513 | Harshfield EL |  | 13814 |
| Phosphatidylinositol_38:0_[M-H]1- levels | 9E-19 | -0.100628 |  | PMID:34503513 | Harshfield EL |  | 13814 |
| Phosphatidylglycerol_34:0_[M-H]1- levels | 4.2E-22 | -0.111613 |  | PMID:34503513 | Harshfield EL |  | 13814 |
| Ceramide_42:2_[M-H]1- levels | 0.000042 | 0.0422376 |  | PMID:34503513 | Harshfield EL |  | 13814 |
| Phosphatidate_36:2_[M-H]1- levels | 4.2E-07 | 0.0628881 |  | PMID:34503513 | Harshfield EL |  | 13814 |
| Sphingomyelin_42:3_[M+OAc]1- levels | 0.0000097 | -0.0417114 |  | PMID:34503513 | Harshfield EL |  | 13814 |
| Lysophosphatidylethanolamine-P_20:0_[M-H]1- levels | 3E-10 | -0.0608044 |  | PMID:34503513 | Harshfield EL |  | 13814 |
| Fatty acid(22:6)_[M-H]1- levels | 2.8E-12 | -0.0845371 |  | PMID:34503513 | Harshfield EL |  | 13814 |
| Phosphatidylinositol_34:1_[M-H]1- levels | 7.5E-08 | 0.0572073 |  | PMID:34503513 | Harshfield EL |  | 13814 |
| Sphingomyelin_40:2_[M+OAc]1- levels | 0.0000036 | -0.0429961 |  | PMID:34503513 | Harshfield EL |  | 13814 |
| Phosphatidylcholine-O_34:3_[M+OAc]1-/Phosphatidylcholine-P_34:2_[M+OAc]1-/Phosphatidylserine-O_38:2_[M-H]1- levels | 0.00098 | 0.0359505 |  | PMID:34503513 | Harshfield EL |  | 13814 |
| Phosphatidylcholine-O_38:2_[M+H]1+/Phosphatidylcholine-P_38:1_[M+H]1+ levels | 0.00016 | -0.0538591 |  | PMID:34503513 | Harshfield EL |  | 13814 |
| Phosphatidylserine_40:5_[M+H]1+/Phosphatidylglycerol_40:7_[M+NH4]1+ levels | 1.6E-41 | -0.176342 |  | PMID:34503513 | Harshfield EL |  | 13814 |
| Phosphatidylethanolamine-P_38:6_[M+H]1+ levels | 0.00022 | -0.0475004 |  | PMID:34503513 | Harshfield EL |  | 13814 |
| Cholesteryl ester_20:3_[M+NH4]1+ levels | 1.1E-33 | -0.126139 |  | PMID:34503513 | Harshfield EL |  | 13814 |
| Phosphatidylcholine_34:3_[M+H]1+/Phosphatidylethanolamine_37:3_[M+H]1+/Phosphatidate_39:4_[M+NH4]1+ levels | 3.6E-47 | 0.174799 |  | PMID:34503513 | Harshfield EL |  | 13814 |
| Diacylglycerol_34:3_[M+H-H2O]1+ levels | 0.00008 | 0.0440123 |  | PMID:34503513 | Harshfield EL |  | 13814 |
| Sphingomyelin_34:2_[M+H]1+ levels | 4.6E-16 | -0.0934677 |  | PMID:34503513 | Harshfield EL |  | 13814 |
| Sphingomyelin_36:2_[M+H]1+ levels | 5.7E-17 | -0.0996437 |  | PMID:34503513 | Harshfield EL |  | 13814 |
| Diacylglycerol_36:3_[M+H-H2O]1+ levels | 3.2E-11 | 0.0738178 |  | PMID:34503513 | Harshfield EL |  | 13814 |
| Phosphatidylcholine_40:5_[M+H]1+ levels | 1.9E-69 | -0.272744 |  | PMID:34503513 | Harshfield EL |  | 13814 |
| Phosphatidylcholine_36:3_[M+H]1+/Phosphatidylethanolamine_39:3_[M+H]1+/Phosphatidate_41:4_[M+NH4]1+ levels | 2E-113 | 0.336511 |  | PMID:34503513 | Harshfield EL |  | 13814 |
| Lysophosphatidylcholine_16:1_[M+H]1+/Lysophosphatidylethanolamine_19:1_[M+H]1+ levels | 0.000092 | 0.0446004 |  | PMID:34503513 | Harshfield EL |  | 13814 |
| Phosphatidylcholine_32:1_[M+H]1+/Phosphatidylethanolamine_35:1_[M+H]1+/Phosphatidate_37:2_[M+NH4]1+ levels | 0.0026 | 0.0385103 |  | PMID:34503513 | Harshfield EL |  | 13814 |
| Diacylglycerol_32:2_[M+H-H2O]1+ levels | 0.0039 | 0.0342939 |  | PMID:34503513 | Harshfield EL |  | 13814 |
| Phosphatidylcholine_32:2_[M+H]1+/Phosphatidylethanolamine_35:2_[M+H]1+/Phosphatidate_37:3_[M+NH4]1+ levels | 2.8E-36 | 0.158709 |  | PMID:34503513 | Harshfield EL |  | 13814 |
| Cholesteryl ester_16:0_[M+NH4]1+ levels | 5.1E-25 | -0.100288 |  | PMID:34503513 | Harshfield EL |  | 13814 |
| Phosphatidylcholine_36:5_[M+H]1+/Phosphatidylethanolamine_39:5_[M+H]1+/Phosphatidate_41:6_[M+NH4]1+ levels | 2.2E-17 | -0.123235 |  | PMID:34503513 | Harshfield EL |  | 13814 |
| Triacylglycerol_56:7_[M+NH4]1+ levels | 8.8E-35 | -0.18674 |  | PMID:34503513 | Harshfield EL |  | 13814 |
| Triacylglycerol_54:3_[M+NH4]1+ levels | 6.9E-09 | 0.0897958 |  | PMID:34503513 | Harshfield EL |  | 13814 |
| Triacylglycerol_51:2_[M+NH4]1+ levels | 0.00035 | 0.0553073 |  | PMID:34503513 | Harshfield EL |  | 13814 |
| Triacylglycerol_50:2_[M+NH4]1+ levels | 0.0011 | 0.0507463 |  | PMID:34503513 | Harshfield EL |  | 13814 |
| Phosphatidylinositol-O_36:1_[M+H]1+ levels | 4.4E-10 | -0.0948879 |  | PMID:34503513 | Harshfield EL |  | 13814 |
| Triacylglycerol_52:6_[M+NH4]1+ levels | 3.1E-18 | -0.132042 |  | PMID:34503513 | Harshfield EL |  | 13814 |
| Red blood cell count | 1.59E-71 | 0.034695 |  | PMID:32888493 | Chen MH |  | 545203 |
| Hematocrit | 8.59E-39 | 0.025276 |  | PMID:32888493 | Chen MH |  | 562259 |
| Eosinophil percentage of white cells | 3.8E-11 | -0.0148217 |  | PMID:32888494 | Vuckovic D |  | 408112 |
| Statin medication | 1E-28 | -0.0927 | 0.911466899 |  | FINNGEN_R6 | 86466 | 260405 |
| Mood disorders (more control exclusions) | 0.00115 | 0.03352 | 1.034088125 |  | FINNGEN_R6 | 31552 | 229657 |
| Other heart diseases | 0.000331 | -0.02601 | 0.974325346 |  | FINNGEN_R6 | 77434 | 260405 |
| Valvular operations | 0.00382 | -0.02481 | 0.975495239 |  | FINNGEN_R6 | 44565 | 227536 |
| Pure hypercholesterolaemia | 0.00083 | -0.04625 | 0.954803232 |  | FINNGEN_R6 | 13092 | 244540 |
| Colorectal cancer (controls excluding all cancers) | 0.00332 | -0.06723 | 0.934980131 |  | FINNGEN_R6 | 4401 | 208471 |
| Cervicalgia | 0.00308 | -0.06876 | 0.933550705 |  | FINNGEN_R6 | 4043 | 198332 |
| Benign neoplasm: Caecum | 0.000359 | -0.1459 | 0.864244123 |  | FINNGEN_R6 | 1250 | 260405 |
| Doctor diagnosed asthma | 0.00242435 | -0.04449938 | 0.956476196 |  | UKB Neale v2 | 11717 | 91787 |
| Myeloid white cell count | 3.996E-10 | -0.02362008 |  | PMID:27863252 | Astle WJ |  | 169219 |
| Mean platelet volume | 9.092E-25 | -0.03895979 |  | PMID:27863252 | Astle WJ |  | 164454 |
| Eosinophil percentage of white cells | 0.000004281 | -0.01717888 |  | PMID:27863252 | Astle WJ |  | 172378 |
| Blood clot in the leg (dvt) | blood clot, dvt, bronchitis, emphysema, asthma, rhinitis, eczema, allergy diagnosed by doctor | 0.000676542 | -0.05937169 | 0.942356443 |  | UKB Neale v2 | 7386 | 360527 |
| For short-sightedness, i.e. only or mainly for distance viewing such as driving, cinema etc (called 'myopia') | reason for glasses/contact lenses | 4.03854E-05 | 0.037208246 | 1.037909139 |  | UKB Neale v2 | 29318 | 360677 |
| Dark brown | hair colour (natural, before greying) | 0.00431047 | -0.01459237 | 0.985513579 |  | UKB Neale v2 | 134627 | 360270 |
| Nvq or hnd or hnc or equivalent | qualifications | 0.00113539 | -0.02050846 | 0.979700404 |  | UKB Neale v2 | 66544 | 357549 |
| Eosinophill percentage | 1.70142E-16 | -0.0380707 |  |  | UKB Neale v2 |  | 349861 |
| Platelet count | 1.59474E-53 | 2.24188 |  |  | UKB Neale v2 |  | 350474 |
| Platelet distribution width | 2.08108E-10 | 0.00817596 |  |  | UKB Neale v2 |  | 350470 |
| Exposure to tobacco smoke at home | 0.00162855 | -0.00316557 |  |  | UKB Neale v2 |  | 326676 |
| Trunk predicted mass | 0.00457468 | -0.0218373 |  |  | UKB Neale v2 |  | 354494 |
| Heel quantitative ultrasound index (qui), direct entry (right) | 1.64427E-05 | 0.397063 |  |  | UKB Neale v2 |  | 114614 |
| Leg fat percentage (right) | 0.00393033 | 0.0394218 |  |  | UKB Neale v2 |  | 354811 |
| Salt added to food | 7.01545E-07 | 0.0104132 |  |  | UKB Neale v2 |  | 360954 |
| Duration of heavy diy | 0.000535442 | -0.0223099 |  |  | UKB Neale v2 |  | 145373 |
| Neutrophill percentage | 0.000421895 | -0.0739923 |  |  | UKB Neale v2 |  | 349861 |
| Average weekly spirits intake | 0.00332422 | -0.00756212 |  |  | UKB Neale v2 |  | 257426 |
| Skin colour | 0.000231158 | -0.00530536 |  |  | UKB Neale v2 |  | 356530 |
| Number of unsuccessful stop-smoking attempts | 0.00121589 | -0.0132765 |  |  | UKB Neale v2 |  | 81026 |
| Sex hormone-binding globulin levels adjusted for BMI | 2.9E-10 | -0.00756353 |  | PMID:32042192 | Ruth KS |  | 180094 |
| Bioavailable testosterone levels | 0.00024 | 0.0102165 |  | PMID:32042192 | Ruth KS |  | 188507 |
| Percentage of invited food questionnaires completed | 0.0019 | 0.290531 |  | PMID:33563987 | Tyrrell J |  | 300639 |
| Hypercholesterolemia | 0.00179 | -0.0282 | 0.972193909 |  | UKB SAIGE | 33242 | 406276 |
| Hemorrhoids | 0.00138 | -0.0324 | 0.968119257 |  | UKB SAIGE | 23896 | 393488 |
| Cholelithiasis or gall stones | 0.0046 | 0.00076988 | 1.000770176 | PMID:33959723 | Donertas HM | 7895 | 484598 |
| Other diseases of respiratory system, nec | 0.00373 | -0.0461 | 0.954946463 |  | UKB SAIGE | 8844 | 408369 |
| Eustachian tube disorders | 0.000141 | -0.41 | 0.66365025 |  | UKB SAIGE | 193 | 405081 |
| Stem cell factor levels | 8.439E-08 | 0.0571 |  | PMID:33067605 | Folkersen L |  | 21758 |
| Depression | 0.00336 | 0.01 | 1.010050167 | PMID:27089181 | Okbay A | 16471 | 75306 |
| Red cell distribution width | 2.2E-180 | -0.0573175 |  | PMID:34226706 | Barton AR |  | 441263 |
| Alanine aminotransferase levels | 2.1E-07 | -0.0115296 |  | PMID:34226706 | Barton AR |  | 437724 |
| Mean sphered cell volume | 4.7E-33 | -0.0230107 |  | PMID:34226706 | Barton AR |  | 437736 |
| Serum albumin levels | 0.00069 | 0.00830249 |  | PMID:34226706 | Barton AR |  | 400938 |
| Insulin disposition index | 0.001945 | 0.064 |  | PMID:24699409 | Prokopenko I |  | 5158 |
| Fasting blood glucose | 2.701E-08 | -0.021 |  | PMID:20081858 | Dupuis J |  | 46186 |
| Phosphatidylcholine-O_40:6_[M+H]1+/Phosphatidylcholine-P_40:5_[M+H]1+ levels | 1.3E-16 | -0.126881 |  | PMID:34503513 | Harshfield EL |  | 13814 |
| Lysophosphatidylcholine_20:4_[M+OAc]1- levels | 1.3E-194 | -0.290183 |  | PMID:34503513 | Harshfield EL |  | 13814 |
| Ceramide_41:1_[M-H]1- levels | 9.9E-07 | 0.051501 |  | PMID:34503513 | Harshfield EL |  | 13814 |
| Phosphatidylserine-O_38:2_[M+H]1+/Phosphatidylglycerol-O_38:4_[M+NH4]1+ levels | 0.000045 | 0.0537965 |  | PMID:34503513 | Harshfield EL |  | 13814 |
| Phosphatidylinositol-O_34:0_[M+H]1+ levels | 3E-07 | -0.0690532 |  | PMID:34503513 | Harshfield EL |  | 13814 |
| Phosphatidylinositol_38:5_[M-H]1- levels | 2.7E-08 | -0.057473 |  | PMID:34503513 | Harshfield EL |  | 13814 |
| Phosphatidylcholine_33:2_[M+OAc]1-/Phosphatidylserine_37:1_[M-H]1- levels | 1.4E-10 | 0.0569608 |  | PMID:34503513 | Harshfield EL |  | 13814 |
| Phosphatidylinositol_38:4_[M-H]1- levels | 2.3E-31 | -0.115465 |  | PMID:34503513 | Harshfield EL |  | 13814 |
| Phosphatidylserine-P_42:2_[M+H]1+ levels | 0.000016 | -0.0609486 |  | PMID:34503513 | Harshfield EL |  | 13814 |
| Lysophosphatidylethanolamine_22:6_[M-H]1- levels | 8.7E-07 | -0.0606505 |  | PMID:34503513 | Harshfield EL |  | 13814 |
| Lysophosphatidylcholine_18:2_[M+OAc]1-/Lysophosphatidylserine_22:1_[M-H]1- levels | 5.7E-08 | 0.0561992 |  | PMID:34503513 | Harshfield EL |  | 13814 |
| Phosphatidate_34:2_[M-H]1- levels | 1.2E-11 | 0.0766408 |  | PMID:34503513 | Harshfield EL |  | 13814 |
| Phosphatidylethanolamine-O_36:2_[M-H]1-/Phosphatidylethanolamine-P_36:1_[M-H]1- levels | 0.0011 | 0.0335267 |  | PMID:34503513 | Harshfield EL |  | 13814 |
| Lysophosphatidylethanolamine-O_16:1_[M-H]1-/Lysophosphatidylethanolamine-P_16:0_[M-H]1- levels | 0.00065 | -0.0369198 |  | PMID:34503513 | Harshfield EL |  | 13814 |
| Fatty acid(16:3)_[M-H]1- levels | 0.0045 | -0.0257366 |  | PMID:34503513 | Harshfield EL |  | 13814 |
| Lysophosphatidylethanolamine_20:0_[M-H]1- levels | 0.0008 | -0.0293387 |  | PMID:34503513 | Harshfield EL |  | 13814 |
| Phosphatidylcholine_35:6_[M+OAc]1-/Phosphatidylserine_39:5_[M-H]1- levels | 0.0026 | 0.0315873 |  | PMID:34503513 | Harshfield EL |  | 13814 |
| Triacylglycerol_58:9_[M+NH4]1+ levels | 4.8E-36 | -0.198153 |  | PMID:34503513 | Harshfield EL |  | 13814 |
| Cholesteryl ester_14:0_[M+NH4]1+ levels | 3.8E-11 | -0.0610936 |  | PMID:34503513 | Harshfield EL |  | 13814 |
| Triacylglycerol_54:6_[M+NH4]1+ levels | 7.4E-09 | -0.0886215 |  | PMID:34503513 | Harshfield EL |  | 13814 |
| Diacylglycerol_38:5_[M+H-H2O]1+ levels | 1.2E-74 | -0.157624 |  | PMID:34503513 | Harshfield EL |  | 13814 |
| Cholesteryl ester_18:2_[M+NH4]1+ levels | 4.1E-14 | 0.0804364 |  | PMID:34503513 | Harshfield EL |  | 13814 |
| Triacylglycerol_53:3_[M+NH4]1+ levels | 8.7E-08 | 0.0816376 |  | PMID:34503513 | Harshfield EL |  | 13814 |
| Triacylglycerol_46:5_[M+NH4]1+ levels | 0.000012 | -0.0623937 |  | PMID:34503513 | Harshfield EL |  | 13814 |
| Phosphatidylcholine-P_40:6_[M+H]1+ levels | 0.0019 | -0.039348 |  | PMID:34503513 | Harshfield EL |  | 13814 |
| Triacylglycerol_52:3_[M+NH4]1+ levels | 0.0000062 | 0.0690342 |  | PMID:34503513 | Harshfield EL |  | 13814 |
| Triacylglycerol_52:2_[M+NH4]1+ levels | 0.00024 | 0.0560963 |  | PMID:34503513 | Harshfield EL |  | 13814 |
| Phosphatidylserine-O_42:6_[M+H]1+ levels | 3.3E-09 | -0.0842267 |  | PMID:34503513 | Harshfield EL |  | 13814 |
| Platelet count | 8.33E-83 | 0.037068 |  | PMID:32888493 | Chen MH |  | 542827 |
| Neutrophil count | 2.36E-27 | -0.021904 |  | PMID:32888493 | Chen MH |  | 519288 |
| Mean corpuscular hemoglobin | 1.06E-08 | -0.011593 |  | PMID:32888493 | Chen MH |  | 486823 |
| Lymphocyte counts | 0.000033 | -0.00925034 |  | PMID:32888494 | Vuckovic D |  | 408112 |
| Mean reticulocyte volume | 1.2E-18 | -0.0197659 |  | PMID:32888494 | Vuckovic D |  | 408112 |
| Mean corpuscular hemoglobin | 4E-07 | -0.0111005 |  | PMID:32888494 | Vuckovic D |  | 408112 |
| High light scatter reticulocyte percentage of red cells | 0.0000094 | 0.00995291 |  | PMID:32888494 | Vuckovic D |  | 408112 |
| Mean corpuscular hemoglobin concentration | 5.2E-17 | 0.0193058 |  | PMID:32888494 | Vuckovic D |  | 408112 |
| Type 2 diabetes with other specified/multiple/unspecified complications | 0.00272 | -0.03265 | 0.967877257 |  | FINNGEN_R6 | 30103 | 245263 |
| Other heart diseases | 0.0000133 | -0.03695 | 0.96372432 |  | FINNGEN_R6 | 72780 | 208601 |
| Cardiovascular diseases (excluding rheumatic etc) | 0.0000951 | -0.02871 | 0.971698216 |  | FINNGEN_R6 | 124584 | 260405 |
| Cardiovascular diseases | 0.0000167 | -0.0312 | 0.969281697 |  | FINNGEN_R6 | 135546 | 260405 |
| Abdominal and pelvic pain | 0.00102 | 0.02408 | 1.024372264 |  | FINNGEN_R6 | 60359 | 251373 |
| Dermatitis and eczema | 0.0000537 | 0.04125 | 1.042112601 |  | FINNGEN_R6 | 24244 | 260405 |
| DVT of lower extremities and pulmonary embolism | 0.00000767 | -0.07015 | 0.932253971 |  | FINNGEN_R6 | 9808 | 260405 |
| Benign neoplasms (controls excluding all cancers) | 0.00249 | -0.02321 | 0.97705728 |  | FINNGEN_R6 | 62983 | 228682 |
| Polyuria | 0.00213 | -0.07045 | 0.931974337 |  | FINNGEN_R6 | 4089 | 244965 |
| Fish oil (including cod liver oil) | mineral and other dietary supplements | 5.39636E-05 | -0.02114393 | 0.97907804 |  | UKB Neale v2 | 114131 | 360016 |
| Mean corpuscular hemoglobin | 0.00008733 | -0.01457077 |  | PMID:27863252 | Astle WJ |  | 172332 |
| Sum eosinophil basophil counts | 1.406E-09 | -0.02267212 |  | PMID:27863252 | Astle WJ |  | 171771 |
| Red blood cell count | 1.833E-20 | 0.03458983 |  | PMID:27863252 | Astle WJ |  | 172952 |
| Red cell distribution width | 1.01E-40 | -0.04959426 |  | PMID:27863252 | Astle WJ |  | 171529 |
| Lymphocyte percentage of white cells | 0.00129 | 0.01202572 |  | PMID:27863252 | Astle WJ |  | 171748 |
| Hematocrit | 5.032E-11 | 0.02433458 |  | PMID:27863252 | Astle WJ |  | 173039 |
| Deep venous thrombosis (dvt) | non-cancer illness code, self-reported | 0.000979417 | -0.05815755 | 0.943501291 |  | UKB Neale v2 | 7237 | 361141 |
| Yes, because of illness | major dietary changes in the last 5 years | 0.00250079 | -0.02430802 | 0.975985039 |  | UKB Neale v2 | 38051 | 360294 |
| Other ulcerative colitis | 0.00445 | 0.13035 | 1.139227043 |  | FINNGEN_R6 | 1014 | 250719 |
| Impetigo | 0.00212 | 0.1646 | 1.178921456 |  | FINNGEN_R6 | 728 | 248555 |
| Sitting height | 1.89623E-05 | -0.0386557 |  |  | UKB Neale v2 |  | 360066 |
| Townsend deprivation index at recruitment | 0.000302974 | 0.0262792 |  |  | UKB Neale v2 |  | 360763 |
| Nitrogen dioxide air pollution; 2007 | 0.00180669 | 0.0773136 |  |  | UKB Neale v2 |  | 356078 |
| Spherical power (left) | 6.86396E-06 | -0.0677297 |  |  | UKB Neale v2 |  | 77739 |
| 6mm asymmetry index (left) | 0.00452847 | 0.210686 |  |  | UKB Neale v2 |  | 40683 |
| Heel bone mineral density (bmd) t-score, automated (right) | 1.64267E-05 | 0.0224175 |  |  | UKB Neale v2 |  | 114614 |
| Haemoglobin concentration | 4.21699E-31 | 0.0284154 |  |  | UKB Neale v2 |  | 350474 |
| Amount of alcohol drunk on a typical drinking day | 0.00261874 | 0.013692 |  |  | UKB Neale v2 |  | 108256 |
| Male-pattern baldness | 6.62673E-08 | -0.0350384 |  | PMID:28196072 | Hagenaars SP |  | 52874 |
| Appendicular lean mass | 0.000004 | -0.0129834 |  | PMID:33097823 | Pei YF |  | 205513 |
| Chronic hepatitis NAS | 0.000971 | 0.31136 | 1.365280634 |  | FINNGEN_R6 | 233 | 260405 |
| Fenofibrate | treatment/medication code | 7.65639E-06 | 0.301510952 | 1.351899922 |  | UKB Neale v2 | 488 | 361141 |
| Myocardial fractal dimension (slice 4) | 0.003682137 | -0.0010062 |  | PMID:32814899 | Meyer HV |  | 18096 |
| Sex hormone-binding globulin levels | 1E-20 | -0.0137795 |  | PMID:32042192 | Ruth KS |  | 189473 |
| Disorders of lipoid metabolism | 0.00151 | -0.0278 | 0.972582864 |  | UKB SAIGE | 35927 | 408961 |
| Nondiabetic hypoglycaemic coma | 0.00393 | -0.45508 | 0.634397214 |  | FINNGEN_R6 | 84 | 258882 |
| Other disorders of circulatory system | 0.00212 | -0.037 | 0.963676135 |  | UKB SAIGE | 16544 | 404449 |
| Hypothyroidism or myxedema | 6.2E-11 | -0.00292734 | 0.99707694 | PMID:33959723 | Donertas HM | 23497 | 484598 |
| HDL cholesterol | 0.000160648 | -0.06 |  | PMID:28887542 | Prins BP |  | 9796 |
| Degenerative skin conditions and other dermatoses | 0.00321 | -0.0597 | 0.942047105 |  | UKB SAIGE | 5522 | 404268 |
| Inflammatory bowel disease | 0.00009217 | 0.0509 | 1.052217666 | PMID:28067908 | de Lange KM | 25042 | 59957 |
| Nicotine metabolite ratio in current smokers | 0.002795 | -0.061632 |  | PMID:32157176 | Buchwald J |  | 5185 |
| Two-hour glucose | 0.004607 | -0.0233 |  | PMID:34059833 | Chen J |  | 63396 |
| Lung function (FEV1/FVC) | 0.00264 | -0.0075 |  | PMID:30804560 | Shrine N |  | 321047 |
| Bipolar disorder vs Tourette's syndrome and other tic disorders (ordinary least squares (OLS)) | 0.000092 | 0.015 | 1.015113065 | PMID:33686288 | Peyrot WJ | 25171 | 25171 |
| Ischemic stroke (large artery atherosclerosis) | 0.000316 | -0.0979 | 0.906739573 | PMID:29531354 | Malik R | 4373 | 410484 |
| Bipolar disorder vs ADHD (ordinary least squares (OLS)) | 0.0001 | 0.014 | 1.014098459 | PMID:33686288 | Peyrot WJ | 39451 | 39451 |
| Schizophrenia vs bipolar disorder (ordinary least squares (OLS)) | 0.001 | -0.0078 | 0.992230341 | PMID:33686288 | Peyrot WJ | 61027 | 61027 |
| Birth weight | 0.0006255 | 0.0206 |  | PMID:33955455 | Beck JJ |  | 42212 |
| Lymphocyte counts | 0.00054 | -0.0073091 |  | PMID:34226706 | Barton AR |  | 443762 |
| Hemoglobin A1c levels | 9E-47 | -0.0275464 |  | PMID:34226706 | Barton AR |  | 437749 |
| Cystatin C levels | 3.7E-28 | -0.0227476 |  | PMID:34226706 | Barton AR |  | 437846 |
| 3-methylglutaconate levels | 0.003064 | 0.0253 |  | PMID:33437055 | Panyard DJ |  | 291 |
| Triglycerides | 7.423E-38 | 0.0447 |  | PMID:24097068 | Willer CJ |  | 94595 |
| Corrected insulin response adjusted for insulin sensitivity index | 0.000945 | 0.072 |  | PMID:24699409 | Prokopenko I |  | 4978 |
| Triacylglycerol_58:10_[M+NH4]1+ levels | 7.8E-61 | -0.252879 |  | PMID:34503513 | Harshfield EL |  | 13814 |
| Phosphatidylglycerol_34:1_[M+H]1+ levels | 0.0000049 | -0.0582619 |  | PMID:34503513 | Harshfield EL |  | 13814 |
| Triacylglycerol_53:5_[M+NH4]1+ levels | 0.00098 | -0.0498766 |  | PMID:34503513 | Harshfield EL |  | 13814 |
| Phosphatidylcholine_36:3_[M+OAc]1-/Phosphatidylserine_40:2_[M-H]1- levels | 2.5E-143 | 0.256482 |  | PMID:34503513 | Harshfield EL |  | 13814 |
| Triacylglycerol_54:7_[M+NH4]1+ levels | 1.2E-15 | -0.122763 |  | PMID:34503513 | Harshfield EL |  | 13814 |
| Phosphatidylcholine_37:5_[M+OAc]1-/Phosphatidylserine_41:4_[M-H]1- levels | 3.6E-59 | -0.146768 |  | PMID:34503513 | Harshfield EL |  | 13814 |
| Phosphatidylethanolamine-O_38:3_[M-H]1-/Phosphatidylethanolamine-P_38:2_[M-H]1- levels | 4.6E-29 | 0.0999503 |  | PMID:34503513 | Harshfield EL |  | 13814 |
| Ceramide_42:1_[M-H]1- levels | 0.0000013 | 0.0506072 |  | PMID:34503513 | Harshfield EL |  | 13814 |
| Phosphatidylethanolamine-O_36:5_[M-H]1-/Phosphatidylethanolamine-P_36:4_[M-H]1- levels | 5.1E-33 | -0.144319 |  | PMID:34503513 | Harshfield EL |  | 13814 |
| Phosphatidylethanolamine_35:2_[M-H]1- levels | 1.6E-32 | 0.111527 |  | PMID:34503513 | Harshfield EL |  | 13814 |
| Lysophosphatidylethanolamine_18:2_[M-H]1- levels | 1.1E-44 | 0.168783 |  | PMID:34503513 | Harshfield EL |  | 13814 |
| Phosphatidylcholine_37:3_[M+OAc]1-/Phosphatidylserine_41:2_[M-H]1- levels | 1.1E-23 | 0.0905546 |  | PMID:34503513 | Harshfield EL |  | 13814 |
| Phosphatidylethanolamine_37:2_[M-H]1- levels | 6.7E-27 | 0.0945461 |  | PMID:34503513 | Harshfield EL |  | 13814 |
| Phosphatidylethanolamine-O_36:4_[M-H]1-/Phosphatidylethanolamine-P_36:3_[M-H]1- levels | 4.2E-19 | 0.10403 |  | PMID:34503513 | Harshfield EL |  | 13814 |
| Phosphatidylethanolamine_34:1_[M-H]1- levels | 0.0000023 | 0.0507602 |  | PMID:34503513 | Harshfield EL |  | 13814 |
| Phosphatidylcholine-O_35:4_[M+OAc]1-/Phosphatidylcholine-P_35:3_[M+OAc]1- levels | 1.4E-14 | -0.0722644 |  | PMID:34503513 | Harshfield EL |  | 13814 |
| Phosphatidylethanolamine_39:2_[M-H]1- levels | 0.0000032 | 0.0433204 |  | PMID:34503513 | Harshfield EL |  | 13814 |
| Lysophosphatidylethanolamine_20:2_[M-H]1- levels | 1.7E-09 | 0.0621237 |  | PMID:34503513 | Harshfield EL |  | 13814 |
| Diacylglycerol_34:2_[M+NH4]1+ levels | 0.00079 | 0.0359987 |  | PMID:34503513 | Harshfield EL |  | 13814 |
| Phosphatidylserine_36:1_[M+H]1+/Phosphatidylglycerol_36:3_[M+NH4]1+ levels | 2.5E-09 | -0.081524 |  | PMID:34503513 | Harshfield EL |  | 13814 |
| Diacylglycerol_38:6_[M+H-H2O]1+ levels | 2.9E-30 | -0.119482 |  | PMID:34503513 | Harshfield EL |  | 13814 |
| Ceramide_42:1_[M+H-H2O]1+ levels | 0.00018 | 0.0345916 |  | PMID:34503513 | Harshfield EL |  | 13814 |
| Phosphatidylcholine-O_38:4_[M+H]1+/Phosphatidylcholine-P_38:3_[M+H]1+ levels | 2.8E-13 | -0.111985 |  | PMID:34503513 | Harshfield EL |  | 13814 |
| Diacylglycerol_36:2_[M+H-H2O]1+ levels | 1.8E-12 | 0.0772979 |  | PMID:34503513 | Harshfield EL |  | 13814 |
| Phosphatidylglycerol_36:1_[M+H]1+ levels | 0.000018 | -0.0532982 |  | PMID:34503513 | Harshfield EL |  | 13814 |
| Phosphatidylcholine-O_36:2_[M+H]1+/Phosphatidylcholine-P_36:1_[M+H]1+/Phosphatidylethanolamine-P_39:1_[M+H]1+ levels | 2.5E-08 | 0.0690494 |  | PMID:34503513 | Harshfield EL |  | 13814 |
| Diacylglycerol_34:1_[M+H-H2O]1+ levels | 0.000023 | 0.0471748 |  | PMID:34503513 | Harshfield EL |  | 13814 |
| Phosphatidylcholine_38:2_[M+H]1+/Phosphatidylethanolamine_41:2_[M+H]1+ levels | 4.9E-27 | 0.169438 |  | PMID:34503513 | Harshfield EL |  | 13814 |
| Phosphatidylinositol-O_34:1_[M+H]1+ levels | 0.0035 | -0.0320828 |  | PMID:34503513 | Harshfield EL |  | 13814 |
| Triacylglycerol_52:1_[M+NH4]1+ levels | 0.000041 | 0.0647943 |  | PMID:34503513 | Harshfield EL |  | 13814 |
| Lysophosphatidylcholine-2O_18:0_[M+H]1+ levels | 1.6E-10 | -0.0752213 |  | PMID:34503513 | Harshfield EL |  | 13814 |
| Phosphatidylethanolamine-O_38:5_[M+H]1+/Phosphatidylethanolamine-P_38:4_[M+H]1+ levels | 2.6E-24 | -0.12156 |  | PMID:34503513 | Harshfield EL |  | 13814 |
| Fasting blood glucose adjusted for BMI | 4.87E-07 | -0.0183 |  | PMID:25625282 | Mahajan A |  | 33231 |
| Asthma | 2E-10 | -0.04354808 | 0.957386523 | PMID:34103634 | Valette K | 56167 | 408422 |
| Plateletcrit | 1E-18 | 0.0197101 |  | PMID:32888494 | Vuckovic D |  | 408112 |
| Neutrophil count | 1.4E-24 | -0.0231187 |  | PMID:32888494 | Vuckovic D |  | 408112 |
| Type 2 diabetes without complications | 0.00107 | -0.04185 | 0.959013622 |  | FINNGEN_R6 | 18839 | 233999 |
| IBD patients in KELA-register | 0.00000157 | 0.10369 | 1.109256532 |  | FINNGEN_R6 | 5047 | 254752 |
| Benign neoplasm: Caecum (controls excluding all cancers) | 0.000287 | -0.14877 | 0.861767299 |  | FINNGEN_R6 | 1250 | 209672 |
| Chalazion | 0.0044 | -0.08977 | 0.914141414 |  | FINNGEN_R6 | 2146 | 243506 |
| Alzheimer’s disease (Late onset) | 0.0021 | -0.08642 | 0.917208923 |  | FINNGEN_R6 | 3104 | 258981 |
| Crohn disease | 0.00348 | 0.0862 | 1.090024311 |  | FINNGEN_R6 | 2532 | 252237 |
| Ever sought or received professional help for mental distress | 0.00121154 | 0.028185181 | 1.028586141 |  | UKB Neale v2 | 46020 | 117677 |
| Sum neutrophil eosinophil counts | 5.387E-12 | -0.02592826 |  | PMID:27863252 | Astle WJ |  | 170384 |
| Ever unenthusiastic/disinterested for a whole week | 0.000172737 | 0.033901693 | 1.034482905 |  | UKB Neale v2 | 42374 | 115145 |
| Simvastatin | treatment/medication code | 1.01953E-06 | -0.03701742 | 0.963659352 |  | UKB Neale v2 | 40921 | 361141 |
| Fractured/broken bones in last 5 years | 0.00110847 | -0.02729053 | 0.973078488 |  | UKB Neale v2 | 34780 | 359241 |
| Long-standing illness, disability or infirmity | 0.000397494 | -0.01873352 | 0.981440861 |  | UKB Neale v2 | 114798 | 352798 |
| Asthma | non-cancer illness code, self-reported | 4.89167E-11 | -0.05075067 | 0.950515638 |  | UKB Neale v2 | 41934 | 361141 |
| No | breathing problems improved/stopped away from workplace or on holiday | 0.000253413 | -0.08418271 | 0.919263284 |  | UKB Neale v2 | 4382 | 91149 |
| Irritability | 1.72371E-06 | 0.026756646 | 1.027117819 |  | UKB Neale v2 | 96862 | 345231 |
| Seen doctor (gp) for nerves, anxiety, tension or depression | 0.0014838 | 0.016375374 | 1.016510185 |  | UKB Neale v2 | 123528 | 358693 |
| Trunk fat-free mass | 0.00307916 | -0.0237703 |  |  | UKB Neale v2 |  | 354530 |
| Reticulocyte percentage | 1.04472E-05 | 0.0101252 |  |  | UKB Neale v2 |  | 344728 |
| Number of treatments/medications taken | 1.44591E-06 | -0.00921985 |  |  | UKB Neale v2 |  | 361141 |
| Heel bone mineral density (bmd) (right) | 1.57735E-05 | 0.00250383 |  |  | UKB Neale v2 |  | 114552 |
| Pulse rate, automated reading | 1.72759E-40 | 0.381377 |  |  | UKB Neale v2 |  | 340162 |
| Fluid intelligence score | 0.000267717 | 0.032956 |  |  | UKB Neale v2 |  | 117131 |
| Mean corpuscular haemoglobin concentration | 1.13449E-08 | 0.01515 |  |  | UKB Neale v2 |  | 350468 |
| White blood cell (leukocyte) count | 4.61063E-09 | -0.0298563 |  |  | UKB Neale v2 |  | 350470 |
| Red blood cell (erythrocyte) count | 1.39015E-37 | 0.0112251 |  |  | UKB Neale v2 |  | 350475 |
| Average weekly champagne plus white wine intake | 3.55506E-08 | 0.0136622 |  |  | UKB Neale v2 |  | 257623 |
| Number of fluid intelligence questions attempted within time limit | 0.00238527 | 0.0116726 |  |  | UKB Neale v2 |  | 117131 |
| Fluticasone | treatment/medication code | 0.00160231 | -0.11841163 | 0.888330318 |  | UKB Neale v2 | 1578 | 361141 |
| Low density lipoprotein cholesterol levels | 1E-90 | -0.0406179 |  | PMID:32493714 | Klimentidis YC |  | 431167 |
| Asthma | 5.86E-07 | -0.0494 | 0.951800333 |  | UKB SAIGE | 26332 | 401837 |
| Benign neoplasm of colon | 4.61E-08 | -0.0605 | 0.941293769 |  | UKB SAIGE | 20204 | 406215 |
| Hypothyroidism nos | 0.00000698 | -0.0596 | 0.942141315 |  | UKB SAIGE | 14171 | 405600 |
| Thyroid problem (not cancer) | 2.6E-10 | -0.00312296 | 0.996881911 | PMID:33959723 | Donertas HM | 28254 | 484598 |
| Respiratory mometasone | treatment/medication code | 0.00249828 | -0.30619998 | 0.736239373 |  | UKB Neale v2 | 216 | 361141 |
| Deep venous thrombosis | 0.00016 | -0.00109149 | 0.998909105 | PMID:33959723 | Donertas HM | 9529 | 484598 |
| Other headache syndromes | 0.00162 | 0.0538 | 1.055273526 |  | UKB SAIGE | 7891 | 406671 |
| Myocardial infarction | 0.004558 | -0.02270788 | 0.977548 | PMID:33532862 | Hartiala JA | 61505 | 639221 |
| Inflammation of the eye | 0.00167 | -0.0831 | 0.920259117 |  | UKB SAIGE | 3174 | 402480 |
| Large proline-rich protein BAG6 levels | 0.003226828 | -0.1363892 |  | PMID:33303764 | Gilly A |  | 1313 |
| Abnormal heart sounds | 0.000793 | -0.155 | 0.856415177 |  | UKB SAIGE | 1049 | 403470 |
| ERO1-like protein beta measurement | 0.0003162 | 0.0496 |  | PMID:33328453 | Pietzner M |  | 10708 |
| O-phosphoseryl-tRNA(Sec) selenium transferase measurement | 0.0005743 | 0.0471 |  | PMID:33328453 | Pietzner M |  | 10708 |
| TNF-related apoptosis-inducing ligand levels | 9.132E-07 | 0.0575 |  | PMID:33067605 | Folkersen L |  | 21758 |
| Hemoglobin levels | 1.5E-48 | 0.0301141 |  | PMID:34226706 | Barton AR |  | 445373 |
| Total cholesterol levels | 1.7E-111 | -0.046981 |  | PMID:34226706 | Barton AR |  | 437878 |
| Reticulocyte count | 4.2E-17 | 0.016879 |  | PMID:34226706 | Barton AR |  | 437291 |
| Insulin-like growth factor 1 levels | 1.7E-22 | -0.0199699 |  | PMID:34226706 | Barton AR |  | 435516 |
| Urate levels | 0.001715093 | 0.019 |  | PMID:23263486 | Kottgen A |  | 110347 |
| HDL cholesterol | 8.301E-28 | -0.0391 |  | PMID:24097068 | Willer CJ |  | 94595 |
| LDL cholesterol | 1.625E-39 | -0.0512 |  | PMID:24097068 | Willer CJ |  | 94595 |
| Homeostasis model assessment of beta-cell function | 0.0001061 | 0.013 |  | PMID:20081858 | Dupuis J |  | 36466 |
| Phosphatidylinositol_36:0_[M+H]1+ levels | 2E-37 | -0.155405 |  | PMID:34503513 | Harshfield EL |  | 13814 |
| Phosphatidylethanolamine-O_40:5_[M-H]1-/Phosphatidylethanolamine-P_40:4_[M-H]1- levels | 4E-72 | -0.163009 |  | PMID:34503513 | Harshfield EL |  | 13814 |
| Phosphatidylcholine_37:4_[M+OAc]1-/Phosphatidylserine_41:3_[M-H]1- levels | 6.1E-110 | -0.201357 |  | PMID:34503513 | Harshfield EL |  | 13814 |
| Phosphatidylethanolamine_38:3_[M-H]1- levels | 6E-82 | 0.150992 |  | PMID:34503513 | Harshfield EL |  | 13814 |
| Phosphatidylethanolamine-O_38:5_[M-H]1-/Phosphatidylethanolamine-P_38:4_[M-H]1- levels | 7.4E-48 | -0.167158 |  | PMID:34503513 | Harshfield EL |  | 13814 |
| Phosphatidylethanolamine_38:5_[M-H]1- levels | 3.9E-63 | -0.174843 |  | PMID:34503513 | Harshfield EL |  | 13814 |
| Diacylglycerol_36:2_[M+NH4]1+ levels | 0.000026 | 0.0583567 |  | PMID:34503513 | Harshfield EL |  | 13814 |
| Phosphatidylcholine_34:3_[M+OAc]1-/Phosphatidylserine_38:2_[M-H]1- levels | 1.6E-42 | 0.149704 |  | PMID:34503513 | Harshfield EL |  | 13814 |
| Phosphatidylserine_40:3_[M+H]1+/Phosphatidylglycerol_40:5_[M+NH4]1+ levels | 1.9E-27 | 0.130734 |  | PMID:34503513 | Harshfield EL |  | 13814 |
| Phosphatidylcholine_38:7_[M+OAc]1-/Phosphatidylserine_42:6_[M-H]1- levels | 6.2E-47 | 0.152508 |  | PMID:34503513 | Harshfield EL |  | 13814 |
| Phosphatidylcholine_36:7_[M+OAc]1-/Phosphatidylserine_40:6_[M-H]1- levels | 2.6E-30 | 0.128551 |  | PMID:34503513 | Harshfield EL |  | 13814 |
| Phosphatidylcholine-O_44:5_[M+OAc]1-/Phosphatidylcholine-P_44:4_[M+OAc]1- levels | 2.9E-36 | -0.123488 |  | PMID:34503513 | Harshfield EL |  | 13814 |
| Phosphatidylcholine_36:2_[M+OAc]1-/Phosphatidylserine_40:1_[M-H]1- levels | 1.2E-34 | 0.12007 |  | PMID:34503513 | Harshfield EL |  | 13814 |
| Phosphatidylethanolamine_38:2_[M-H]1- levels | 5.3E-22 | 0.0735809 |  | PMID:34503513 | Harshfield EL |  | 13814 |
| Phosphatidylcholine_32:2_[M+OAc]1-/Phosphatidylserine_36:1_[M-H]1- levels | 4.1E-28 | 0.130295 |  | PMID:34503513 | Harshfield EL |  | 13814 |
| Phosphatidylcholine-O_40:6_[M+OAc]1-/Phosphatidylcholine-P_40:5_[M+OAc]1- levels | 4.2E-22 | -0.098135 |  | PMID:34503513 | Harshfield EL |  | 13814 |
| Phosphatidylethanolamine-O_36:3_[M-H]1-/Phosphatidylethanolamine-P_36:2_[M-H]1- levels | 1.4E-17 | 0.0921369 |  | PMID:34503513 | Harshfield EL |  | 13814 |
| Phosphatidylcholine_44:5_[M+OAc]1- levels | 2.9E-24 | -0.102476 |  | PMID:34503513 | Harshfield EL |  | 13814 |
| Phosphatidylethanolamine_34:0_[M-H]1- levels | 6.1E-14 | -0.055455 |  | PMID:34503513 | Harshfield EL |  | 13814 |
| Phosphatidylcholine_36:6_[M+OAc]1-/Phosphatidylserine_40:5_[M-H]1- levels | 2E-09 | 0.0636299 |  | PMID:34503513 | Harshfield EL |  | 13814 |
| Phosphatidylethanolamine_40:6_[M-H]1- levels | 4.2E-13 | -0.0704865 |  | PMID:34503513 | Harshfield EL |  | 13814 |
| Phosphatidylcholine_37:6_[M+OAc]1-/Phosphatidylserine_41:5_[M-H]1- levels | 3.1E-11 | -0.0608592 |  | PMID:34503513 | Harshfield EL |  | 13814 |
| Phosphatidylserine-O_40:4_[M+H]1+/Phosphatidylglycerol-O_40:6_[M+NH4]1+ levels | 0.000022 | -0.0575408 |  | PMID:34503513 | Harshfield EL |  | 13814 |
| Sphingomyelin_41:2_[M+OAc]1- levels | 0.00067 | -0.0299988 |  | PMID:34503513 | Harshfield EL |  | 13814 |
| Phosphatidylethanolamine-P_40:6_[M-H]1- levels | 0.002 | -0.0351966 |  | PMID:34503513 | Harshfield EL |  | 13814 |
| Cholesteryl ester_17:0_[M+NH4]1+ levels | 2.9E-07 | -0.0500389 |  | PMID:34503513 | Harshfield EL |  | 13814 |
| Cholesteryl ester_18:0_[M+NH4]1+ levels | 0.00076 | -0.0298139 |  | PMID:34503513 | Harshfield EL |  | 13814 |
| Cholesteryl ester_22:6_[M+NH4]1+ levels | 1E-31 | -0.143608 |  | PMID:34503513 | Harshfield EL |  | 13814 |
| Phosphatidylcholine_33:2_[M+H]1+/Phosphatidylethanolamine_36:2_[M+H]1+/Phosphatidate_38:3_[M+NH4]1+ levels | 8.5E-102 | 0.251214 |  | PMID:34503513 | Harshfield EL |  | 13814 |
| Triacylglycerol_56:5_[M+NH4]1+ levels | 3.3E-30 | -0.173948 |  | PMID:34503513 | Harshfield EL |  | 13814 |
| Diacylglycerol_38:4_[M+H-H2O]1+ levels | 4.6E-70 | -0.134527 |  | PMID:34503513 | Harshfield EL |  | 13814 |
| Triacylglycerol_56:6_[M+NH4]1+ levels | 3.9E-63 | -0.246599 |  | PMID:34503513 | Harshfield EL |  | 13814 |
| Lysophosphatidylcholine-2O_16:0_[M+H]1+ levels | 7E-07 | -0.0569675 |  | PMID:34503513 | Harshfield EL |  | 13814 |
| Sphingomyelin_34:1_[M+H]1+ levels | 0.00049 | -0.0436136 |  | PMID:34503513 | Harshfield EL |  | 13814 |
| Phosphatidylcholine_38:6_[M+H]1+/Phosphatidylethanolamine_41:6_[M+H]1+ levels | 2.9E-17 | -0.131852 |  | PMID:34503513 | Harshfield EL |  | 13814 |
| Phosphatidylcholine_33:3_[M+H]1+/Phosphatidylethanolamine_36:3_[M+H]1+/Phosphatidate_38:4_[M+NH4]1+ levels | 1.3E-52 | 0.151301 |  | PMID:34503513 | Harshfield EL |  | 13814 |
| Hemoglobin concentration | 5.65E-55 | 0.030291 |  | PMID:32888493 | Chen MH |  | 563946 |
| High light scatter reticulocyte count | 1.9E-11 | 0.0150695 |  | PMID:32888494 | Vuckovic D |  | 408112 |
| Eosinophil counts | 1.7E-22 | -0.0217971 |  | PMID:32888494 | Vuckovic D |  | 408112 |
| Red blood cell count | 3.5E-54 | 0.034189 |  | PMID:32888494 | Vuckovic D |  | 408112 |
| Diseases of the ear and mastoid process | 0.000482 | 0.0264 | 1.026751567 |  | FINNGEN_R6 | 49615 | 260405 |
| Type 2 diabetes | 0.000212 | -0.03555 | 0.965074479 |  | FINNGEN_R6 | 41245 | 256405 |
| Diseases of appendix | 0.002 | 0.03382 | 1.034398398 |  | FINNGEN_R6 | 20330 | 260405 |
| Anxiety disorders (more control exclusions) | 0.00411 | 0.03195 | 1.032465881 |  | FINNGEN_R6 | 25224 | 223329 |
| Depression | 0.000636 | 0.03449 | 1.035091677 |  | FINNGEN_R6 | 28098 | 256915 |
| Diseases of middle ear and mastoid | 0.00261 | 0.03788 | 1.038606593 |  | FINNGEN_R6 | 15420 | 260405 |
| Asthma/COPD (KELA code 203) | 0.00385 | -0.03129 | 0.969194466 |  | FINNGEN_R6 | 24334 | 244792 |
| Inflammatory bowel disease | 0.0000369 | 0.07441 | 1.077248387 |  | FINNGEN_R6 | 7206 | 260405 |
| Alzheimer disease | 0.00488 | -0.06645 | 0.9357097 |  | FINNGEN_R6 | 4500 | 260405 |
| Mucosal proctocolitis | 0.0000103 | 0.13181 | 1.140891529 |  | FINNGEN_R6 | 2468 | 252173 |
| Nasal polyp | 0.0000181 | -0.10384 | 0.901369505 |  | FINNGEN_R6 | 3919 | 203127 |
| Miserableness | 0.0010609 | 0.016246203 | 1.01637889 |  | UKB Neale v2 | 151752 | 355182 |
| Platelet count | 2.873E-22 | 0.03710347 |  | PMID:27863252 | Astle WJ |  | 166066 |
| Benign mammary dysplasia | 0.000785 | -0.08999 | 0.913940325 |  | FINNGEN_R6 | 3031 | 139645 |
| Wheeze or whistling in the chest in last year | 1.73169E-06 | -0.02937307 | 0.971054127 |  | UKB Neale v2 | 73828 | 354523 |
| Salbutamol | treatment/medication code | 0.00333786 | -0.06693112 | 0.93525962 |  | UKB Neale v2 | 4305 | 361141 |
| Plateletcrit | 1.188E-08 | 0.0218714 |  | PMID:27863252 | Astle WJ |  | 164339 |
| Levothyroxine sodium | treatment/medication code | 1.12481E-07 | -0.06582888 | 0.93629107 |  | UKB Neale v2 | 14689 | 361141 |
| Hemoglobin concentration | 1.036E-13 | 0.02766451 |  | PMID:27863252 | Astle WJ |  | 172925 |
| Mean corpuscular hemoglobin concentration | 0.00009765 | 0.01415378 |  | PMID:27863252 | Astle WJ |  | 172851 |
| None of the above | medication for pain relief, constipation, heartburn | 0.00353902 | 0.014528117 | 1.014634163 |  | UKB Neale v2 | 159041 | 357084 |
| Asthma | blood clot, dvt, bronchitis, emphysema, asthma, rhinitis, eczema, allergy diagnosed by doctor | 3.88726E-10 | -0.04846903 | 0.952686848 |  | UKB Neale v2 | 41633 | 360527 |
| Hearing aid user | 0.00376276 | -0.04182347 | 0.959039062 |  | UKB Neale v2 | 10942 | 219358 |
| Heavy diy (eg: weeding, lawn mowing, carpentry, digging) | types of physical activity in last 4 weeks | 0.000176162 | -0.01830882 | 0.981857768 |  | UKB Neale v2 | 156597 | 359263 |
| None of the above | mineral and other dietary supplements | 0.00140275 | 0.015607657 | 1.015730093 |  | UKB Neale v2 | 155206 | 360016 |
| Heart attack/myocardial infarction | non-cancer illness code, self-reported | 0.002628 | -0.04924611 | 0.951946817 |  | UKB Neale v2 | 8239 | 361141 |
| Benign neoplasm: Ascending colon (controls excluding all cancers) | 0.00194 | -0.11416 | 0.892115206 |  | FINNGEN_R6 | 1605 | 209792 |
| Leg | fractured bone site(s) | 0.00233014 | -0.0945306 | 0.909799899 |  | UKB Neale v2 | 2311 | 359241 |
| Platelet crit | 2.48282E-16 | 0.000958668 |  |  | UKB Neale v2 |  | 350471 |
| Getting up in morning | 3.99336E-05 | -0.00765334 |  |  | UKB Neale v2 |  | 360231 |
| Mean reticulocyte volume | 1.12786E-12 | -0.139378 |  |  | UKB Neale v2 |  | 344728 |
| Heel bone mineral density (bmd) | 4.13733E-05 | 0.00176586 |  |  | UKB Neale v2 |  | 206496 |
| Arm fat percentage (right) | 0.00389695 | 0.0535502 |  |  | UKB Neale v2 |  | 354760 |
| Number of vehicles in household | 0.000316683 | -0.00754674 |  |  | UKB Neale v2 |  | 358754 |
| Forced expiratory volume in 1-second (fev1), predicted | 9.18579E-05 | -0.00363823 |  |  | UKB Neale v2 |  | 117241 |
| Neutrophill count | 1.45162E-09 | -0.0213367 |  |  | UKB Neale v2 |  | 349856 |
| Reticulocyte count | 2.33312E-09 | 0.000589229 |  |  | UKB Neale v2 |  | 344729 |
| Maximum heart rate during fitness test | 0.00197762 | 0.380651 |  |  | UKB Neale v2 |  | 53984 |
| Red cell distribution width | 4.1E-38 | -0.0518822 |  | PMID:28957414 | Pilling LC |  | 116666 |
| Home location at assessment - north co-ordinate (rounded) | 0.00304584 | -954.917 |  |  | UKB Neale v2 |  | 357793 |
| Sleep duration | 0.0029 | 0.0128215 |  | PMID:27494321 | Jones SE |  | 127573 |
| Pyogenic granuloma | 0.00298 | -0.27793 | 0.757349834 |  | FINNGEN_R6 | 236 | 246845 |
| Secondary malignant neoplasm of other and unspecified sites (controls excluding all cancers) | 0.000958 | -0.31386 | 0.730621308 |  | FINNGEN_R6 | 230 | 204300 |
| Liver enzyme levels (alanine transaminase) | 0.0000036 | -0.00188039 |  | PMID:33972514 | Pazoki R |  | 437267 |
| Hyperlipidemia | 0.00184 | -0.0273 | 0.973069277 |  | UKB SAIGE | 35844 | 408878 |
| Cholelithiasis | 1.23E-09 | 0.0803 | 1.083612103 |  | UKB SAIGE | 13777 | 405084 |
| Cholelithiasis and cholecystitis | 6.07E-08 | 0.0664 | 1.068654093 |  | UKB SAIGE | 16225 | 407532 |
| Liver, biliary or pancreas problem | 0.0021 | 0.00107994 | 1.001080523 | PMID:33959723 | Donertas HM | 13495 | 484598 |
| Phlebitis and thrombophlebitis | 0.00292 | -0.0718 | 0.930717021 |  | UKB SAIGE | 3900 | 373492 |
| Falling risk | 0.000086 | -0.0184786 | 0.981691083 | PMID:32999390 | Trajanoska K | 89076 | 451179 |
| Complement C1r subcomponent-like protein measurement | 0.002232 | 0.0427 |  | PMID:33328453 | Pietzner M |  | 10708 |
| D-dimer measurement | 0.001258 | 0.0454 |  | PMID:33328453 | Pietzner M |  | 10708 |
| Bipolar disorder vs obsessive compulsive disorder (ordinary least squares (OLS)) | 0.0035 | 0.0096 | 1.009646228 | PMID:33686288 | Peyrot WJ | 23040 | 23040 |
| Serum alkaline phosphatase levels | 3.6E-154 | 0.0492135 |  | PMID:34226706 | Barton AR |  | 437896 |
| C-reactive protein levels | 1.2E-12 | -0.0149171 |  | PMID:34226706 | Barton AR |  | 436939 |
| Sex hormone-binding globulin levels | 8.8E-31 | -0.0238264 |  | PMID:34226706 | Barton AR |  | 397043 |
| Direct bilirubin levels | 3.6E-08 | -0.0121374 |  | PMID:34226706 | Barton AR |  | 372420 |
| Circulating plasma alpha-Klotho levels | 0.002576 | 0.0665 |  | PMID:34542150 | Gergei I |  | 4675 |
| X-10457 levels | 0.002139 | 0.0419 |  | PMID:33437055 | Panyard DJ |  | 291 |
| Orotidine levels | 0.004718 | 0.0358 |  | PMID:33437055 | Panyard DJ |  | 291 |
| Phosphatidylinositol_34:0_[M+H]1+ levels | 2.2E-40 | -0.178344 |  | PMID:34503513 | Harshfield EL |  | 13814 |
| Phosphatidylinositol_36:2_[M+NH4]1+ levels | 4.4E-29 | 0.153207 |  | PMID:34503513 | Harshfield EL |  | 13814 |
| Ceramide_42:1_[M+H]1+ levels | 8.2E-07 | 0.0546933 |  | PMID:34503513 | Harshfield EL |  | 13814 |
| Phosphatidylcholine-O_38:5_[M+OAc]1-/Phosphatidylcholine-P_38:4_[M+OAc]1-/Phosphatidylserine-O_42:4_[M-H]1- levels | 2.2E-78 | -0.190438 |  | PMID:34503513 | Harshfield EL |  | 13814 |
| Phosphatidylcholine_36:5_[M+OAc]1-/Phosphatidylserine_40:4_[M-H]1- levels | 1.7E-96 | -0.238008 |  | PMID:34503513 | Harshfield EL |  | 13814 |
| Phosphatidylethanolamine_36:2_[M-H]1- levels | 1.1E-54 | 0.120068 |  | PMID:34503513 | Harshfield EL |  | 13814 |
| Phosphatidylethanolamine_36:3_[M-H]1- levels | 5.4E-61 | 0.177944 |  | PMID:34503513 | Harshfield EL |  | 13814 |
| Phosphatidylinositol_36:2_[M-H]1- levels | 2E-44 | 0.147919 |  | PMID:34503513 | Harshfield EL |  | 13814 |
| Phosphatidylcholine_40:4_[M+OAc]1-/Phosphatidylserine_44:3_[M-H]1- levels | 6.1E-43 | -0.137445 |  | PMID:34503513 | Harshfield EL |  | 13814 |
| Phosphatidylglycerol_35:3_[M-H]1- levels | 9.1E-30 | 0.0894456 |  | PMID:34503513 | Harshfield EL |  | 13814 |
| Phosphatidylcholine-O_38:6_[M+OAc]1-/Phosphatidylcholine-P_38:5_[M+OAc]1-/Phosphatidylserine-P_42:1_[M-H]1- levels | 1.1E-39 | -0.140443 |  | PMID:34503513 | Harshfield EL |  | 13814 |
| Phosphatidylcholine-O_36:4_[M+OAc]1-/Phosphatidylcholine-P_36:3_[M+OAc]1-/Phosphatidylserine-O_40:3_[M-H]1- levels | 7.4E-38 | -0.137528 |  | PMID:34503513 | Harshfield EL |  | 13814 |
| Sphingomyelin_38:2_[M+OAc]1- levels | 5.3E-08 | -0.053713 |  | PMID:34503513 | Harshfield EL |  | 13814 |
| Fatty acid(22:4)_[M-H]1- levels | 3.7E-38 | -0.138599 |  | PMID:34503513 | Harshfield EL |  | 13814 |
| Phosphatidylcholine_40:6_[M+OAc]1-/Phosphatidylserine_44:5_[M-H]1- levels | 1.5E-26 | -0.1126 |  | PMID:34503513 | Harshfield EL |  | 13814 |
| Phosphatidylcholine_35:2_[M+OAc]1-/Phosphatidylserine_39:1_[M-H]1- levels | 3.4E-13 | 0.0651585 |  | PMID:34503513 | Harshfield EL |  | 13814 |
| Phosphatidylethanolamine_36:4_[M-H]1- levels | 0.00011 | -0.0464 |  | PMID:34503513 | Harshfield EL |  | 13814 |
| Phosphatidylcholine-P_40:6_[M+OAc]1- levels | 0.000011 | -0.0460857 |  | PMID:34503513 | Harshfield EL |  | 13814 |
| Phosphatidylinositol-O_35:0_[M-H]1- levels | 0.00013 | -0.0354934 |  | PMID:34503513 | Harshfield EL |  | 13814 |
| Phosphatidylcholine_40:10_[M+OAc]1- levels | 0.000034 | -0.0429163 |  | PMID:34503513 | Harshfield EL |  | 13814 |
| Phosphatidylcholine-O_34:4_[M+OAc]1-/Phosphatidylcholine-P_34:3_[M+OAc]1-/Phosphatidylserine-O_38:3_[M-H]1- levels | 0.0034 | -0.0303237 |  | PMID:34503513 | Harshfield EL |  | 13814 |
| Triacylglycerol_56:4_[M+NH4]1+ levels | 1.9E-16 | 0.126499 |  | PMID:34503513 | Harshfield EL |  | 13814 |
| Phosphatidylinositol_40:6_[M-H]1- levels | 0.00022 | 0.0411342 |  | PMID:34503513 | Harshfield EL |  | 13814 |
| Phosphatidylethanolamine-O_34:2_[M-H]1-/Phosphatidylethanolamine-P_34:1_[M-H]1- levels | 0.0000035 | 0.0496537 |  | PMID:34503513 | Harshfield EL |  | 13814 |
| Phosphatidylcholine-O_35:3_[M+OAc]1-/Phosphatidylcholine-P_35:2_[M+OAc]1-/Phosphatidylserine-P_39:1_[M-H]1- levels | 0.000027 | 0.0428643 |  | PMID:34503513 | Harshfield EL |  | 13814 |
| Phosphatidylcholine_39:6_[M+OAc]1- levels | 3E-10 | -0.0587599 |  | PMID:34503513 | Harshfield EL |  | 13814 |
| Cholesteryl ester_15:0_[M+NH4]1+ levels | 5.8E-10 | -0.064592 |  | PMID:34503513 | Harshfield EL |  | 13814 |
| Ceramide_42:2_[M+H]1+ levels | 0.000023 | 0.0514417 |  | PMID:34503513 | Harshfield EL |  | 13814 |
| Phosphatidylcholine_34:2_[M+H]1+/Phosphatidylethanolamine_37:2_[M+H]1+/Phosphatidate_39:3_[M+NH4]1+ levels | 2.7E-80 | 0.222379 |  | PMID:34503513 | Harshfield EL |  | 13814 |
| Cholesteryl ester_18:3_[M+NH4]1+ levels | 1.9E-42 | -0.146205 |  | PMID:34503513 | Harshfield EL |  | 13814 |
| Triacylglycerol_58:8_[M+NH4]1+ levels | 1.3E-17 | -0.135233 |  | PMID:34503513 | Harshfield EL |  | 13814 |
| Triacylglycerol_57:11_[M+NH4]1+ levels | 2.6E-08 | -0.0845949 |  | PMID:34503513 | Harshfield EL |  | 13814 |
| Phosphatidylcholine_34:4_[M+H]1+/Phosphatidylethanolamine_37:4_[M+H]1+/Phosphatidate_39:5_[M+NH4]1+ levels | 9.8E-23 | -0.125925 |  | PMID:34503513 | Harshfield EL |  | 13814 |
| Phosphatidylcholine_38:5_[M+H]1+/Phosphatidylethanolamine_41:5_[M+H]1+/Phosphatidate_43:6_[M+NH4]1+ levels | 1.4E-120 | -0.358514 |  | PMID:34503513 | Harshfield EL |  | 13814 |
| Phosphatidylcholine-O_31:2_[M+H]1+/Phosphatidylcholine-P_31:1_[M+H]1+/Phosphatidylethanolamine-O_34:2_[M+H]1+ levels | 3.7E-15 | -0.0881461 |  | PMID:34503513 | Harshfield EL |  | 13814 |
| Triacylglycerol_58:7_[M+NH4]1+ levels | 4.7E-27 | -0.166932 |  | PMID:34503513 | Harshfield EL |  | 13814 |
| Cholesteryl ester_18:1_[M+NH4]1+ levels | 0.000011 | -0.039362 |  | PMID:34503513 | Harshfield EL |  | 13814 |
| Sphingomyelin_36:1_[M+H]1+ levels | 0.0013 | -0.03963 |  | PMID:34503513 | Harshfield EL |  | 13814 |
| Diacylglycerol_44:7_[M+H-H2O]1+ levels | 0.00038 | -0.0428482 |  | PMID:34503513 | Harshfield EL |  | 13814 |
| Diacylglycerol_36:1_[M+H-H2O]1+ levels | 0.000023 | 0.0493699 |  | PMID:34503513 | Harshfield EL |  | 13814 |
| Phosphatidylcholine_36:2_[M+H]1+/Phosphatidylethanolamine_39:2_[M+H]1+/Phosphatidate_41:3_[M+NH4]1+ levels | 7.5E-34 | 0.186785 |  | PMID:34503513 | Harshfield EL |  | 13814 |
| Triacylglycerol_54:4_[M+NH4]1+ levels | 0.0000028 | 0.0726822 |  | PMID:34503513 | Harshfield EL |  | 13814 |
| Triacylglycerol_52:4_[M+NH4]1+ levels | 0.0044 | 0.0441662 |  | PMID:34503513 | Harshfield EL |  | 13814 |
| Sphingomyelin_32:2_[M+H]1+ levels | 3.5E-07 | -0.0563827 |  | PMID:34503513 | Harshfield EL |  | 13814 |
| Diastolic blood pressure | 0.0001918 | 0.006221504 |  | PMID:33230300 | Surendran P |  | 810865 |

**Supplementary Table S5. PheWAS associations with rs174546 in OpenTarget Genetics.** A p-value equal to or less than 5*e*−8 was considered statistically significant.

| **Study ID** | **Reported Trait** | **PMID** | **Author** | **Date** | **N** | **Index Variant RSID** | **P-Value** | **Beta** |
| --- | --- | --- | --- | --- | --- | --- | --- | --- |
| GCST006571 | Educational attainment (MTAG) [MTAG] | PMID:30038396 | Lee JJ | 2018-07-23 | 1131438 | rs76167224 | 2E-09 | 0.0186 |
| GCST90025994 | Body mass index | PMID:34226706 | Barton AR | 2021-07-05 | 457756 | rs2292238 | 3.3E-16 | -0.0162 |
| NEALE2_21002_raw | Weight |  | UKB Neale v2 | 2018-08-01 | 360116 | rs3759094 | 3E-15 | -0.2771 |
| NEALE2_23098_raw | Weight |  | UKB Neale v2 | 2018-08-01 | 354838 | rs3759094 | 7.3E-14 | -0.2638 |
| NEALE2_23107_raw | Impedance of leg (right) |  | UKB Neale v2 | 2018-08-01 | 354817 | rs4759228 | 5.6E-14 | 0.6302 |
| NEALE2_23118_raw | Leg predicted mass (left) |  | UKB Neale v2 | 2018-08-01 | 354766 | rs4759228 | 1.7E-14 | -0.0226 |
| NEALE2_23129_raw | Trunk fat-free mass |  | UKB Neale v2 | 2018-08-01 | 354530 | rs4759228 | 1.7E-14 | -0.0643 |
| NEALE2_23130_raw | Trunk predicted mass |  | UKB Neale v2 | 2018-08-01 | 354494 | rs4759228 | 1.4E-14 | -0.0619 |
| FINNGEN_R6_M13_ARTHROPATHIES | Arthropathies |  | FINNGEN_R6 | 2022-01-24 | 260405 | rs201829738 | 1.28E-10 |  |
| FINNGEN_R6_M13_ARTHROSIS | Arthrosis |  | FINNGEN_R6 | 2022-01-24 | 218881 | rs201829738 | 7.59E-10 |  |
| FINNGEN_R6_M13_ARTHROSIS_KNEE | Gonarthrosis |  | FINNGEN_R6 | 2022-01-24 | 201213 | rs201829738 | 1.74E-09 |  |
| FINNGEN_R6_M13_MUSCULOSKELETAL | Diseases of the musculoskeletal system and connective tissue |  | FINNGEN_R6 | 2022-01-24 | 260405 | rs201829738 | 2.24E-09 |  |
| FINNGEN_R6_M13_OTHERJOINT | Other joint disorders |  | FINNGEN_R6 | 2022-01-24 | 226692 | rs201829738 | 7.42E-10 |  |
| NEALE2_23100_raw | Whole body fat mass |  | UKB Neale v2 | 2018-08-01 | 354244 | rs2292238 | 1.12444E-10 | -0.1436 |
| NEALE2_23102_raw | Whole body water mass |  | UKB Neale v2 | 2018-08-01 | 354834 | rs4759228 | 8.26128E-16 | -0.0963 |
| NEALE2_23105_raw | Basal metabolic rate |  | UKB Neale v2 | 2018-08-01 | 354825 | rs4759228 | 1E-15 | -17.461 |
| NEALE2_23117_raw | Leg fat-free mass (left) |  | UKB Neale v2 | 2018-08-01 | 354771 | rs4759228 | 1.4E-14 | -0.0242 |
| NEALE2_21001_raw | Body mass index (bmi) |  | UKB Neale v2 | 2018-08-01 | 359983 | rs2292238 | 1E-15 | -0.0908 |
| NEALE2_23104_raw | Body mass index (bmi) |  | UKB Neale v2 | 2018-08-01 | 354831 | rs2292238 | 4E-15 | -0.0896 |
| NEALE2_23120_raw | Arm fat mass (right) |  | UKB Neale v2 | 2018-08-01 | 354736 | rs2292238 | 1.0547E-11 | -0.0101 |
| GCST007039 | Body mass index | PMID:30595370 | Kichaev G | 2018-12-27 | 458000 | rs4759228 | 1E-16 |  |
| GCST009871 | Body mass index | PMID:31669095 | Zhu Z | 2019-10-24 | 457822 | rs3759094 | 9E-16 |  |
| GCST010988 | Adult body size | PMID:32376654 | Richardson TG | 2020-05-06 | 453169 | rs4759228 | 1E-14 |  |
| GCST90018947 | Body mass index | PMID:34594039 | Sakaue S | 2021-09-30 | 523818 | rs4759228 | 2E-15 |  |
| NEALE2_23106_raw | Impedance of whole body |  | UKB Neale v2 | 2018-08-01 | 354795 | rs4759228 | 2.30932E-17 | 1.46576 |
| NEALE2_23108_raw | Impedance of leg (left) |  | UKB Neale v2 | 2018-08-01 | 354811 | rs4759228 | 9E-15 | 0.64907 |
| GCST90018862 | Hypothyroidism | PMID:34594039 | Sakaue S | 2021-09-30 | 583911 | rs11171710 | 3E-12 | -0.0698 |
| GCST90018980 | Medication use (drugs for peptic ulcer and gastro-oesophageal reflux disease) | PMID:34594039 | Sakaue S | 2021-09-30 | 311093 | rs11611029 | 3E-08 | 0.0316 |
| NEALE2_23113_raw | Leg fat-free mass (right) |  | UKB Neale v2 | 2018-08-01 | 354798 | rs2069408 | 1E-15 | -0.0241 |
| NEALE2_23114_raw | Leg predicted mass (right) |  | UKB Neale v2 | 2018-08-01 | 354798 | rs2069408 | 2E-15 | -0.0225 |
| GCST90018855 | Hashimoto thyroiditis | PMID:34594039 | Sakaue S | 2021-09-30 | 568833 | rs11611029 | 1E-08 | -0.0958 |
| NEALE2_49_raw | Hip circumference |  | UKB Neale v2 | 2018-08-01 | 360521 | rs2069408 | 1.5962E-09 | -0.1372 |
| NEALE2_30010_raw | Red blood cell (erythrocyte) count |  | UKB Neale v2 | 2018-08-01 | 350475 | rs11425044 | 1.53377E-09 | -0.0052 |
| NEALE2_23124_raw | Arm fat mass (left) |  | UKB Neale v2 | 2018-08-01 | 354673 | rs2292238 | 2.9547E-11 | -0.011 |
| NEALE2_23126_raw | Arm predicted mass (left) |  | UKB Neale v2 | 2018-08-01 | 354653 | rs4759228 | 4.0168E-16 | -0.0094 |
| NEALE2_23112_raw | Leg fat mass (right) |  | UKB Neale v2 | 2018-08-01 | 354807 | rs2292238 | 2.668E-12 | -0.0264 |
| NEALE2_23116_raw | Leg fat mass (left) |  | UKB Neale v2 | 2018-08-01 | 354788 | rs1364324611 | 3.279E-12 | 0.02568 |
| GCST90020232 | Body fat percentage | PMID:33980691 | Martin S | 2021-05-12 | 442278 | rs2292238 | 5E-08 | -0.008 |
| GCST90020236 | Alanine aminotransferase levels | PMID:33980691 | Martin S | 2021-05-12 | 429203 | rs2292238 | 2E-08 | -0.011 |
| NEALE2_23101_raw | Whole body fat-free mass |  | UKB Neale v2 | 2018-08-01 | 354808 | rs4759228 | 9.17975E-16 | -0.1305 |
| GCST90020237 | Aspartate aminotransferase levels | PMID:33980691 | Martin S | 2021-05-12 | 424778 | rs2292238 | 1E-09 | -0.012 |
| GCST90038594 | Metabolic biomarkers (multivariate analysis) | PMID:33980691 | Martin S | 2021-05-12 | 389354 | rs2292238 | 1E-09 |  |
| GCST011623_3 | Odorant perception [Licorice intensity] | PMID:33035477 | Gisladottir RS | 2020-09-25 | 9122 | rs60683621 | 9E-16 |  |
| GCST011623_4 | Odorant perception [Licorice naming problem] | PMID:33035477 | Gisladottir RS | 2020-09-25 | 9122 | rs60683621 | 1E-09 |  |
| NEALE2_23099_raw | Body fat percentage |  | UKB Neale v2 | 2018-08-01 | 354628 | rs1364324611 | 6.52181E-09 | 0.08851 |
| NEALE2_23119_raw | Arm fat percentage (right) |  | UKB Neale v2 | 2018-08-01 | 354760 | rs2292238 | 2.63012E-10 | -0.1136 |
| NEALE2_23123_raw | Arm fat percentage (left) |  | UKB Neale v2 | 2018-08-01 | 354707 | rs2292238 | 6.88929E-10 | -0.1119 |
| NEALE2_23122_raw | Arm predicted mass (right) |  | UKB Neale v2 | 2018-08-01 | 354726 | rs2069408 | 1.1E-14 | -0.008 |
| NEALE2_23125_raw | Arm fat-free mass (left) |  | UKB Neale v2 | 2018-08-01 | 354668 | rs2069408 | 4.12617E-16 | -0.0096 |
| GCST009003 | Body mass index | PMID:30239722 | Pulit SL | 2018-09-14 | 434794 | rs10783779 | 5E-15 | -0.0137 |
| GCST009004 | Body mass index | PMID:30239722 | Pulit SL | 2018-09-14 | 806834 | rs10783779 | 5E-15 | -0.0137 |
| NEALE2_23111_raw | Leg fat percentage (right) |  | UKB Neale v2 | 2018-08-01 | 354811 | rs1364324611 | 2.28033E-10 | 0.08415 |
| NEALE2_23115_raw | Leg fat percentage (left) |  | UKB Neale v2 | 2018-08-01 | 354791 | rs1364324611 | 4.2185E-11 | 0.08321 |
| NEALE2_23109_raw | Impedance of arm (right) |  | UKB Neale v2 | 2018-08-01 | 354792 | rs4759228 | 3.2E-14 | 0.75076 |
| NEALE2_2744 | Birth weight of first child |  | UKB Neale v2 | 2018-08-01 | 155202 | rs4332553 | 2.66581E-09 | -0.017 |
| GCST004785 | Vitiligo | PMID:27723757 | Jin Y | 2016-11-01 | 40258 | rs2017445 | 7E-31 |  |
| GCST90025980 | Aspartate aminotransferase levels | PMID:34226706 | Barton AR | 2021-07-05 | 436275 | rs1131017 | 7E-17 | 0.01704 |
| GCST90095129 | Vertex-wise sulcal depth | PMID:34910505 | van der Meer D | 2021-12-15 | 33748 | rs11171739 | 2E-33 | -12.04 |
| NEALE2_23110_raw | Impedance of arm (left) |  | UKB Neale v2 | 2018-08-01 | 354807 | rs4759228 | 1.1E-14 | 0.78513 |
| NEALE2_23121_raw | Arm fat-free mass (right) |  | UKB Neale v2 | 2018-08-01 | 354732 | rs2069408 | 1E-14 | -0.0085 |
| GCST007800 | Asthma (childhood onset) | PMID:30929738 | Ferreira MAR | 2019-03-28 | 314633 | rs705700 | 3E-24 |  |
| GCST007069 | Red blood cell count | PMID:30595370 | Kichaev G | 2018-12-27 | 445000 | rs11171731 | 1E-08 |  |
| GCST008377 | Type 1 diabetes | PMID:31152121 | Zhu M | 2019-05-31 | 2262 | rs705699 | 7E-20 |  |
| GCST006585_1384 | Blood protein levels [PMEL, 6472_40_3] | PMID:30072576 | Emilsson V | 2018-08-02 | 3200 | rs3213122 | 2E-13 | 0.30151 |
| GCST90089451 | Serum levels of protein PMEL | PMID:35078996 | Gudjonsson A | 2022-01-25 | 5363 | rs12309895 | 4E-22 |  |
| GCST008916 | Asthma | PMID:31619474 | Zhu Z | 2019-10-17 | 394283 | rs705700 | 1E-16 |  |
| GCST007065 | Eosinophil counts | PMID:30595370 | Kichaev G | 2018-12-27 | 440000 | rs773110 | 4E-31 |  |
| GCST001634 | Polycystic ovary syndrome | PMID:22885925 | Shi Y | 2012-08-12 | 5255 | rs705702 | 9E-26 |  |
| NEALE2_2139_raw | Age first had sexual intercourse |  | UKB Neale v2 | 2018-08-01 | 317694 | rs7297175 | 6.7123E-11 | -0.0621 |
| GCST009841 | Asthma (childhood onset) | PMID:31669095 | Zhu Z | 2019-10-24 | 406621 | rs705699 | 9E-17 |  |
| GCST009850 | Atopic asthma | PMID:31669095 | Zhu Z | 2019-10-24 | 417151 | rs705699 | 4E-16 |  |
| GCST90018943 | Alanine aminotransferase levels | PMID:34594039 | Sakaue S | 2021-09-30 | 494681 | rs1873914 | 3E-14 |  |
| GCST006573 | Self-reported math ability | PMID:30038396 | Lee JJ | 2018-07-23 | 564698 | rs11171739 | 1E-10 | -0.0128 |
| GCST010703 | Brain morphology (MOSTest) | PMID:32665545 | van der Meer D | 2020-07-14 | 26502 | rs11171739 | 4E-10 |  |
| GCST006569 | Self-reported math ability (MTAG) [MTAG] | PMID:30038396 | Lee JJ | 2018-07-23 | 670471 | rs1131017 | 5E-19 |  |
| GCST010571 | Autoimmune thyroid disease | PMID:32581359 | Saevarsdottir S | 2020-06-24 | 755406 | rs2271194 | 2E-11 |  |
| GCST009524 | Household income (MTAG) | PMID:31844048 | Hill WD | 2019-12-16 | 505541 | rs10876864 | 8E-14 | -0.0152 |
| GCST005316 | Intelligence (MTAG) | PMID:29326435 | Hill WD | 2018-01-11 | 248482 | rs10876864 | 2E-12 | -0.0208 |
| GCST90018953 | Eosinophil counts | PMID:34594039 | Sakaue S | 2021-09-30 | 442919 | rs10876864 | 2E-18 | -0.0148 |
| GCST008832 | Gastroesophageal reflux disease | PMID:31527586 | An J | 2019-09-16 | 385276 | rs11171710 | 1E-08 |  |
| GCST011520 | Insulin-like growth factor 1 levels | PMID:33587031 | Sinnott-Armstrong N | 2021-02-15 | 317114 | rs11171710 | 7E-09 | 0.00388 |
| GCST007564 | Asthma or allergic disease (pleiotropy) | PMID:29785011 | Zhu Z | 2018-05-21 | 116538 | rs10876864 | 1E-13 |  |
| GCST90095130 | Vertex-wise cortical surface area | PMID:34910505 | van der Meer D | 2021-12-15 | 33748 | rs1131017 | 5E-20 |  |
| GCST90002320 | Lymphocyte counts | PMID:32888493 | Chen MH | 2020-09-01 | 643370 | rs1131017 | 4E-10 |  |
| GCST010242 | HDL cholesterol levels | PMID:32203549 | Richardson TG | 2020-03-23 | 403943 | rs11171710 | 4E-09 | -0.0114 |
| GCST007325 | General risk tolerance (MTAG) | PMID:30643258 | Karlsson Linner R | 2019-01-14 | 975353 | rs59626664 | 2E-09 |  |
| GCST009873 | Autoimmune traits (pleiotropy) | PMID:30572963 | Marquez A | 2018-12-20 | 59467 | rs11171739 | 2E-20 |  |
| GCST011156 | Body mass index | PMID:32700739 | Salinas YD | 2020-07-23 | 305945 | rs705708 | 1E-08 | -0.065 |
| GCST006585_105 | Blood protein levels [APOF, 12370_30_3] | PMID:30072576 | Emilsson V | 2018-08-02 | 3200 | rs808919 | 7E-16 |  |
| GCST006570 | Cognitive performance (MTAG) [MTAG] | PMID:30038396 | Lee JJ | 2018-07-23 | 402382 | rs1131017 | 6E-23 |  |
| NEALE2_48_raw | Waist circumference |  | UKB Neale v2 | 2018-08-01 | 360564 | rs4759228 | 7.0616E-11 | -0.2002 |
| GCST90025979 | Alanine aminotransferase levels | PMID:34226706 | Barton AR | 2021-07-05 | 437724 | rs1131017 | 4.1E-10 | 0.01329 |
| FINNGEN_R6_DM_KETOACIDOSIS | Diabetic ketoacidosis |  | FINNGEN_R6 | 2022-01-24 | 196542 | rs2069408 | 9.57E-10 |  |
| GCST90000026 | Appendicular lean mass | PMID:33097823 | Pei YF | 2020-10-23 | 205513 | rs59626664 | 3.6E-12 | 0.03825 |
| FINNGEN_R6_AUTOIMMUNE | Autoimmune diseases |  | FINNGEN_R6 | 2022-01-24 | 260405 | rs2069408 | 6.29E-11 |  |
| GCST007995 | Asthma (childhood onset) | PMID:31036433 | Pividori M | 2019-04-26 | 327670 | rs705699 | 1E-11 |  |
| GCST90011899 | Aspartate aminotransferase levels | PMID:33547301 | Chen VL | 2021-02-05 | 389565 | rs705699 | 8E-11 | -6.5 |
| NEALE2_3143_raw | Ankle spacing width |  | UKB Neale v2 | 2018-08-01 | 206589 | rs4759228 | 1.1734E-11 | -0.0913 |
| GCST007563 | Allergic disease (asthma, hay fever or eczema) | PMID:29785011 | Zhu Z | 2018-05-21 | 102453 | rs11171739 | 4E-10 |  |
| GCST010282_34 | Cortical surface area [total] | PMID:32193296 | Grasby KL | 2020-03-01 | 33992 | rs11171739 | 4E-11 | -526.55 |
| GCST009842 | Asthma (adult onset) | PMID:31669095 | Zhu Z | 2019-10-24 | 426604 | rs705700 | 1E-09 |  |
| GCST90025995 | Serum total protein level | PMID:34226706 | Barton AR | 2021-07-05 | 400482 | rs1131017 | 1.7E-09 | 0.01282 |
| GCST002318_2 | Rheumatoid arthritis [EA] | PMID:24390342 | Okada Y | 2013-12-25 | 80799 | rs773125 | 2E-08 |  |
| GCST009845 | Nonatopic asthma | PMID:31669095 | Zhu Z | 2019-10-24 | 450910 | rs705700 | 2E-09 |  |
| GCST010985 | Allergic disease (asthma, hay fever and/or eczema) (age of onset) | PMID:32603359 | Ferreira MAR | 2020-06-30 | 117130 | rs705699 | 4E-09 | -0.025 |
| GCST006911 | Asthma (moderate or severe) | PMID:30552067 | Shrine N | 2018-12-11 | 30810 | rs7305461 | 1E-09 |  |
| GCST000043 | Type 1 diabetes | PMID:17554300 | Wellcome Trust Case Control Consortium | 2007-06-07 | 4901 | rs11171739 | 1E-11 |  |
| GCST007922 | Medication use (drugs for peptic ulcer and gastro-oesophageal reflux disease) | PMID:31015401 | Wu Y | 2019-04-23 | 132367 | rs11171710 | 3E-09 | 0.04655 |
| GCST002318 | Rheumatoid arthritis | PMID:24390342 | Okada Y | 2013-12-25 | 80799 | rs773125 | 1E-10 |  |
| GCST006959 | Rheumatoid arthritis | PMID:30423114 | Laufer VA | 2018-11-13 | 2308 | rs773125 | 1E-09 | -0.0859 |
| FINNGEN_R6_T1D | Type1 diabetes | definitions combined" |  | FINNGEN_R6 | 2022-01-24 | 12_55991020_G_A | rs705699 | ####### |
| GCST009875 | Type 1 diabetes | PMID:30572963 | Marquez A | 2018-12-20 | 28978 | rs11171739 | 1E-18 |  |
| FINNGEN_R6_T1D_STRICT | Type 1 diabetes | strict definition" |  | FINNGEN_R6 | 2022-01-24 | 12_55991020_G_A | rs705699 | ####### |
| FINNGEN_R6_E4_DM1_STRICT | Type 1 diabetes | strict (exclude DM2)" |  | FINNGEN_R6 | 2022-01-24 | 12_55991020_G_A | rs705699 | ####### |
| GCST010984 | Allergic disease (asthma, hay fever and/or eczema) (multivariate analysis) | PMID:32603359 | Ferreira MAR | 2020-06-30 | 477968 | rs705699 | 6E-09 | -0.024 |
| GCST011500 | Gastroesophageal reflux disease, peptic ulcer disease and/or corresponding medications and treatment | PMID:33608531 | Wu Y | 2021-02-19 | 456327 | rs1873914 | 5E-08 |  |
| GCST90091060 | Cortical surface area | PMID:34560273 | Shadrin AA | 2021-09-21 | 35657 | rs1131017 | 2E-10 |  |
| GCST010084 | Leisure sedentary behaviour (television watching) | PMID:32317632 | van de Vegte YJ | 2020-04-21 | 408815 | rs10876864 | 1E-09 |  |
| NEALE2_20151_raw | Forced vital capacity (fvc), best measure |  | UKB Neale v2 | 2018-08-01 | 272338 | rs59822547 | 6.15E-13 | 0.02681 |
| GCST007089 | Polycystic ovary syndrome | PMID:30566500 | Day F | 2018-12-19 | 113238 | rs2271194 | 5E-09 |  |
| GCST90011298 | Verbal-numerical reasoning | PMID:32895543 | de la Fuente J | 2020-09-07 | 171304 | rs11171739 | 3E-08 |  |
| GCST90000025 | Appendicular lean mass | PMID:33097823 | Pei YF | 2020-10-23 | 450243 | rs10876863 | 2.06E-13 | -0.0145 |
| GCST009577 | Appendicular lean mass | PMID:31761296 | Hernandez Cordero AI | 2019-11-21 | 181862 | rs10876864 | 6E-09 | 0.04037 |
| GCST90011875 | Cognitive aspects of educational attainment | PMID:33414549 | Demange PA | 2021-01-07 | 257700 | rs10876864 | 7E-09 | -0.0369 |
| NEALE2_738 | Average total household income before tax |  | UKB Neale v2 | 2018-08-01 | 311028 | rs7305461 | 2.12092E-08 | -0.0161 |
| GCST009096 | Lifetime smoking index | PMID:31689377 | Wootton RE | 2019-11-06 | 462690 | rs7297175 | 7E-09 | 0.012 |
| NEALE2_23128_raw | Trunk fat mass |  | UKB Neale v2 | 2018-08-01 | 354597 | rs3759094 | 1.27788E-08 | -0.0734 |
| FINNGEN_R6_E4_DM1OPTH | Type 1 diabetes with ophthalmic complications |  | FINNGEN_R6 | 2022-01-24 | 218828 | rs705700 | 1.26E-11 |  |
| GCST010681 | Type 1 diabetes | PMID:32005708 | Forgetta V | 2020-01-31 | 24840 | rs1131017 | 4.242E-25 |  |
| GCST90018976 | Serum total protein level | PMID:34594039 | Sakaue S | 2021-09-30 | 448242 | rs1131017 | 6E-12 |  |
| GCST90002363 | Red blood cell count | PMID:32888493 | Chen MH | 2020-09-01 | 545203 |  | 1.52E-08 | 0.01111 |
| GCST004617 | Eosinophil percentage of granulocytes | PMID:27863252 | Astle WJ | 2016-11-17 | 170536 | rs10876864 | 1.33E-11 | -0.0244 |
| NEALE2_845 | Age completed full time education |  | UKB Neale v2 | 2018-08-01 | 240547 | rs10876870 | 1.38307E-08 | -0.0129 |
| GCST004623 | Neutrophil percentage of granulocytes | PMID:27863252 | Astle WJ | 2016-11-17 | 170672 | rs10876864 | 7.084E-11 | 0.0235 |
| NEALE2_6152_9 | Hayfever, allergic rhinitis or eczema | blood clot, dvt, bronchitis, emphysema, asthma, rhinitis, eczema, allergy diagnosed by doctor |  | UKB Neale v2 | 2018-08-01 | 360527 | rs11171739 | 1.48709E-18 |  |
| GCST006442_2 | Educational attainment (years of education) [conditional-joint] | PMID:30038396 | Lee JJ | 2018-07-23 | 1131881 | rs3741499 | 5E-35 | -0.0184 |
| GCST90002316 | Lymphocyte counts | PMID:32888493 | Chen MH | 2020-09-01 | 524923 | rs1131017 | 9.76E-13 | 0.01364 |
| NEALE2_20002_1226 | Hypothyroidism/myxoedema | non-cancer illness code, self-reported |  | UKB Neale v2 | 2018-08-01 | 361141 | rs1398310988 | 1.4988E-11 |  |
| GCST90000514 | Gastroesophageal reflux disease | PMID:34187846 | Ong JS | 2021-06-29 | 602604 | rs773109 | 8.7E-14 |  |
| NEALE2_1070 | Time spent watching television (tv) |  | UKB Neale v2 | 2018-08-01 | 341859 | rs11611029 | 1.18052E-08 | 0.0103 |
| GCST90027161 | Atopic dermatitis | PMID:34454985 | Sliz E | 2021-08-26 | 796661 | rs4759228 | 4.611E-08 |  |
| GCST90013405 | Liver enzyme levels (alanine transaminase) | PMID:33972514 | Pazoki R | 2021-05-10 | 437267 | rs1131017 | 2.9E-11 | 0.00262 |
| NEALE2_3761_raw | Age hay fever, rhinitis or eczema diagnosed |  | UKB Neale v2 | 2018-08-01 | 72232 | rs1131017 | 8.76996E-09 | 0.46806 |
| GCST006572 | Cognitive performance | PMID:30038396 | Lee JJ | 2018-07-23 | 257841 | rs1131017 | 3.85E-09 | -0.017 |
| GCST001191 | Type 1 diabetes | PMID:21829393 | Plagnol V | 2011-08-04 | 19102 | rs2292239 | 3E-27 |  |
| GCST90086149 | Atopic dermatitis | PMID:34116867 | Tanaka N | 2021-06-02 | 215554 | rs11171739 | 6E-10 |  |
| GCST000392 | Type 1 diabetes | PMID:19430480 | Barrett JC | 2009-05-10 | 16559 | rs2292239 | 2E-25 |  |
| GCST009868 | Adult onset asthma and/or BMI | PMID:31669095 | Zhu Z | 2019-10-24 | 457822 | rs4759229 | 2E-23 |  |
| FINNGEN_R6_T1D_WIDE | Type 1 diabetes | wide definition" |  | FINNGEN_R6 | 2022-01-24 | 12_55976127_A_G | rs773108 | 2.2E-11 |
| GCST007073 | Hypothyroidism | PMID:30595370 | Kichaev G | 2018-12-27 | 459000 | rs772920 | 7E-15 |  |
| GCST007071 | Autoimmune traits | PMID:30595370 | Kichaev G | 2018-12-27 | 459000 | rs773107 | 1E-09 |  |
| SAIGE_244_4 | Hypothyroidism nos |  | UKB SAIGE | 2018-10-24 | 405600 | rs1131017 | 3.74E-08 |  |
| NEALE2_6152_100 | None of the above | blood clot, dvt, bronchitis, emphysema, asthma, rhinitis, eczema, allergy diagnosed by doctor |  | UKB Neale v2 | 2018-08-01 | 360527 | rs1131017 | 1.12234E-22 |  |
| GCST006481 | Lung function (FEV1) | PMID:30061609 | Wyss AB | 2018-07-30 | 90715 | rs772920 | 2E-08 |  |
| GCST90038599 | Diabetes or endocrine disease | PMID:33959723 | Donertas HM | 2021-04-08 | 484598 | rs7305461 | 2.5E-11 |  |
| FINNGEN_R6_J10_NASALPOLYP | Nasal polyp |  | FINNGEN_R6 | 2022-01-24 | 203127 | rs705702 | 3.67E-08 |  |
| GCST90000059 | Diastolic blood pressure | PMID:33230300 | Surendran P | 2020-11-23 | 810865 | rs2292239 | 6.441E-11 | 0.01083 |
| GCST90025989 | Insulin-like growth factor 1 levels | PMID:34226706 | Barton AR | 2021-07-05 | 435516 | rs10876864 | 1E-14 | 0.01509 |
| FINNGEN_R6_E4_DM1NOCOMP | Type 1 diabetes without complications |  | FINNGEN_R6 | 2022-01-24 | 221536 | rs705702 | 2.22E-13 |  |
| GCST90002388 | Lymphocyte counts | PMID:32888494 | Vuckovic D | 2020-09-01 | 408112 | rs1131017 | 3.6E-11 | 0.01415 |
| FINNGEN_R6_E4_DM1 | Type 1 diabetes |  | FINNGEN_R6 | 2022-01-24 | 222769 | rs705702 | 1.9E-11 |  |
| GCST90025984 | Lymphocyte counts | PMID:34226706 | Barton AR | 2021-07-05 | 443762 | rs1131017 | 3.8E-09 | 0.01173 |
| GCST001670 | Vitiligo | PMID:22951725 | Tang XF | 2012-09-06 | 2818 | rs10876864 | 8E-12 |  |
| GCST004624 | Sum eosinophil basophil counts | PMID:27863252 | Astle WJ | 2016-11-17 | 171771 | rs772920 | 5.331E-12 | 0.02597 |
| NEALE2_6138_1 | College or university degree | qualifications |  | UKB Neale v2 | 2018-08-01 | 357549 | rs7305461 | 1.34717E-10 |  |
| GCST009518_82 | Brain region volumes [total brain volume] | PMID:31676860 | Zhao B | 2019-11-01 | 19629 | rs7955865 | 3E-08 |  |
| GCST009866 | Nonatopic asthma and/or BMI | PMID:31669095 | Zhu Z | 2019-10-24 | 457822 | rs4759229 | 2E-21 |  |
| GCST004866 | Alopecia areata | PMID:25608926 | Betz RC | 2015-01-22 | 7565 | rs2292239 | 4E-09 |  |
| GCST007603 | Smoking initiation | PMID:30617275 | Erzurumluoglu AM | 2019-01-07 | 346813 | rs2292239 | 3E-08 | 0.0121 |
| GCST90011874 | Noncognitive aspects of educational attainment | PMID:33414549 | Demange PA | 2021-01-07 | 510795 | rs705696 | 8E-09 | 0.04284 |
| SAIGE_471 | Nasal polyps |  | UKB SAIGE | 2018-10-24 | 393356 | rs705702 | 3.74E-09 |  |
| GCST000038 | Type 1 diabetes | PMID:17554260 | Todd JA | 2007-06-06 | 5000 | rs2292239 | 2E-20 |  |
| GCST000258 | Type 1 diabetes | PMID:18978792 | Cooper JD | 2008-11-02 | 8207 | rs2292239 | 3E-16 |  |
| GCST90002389 | Lymphocyte percentage of white cells | PMID:32888494 | Vuckovic D | 2020-09-01 | 408112 | rs1131017 | 2.7E-09 | 0.01294 |
| GCST90095131 | Vertex-wise cortical thickness | PMID:34910505 | van der Meer D | 2021-12-15 | 33748 | rs7955865 | 7E-12 |  |
| GCST007468 | Smoking initiation (ever regular vs never regular) (MTAG) | PMID:30643251 | Liu M | 2019-01-14 | 1359002 | rs3741499 | 4E-13 | 0.00824 |
| GCST90000046 | Age at first sexual intercourse | PMID:34211149 | Mills MC | 2021-07-01 | 182791 | rs7297175 | 3.3E-09 | -0.0183 |
| NEALE2_3063_raw | Forced expiratory volume in 1-second (fev1) |  | UKB Neale v2 | 2018-08-01 | 329404 | rs772920 | 6.64003E-09 | -0.0087 |
| GCST011703 | Smoking initiation | PMID:33082346 | Xu K | 2020-10-20 | 842717 | rs3741499 | 2E-08 |  |
| GCST90091061 | Cortical thickness | PMID:34560273 | Shadrin AA | 2021-09-21 | 35657 | rs4759229 | 4E-08 |  |
| GCST90025987 | Eosinophil counts | PMID:34226706 | Barton AR | 2021-07-05 | 440275 | rs705702 | 1.6E-41 | 0.0272 |
| GCST007474 | Smoking initiation (ever regular vs never regular) | PMID:30643251 | Liu M | 2019-01-14 | 1232091 | rs4759229 | 7E-09 | 0.01557 |
| NEALE2_20002_1382 | Sjogren's syndrome/sicca syndrome | non-cancer illness code, self-reported |  | UKB Neale v2 | 2018-08-01 | 361141 | rs149449770 | 4.47855E-08 |  |
| NEALE2_5084_raw | Spherical power (right) |  | UKB Neale v2 | 2018-08-01 | 77983 | rs3138142 | 7.94975E-41 | 0.22025 |
| GCST90018911 | Retinal detachment | PMID:34594039 | Sakaue S | 2021-09-30 | 647323 | rs3138142 | 5E-08 | -0.1184 |
| SAIGE_367_1 | Myopia |  | UKB SAIGE | 2018-10-24 | 407787 | rs3138141 | 1.1E-09 |  |
| GCST012400 | Low myopia vs hyperopia | PMID:33830181 | Tideman JWL | 2021-04-08 | 22025 | rs3138142 | 4E-19 |  |
| GCST006289_2 | Spherical equivalent [EA] | PMID:29808027 | Tedja MS | 2018-05-28 | 66127 | rs3138144 | 5E-12 |  |
| NEALE2_2217_raw | Age started wearing glasses or contact lenses |  | UKB Neale v2 | 2018-08-01 | 310992 | rs3138142 | 2.46646E-26 | 0.52215 |
| GCST003219_2 | Advanced age-related macular degeneration [EA] | PMID:26691988 | Fritsche LG | 2015-12-21 | 33976 | rs3138141 | 4E-09 |  |
| GCST003997 | Myopia | PMID:27182965 | Pickrell JK | 2016-05-16 | 191843 | rs3138141 | 2E-51 |  |
| GCST006290 | Myopia (age of diagnosis) | PMID:29808027 | Tedja MS | 2018-05-28 | 104293 | rs3138141 | 4E-42 | 0.1201 |
| GCST006291 | Spherical equivalent or myopia (age of diagnosis) | PMID:29808027 | Tedja MS | 2018-05-28 | 170420 | rs3138141 | 2E-43 | 13.803 |
| GCST006976 | Macular thickness | PMID:30535121 | Gao XR | 2018-12-07 | 59814 | rs3138142 | 9E-105 |  |
| GCST010378 | Spherical equivalent | PMID:32352494 | Han X | 2020-04-30 | 95827 | rs3138142 | 2E-57 | 0.21 |
| NEALE2_5085_raw | Spherical power (left) |  | UKB Neale v2 | 2018-08-01 | 77739 | rs3138142 | 3.53319E-40 | 0.2213 |
| GCST001858 | Refractive error | PMID:23396134 | Verhoeven VJ | 2013-02-10 | 45758 | rs3138144 | 4E-12 |  |
| GCST006289 | Spherical equivalent | PMID:29808027 | Tedja MS | 2018-05-28 | 66127 | rs3138144 | 8E-14 |  |
| GCST010002 | Refractive error | PMID:32231278 | Hysi PG | 2020-03-30 | 542934 | rs3138142 | 6E-174 |  |
| GCST003455 | Spherical equivalent (joint analysis main effects and education interaction) | PMID:27020472 | Fan Q | 2016-03-29 | 50351 | rs3138142 | 1E-08 |  |
| GCST012401 | Hyperopia | PMID:33830181 | Tideman JWL | 2021-04-08 | 32244 | rs3138142 | 2E-12 |  |
| GCST003455_3 | Spherical equivalent (joint analysis main effects and education interaction) [European ancestry] | PMID:27020472 | Fan Q | 2016-03-29 | 50351 | rs3138142 | 4E-08 |  |
| NEALE2_6138_100 | None of the above | qualifications |  | UKB Neale v2 | 2018-08-01 | 357549 | rs36159461 | 4.739E-12 |  |
| GCST009962 | High myopia | PMID:31816047 | Boutin TS | 2019-12-09 | 50372 | rs3138142 | 7E-12 |  |
| GCST012403 | High myopia | PMID:33830181 | Tideman JWL | 2021-04-08 | 24580 | rs3138142 | 7E-12 |  |
| GCST90002302 | Eosinophil counts | PMID:32888493 | Chen MH | 2020-09-01 | 583850 | rs1689510 | 9E-37 |  |
| NEALE2_20003_1141191044 | Levothyroxine sodium | treatment/medication code |  | UKB Neale v2 | 2018-08-01 | 361141 | rs772920 | 1.86285E-09 |  |
| GCST90038637 | Hypothyroidism or myxedema | PMID:33959723 | Donertas HM | 2021-04-08 | 484598 | rs772920 | 9.5E-17 |  |
| GCST90013679 | Asthma | PMID:32514122 | Ishigaki K | 2020-06-08 | 209808 | rs705704 | 7E-10 |  |
| GCST004166 | Nonsyndromic cleft lip with cleft palate | PMID:28232668 | Yu Y | 2017-02-24 | 6084 | rs705704 | 1E-09 |  |
| GCST90000529 | Type 1 diabetes | PMID:33830302 | Inshaw JRJ | 2021-04-08 | 17685 | rs4759229 | ############# |  |
| GCST000719 | Alopecia areata | PMID:20596022 | Petukhova L | 2010-07-01 | 4332 | rs1701704 | 3E-08 |  |
| GCST90018999 | Medication use (adrenergics, inhalants) | PMID:34594039 | Sakaue S | 2021-09-30 | 355171 | rs2640562 | 2E-21 | 0.0837 |
| NEALE2_20084_477 | Vitamin b12 | vitamin and/or mineral supplement use |  | UKB Neale v2 | 2018-08-01 | 51427 | rs147496211 | 2.30483E-08 |  |
| GCST90018575 | Asthma | PMID:34594039 | Sakaue S | 2021-09-30 | 175948 | rs1702877 | 1E-11 | 0.1172 |
| GCST005536 | Type 1 diabetes | PMID:25751624 | Onengut-Gumuscu S | 2015-04-01 | 29652 | rs1701704 | ############# | 0.22298 |
| GCST008810 | Smoking initiation (ever regular vs never regular) | PMID:30679032 | Brazel DM | 2018-12-06 | 433216 | rs1701704 | 3E-08 | -0.0126 |
| GCST006571 | Educational attainment (MTAG) [MTAG] | PMID:30038396 | Lee JJ | 2018-07-23 | 1131438 | rs1689510 | 3E-46 |  |
| GCST000141 | Type 1 diabetes | PMID:18198356 | Hakonarson H | 2008-01-15 | 3105 | rs1701704 | 9E-10 |  |
| GCST003097 | Pediatric autoimmune diseases | PMID:26301688 | Li YR | 2015-08-24 | 16753 | rs1689510 | 4E-09 |  |
| GCST90018990 | Medication use (thyroid preparations) | PMID:34594039 | Sakaue S | 2021-09-30 | 484308 | rs705704 | 9E-16 | 0.0742 |
| GCST90019000 | Medication use (glucocorticoids) | PMID:34594039 | Sakaue S | 2021-09-30 | 384426 | rs1689510 | 5E-14 |  |
| GCST009719 | Allergic rhinitis | PMID:31361310 | Johansson A | 2019-07-30 | 258688 | rs2640564 | 1E-09 |  |
| NEALE2_6147_1 | For short-sightedness, i.e. only or mainly for distance viewing such as driving, cinema etc (called 'myopia') | reason for glasses/contact lenses |  | UKB Neale v2 | 2018-08-01 | 360677 | rs3138142 | 5.522E-12 |  |
| GCST90018895 | Pediatric asthma | PMID:34594039 | Sakaue S | 2021-09-30 | 601193 | rs1702877 | 6E-10 | 0.0602 |
| GCST003677 | Educational attainment (college completion) | PMID:27225129 | Okbay A | 2016-05-26 | 280007 | rs2456973 | 2E-09 |  |
| GCST005141 | Cognitive ability (MTAG) | PMID:29186694 | Lam M | 2017-11-28 | 436124 | rs2456973 | 1E-08 |  |
| GCST007327 | Smoking status (ever vs never smokers) | PMID:30643258 | Karlsson Linner R | 2019-01-14 | 518633 | rs772921 | 4E-09 | -0.0125 |
| GCST007037 | Educational attainment (years of education) | PMID:30595370 | Kichaev G | 2018-12-27 | 455000 | rs1689510 | 8E-14 |  |
| GCST004785 | Vitiligo | PMID:27723757 | Jin Y | 2016-11-01 | 40258 | rs2017445 | 7E-31 |  |
| GCST011157 | Asthma | PMID:32700739 | Salinas YD | 2020-07-23 | 305945 | rs1701704 | 2E-14 |  |
| GCST006269 | General cognitive ability | PMID:29844566 | Davies G | 2018-05-29 | 300486 | rs1702877 | 6E-12 | 6.874 |
| GCST007462 | Age of smoking initiation (MTAG) | PMID:30643251 | Liu M | 2019-01-14 | 931815 | rs1701704 | 4E-11 | 0.0102 |
| GCST007066 | Heel bone mineral density | PMID:30595370 | Kichaev G | 2018-12-27 | 446000 | rs772921 | 9E-10 |  |
| GCST007942 | Medication use (glucocorticoids) | PMID:31015401 | Wu Y | 2019-04-23 | 205700 | rs1689510 | 1E-11 |  |
| GCST90061435 | Externalizing behaviour (multivariate analysis) | PMID:34446935 | Karlsson Linner R | 2021-08-26 | 1492085 | rs2640562 | 1E-12 | -0.009 |
| GCST001183 | Asthma | PMID:21804548 | Hirota T | 2011-07-31 | 4836 | rs1701704 | 2E-13 |  |
| GCST001191 | Type 1 diabetes | PMID:21829393 | Plagnol V | 2011-08-04 | 19102 | rs1701704 | 5E-18 |  |
| GCST009001 | Body mass index | PMID:30239722 | Pulit SL | 2018-09-14 | 374756 | rs705704 | 6E-15 | -0.0137 |
| GCST007076 | Respiratory diseases | PMID:30595370 | Kichaev G | 2018-12-27 | 459000 | rs1689510 | 2E-19 |  |
| GCST90018883 | Nasal polyps | PMID:34594039 | Sakaue S | 2021-09-30 | 602065 | rs10876866 | 9E-10 | 0.1326 |
| GCST001509 | Vitiligo | PMID:22561518 | Jin Y | 2012-05-06 | 3228 | rs2456973 | 3E-14 |  |
| GCST007080 | Lung function (FEV1/FVC) | PMID:30595370 | Kichaev G | 2018-12-27 | 370000 | rs1701704 | 1E-19 |  |
| GCST009798 | Asthma | PMID:31959851 | Olafsdottir TA | 2020-01-20 | 787635 | rs10876866 | 1E-15 |  |
| GCST006568 | Highest math class taken (MTAG) [MTAG] | PMID:30038396 | Lee JJ | 2018-07-23 | 811539 | rs2640564 | 4E-37 | 0.0223 |
| GCST90018795 | Asthma | PMID:34594039 | Sakaue S | 2021-09-30 | 625448 | rs1702877 | 3E-18 | 0.0663 |
| GCST009717 | Hay fever and/or eczema | PMID:31361310 | Johansson A | 2019-07-30 | 323807 | rs1689510 | 2E-19 |  |
| GCST009720_2 | Asthma [conditional] | PMID:31361310 | Johansson A | 2019-07-30 | 281699 | rs1689510 | 3E-20 |  |
| GCST90018925 | Type 1 diabetes | PMID:34594039 | Sakaue S | 2021-09-30 | 590946 | rs10876866 | 3E-08 | 0.1105 |
| GCST007932 | Medication use (thyroid preparations) | PMID:31015401 | Wu Y | 2019-04-23 | 305582 | rs7302200 | 1E-14 | 0.07457 |
| GCST008595 | Cognitive ability, years of educational attainment or schizophrenia (pleiotropy) | PMID:31374203 | Lam M | 2019-08-01 | 556676 | rs7302200 | 1E-14 |  |
| GCST003676 | Educational attainment (years of education) | PMID:27225129 | Okbay A | 2016-05-26 | 405072 | rs2456973 | 6E-16 | 0.01907 |
| GCST007799 | Asthma (adult onset) | PMID:30929738 | Ferreira MAR | 2019-03-28 | 327253 | rs7302200 | 4E-15 |  |
| GCST010043 | Asthma | PMID:32296059 | Han Y | 2020-04-15 | 536345 | rs1689510 | 2E-31 |  |
| GCST007941 | Medication use (adrenergics, inhalants) | PMID:31015401 | Wu Y | 2019-04-23 | 176445 | rs34415530 | 5E-17 | 0.07985 |
| GCST006442 | Educational attainment (years of education) | PMID:30038396 | Lee JJ | 2018-07-23 | 1131881 | rs1689510 | 1E-38 |  |
| GCST007798 | Asthma | PMID:30929738 | Ferreira MAR | 2019-03-28 | 341215 | rs7302200 | 8E-25 |  |
| GCST006574 | Highest math class taken | PMID:30038396 | Lee JJ | 2018-07-23 | 430445 | rs10876866 | 9E-20 | 0.022 |
| GCST007075 | Eczema | PMID:30595370 | Kichaev G | 2018-12-27 | 459000 | rs61938962 | 6E-23 |  |
| GCST90000047 | Age at first sexual intercourse | PMID:34211149 | Mills MC | 2021-07-01 | 397338 | rs7955865 | 8.4E-10 | -0.0134 |
| NEALE2_5832_2 | Left eye | which eye(s) affected by hypermetropia (long sight) |  | UKB Neale v2 | 2018-08-01 | 15090 | rs149676330 | 4.91084E-08 |  |
| GCST005519 | Heart rate response to beta blockers [AA] | PMID:29478026 | Shahin MH | 2018-02-24 | 699 | rs17117817 | 2E-09 | -5.53 |
| FINNGEN_R6_E4_HYTHYNAS | Hypothyroidism | other/unspecified" |  | FINNGEN_R6 | 2022-01-24 | 12_56053020_C_T | rs61938963 | ####### |
| NEALE2_30180_raw | Lymphocyte percentage |  | UKB Neale v2 | 2018-08-01 | 349861 |  | 4.45411E-10 | -0.1145 |
| GCST90014023 | Type 1 diabetes | PMID:34012112 | Chiou J | 2021-05-19 | 520580 | rs1701704 | 4.52E-63 |  |
| GCST90013791 | Type 1 diabetes |  | Crouch D | 2021-02-22 | 7977 | rs705704 | ############# |  |
| GCST90002381 | Eosinophil counts | PMID:32888494 | Vuckovic D | 2020-09-01 | 408112 | rs1689510 | 6.7E-34 | 0.02722 |
| GCST90025982 | Heel bone mineral density T score | PMID:34226706 | Barton AR | 2021-07-05 | 445855 | rs1701704 | 1E-08 | 0.01168 |
| GCST90038616 | Asthma | PMID:33959723 | Donertas HM | 2021-04-08 | 484598 | rs1689510 | 2.7E-23 |  |
| NEALE2_30150 | Eosinophill count |  | UKB Neale v2 | 2018-08-01 | 349856 | rs1689510 | 2.20508E-16 | 0.01525 |
| GCST005038 | Allergic disease (asthma, hay fever or eczema) | PMID:29083406 | Ferreira MA | 2017-10-30 | 360838 | rs1689510 | 3.388E-17 |  |
| GCST90038664 | Allergic rhinitis | PMID:33959723 | Donertas HM | 2021-04-08 | 484598 | rs34415530 | 5.5E-09 |  |
| NEALE2_30210_raw | Eosinophill percentage |  | UKB Neale v2 | 2018-08-01 | 349861 | rs1689510 | 1.18824E-31 | 0.05432 |
| GCST90038635 | Thyroid problem (not cancer) | PMID:33959723 | Donertas HM | 2021-04-08 | 484598 | rs7302200 | 1.3E-12 |  |
| GCST004606 | Eosinophil counts | PMID:27863252 | Astle WJ | 2016-11-17 | 172275 | rs7302200 | 2.96E-13 | 0.02746 |
| SAIGE_495 | Asthma |  | UKB SAIGE | 2018-10-24 | 401837 | rs1702877 | 3.37E-10 |  |
| NEALE2_6152_8 | Asthma | blood clot, dvt, bronchitis, emphysema, asthma, rhinitis, eczema, allergy diagnosed by doctor |  | UKB Neale v2 | 2018-08-01 | 360527 | rs1689510 | 8.26147E-18 |  |
| GCST007432 | FEV1 | PMID:30804560 | Shrine N | 2019-02-25 | 321047 | rs705704 | 3.941E-10 | -0.0155 |
| GCST90025978 | Lung function (FEV1/FVC) | PMID:34226706 | Barton AR | 2021-07-05 | 371898 | rs1701704 | 6.1E-20 | -0.0202 |
| NEALE2_6138_2 | A levels/as levels or equivalent | qualifications |  | UKB Neale v2 | 2018-08-01 | 357549 |  | 3.81625E-08 |  |
| NEALE2_20116_0 | Never | smoking status |  | UKB Neale v2 | 2018-08-01 | 359706 | rs772921 | 2.74073E-09 |  |
| GCST90000048 | Age at first birth | PMID:34211149 | Mills MC | 2021-07-01 | 418758 | rs1702877 | 3.898E-09 | 0.0628 |
| GCST90014325 | Asthma | PMID:34103634 | Valette K | 2021-06-08 | 408442 | rs1689510 | ############# |  |
| NEALE2_20002_1111 | Asthma | non-cancer illness code, self-reported |  | UKB Neale v2 | 2018-08-01 | 361141 | rs1689510 | 1.19737E-18 |  |
| GCST004600 | Eosinophil percentage of white cells | PMID:27863252 | Astle WJ | 2016-11-17 | 172378 | rs1689510 | 5E-14 | 0.02831 |
| FINNGEN_R6_E4_HYTHY_AI_STRICT | Hypothyroidism | strict autoimmune" |  | FINNGEN_R6 | 2022-01-24 | 12_56053020_C_T | rs61938963 | ####### |
| GCST90000050 | Age at first birth | PMID:34211149 | Mills MC | 2021-07-01 | 542901 | rs1702877 | 8.224E-10 | 0.0602 |
| NEALE2_2754_raw | Age at first live birth |  | UKB Neale v2 | 2018-08-01 | 131987 | rs2456973 | 3.89199E-08 | 0.10042 |
| GCST90038596 | Respiratory or ear-nose-throat disease | PMID:33959723 | Donertas HM | 2021-04-08 | 484598 | rs7302200 | 6.3E-18 |  |
| GCST90002298 | Eosinophil counts | PMID:32888493 | Chen MH | 2020-09-01 | 474237 | rs1689510 | 1.06E-36 | 0.02651 |
| GCST90002382 | Eosinophil percentage of white cells | PMID:32888494 | Vuckovic D | 2020-09-01 | 408112 | rs1689510 | 8.9E-39 | 0.02933 |
| NEALE2_20150_raw | Forced expiratory volume in 1-second (fev1), best measure |  | UKB Neale v2 | 2018-08-01 | 272338 | rs1689510 | 2.9686E-10 | -0.0101 |
| GCST007431 | Lung function (FEV1/FVC) | PMID:30804560 | Shrine N | 2019-02-25 | 321047 | rs61938962 | 7.04E-09 | -0.0146 |

**Supplementary Table S6. Associations with *GDF11* variants identified in the OpenTarget Genetics Portal.** A p-value equal to or less than 5*e*−8 was considered statistically significant.

| **Study ID** | **Trait** | **Trait Category** | **P-value** | **Beta** | **Odds Ratio** | **PMID** | **N Cases** | **N Overall** |
| --- | --- | --- | --- | --- | --- | --- | --- | --- |
| GCST90002394 | Monocyte percentage of white cells | measurement | 0.00011 | 0.00865518 |  | PMID:32888494 |  | 408112 |
| GCST90002388 | Lymphocyte counts | measurement | 2.00E-09 | -0.0134073 |  | PMID:32888494 |  | 408112 |
| GCST90002381 | Eosinophil counts | measurement | 6.70E-34 | 0.0272152 |  | PMID:32888494 |  | 408112 |
| FINNGEN_R6_DM_KETOACIDOSIS | Diabetic ketoacidosis | pancreas disease | 3.03E-09 | 0.13467 | 1.14415915 |  | 5706 | 196542 |
| FINNGEN_R6_T1D | Type1 diabetes, definitions combined | Uncategorised | 8.51E-08 | 0.15976 | 1.173229262 |  | 3440 | 217748 |
| GCST004632 | Lymphocyte percentage of white cells | measurement | 0.00002012 | -0.01602103 |  | PMID:27863252 |  | 171748 |
| NEALE2_2644 | Light smokers, at least 100 smokes in lifetime | Uncategorised | 6.61E-06 | -0.04287384 | 0.958032247 |  | 44253 | 97021 |
| NEALE2_2257 | Hearing difficulty/problems with background noise | phenotype | 0.00223414 | -0.015598375 | 0.98452265 |  | 134141 | 353983 |
| FINNGEN_R6_C3_BLADDER | Malignant neoplasm of bladder | cell proliferation disorder | 0.00408 | -0.10709 | 0.898444809 |  | 1701 | 260405 |
| NEALE2_20002_1065 | Hypertension | non-cancer illness code, self-reported | cardiovascular disease | 2.71E-06 | -0.025848382 | 0.974482828 |  | 93560 | 361141 |
| NEALE2_20003_1140865634 | Omeprazole | treatment/medication code | Uncategorised | 0.000450798 | -0.036983549 | 0.963691989 |  | 21119 | 361141 |
| NEALE2_1618 | Alcohol usually taken with meals | measurement | 0.00074868 | 0.024107675 | 1.024400615 |  | 59552 | 184716 |
| NEALE2_20002_1138 | Gastro-oesophageal reflux (gord) / gastric reflux | non-cancer illness code, self-reported | gastrointestinal disease | 0.00166407 | -0.038864763 | 0.961880782 |  | 15210 | 361141 |
| NEALE2_6152_9 | Hayfever, allergic rhinitis or eczema | blood clot, dvt, bronchitis, emphysema, asthma, rhinitis, eczema, allergy diagnosed by doctor | respiratory or thoracic disease | 7.13E-17 | 0.048898977 | 1.05011426 |  | 83407 | 360527 |
| NEALE2_20002_1453 | Psoriasis | non-cancer illness code, self-reported | integumentary system disease | 0.00498163 | 0.06518107 | 1.067352272 |  | 4192 | 361141 |
| NEALE2_20002_1111 | Asthma | non-cancer illness code, self-reported | Uncategorised | 1.20E-18 | 0.068339433 | 1.070728687 |  | 41934 | 361141 |
| NEALE2_6138_3 | O levels/gcses or equivalent | qualifications | measurement | 0.00032555 | 0.017636158 | 1.017792594 |  | 168672 | 357549 |
| NEALE2_22506_114 | Never smoked | tobacco smoking | Uncategorised | 0.000345042 | 0.0356325 | 1.036274946 |  | 36833 | 91353 |
| NEALE2_30210_raw | Eosinophill percentage | measurement | 1.19E-31 | 0.0543237 |  |  |  | 349861 |
| NEALE2_23118_raw | Leg predicted mass (left) | measurement | 1.07E-10 | -0.0182887 |  |  |  | 354766 |
| NEALE2_3872_raw | Age of primiparous women at birth of child | measurement | 0.00160886 | 0.182318 |  |  |  | 25545 |
| NEALE2_894 | Duration of moderate activity | biological process | 0.0038499 | -0.00692242 |  |  |  | 268826 |
| NEALE2_23112_raw | Leg fat mass (right) | measurement | 1.24E-08 | -0.0222564 |  |  |  | 354807 |
| NEALE2_3063_raw | Forced expiratory volume in 1-second (fev1) | measurement | 2.25E-08 | -0.00834792 |  |  |  | 329404 |
| NEALE2_2744 | Birth weight of first child | measurement | 2.14E-05 | 0.0125262 |  |  |  | 155202 |
| NEALE2_49_raw | Hip circumference | measurement | 3.20E-06 | -0.105816 |  |  |  | 360521 |
| NEALE2_2375 | Relative age of first facial hair | measurement | 0.00400833 | 0.00467952 |  |  |  | 161470 |
| NEALE2_4119_raw | Ankle spacing width (right) | Uncategorised | 2.08E-05 | -0.0744034 |  |  |  | 114614 |
| NEALE2_20003_1140888098 | Fluticasone | treatment/medication code | measurement | 0.000200917 | 0.140114267 | 1.150405245 |  | 1578 | 361141 |
| NEALE2_23099_raw | Body fat percentage | measurement | 4.01E-05 | -0.0648011 |  |  |  | 354628 |
| GCST003499 | Reaction time | measurement | 0.0014534 | -0.0142415 |  | PMID:27046643 |  | 111483 |
| NEALE2_129_raw | Place of birth in uk - north co-ordinate | Uncategorised | 1.28E-06 | -1460.86 |  |  |  | 338701 |
| GCST90012107 | Sex hormone-binding globulin levels | measurement | 0.00022 | 0.00468117 |  | PMID:32042192 |  | 189473 |
| GCST90013405 | Liver enzyme levels (alanine transaminase) | measurement | 2.70E-10 | -0.00254537 |  | PMID:33972514 |  | 437267 |
| NEALE2_1070 | Time spent watching television (tv) | Uncategorised | 7.37E-07 | -0.00932313 |  |  |  | 341859 |
| FINNGEN_R6_CD2_BENIGN_OESOPHAGUS_EXALLC | Benign neoplasm: Oesophagus (controls excluding all cancers) | cell proliferation disorder | 0.00344 | -0.34 | 0.711770323 |  | 171 | 209196 |
| SAIGE_244_4 | Hypothyroidism nos | Uncategorised | 0.00000294 | 0.0623 | 1.064281581 |  | 14171 | 405600 |
| SAIGE_599_4 | Urinary incontinence | phenotype | 0.0041 | -0.0469 | 0.954182811 |  | 8856 | 393786 |
| GCST90038603 | Immunological or systemic disorder | immune system disease | 0.0000027 | 0.00283213 | 1.002836144 | PMID:33959723 | 41530 | 484598 |
| GCST90038681 | Psoriasis | integumentary system disease | 0.00019 | 0.000862263 | 1.000862635 | PMID:33959723 | 5427 | 484598 |
| GCST90000617 | Serum 25-Hydroxyvitamin D levels | measurement | 0.00069532 | -0.00706242 |  | PMID:32242144 |  | 417580 |
| FINNGEN_R6_H7_CONJUNCTIVA | Disorders of conjunctiva | disease of visual system | 0.0026 | 0.03533 | 1.03596152 |  | 19762 | 260405 |
| FINNGEN_R6_AUTOIMMUNE | Autoimmune diseases | immune system disease | 1.09E-09 | 0.05326 | 1.054703832 |  | 52657 | 260405 |
| FINNGEN_R6_J10_ASTHMA_INCLAVO | Asthma, including avohilmo | Uncategorised | 0.0000837 | 0.03986 | 1.040665071 |  | 31664 | 260405 |
| FINNGEN_R6_E4_ENDONUTRMET | Endocrine, nutritional and metabolic diseases | endocrine system disease | 0.0022 | 0.02243 | 1.022683444 |  | 97333 | 260405 |
| FINNGEN_R6_E4_HYTHY_AI_STRICT_PURCH | Hypothyroidism, strict autoimmune, 3 medication purchases required | endocrine system disease | 9.19E-10 | 0.06465 | 1.066785584 |  | 32581 | 260405 |
| FINNGEN_R6_E4_DM1 | Type 1 diabetes | Uncategorised | 7.86E-11 | 0.13395 | 1.143335651 |  | 7609 | 222769 |
| FINNGEN_R6_E4_THYROID | Disorders of the thyroid gland | endocrine system disease | 0.00000808 | 0.04354 | 1.044501774 |  | 38430 | 260405 |
| FINNGEN_R6_HYPOTHY_LEVOTHY | Hypothyroidism, levothyroxin purchases | endocrine system disease | 0.00000404 | 0.06204 | 1.064004904 |  | 32581 | 98164 |
| FINNGEN_R6_CD2_BENIGN_LEIOMYOMA_UTERI | Leiomyoma of uterus | cell proliferation disorder | 0.000714 | 0.04271 | 1.043635197 |  | 21518 | 147061 |
| FINNGEN_R6_E4_DM1NOCOMP | Type 1 diabetes without complications | Uncategorised | 1.23E-12 | 0.1587 | 1.171986298 |  | 6376 | 221536 |
| FINNGEN_R6_DM_BCKGRND_RETINA | Background diabetic retinopathy | disease of visual system | 0.000537 | 0.11041 | 1.116735838 |  | 2510 | 244818 |
| FINNGEN_R6_E4_DM1COMA | Type 1 diabetes with coma | phenotype | 0.0000678 | 0.16125 | 1.174978677 |  | 1578 | 216738 |
| FINNGEN_R6_H7_MACULOPATHYDIAB | Diabetic maculopathy | disease of visual system | 0.00152 | 0.10553 | 1.111299443 |  | 2273 | 243463 |
| FINNGEN_R6_D3_OTHERBLOOD | Oher diseases of blood and blood-forming organs | hematologic disease | 0.00166 | 0.15793 | 1.171084216 |  | 923 | 257553 |
| NEALE2_20111_8 | High blood pressure | illnesses of siblings | measurement | 0.00274072 | -0.020379435 | 0.979826822 |  | 58495 | 281619 |
| GCST004617 | Eosinophil percentage of granulocytes | measurement | 6.16E-11 | 0.02468266 |  | PMID:27863252 |  | 170536 |
| NEALE2_4968 | Positional arithmetic | fi4 | biological process | 0.00186158 | 0.038121256 | 1.038857193 |  | 17346 | 115690 |
| NEALE2_6145_2 | Serious illness, injury or assault of a close relative | illness, injury, bereavement, stress in last 2 years | Uncategorised | 0.00042754 | 0.027342984 | 1.027720234 |  | 41459 | 358836 |
| NEALE2_20003_1141191044 | Levothyroxine sodium | treatment/medication code | measurement | 2.55E-09 | 0.074251334 | 1.077077478 |  | 14689 | 361141 |
| NEALE2_6153_2 | Blood pressure medication | medication for cholesterol, blood pressure, diabetes, or take exogenous hormones | Uncategorised | 0.000821663 | -0.028976771 | 0.97143903 |  | 33519 | 193148 |
| NEALE2_20003_1140875408 | Allopurinol | treatment/medication code | measurement | 0.00264669 | -0.069993628 | 0.932399761 |  | 4108 | 361141 |
| NEALE2_6154_3 | Paracetamol | medication for pain relief, constipation, heartburn | Uncategorised | 0.000584211 | -0.020636528 | 0.979574947 |  | 77936 | 357084 |
| NEALE2_20003_1140888552 | Enalapril | treatment/medication code | Uncategorised | 0.000751978 | -0.09533583 | 0.909067591 |  | 2803 | 361141 |
| GCST006288 | Heel bone mineral density | measurement | 0.00033 | 0.0132192 |  | PMID:28869591 |  | 142487 |
| NEALE2_23111_raw | Leg fat percentage (right) | measurement | 0.000011865 | -0.0601312 |  |  |  | 354811 |
| NEALE2_30050_raw | Mean corpuscular haemoglobin | measurement | 0.00182524 | 0.0142525 |  |  |  | 350472 |
| NEALE2_20016_raw | Fluid intelligence score | biological process | 4.02E-05 | 0.0372078 |  |  |  | 117131 |
| NEALE2_23107_raw | Impedance of leg (right) | Uncategorised | 2.85E-10 | 0.508875 |  |  |  | 354817 |
| NEALE2_1558 | Alcohol intake frequency. | measurement | 5.84E-06 | -0.0164377 |  |  |  | 360726 |
| NEALE2_23127_raw | Trunk fat percentage | measurement | 0.000701632 | -0.0610911 |  |  |  | 354619 |
| NEALE2_2714 | Age when periods started (menarche) | measurement | 0.00214382 | 0.00772607 |  |  |  | 188644 |
| NEALE2_100160 | Low calorie drink intake | measurement | 0.00377981 | -0.0182291 |  |  |  | 51427 |
| NEALE2_23119_raw | Arm fat percentage (right) | measurement | 7.93E-07 | -0.0919844 |  |  |  | 354760 |
| NEALE2_23114_raw | Leg predicted mass (right) | measurement | 4.36E-11 | -0.0186272 |  |  |  | 354798 |
| NEALE2_23117_raw | Leg fat-free mass (left) | measurement | 7.77E-11 | -0.0196503 |  |  |  | 354771 |
| GCST90038604 | Hypertension | cardiovascular disease | 0.0022 | -0.00270141 | 0.997302236 | PMID:33959723 | 129909 | 484598 |
| GCST009982 | Trauma exposure | measurement | 0.002141904 | -0.042490052 | 0.9584 | PMID:31969693 | 35269 | 98720 |
| SAIGE_471 | Nasal polyps | cell proliferation disorder | 9.64E-09 | 0.151 | 1.162996658 |  | 3311 | 393356 |
| GCST003155 | Systemic lupus erythematosus | urinary system disease | 0.0022462 | 0.086177696 | 1.09 | PMID:26502338 | 5201 | 14267 |
| GCST003156 | Systemic lupus erythematosus | urinary system disease | 0.0022462 | 0.086177696 | 1.09 | PMID:26502338 | 5201 | 14267 |
| GCST90013410 | Basal cell carcinoma | cell proliferation disorder | 0.0019 | -0.03593772 | 0.964700373 | PMID:33549134 | 17416 | 392871 |
| GCST007429 | Lung function (FVC) | measurement | 0.000297 | -0.009 |  | PMID:30804560 |  | 321047 |
| GCST90011440 | OTU99_171 (Bacteroides) abundance | measurement | 0.001697313 | 0.130830462 |  | PMID:33462482 |  | 8956 |
| GCST90026246 | Pantothenate levels | measurement | 0.003546 | -0.0665 |  | PMID:33437055 |  | 291 |
| FINNGEN_R6_J10_ASTHMACOPDKELA | Asthma/COPD (KELA code 203) | respiratory or thoracic disease | 0.000209 | 0.04248 | 1.043395188 |  | 24334 | 244792 |
| GCST90002389 | Lymphocyte percentage of white cells | measurement | 1.90E-07 | -0.0118497 |  | PMID:32888494 |  | 408112 |
| FINNGEN_R6_RX_ANTIHYP | Antihypertensive medication - note that there are other indications | cardiovascular disease | 0.000485 | -0.029 | 0.971416464 |  | 134687 | 260405 |
| FINNGEN_R6_H7_CHOROIDRETINA | Disorders of choroid and retina | disease of visual system | 0.000173 | 0.04496 | 1.04598602 |  | 19215 | 260405 |
| FINNGEN_R6_E4_HYTHYNAS | Hypothyroidism, other/unspecified | endocrine system disease | 8.35E-09 | 0.0607 | 1.062580092 |  | 32871 | 254846 |
| FINNGEN_R6_Z21_SPECIAL_SCREEN_EXAM_INFECTIOUS_PARASITIC_DISEA | Special screening examination for infectious and parasitic diseases | infectious disease | 0.00416 | 0.0539 | 1.055379059 |  | 7935 | 145101 |
| FINNGEN_R6_E4_DM1OPTH | Type 1 diabetes with ophthalmic complications | injury, poisoning or other complication | 1.55E-10 | 0.18366 | 1.201607207 |  | 3668 | 218828 |
| FINNGEN_R6_E4_DM1NASCOMP | Type 1 diabetes with other specified/multiple/unspecified complications | injury, poisoning or other complication | 6.78E-08 | 0.1408 | 1.151194386 |  | 4455 | 219615 |
| FINNGEN_R6_T1D_STRICT1 | Type 1 diabetes, strict definition, subgroup 1 | Uncategorised | 6.33E-08 | 0.17319 | 1.189092011 |  | 2992 | 215069 |
| FINNGEN_R6_L12_ATROPHICSKIN | Atrophic disorders of skin | integumentary system disease | 0.00206 | 0.10531 | 1.111054984 |  | 2035 | 248644 |
| NEALE2_4957 | Word interpolation | fi3 | biological process | 0.00172367 | 0.037773738 | 1.038496234 |  | 18062 | 116815 |
| GCST004623 | Neutrophil percentage of granulocytes | measurement | 6.83E-10 | -0.02329096 |  | PMID:27863252 |  | 170672 |
| NEALE2_6159_1 | Headache | pain type(s) experienced in last month | phenotype | 3.36E-07 | -0.031328288 | 0.969157358 |  | 71672 | 360391 |
| NEALE2_5001 | Synonym | fi7 | Uncategorised | 0.00281218 | 0.046459822 | 1.04755599 |  | 10534 | 84560 |
| NEALE2_6164_100 | None of the above | types of physical activity in last 4 weeks | Uncategorised | 0.00174178 | -0.033018609 | 0.967520555 |  | 21255 | 359263 |
| NEALE2_20003_1140881856 | Salbutamol | treatment/medication code | measurement | 0.000309132 | 0.08262724 | 1.086136865 |  | 4305 | 361141 |
| FINNGEN_R6_E4_GLUCOPANCREAS | Other disorders of glucose regulation and pancreatic internal secretion | pancreas disease | 0.00241 | 0.1163 | 1.123332821 |  | 1607 | 260405 |
| NEALE2_22506_113 | Ex-smoker | tobacco smoking | Uncategorised | 0.000668 | -0.034441552 | 0.966144807 |  | 33335 | 91353 |
| NEALE2_1448_1 | White | bread type | measurement | 3.71E-06 | -0.026293945 | 0.974048731 |  | 91452 | 348424 |
| NEALE2_23130_raw | Trunk predicted mass | measurement | 4.05E-10 | -0.048349 |  |  |  | 354494 |
| GCST006268 | Reaction time | measurement | 0.000137214 | -0.0066072 |  | PMID:29844566 |  | 330069 |
| NEALE2_1249 | Past tobacco smoking | Uncategorised | 1.12E-05 | 0.0140777 |  |  |  | 332141 |
| NEALE2_2986 | Started insulin within one year diagnosis of diabetes | Uncategorised | 2.74E-05 | 0.1470433 | 1.15840412 |  | 1999 | 16415 |
| NEALE2_23125_raw | Arm fat-free mass (left) | measurement | 2.32E-12 | -0.00825833 |  |  |  | 354668 |
| NEALE2_23108_raw | Impedance of leg (left) | Uncategorised | 3.61E-10 | 0.505274 |  |  |  | 354811 |
| NEALE2_23098_raw | Weight | measurement | 1.01E-10 | -0.227346 |  |  |  | 354838 |
| NEALE2_23110_raw | Impedance of arm (left) | Uncategorised | 3.07E-12 | 0.681419 |  |  |  | 354807 |
| NEALE2_21002_raw | Weight | measurement | 3.03E-11 | -0.232719 |  |  |  | 360116 |
| NEALE2_23106_raw | Impedance of whole body | Uncategorised | 6.11E-13 | 1.19767 |  |  |  | 354795 |
| GCST90012102 | Bioavailable testosterone levels | measurement | 0.000049 | -0.00982012 |  | PMID:32042192 |  | 188507 |
| FINNGEN_R6_CD2_BENIGN_OESOPHAGUS | Benign neoplasm: Oesophagus | cell proliferation disorder | 0.00413 | -0.33352 | 0.716397571 |  | 171 | 260405 |
| FINNGEN_R6_M13_MULTIFIBRO | Multifocal fibrosclerosis | immune system disease | 0.000704 | 0.49794 | 1.645328401 |  | 108 | 253458 |
| GCST90038596 | Respiratory or ear-nose-throat disease | Uncategorised | 1.10E-17 | 0.00707049 | 1.007095545 | PMID:33959723 | 86427 | 484598 |
| GCST90038661 | Allergy, hypersensitivity or anaphylaxis | immune system disease | 0.0000012 | 0.00273871 | 1.002742464 | PMID:33959723 | 35890 | 484598 |
| GCST90038679 | Dermatologic disease | integumentary system disease | 0.00077 | 0.00146766 | 1.001468738 | PMID:33959723 | 20101 | 484598 |
| GCST90000615 | Serum 25-Hydroxyvitamin D levels | measurement | 0.00175832 | -0.00650053 |  | PMID:32242144 |  | 417580 |
| SAIGE_250_1 | Type 1 diabetes | Uncategorised | 0.0000262 | 0.123 | 1.130884421 |  | 2660 | 391416 |
| GCST90000047 | Age at first sexual intercourse | measurement | 2.60E-08 | 0.0123 |  | PMID:34211149 |  | 397338 |
| GCST90027161 | Atopic dermatitis | integumentary system disease | 0.000006149 | 0.0491 | 1.050325378 | PMID:34454985 | 22474 | 796661 |
| SAIGE_709_2 | Sicca syndrome | disease of visual system | 0.00428 | -0.189 | 0.827786507 |  | 513 | 399917 |
| GCST90000050 | Age at first birth | measurement | 1.64E-09 | 0.0588 |  | PMID:34211149 |  | 542901 |
| GCST90011372 | OTU97_140 (Bacteroides) abundance | measurement | 0.001139833 | 0.135395226 |  | PMID:33462482 |  | 8956 |
| GCST012241 | Oropharynx cancer | cell proliferation disorder | 0.001329077 | -0.192 | 0.825306868 | PMID:27749845 | 1090 | 4018 |
| GCST005038 | Allergic disease (asthma, hay fever or eczema) | immune system disease | 3.39E-17 | 0.051 | 1.052322893 | PMID:29083406 | 180129 | 360838 |
| GCST90026199 | Methyl glucopyranoside (alpha + beta) levels | measurement | 0.002407 | -0.088 |  | PMID:33437055 |  | 291 |
| GCST90002316 | Lymphocyte counts | measurement | 1.23E-10 | -0.012851 |  | PMID:32888493 |  | 524923 |
| GCST90002310 | Hemoglobin concentration | measurement | 0.00048 | -0.006783 |  | PMID:32888493 |  | 563946 |
| GCST90002386 | High light scatter reticulocyte percentage of red cells | measurement | 0.0013 | 0.00722715 |  | PMID:32888494 |  | 408112 |
| GCST90002392 | Mean corpuscular volume | measurement | 0.0031 | 0.00644103 |  | PMID:32888494 |  | 408112 |
| FINNGEN_R6_HYPOTHYROIDISM | Hypothyroidism (congenital or acquired) | endocrine system disease | 0.00000625 | 0.06069 | 1.062569467 |  | 32925 | 98381 |
| FINNGEN_R6_L12_ATOPIC | Atopic dermatitis | integumentary system disease | 0.00475 | 0.05051 | 1.051807381 |  | 8383 | 244544 |
| FINNGEN_R6_T1D_WIDE | Type 1 diabetes, wide definition | Uncategorised | 8.17E-11 | 0.12416 | 1.132197008 |  | 8615 | 222923 |
| FINNGEN_R6_ATOPIC_STRICT_REIMB | Atopic dermatitis, strict definition with reimbursement | integumentary system disease | 0.00124 | 0.06407 | 1.066167028 |  | 6739 | 242769 |
| FINNGEN_R6_E4_DM1REN | Type 1 diabetes with renal complications | urinary system disease | 0.0000411 | 0.18753 | 1.206266437 |  | 1195 | 216355 |
| FINNGEN_R6_DM_MACULOPATHY | Diabetic maculopathy | disease of visual system | 0.00174 | 0.10431 | 1.109944484 |  | 2273 | 253236 |
| NEALE2_6143_4 | Cycle | transport type for commuting to job workplace | Uncategorised | 0.00215057 | 0.038673066 | 1.039430603 |  | 14885 | 190832 |
| NEALE2_6164_4 | Light diy (eg: pruning, watering the lawn) | types of physical activity in last 4 weeks | Uncategorised | 1.94E-05 | 0.021216577 | 1.021443249 |  | 172584 | 359263 |
| FINNGEN_R6_ASTHMA_EOSINOPHIL_SUGG | Suggestive for eosinophilic asthma | Uncategorised | 0.000579 | 0.14063 | 1.1509987 |  | 1464 | 159916 |
| NEALE2_20003_1141176832 | Seretide 50 evohaler | treatment/medication code | measurement | 8.63E-05 | 0.09029859 | 1.094501041 |  | 4269 | 361141 |
| FINNGEN_R6_O15_PREG_PROLONGED | Prolonged pregnancy | pregnancy or perinatal disease | 0.00354 | 0.09391 | 1.09846088 |  | 2393 | 125510 |
| NEALE2_22127 | Doctor diagnosed asthma | Uncategorised | 5.60E-07 | 0.073788349 | 1.076578922 |  | 11717 | 91787 |
| NEALE2_20002_1226 | Hypothyroidism/myxoedema | non-cancer illness code, self-reported | Uncategorised | 1.28E-10 | 0.073375395 | 1.076134437 |  | 17574 | 361141 |
| NEALE2_20003_2038460150 | Paracetamol | treatment/medication code | Uncategorised | 0.00330782 | -0.018729791 | 0.981444522 |  | 66347 | 361141 |
| NEALE2_20003_1140861998 | Ventolin 100micrograms inhaler | treatment/medication code | measurement | 4.22E-06 | 0.068440743 | 1.070837168 |  | 10380 | 361141 |
| NEALE2_23102_raw | Whole body water mass | measurement | 2.09E-11 | -0.0771149 |  |  |  | 354834 |
| NEALE2_20003_1140862382 | Becotide 50 inhaler | treatment/medication code | measurement | 0.00260347 | 0.096065218 | 1.100830856 |  | 2206 | 361141 |
| NEALE2_30040_raw | Mean corpuscular volume | measurement | 0.00106338 | 0.0359217 |  |  |  | 350473 |
| NEALE2_30530_raw | Sodium in urine | phenotype | 0.000356918 | -0.37802 |  |  |  | 350061 |
| NEALE2_23116_raw | Leg fat mass (left) | measurement | 1.84E-08 | -0.0214622 |  |  |  | 354788 |
| NEALE2_806 | Job involves mainly walking or standing | Uncategorised | 0.000303514 | -0.0131326 |  |  |  | 204956 |
| NEALE2_30120_raw | Lymphocyte count | measurement | 0.000533798 | -0.00981545 |  |  |  | 349856 |
| NEALE2_22501_raw | Year ended full time education | measurement | 0.00100042 | 0.0532828 |  |  |  | 91777 |
| NEALE2_23100_raw | Whole body fat mass | measurement | 1.25E-07 | -0.121973 |  |  |  | 354244 |
| NEALE2_2139_raw | Age first had sexual intercourse | measurement | 1.90E-08 | 0.0558946 |  |  |  | 317694 |
| GCST90000027 | Appendicular lean mass | measurement | 0.000099 | -0.0105912 |  | PMID:33097823 |  | 244730 |
| GCST90000288 | Myocardial fractal dimension (slice 2) | measurement | 9.68E-05 | 0.0019886 |  | PMID:32814899 |  | 18096 |
| GCST90000290 | Myocardial fractal dimension (slice 4) | measurement | 1.35E-05 | 0.0015068 |  | PMID:32814899 |  | 18096 |
| GCST006979 | Heel bone mineral density | measurement | 0.0000016 | 0.00990872 |  | PMID:30598549 |  | 426824 |
| NEALE2_20003_1141145668 | Diovan 40mg capsule | treatment/medication code | measurement | 0.00339196 | 0.230506323 | 1.259237429 |  | 361 | 361141 |
| GCST90038646 | Migraine | cardiovascular disease | 0.0041 | -0.00102816 | 0.998972368 | PMID:33959723 | 13971 | 484598 |
| GCST90038599 | Diabetes or endocrine disease | pancreas disease | 2.70E-11 | 0.00432865 | 1.004338032 | PMID:33959723 | 51949 | 484598 |
| GCST005195 | Coronary artery disease | cardiovascular disease | 0.0023 | 0.0226149 | 1.022872555 | PMID:29212778 | 122733 | 547261 |
| GCST90000048 | Age at first birth | measurement | 4.57E-09 | 0.0622 |  | PMID:34211149 |  | 418758 |
| GCST90026008 | Lactate levels | measurement | 0.003323 | 0.0188 |  | PMID:33437055 |  | 291 |
| FINNGEN_R6_MIGRAINE_TRIPTAN | Migraine, single triptan purchase ok & required. ICD-code if available is included | Uncategorised | 0.00472 | -0.03219 | 0.968322583 |  | 25316 | 260405 |
| GCST90002403 | Red blood cell count | measurement | 0.00012 | -0.00852025 |  | PMID:32888494 |  | 408112 |
| FINNGEN_R6_RX_PARACETAMOL_NSAID | Paracetamol of NSAID medication | biological process | 0.00218 | -0.02706 | 0.973302842 |  | 213342 | 260405 |
| FINNGEN_R6_DM_RETINOPATHY | Diabetic retinopathy | disease of visual system | 0.0000507 | 0.05085 | 1.052165057 |  | 18097 | 257444 |
| FINNGEN_R6_L12_PAPULOSQUAMOUS | Papulosquamous disorders | integumentary system disease | 0.00439 | 0.05144 | 1.052786017 |  | 8082 | 260405 |
| FINNGEN_R6_O15_PREG_DM | Diabetes mellitus in pregnancy | pregnancy or perinatal disease | 0.000283 | 0.06974 | 1.072229365 |  | 7676 | 138100 |
| GCST90014023 | Type 1 diabetes | pancreas disease | 4.99E-61 | 0.241255 | 1.27284557 | PMID:34012112 | 18942 | 520580 |
| FINNGEN_R6_E4_DM1KETO | Type 1 diabetes with ketoacidosis | pancreas disease | 0.00000274 | 0.19046 | 1.20980598 |  | 1628 | 216788 |
| GCST004627 | Lymphocyte counts | measurement | 0.0001073 | -0.01470731 |  | PMID:27863252 |  | 171643 |
| GCST004606 | Eosinophil counts | measurement | 6.11E-13 | 0.02707775 |  | PMID:27863252 |  | 172275 |
| NEALE2_20002_1387 | Hayfever/allergic rhinitis | non-cancer illness code, self-reported | respiratory or thoracic disease | 8.09E-07 | 0.052705893 | 1.054119576 |  | 20667 | 361141 |
| NEALE2_2664_3 | Health precaution | reason for reducing amount of alcohol drunk | Uncategorised | 0.00307996 | 0.025583983 | 1.025914062 |  | 43868 | 134033 |
| NEALE2_30150 | Eosinophill count | measurement | 2.21E-16 | 0.0152464 |  |  |  | 349856 |
| NEALE2_30180_raw | Lymphocyte percentage | measurement | 1.22E-08 | -0.104077 |  |  |  | 349861 |
| NEALE2_2867_raw | Age started smoking in former smokers | biological process | 0.00173768 | 0.055967 |  |  |  | 88898 |
| NEALE2_23109_raw | Impedance of arm (right) | Uncategorised | 4.42E-11 | 0.627328 |  |  |  | 354792 |
| NEALE2_23115_raw | Leg fat percentage (left) | measurement | 4.07E-06 | -0.0601333 |  |  |  | 354791 |
| NEALE2_102_raw | Pulse rate, automated reading | measurement | 0.000001699 | -0.137566 |  |  |  | 340162 |
| NEALE2_3786_raw | Age asthma diagnosed | Uncategorised | 0.00411737 | -0.383012 |  |  |  | 36955 |
| NEALE2_30510_raw | Creatinine (enzymatic) in urine | measurement | 0.00166777 | -43.0571 |  |  |  | 350812 |
| NEALE2_23129_raw | Trunk fat-free mass | measurement | 3.96E-10 | -0.0504499 |  |  |  | 354530 |
| NEALE2_3143_raw | Ankle spacing width | Uncategorised | 4.81E-08 | -0.0707868 |  |  |  | 206589 |
| NEALE2_30010_raw | Red blood cell (erythrocyte) count | measurement | 0.000154349 | -0.00333088 |  |  |  | 350475 |
| NEALE2_20477 | Ever talked to a health professional about unusual or psychotic experiences | phenotype | 0.00162612 | -0.150923036 | 0.859913878 |  | 1225 | 5788 |
| NEALE2_22704_raw | Home location - north co-ordinate (rounded) | Uncategorised | 5.07E-06 | -1479.79 |  |  |  | 361187 |
| GCST90000289 | Myocardial fractal dimension (slice 3) | measurement | 4.33E-06 | 0.0022609 |  | PMID:32814899 |  | 18096 |
| NEALE2_20003_1141157126 | Montelukast product | treatment/medication code | measurement | 0.000118151 | 0.222137799 | 1.248743441 |  | 672 | 361141 |
| NEALE2_22702_raw | Home location - east co-ordinate (rounded) | Uncategorised | 0.00264309 | 573.133 |  |  |  | 361187 |
| FINNGEN_R6_D3_COMBIMMUDEF | Combined immunodeficiencies | phenotype | 0.0003 | 0.38219 | 1.465490502 |  | 208 | 256857 |
| FINNGEN_R6_L12_FACTITIALDERMAT | Factitial dermatitis | integumentary system disease | 0.00139 | 0.51013 | 1.665507697 |  | 92 | 246701 |
| GCST90038664 | Allergic rhinitis | respiratory or thoracic disease | 1.50E-08 | 0.00283219 | 1.002836204 | PMID:33959723 | 27415 | 484598 |
| GCST90038602 | Hematological or dermatological disease | integumentary system disease | 0.00012 | 0.0019228 | 1.00192465 | PMID:33959723 | 26874 | 484598 |
| GCST006250 | Intelligence | biological process | 0.00000127 | 0.014343873 |  | PMID:29942086 |  | 269867 |
| GCST90000046 | Age at first sexual intercourse | measurement | 7.40E-08 | 0.0176 |  | PMID:34211149 |  | 182791 |
| GCST90013791 | Type 1 diabetes | Uncategorised | ############ | 0.192082837 | 1.211770892 |  | 15573 | 173981 |
| GCST010681 | Type 1 diabetes | pancreas disease | 3.05E-22 | 0.2382 | 1.26896296 | PMID:32005708 | 9266 | 24840 |
| NEALE2_4080_raw | Systolic blood pressure, automated reading | measurement | 0.000331424 | -0.159553 |  |  |  | 340159 |
| GCST90002298 | Eosinophil counts | measurement | 1.06E-36 | 0.026511 |  | PMID:32888493 |  | 474237 |
| GCST90002384 | Hemoglobin | measurement | 0.0026 | -0.0067907 |  | PMID:32888494 |  | 408112 |
| FINNGEN_R6_C3_OTHER_SKIN_EXALLC | Other malignant neoplasms of skin (=non-melanoma skin cancer) (controls excluding all cancers) | cell proliferation disorder | 0.00378 | -0.04388 | 0.957068799 |  | 13705 | 217775 |
| FINNGEN_R6_H7_RETINALDISOTH | Other retinal disorders | disease of visual system | 0.0000607 | 0.05664 | 1.058274763 |  | 13627 | 254817 |
| FINNGEN_R6_H7_EYE | Diseases of the eye and adnexa | disease of visual system | 0.00176 | 0.02121 | 1.021436531 |  | 92826 | 260405 |
| FINNGEN_R6_C3_SKIN_EXALLC | Malignant neoplasm of skin (controls excluding all cancers) | cell proliferation disorder | 0.00367 | -0.04394 | 0.957011376 |  | 13707 | 217777 |
| FINNGEN_R6_J10_LOWCHRON | Chronic lower respiratory diseases | respiratory or thoracic disease | 0.000811 | 0.03102 | 1.031506134 |  | 39947 | 260405 |
| FINNGEN_R6_ATOPIC_STRICT | Atopic dermatitis, strict definition | integumentary system disease | 0.00179 | 0.06337 | 1.065420972 |  | 6412 | 260078 |
| FINNGEN_R6_N14_BREAST | Disorders of breast | reproductive system or breast disease | 0.00241 | -0.04822 | 0.952924121 |  | 10447 | 147061 |
| FINNGEN_R6_E4_HYTHY_AI_STRICT | Hypothyroidism, strict autoimmune | endocrine system disease | 8.81E-11 | 0.0734 | 1.076160915 |  | 28688 | 235230 |
| FINNGEN_R6_C3_CANCER | Malignant neoplasm | cell proliferation disorder | 0.00241 | -0.02551 | 0.974812631 |  | 51271 | 260405 |
| FINNGEN_R6_HYPOTHY_REIMB | Hypothyroidism, drug reimbursement | endocrine system disease | 0.00457 | 0.06014 | 1.061985214 |  | 8575 | 74138 |
| FINNGEN_R6_J10_NASALPOLYP | Nasal polyp | cell proliferation disorder | 6.22E-07 | 0.12768 | 1.136189364 |  | 3919 | 203127 |
| FINNGEN_R6_T1D_WIDE1 | Type 1 diabetes, wide definition, subgroup 1 | Uncategorised | 1.06E-11 | 0.15272 | 1.164998734 |  | 6272 | 218349 |
| FINNGEN_R6_T1D_STRICT | Type 1 diabetes, strict definition | Uncategorised | 3.02E-08 | 0.17136 | 1.186917963 |  | 3242 | 217550 |
| FINNGEN_R6_DM_MACULOPATHY_EXMORE | Diabetic maculopathy (more control exclusions) | disease of visual system | 0.00136 | 0.10802 | 1.114070027 |  | 2273 | 216574 |
| FINNGEN_R6_D3_COAGDEF_PURPUR_HAEMORRHAGIC | Coagulation defects, purpura and other haemorrhagic conditions | hematologic disease | 0.00464 | -0.07542 | 0.927353916 |  | 3351 | 260405 |
| FINNGEN_R6_D3_ANAEMIA_B12_DEF | Vitamin B12 deficiency anaemia | phenotype | 0.00131 | 0.11065 | 1.117003887 |  | 2064 | 253083 |
| GCST004600 | Eosinophil percentage of white cells | measurement | 4.96E-14 | 0.02831321 |  | PMID:27863252 |  | 172378 |
| NEALE2_1448_3 | Wholemeal or wholegrain | bread type | measurement | 9.04E-06 | 0.022505194 | 1.022760346 |  | 147411 | 348424 |
| NEALE2_20116_1 | Previous | smoking status | biological process | 1.41E-05 | -0.022268755 | 0.977977363 |  | 127550 | 359706 |
| NEALE2_20428 | Professional informed about anxiety | phenotype | 0.000867277 | 0.053232235 | 1.054674549 |  | 14128 | 36366 |
| NEALE2_6152_100 | None of the above | blood clot, dvt, bronchitis, emphysema, asthma, rhinitis, eczema, allergy diagnosed by doctor | Uncategorised | 6.63E-22 | -0.051080886 | 0.950201809 |  | 116005 | 360527 |
| NEALE2_6138_1 | College or university degree | qualifications | measurement | 1.07E-09 | 0.032096414 | 1.032617059 |  | 115981 | 357549 |
| NEALE2_6159_3 | Neck or shoulder pain | pain type(s) experienced in last month | phenotype | 0.00034826 | -0.021207127 | 0.979016163 |  | 82120 | 360391 |
| NEALE2_1717 | Skin colour | phenotype | 0.00430177 | -0.00413061 |  |  |  | 356530 |
| NEALE2_23120_raw | Arm fat mass (right) | measurement | 1.70E-08 | -0.00866394 |  |  |  | 354736 |
| NEALE2_23105_raw | Basal metabolic rate | measurement | 1.10E-11 | -14.2232 |  |  |  | 354825 |
| NEALE2_2754_raw | Age at first live birth | measurement | 7.21E-08 | 0.0987723 |  |  |  | 131987 |
| NEALE2_2217_raw | Age started wearing glasses or contact lenses | disease of visual system | 0.00059601 | -0.152983 |  |  |  | 310992 |
| NEALE2_738 | Average total household income before tax | Uncategorised | 1.65E-06 | 0.0140621 |  |  |  | 311028 |
| GCST90012792 | Acceptance of an invitation to participate in a mental health questionnaire | phenotype | 0.00022 | 0.00378095 | 1.003788107 | PMID:33563987 | 146074 | 294787 |
| NEALE2_48_raw | Waist circumference | measurement | 2.12E-07 | -0.153338 |  |  |  | 360564 |
| NEALE2_20075_raw | Home location at assessment - north co-ordinate (rounded) | Uncategorised | 7.86E-06 | -1446.34 |  |  |  | 357793 |
| NEALE2_20150_raw | Forced expiratory volume in 1-second (fev1), best measure | measurement | 2.97E-10 | -0.010138 |  |  |  | 272338 |
| NEALE2_1190 | Nap during day | Uncategorised | 0.00232026 | -0.00440748 |  |  |  | 360855 |
| FINNGEN_R6_ST19_INJURY_MUSCLE_TENDON_ANKLE_FOOT_LEVEL | Injury of muscle and tendon at ankle and foot level | injury, poisoning or other complication | 0.000743 | -0.26502 | 0.767190606 |  | 376 | 245974 |
| NEALE2_130_raw | Place of birth in uk - east co-ordinate | Uncategorised | 0.00439718 | 509.58 |  |  |  | 338701 |
| GCST003496 | Educational attainment | measurement | 7.29E-06 | 0.0200923 |  | PMID:27046643 |  | 111114 |
| GCST90012111 | Sex hormone-binding globulin levels | measurement | 0.0017 | 0.00314598 |  | PMID:32042192 |  | 370125 |
| SAIGE_495 | Asthma | Uncategorised | 4.47E-10 | 0.0619 | 1.063855954 |  | 26332 | 401837 |
| GCST009763 | Snoring | measurement | 0.0017 | -0.00451912 | 0.995491076 | PMID:32060260 | 61792 | 218346 |
| SAIGE_244 | Hypothyroidism | endocrine system disease | 0.000015 | 0.0563 | 1.057915011 |  | 14871 | 406300 |
| GCST90038616 | Asthma | respiratory or thoracic disease | 2.70E-23 | 0.00688242 | 1.006906158 | PMID:33959723 | 56087 | 484598 |
| GCST90038635 | Thyroid problem (not cancer) | endocrine system disease | 3.30E-12 | 0.00342143 | 1.00342729 | PMID:33959723 | 28254 | 484598 |
| GCST90038624 | Gastroesophageal reflux disease or gastric reflux | gastrointestinal disease | 0.0039 | -0.0012554 | 0.998745388 | PMID:33959723 | 20381 | 484598 |
| GCST90019384 | SPARC-related modular calcium-binding protein 1 measurement | measurement | 0.002212 | 0.0422 |  | PMID:33328453 |  | 10708 |
| GCST007431 | Lung function (FEV1/FVC) | measurement | 8.36E-09 | -0.0145 |  | PMID:30804560 |  | 321047 |
| SAIGE_149_1 | Cancer of oropharynx | cell proliferation disorder | 0.00164 | -0.301 | 0.740077773 |  | 246 | 407067 |
| GCST006572 | Cognitive performance | measurement | 1.28E-07 | 0.01592 |  | PMID:30038396 |  | 257841 |
| GCST90026049 | 1-methylnicotinamide levels | measurement | 0.004286 | -0.0844 |  | PMID:33437055 |  | 291 |
| GCST90014325 | Asthma | respiratory or thoracic disease | ############ | 0.055648442 | 1.057225942 | PMID:34103634 | 56167 | 408422 |
| GCST90002334 | Mean corpuscular volume | measurement | 0.000227 | 0.007073 |  | PMID:32888493 |  | 544127 |
| GCST90002383 | Hematocrit | measurement | 0.0046 | -0.00638293 |  | PMID:32888494 |  | 408112 |
| GCST90002382 | Eosinophil percentage of white cells | measurement | 8.90E-39 | 0.029327 |  | PMID:32888494 |  | 408112 |
| GCST90002387 | Immature fraction of reticulocytes | measurement | 0.0039 | 0.00667803 |  | PMID:32888494 |  | 408112 |
| FINNGEN_R6_DM_RETINA_PROLIF | Proliferative diabetic retinopathy | disease of visual system | 0.00102 | 0.05271 | 1.054123905 |  | 10860 | 253168 |
| FINNGEN_R6_ST19_INJURI_ANKLE_FOOT | Injuries to the ankle and foot | musculoskeletal or connective tissue disease | 0.00275 | -0.03957 | 0.961202667 |  | 14807 | 260405 |
| FINNGEN_R6_O15_OTHER_MATERN_DIS_ELSEWHERE | Other maternal diseases classifiable elsewhere but complicating pregnancy, childbirth and the puerperium | pregnancy or perinatal disease | 0.00492 | 0.05813 | 1.059852768 |  | 6243 | 146815 |
| FINNGEN_R6_E4_DM1_STRICT | Type 1 diabetes, strict (exclude DM2) | Uncategorised | 1.71E-07 | 0.15662 | 1.1695511 |  | 3392 | 219160 |
| FINNGEN_R6_H7_RETINOPATHYDIAB | Diabetic retinopathy | disease of visual system | 0.0000476 | 0.09937 | 1.10447488 |  | 4525 | 245715 |
| FINNGEN_R6_APPENDACUT_COMPLIC | Acute appendicitis, with complications | gastrointestinal disease | 0.00301 | -0.06868 | 0.933625392 |  | 4496 | 244571 |
| FINNGEN_R6_H7_RETINOPATHYDIAB_BKG | Diabetic background retinopathy | disease of visual system | 0.00064 | 0.10876 | 1.114894743 |  | 2510 | 243700 |
| NEALE2_6154_100 | None of the above | medication for pain relief, constipation, heartburn | Uncategorised | 0.00242774 | 0.015165198 | 1.015280773 |  | 159041 | 357084 |
| NEALE2_22606_2 | Often | workplace very noisy | Uncategorised | 0.00480664 | -0.035231575 | 0.965381833 |  | 17469 | 90653 |
| NEALE2_6150_100 | None of the above | vascular/heart problems diagnosed by doctor | Uncategorised | 1.97E-05 | 0.022408704 | 1.022661665 |  | 106855 | 360420 |
| NEALE2_1210 | Snoring | phenotype | 0.00271543 | 0.015609701 | 1.015732169 |  | 125656 | 336320 |
| NEALE2_6164_1 | Walking for pleasure (not as a means of transport) | types of physical activity in last 4 weeks | Uncategorised | 0.000545742 | 0.019128756 | 1.019312883 |  | 100689 | 359263 |
| NEALE2_6150_4 | High blood pressure | vascular/heart problems diagnosed by doctor | measurement | 5.79E-06 | -0.024737097 | 0.975566357 |  | 97139 | 360420 |
| GCST004624 | Sum eosinophil basophil counts | measurement | 1.52E-11 | 0.0254026 |  | PMID:27863252 |  | 171771 |
| NEALE2_6138_100 | None of the above | qualifications | Uncategorised | 3.12E-11 | -0.042347026 | 0.958537086 |  | 61093 | 357549 |
| NEALE2_22609_2 | Often | workplace very dusty | Uncategorised | 0.000235549 | -0.058627872 | 0.943057642 |  | 9561 | 89631 |
| NEALE2_1508_3 | Ground coffee (include espresso, filter etc) | coffee type | biological process | 0.00270112 | 0.019919931 | 1.020119657 |  | 64962 | 283449 |
| NEALE2_20160 | Ever smoked | biological process | 0.00303717 | -0.015004323 | 0.985107681 |  | 141560 | 359751 |
| NEALE2_20154_raw | Forced expiratory volume in 1-second (fev1), predicted percentage | measurement | 0.000362775 | -0.261235 |  |  |  | 117241 |
| NEALE2_404_raw | Duration to first press of snap-button in each round | Uncategorised | 6.74E-06 | -1.32968 |  |  |  | 358500 |
| NEALE2_23126_raw | Arm predicted mass (left) | measurement | 7.37E-13 | -0.00794276 |  |  |  | 354653 |
| NEALE2_3761_raw | Age hay fever, rhinitis or eczema diagnosed | integumentary system disease | 9.53E-08 | -0.452316 |  |  |  | 72232 |
| NEALE2_4194_raw | Pulse rate | measurement | 0.00158152 | -0.156979 |  |  |  | 118850 |
| NEALE2_23124_raw | Arm fat mass (left) | measurement | 5.63E-08 | -0.00932211 |  |  |  | 354673 |
| NEALE2_1578 | Average weekly champagne plus white wine intake | measurement | 0.000164502 | 0.00939307 |  |  |  | 257623 |
| NEALE2_23101_raw | Whole body fat-free mass | Uncategorised | 1.54E-11 | -0.1054 |  |  |  | 354808 |
| NEALE2_1200 | Sleeplessness / insomnia | nervous system disease | 0.000125677 | -0.00677239 |  |  |  | 360738 |
| NEALE2_23104_raw | Body mass index (bmi) | measurement | 3.55E-11 | -0.0782187 |  |  |  | 354831 |
| NEALE2_23128_raw | Trunk fat mass | measurement | 4.00E-06 | -0.0593248 |  |  |  | 354597 |
| NEALE2_23123_raw | Arm fat percentage (left) | measurement | 3.35E-06 | -0.0873651 |  |  |  | 354707 |
| NEALE2_3486 | Ever tried to stop smoking | biological process | 0.00326229 | -0.0185913 |  |  |  | 27467 |
| NEALE2_3062_raw | Forced vital capacity (fvc) | measurement | 0.00056427 | -0.00667552 |  |  |  | 329404 |
| GCST90012112 | Total testosterone levels | measurement | 0.0000067 | -0.0134935 |  | PMID:32042192 |  | 230454 |
| SAIGE_530_14 | Reflux esophagitis | gastrointestinal disease | 0.000945 | -0.0495 | 0.951705158 |  | 10551 | 379826 |
| GCST90038637 | Hypothyroidism or myxedema | endocrine system disease | 3.90E-16 | 0.0036869 | 1.003693705 | PMID:33959723 | 23497 | 484598 |
| GCST90038670 | Nasal or sinus disorder | Uncategorised | 0.0015 | 0.00081016 | 1.000810488 | PMID:33959723 | 6734 | 484598 |
| GCST90000025 | Appendicular lean mass | measurement | 7.71E-11 | -0.013 |  | PMID:33097823 |  | 450243 |
| GCST90019423 | Vitamin K-dependent protein C measurement | measurement | 0.002855 | -0.0427 |  | PMID:33328453 |  | 10708 |
| GCST90011452 | OTU99_30 (Parasutterella) abundance | measurement | 0.003733327 | -0.083325125 |  | PMID:33462482 |  | 8956 |
| GCST007430 | Peak expiratory flow | measurement | 0.000454 | -0.0093 |  | PMID:30804560 |  | 321047 |
| GCST90026078 | 3-carboxy-4-methyl-5-propyl-2-furanpropanoate (cmpf) levels | measurement | 0.00128 | -0.2163 |  | PMID:33437055 |  | 291 |
| GCST90002363 | Red blood cell count | measurement | 0.00000421 | -0.008964 |  | PMID:32888493 |  | 545203 |
| GCST90002304 | Hematocrit | measurement | 0.000877 | -0.006486 |  | PMID:32888493 |  | 562259 |
| FINNGEN_R6_HYPOTHY_PURCHASE | Hypothyroidism and >3 levothyroxin purchases | endocrine system disease | 0.00000404 | 0.06204 | 1.064004904 |  | 32581 | 98164 |
| FINNGEN_R6_DM_RETINOPATHY_EXMORE | Diabetic retinopathy (more control exclusions) | disease of visual system | 0.0000929 | 0.05046 | 1.051754792 |  | 18097 | 224461 |
| GCST90002396 | Mean reticulocyte volume | measurement | 0.0049 | 0.00633237 |  | PMID:32888494 |  | 408112 |
| FINNGEN_R6_E4_DMNAS | Unspecified diabetes | pancreas disease | 0.00000456 | 0.10941 | 1.115619661 |  | 4777 | 219937 |
| FINNGEN_R6_O15_OBSTET_NAS | Other obstetric conditions, not elsewhere classified | pregnancy or perinatal disease | 0.00323 | 0.05971 | 1.061528659 |  | 6489 | 147061 |
| NEALE2_22611_2 | Often | workplace had a lot of cigarette smoke from other people smoking | Uncategorised | 0.000287385 | -0.048349772 | 0.952800466 |  | 14941 | 89803 |
| FINNGEN_R6_D3_PURPURA_AND3_OTHER_HAEMORRHAGIC | Purpura and other haemorrhagic conditions | hematologic disease | 0.00238 | -0.09801 | 0.906639837 |  | 2258 | 259312 |
| NEALE2_6154_5 | Omeprazole (e.g. zanprol) | medication for pain relief, constipation, heartburn | Uncategorised | 0.000453381 | -0.036697464 | 0.963967726 |  | 21464 | 357084 |
| FINNGEN_R6_H7_RETINOPATHYDIAB_NAS | Other diabetic retinopathy | disease of visual system | 0.000158 | 0.16649 | 1.181151724 |  | 1279 | 242469 |
| NEALE2_6138_2 | A levels/as levels or equivalent | qualifications | measurement | 1.03E-07 | 0.029294798 | 1.029728111 |  | 98304 | 357549 |
| NEALE2_6159_100 | None of the above | pain type(s) experienced in last month | Uncategorised | 1.62E-06 | 0.024302896 | 1.024600618 |  | 145514 | 360391 |
| FINNGEN_R6_L12_LICHENSCLERATROPH | Lichen sclerosus et atrophicus | integumentary system disease | 0.000464 | 0.15555 | 1.16830035 |  | 1217 | 247826 |
| FINNGEN_R6_C3_BLADDER_EXALLC | Malignant neoplasm of bladder (controls excluding all cancers) | cell proliferation disorder | 0.0024 | -0.11492 | 0.891437456 |  | 1701 | 205771 |
| NEALE2_22126 | Doctor diagnosed hayfever or allergic rhinitis | respiratory or thoracic disease | 3.21E-06 | 0.054515304 | 1.056028638 |  | 20904 | 91787 |
| NEALE2_20116_0 | Never | smoking status | Uncategorised | 6.95E-09 | 0.028615551 | 1.029028909 |  | 164638 | 359706 |
| NEALE2_6152_8 | Asthma | blood clot, dvt, bronchitis, emphysema, asthma, rhinitis, eczema, allergy diagnosed by doctor | Uncategorised | 8.26E-18 | 0.066854628 | 1.069140044 |  | 41633 | 360527 |
| NEALE2_20023_raw | Mean time to correctly identify matches | measurement | 1.13E-05 | -1.1826 |  |  |  | 358695 |
| NEALE2_20151_raw | Forced vital capacity (fvc), best measure | measurement | 3.59E-05 | -0.00813025 |  |  |  | 272338 |
| NEALE2_23121_raw | Arm fat-free mass (right) | measurement | 9.57E-11 | -0.00708043 |  |  |  | 354732 |
| NEALE2_845 | Age completed full time education | measurement | 1.65E-07 | 0.0124417 |  |  |  | 240547 |
| NEALE2_23122_raw | Arm predicted mass (right) | measurement | 8.12E-11 | -0.00669823 |  |  |  | 354726 |
| FINNGEN_R6_E4_DM1NEU | Type 1 diabetes with neurological complications | nervous system disease | 0.00234 | 0.17502 | 1.191270042 |  | 740 | 215900 |
| NEALE2_21001_raw | Body mass index (bmi) | measurement | 1.37E-11 | -0.0795536 |  |  |  | 359983 |
| NEALE2_23113_raw | Leg fat-free mass (right) | measurement | 4.39E-11 | -0.0198634 |  |  |  | 354798 |
| GCST90000026 | Appendicular lean mass | measurement | 3.00E-08 | -0.0158685 |  | PMID:33097823 |  | 205513 |
| GCST003498 | Verbal-numerical reasoning | biological process | 0.00421217 | 0.0224878 |  | PMID:27046643 |  | 36035 |
| NEALE2_20002_1274 | Eye infection | non-cancer illness code, self-reported | infectious disease | 0.0045022 | -0.22415741 | 0.799189324 |  | 359 | 361141 |
| GCST90000529 | Type 1 diabetes | pancreas disease | ############ | 0.224165645 | 1.251278271 | PMID:33830302 | 7467 | 17685 |
| GCST90000045 | Age at first sexual intercourse | measurement | 0.0008 | 0.0098 |  | PMID:34211149 |  | 214547 |
| SAIGE_715 | Other inflammatory spondylopathies | musculoskeletal or connective tissue disease | 0.00489 | -0.103 | 0.902126973 |  | 1671 | 366756 |
| SAIGE_433_1 | Occlusion and stenosis of precerebral arteries | cardiovascular disease | 0.00348 | 0.127 | 1.135417018 |  | 1185 | 400202 |
| GCST007432 | FEV1 | measurement | 5.57E-10 | -0.0153 |  | PMID:30804560 |  | 321047 |
| SAIGE_377 | Disorders of optic nerve and visual pathways | Uncategorised | 0.00408 | 0.224 | 1.251071019 |  | 370 | 401615 |

**Supplementary Table S7. Traits associated with rs1689510 in the UK Biobank, FinnGen, and/or GWAS Catalog summary statistics repository sourced from the OpenTargets Genetics Portal.** A p-value equal to or less than 5*e*−8 was considered statistically significant.

| **rsid** | **hg19_coordinates** | **a1** | **a2** | **trait** | **pmid** | **beta** | **p** | **n** |
| --- | --- | --- | --- | --- | --- | --- | --- | --- |
| rs61134397 | chr12:56138580 | A | C | Cause of death: pharynx, unspecified | UKBB | -0.04566 | 1.763E-21 | 7637 |
| rs61134397 | chr12:56138580 | A | C | Cause of death: liver cell carcinoma | UKBB | -0.06698 | 1.285E-07 | 7637 |
| rs61134397 | chr12:56138580 | A | C | Parkinsons disease | UKBB | -0.00188 | 9.069E-06 | 337199 |
| rs61134397 | chr12:56138580 | A | C | Other erythematous conditions | UKBB | -0.00169 | 7.432E-06 | 337199 |
| rs61134397 | chr12:56138580 | A | C | Supervision of high-risk pregnancy | UKBB | -0.00215 | 1.259E-06 | 337199 |
| rs117721518 | chr12:56139172 | A | G | Cause of death: car occupant injured in unspecified traffic accident | UKBB | 0.01697 | 4.097E-08 | 7637 |
| rs188443236 | chr12:56141352 | A | T | Treatment with diclomax sr 75mg m or r capsule | UKBB | -0.0025 | 2.436E-06 | 337159 |
| rs188443236 | chr12:56141352 | A | T | Treatment with premique cycle 10mg tablet | UKBB | -0.00374 | 2.469E-08 | 337159 |
| rs188443236 | chr12:56141352 | A | T | Cause of death: follicular non-hodgkins lymphoma, unspecified | UKBB | -0.04802 | 5.671E-07 | 7637 |
| rs188443236 | chr12:56141352 | A | T | Other hearing loss | UKBB | -0.00508 | 8.566E-07 | 337199 |
| rs188443236 | chr12:56141352 | A | T | Preterm delivery | UKBB | -0.00342 | 9.957E-06 | 337199 |
| rs35639297 | chr12:56142553 | T | G | Cause of death: car occupant injured in unspecified traffic accident | UKBB | 0.0204 | 1.358E-09 | 7637 |
| rs144857205 | chr12:56143668 | A | G | Self-reported atrial fibrillation | UKBB | 0.01382 | 3.458E-06 | 337159 |
| rs144857205 | chr12:56143668 | A | G | Treatment with promethazine product | UKBB | 0.002095 | 2.697E-06 | 337159 |
| rs144857205 | chr12:56143668 | A | G | Cause of death: cardiomegaly | UKBB | 0.05667 | 1.493E-06 | 7637 |
| rs545328899 | chr12:56144359 | C | G | Abnormalities of gait and mobility | UKBB | -0.00246 | 3.256E-07 | 337199 |
| rs139996303 | chr12:56144742 | A | G | Self-reported respiratory infection | UKBB | -0.00105 | 5.461E-06 | 337159 |
| rs139996303 | chr12:56144742 | A | G | Cause of death: chronic or unspecified with haemorrhage | UKBB | -0.01729 | 2.802E-06 | 7637 |
| rs139996303 | chr12:56144742 | A | G | Cause of death: other specified place | UKBB | -0.01749 | 1.63E-07 | 7637 |
| rs560154222 | chr12:56144747 | A | G | Treatment with viagra 25mg tablet | UKBB | -0.00307 | 3.149E-07 | 337159 |
| rs560154222 | chr12:56144747 | A | G | Cause of death: multiple sclerosis | UKBB | -0.08241 | 5.445E-07 | 7637 |
| rs560154222 | chr12:56144747 | A | G | Cause of death: hypertensive heart disease without heart failure | UKBB | -0.0837 | 2.475E-06 | 7637 |
| rs141733058 | chr12:56144800 | C | G | Cause of death: appendix | UKBB | -0.01674 | 9.195E-07 | 7637 |
| rs571587078 | chr12:56146094 | A | T | Cause of death: car occupant injured in unspecified traffic accident | UKBB | -0.02089 | 8.875E-10 | 7637 |
| rs7297523 | chr12:56147315 | A | G | Self-reported pulmonary fibrosis | UKBB | 0.000456 | 8.991E-06 | 337159 |
| rs12304296 | chr12:56148726 | A | G | Self-reported pulmonary fibrosis | UKBB | 0.000457 | 8.686E-06 | 337159 |
| rs547761429 | chr12:56148936 | C | G | Malignant neoplasm of pancreas | UKBB | -0.00298 | 7.516E-06 | 337199 |
| rs547761429 | chr12:56148936 | C | G | Multiple valve diseases | UKBB | -0.00328 | 6.46E-08 | 337199 |
| rs145612377 | chr12:56150611 | A | C | Self-reported uterine polyps | UKBB | 0.006947 | 5.65E-06 | 337159 |
| rs145612377 | chr12:56150611 | A | C | Cause of death: intrahepatic bile duct carcinoma | UKBB | 0.1016 | 1.256E-06 | 7637 |
| rs145612377 | chr12:56150611 | A | C | Cause of death: pneumonitis due to food and vomit | UKBB | 0.03463 | 1.761E-08 | 7637 |
| rs145612377 | chr12:56150611 | A | C | Cause of death: alcoholic liver disease, unspecified | UKBB | 0.1216 | 5.392E-17 | 7637 |
| rs145612377 | chr12:56150611 | A | C | Cause of death: home | UKBB | 0.03529 | 3.675E-13 | 7637 |
| rs145612377 | chr12:56150611 | A | C | Unspecified jaundice | UKBB | 0.00331 | 1.559E-06 | 337199 |

**Supplementary Table S8. Associations with *GDF11* variants identified in PhenoScanner.** A p-value less than 1e-5 was considered statistically significant. A1 = the effect allele; A2 = the non-effect allele.

| **Trait** | **Variant** | **Position** | **-log10(p-value)** |
| --- | --- | --- | --- |
| J40-J47 Chronic lower respiratory diseases | rs1702877 | 56427808 | 10.48192057 |
| N99-N99 Other disorders of the genitourinary system | rs7136420 | 56578682 | 15.94332378 |
| asthma | rs1689510 | 56396768 | 22.13053359 |
| J45 Asthma | rs1689510 | 56396768 | 11.32382786 |
| hypothyroidism/myxoedema | rs705702 | 56390636 | 15.02663658 |
| Platelet distribution width | rs2950387 | 57021006 | 218.3157023 |
| Comparative height size at age 10 | rs2277339 | 57146069 | 11.78080856 |
| Mean reticulocyte volume | rs2277339 | 57146069 | 15.419828 |
| Reticulocyte percentage | rs2950390 | 57055291 | 8.410128556 |
| High light scatter reticulocyte count | rs11171911 | 57017515 | 10.74994613 |
| Mean sphered cell volume | rs2277339 | 57146069 | 22.22634518 |
| High light scatter reticulocyte percentage | rs9634277 | 57034694 | 11.08611201 |
| E03 Other hypothyroidism | rs705699 | 56384804 | 8.803436965 |
| Lymphocyte count | rs1131017 | 56435929 | 12.10080897 |
| Monocyte count | rs2277339 | 57146069 | 10.61957167 |
| Eosinophill count | rs1689510 | 56396768 | 36.42416581 |
| Lymphocyte percentage | rs1131017 | 56435929 | 9.549673896 |
| Eosinophill percentage | rs1689510 | 56396768 | 46.03330929 |
| Platelet count | rs2950387 | 57021006 | 83.39013891 |
| Mean platelet (thrombocyte) volume | rs10783794 | 56987179 | 300 |
| C43 Malignant melanoma of skin | rs78874486 | 56060691 | 9.085080635 |
| Mean corpuscular haemoglobin concentration | rs2657910 | 56891649 | 8.100037309 |
| Platelet crit | rs2277339 | 57146069 | 11.079355 |
| Mean corpuscular haemoglobin | rs2277339 | 57146069 | 21.46354257 |
| Leg fat percentage (right) | rs2292238 | 56493822 | 9.173614172 |
| Mean corpuscular volume | rs2277339 | 57146069 | 29.16703149 |
| Red blood cell (erythrocyte) count | rs2657910 | 56891649 | 16.08319349 |
| carpal tunnel syndrome | rs576367683 | 55927109 | 8.994476307 |
| Haematocrit percentage | rs2657910 | 56891649 | 9.239909396 |
| Whole body fat mass | rs2292238 | 56493822 | 10.38874416 |
| Trunk predicted mass | rs2277339 | 57146069 | 23.97872799 |
| Trunk fat-free mass | rs2277339 | 57146069 | 24.12930939 |
| Trunk fat mass | rs3759094 | 56497903 | 8.088837063 |
| Arm predicted mass (left) | rs2069408 | 56364321 | 18.45268074 |
| Arm fat-free mass (left) | rs2069408 | 56364321 | 18.0691579 |
| Arm fat mass (left) | rs2292238 | 56493822 | 11.97240579 |
| Arm fat percentage (left) | rs2292238 | 56493822 | 9.581234825 |
| Arm predicted mass (right) | rs2069408 | 56364321 | 15.92657494 |
| Arm fat-free mass (right) | rs4759228 | 56508409 | 16.04303961 |
| Arm fat mass (right) | rs2292238 | 56493822 | 12.60586365 |
| Arm fat percentage (right) | rs2292238 | 56493822 | 9.92955575 |
| Leg predicted mass (left) | rs4759228 | 56508409 | 15.69751755 |
| Leg fat-free mass (left) | rs4759228 | 56508409 | 15.73985703 |
| Leg fat mass (left) | rs2292238 | 56493822 | 11.28217977 |
| Leg fat percentage (left) | rs2292238 | 56493822 | 9.487876173 |
| Impedance of arm (right) | rs3741499 | 56474379 | 18.58876763 |
| Leg predicted mass (right) | rs2069408 | 56364321 | 16.7144877 |
| Leg fat-free mass (right) | rs2069408 | 56364321 | 16.89032023 |
| Impedance of whole body | rs4759228 | 56508409 | 21.25662352 |
| Whole body fat-free mass | rs2277339 | 57146069 | 19.68317031 |
| Leg fat mass (right) | rs2292238 | 56493822 | 11.40918045 |
| Impedance of arm (left) | rs3741499 | 56474379 | 18.86713221 |
| hayfever/allergic rhinitis | rs34415530 | 56444632 | 8.265000265 |
| Impedance of leg (left) | rs4759228 | 56508409 | 18.00488715 |
| Impedance of leg (right) | rs2069408 | 56364321 | 16.89106308 |
| Basal metabolic rate | rs2069408 | 56364321 | 17.42603822 |
| Body mass index (BMI) | rs2292238 | 56493822 | 16.46986436 |
| Whole body water mass | rs2277339 | 57146069 | 19.72129212 |
| Weight | rs3759094 | 56497903 | 14.72056121 |
| Sitting height | rs11171803 | 56718422 | 10.25687453 |
| Body mass index (BMI) | rs2292238 | 56493822 | 17.26242573 |
| Weight | rs3759094 | 56497903 | 16.56791301 |
| Standing height | rs59917308 | 56658708 | 27.45500232 |
| Hip circumference | rs2292238 | 56493822 | 9.373793124 |
| Waist circumference | rs3759094 | 56497903 | 11.78536805 |
| H18 Other disorders of cornea | rs145519382 | 56852029 | 9.503665494 |
| thyroid problem (not cancer) | rs705702 | 56390636 | 10.22374205 |
| N99 Postprocedural disorders of genito-urinary system, not elsewhere classified | rs7136420 | 56578682 | 15.94332378 |

**Supplementary Table S9. Traits associated with GDF11 variants in GeneATLAS.** Only associations that met the threshold (p < 1e-08) are listed in the table.

| **Study ID** | **Reported Trait** | **PMID** | **Author** | **Date** | **Study N Initial** | **Index Variant ID** | **Index Variant RSID** | **P-Value** | **Beta** |
| --- | --- | --- | --- | --- | --- | --- | --- | --- | --- |
| GCST004630 | Mean corpuscular hemoglobin | PMID:27863252 | Astle WJ | 2016-11-17 | 172332 | 2_189568259_C_T | rs112257498 | 1.025E-09 | -0.02233584 |
| GCST90002326 | Mean corpuscular hemoglobin | PMID:32888493 | Chen MH | 2020-09-01 | 630125 | 2_189568259_C_T | rs112257498 | 9E-17 |  |
| GCST90002334 | Mean corpuscular volume | PMID:32888493 | Chen MH | 2020-09-01 | 544127 | 2_189568259_C_T | rs112257498 | 1.71E-08 | -0.010975 |
| GCST90002338 | Mean corpuscular volume | PMID:32888493 | Chen MH | 2020-09-01 | 696882 | 2_189568259_C_T | rs112257498 | 2E-10 |  |
| GCST90018964 | Mean corpuscular hemoglobin | PMID:34594039 | Sakaue S | 2021-09-30 | 478500 | 2_189568259_C_T | rs112257498 | 3E-13 | -0.0157 |
| GCST90002304 | Hematocrit | PMID:32888493 | Chen MH | 2020-09-01 | 562259 | 2_189581815_C_G | rs3811621 | 2.23E-11 | 0.014038 |
| GCST011427_541 | Protein quantitative trait loci (liver) [SDHB] | PMID:32778093 | He B | 2020-08-10 | 287 | 2_189587523_A_C | rs146361420 | 1E-08 | 0.1952 |
| GCST011427_617 | Protein quantitative trait loci (liver) [VDAC1] | PMID:32778093 | He B | 2020-08-10 | 287 | 2_189587523_A_C | rs146361420 | 3E-08 | 0.3086 |
| GCST007388 | Insomnia symptoms (never/rarely vs. usually) | PMID:30804566 | Lane JM | 2019-02-25 | 237627 | 2_190424107_A_G | rs4577309 | 1E-09 |  |
| GCST004605 | Mean corpuscular hemoglobin concentration | PMID:27863252 | Astle WJ | 2016-11-17 | 172851 | 2_189576591_A_G | rs2882877 | 7.605E-09 | 0.0209684 |
| GCST90002308 | Hematocrit | PMID:32888493 | Chen MH | 2020-09-01 | 737823 | 2_189587075_T_C | rs17198990 | 4E-12 |  |
| GCST90012022 | Fibroblast growth factor 23 levels | PMID:33067605 | Folkersen L | 2020-10-16 | 21758 | 2_189588022_C_A | rs6706281 | 2.429E-18 | -0.1057 |
| GCST010084 | Leisure sedentary behaviour (television watching) | PMID:32317632 | van de Vegte YJ | 2020-04-21 | 408815 | 2_190424107_A_G | rs4577309 | 2E-13 |  |
| GCST90025948 | Serum phosphate levels | PMID:34226706 | Barton AR | 2021-07-05 | 400159 | 2_190457000_G_A | rs62181017 | 3.1E-16 | -0.0182445 |
| GCST007876 | Estimated glomerular filtration rate | PMID:31015462 | Graham SE | 2019-04-23 | 350514 | 2_190413615_G_A | rs6725814 | 3E-08 | -5.52 |
| GCST90019516 | Serum phosphate levels | PMID:33462484 | Sinnott-Armstrong N | 2021-01-18 | 325141 | 2_190413615_G_A | rs6725814 | 6E-12 | 0.0176 |
| NEALE2_1448_1 | White | bread type |  | UKB Neale v2 | 2018-08-01 | 348424 | 2_190408694_GCTCCCTCC_G | rs374314861 | 9.70306E-09 |  |
| GCST90002334 | Mean corpuscular volume | PMID:32888493 | Chen MH | 2020-09-01 | 544127 | 2_189601000_C_T | rs11683621 | 4.53E-08 | 0.014059 |
| NEALE2_30070_raw | Red blood cell (erythrocyte) distribution width |  | UKB Neale v2 | 2018-08-01 | 350473 | 2_189571446_A_T | rs1371467 | 2.78625E-09 | 0.0142713 |
| GCST007387 | Insomnia symptoms (never/rarely vs. sometimes/usually) | PMID:30804566 | Lane JM | 2019-02-25 | 453379 | 2_190424107_A_G | rs4577309 | 9E-09 |  |
| GCST007074 | Red cell distribution width | PMID:30595370 | Kichaev G | 2018-12-27 | 445000 | 2_189558279_G_A | rs12478088 | 2E-08 |  |
| GCST90018966 | Mean corpuscular volume | PMID:34594039 | Sakaue S | 2021-09-30 | 480305 | 2_189558370_T_C | rs12466208 | 1E-09 | -0.0132 |
| GCST90002400 | Plateletcrit | PMID:32888494 | Vuckovic D | 2020-09-01 | 408112 | 2_190449176_T_G | rs6742904 | 1.1E-12 | 0.0161934 |
| GCST002578 | Ferritin levels | PMID:25162662 | Liao M | 2014-08-27 | 1999 | 2_189784590_G_C | rs5742933 | 2E-10 |  |
| GCST007932 | Medication use (thyroid preparations) | PMID:31015401 | Wu Y | 2019-04-23 | 305582 | 2_190398199_T_C | rs10202630 | 3E-09 | 0.05442924 |
| GCST90010715 | Arthritis (juvenile idiopathic) | PMID:33106285 | Lopez-Isac E | 2020-10-26 | 12501 | 2_190398036_G_C | rs6434390 | 1E-09 |  |
| GCST007071 | Autoimmune traits | PMID:30595370 | Kichaev G | 2018-12-27 | 459000 | 2_190398199_T_C | rs10202630 | 4E-08 |  |
| GCST90002372 | Red cell distribution width | PMID:32888493 | Chen MH | 2020-09-01 | 563352 | 2_189647251_T_C | rs62183651 | 8E-10 |  |
| GCST007081 | Lung function (FVC) | PMID:30595370 | Kichaev G | 2018-12-27 | 372000 | 2_190504195_G_C | rs10209172 | 5E-09 |  |
| NEALE2_21021_raw | Pulse wave arterial stiffness index |  | UKB Neale v2 | 2018-08-01 | 118469 | 2_190465728_C_T | rs183595018 | 2.435E-12 | 0.808918 |
| GCST90002404 | Red cell distribution width | PMID:32888494 | Vuckovic D | 2020-09-01 | 408112 | 2_189571446_A_T | rs1371467 | 6.3E-10 | 0.0139613 |
| NEALE2_30090_raw | Platelet crit |  | UKB Neale v2 | 2018-08-01 | 350471 | 2_190384121_C_T | rs75770358 | 4.9108E-11 | 0.000794662 |
| GCST011995 | Restless legs syndrome | PMID:33239738 | Didriksen M | 2020-11-25 | 480982 | 2_189584800_A_T | rs10188680 | 5E-08 |  |
| GCST007068 | Mean corpuscular hemoglobin | PMID:30595370 | Kichaev G | 2018-12-27 | 443000 | 2_189587860_T_C | rs6718798 | 2E-12 |  |
| GCST90018969 | Platelet count | PMID:34594039 | Sakaue S | 2021-09-30 | 499097 | 2_190534112_C_T | rs61730953 | 5E-08 | 0.0123 |
| GCST90016674 | Liver iron content | PMID:34128465 | Liu Y | 2021-06-15 | 32858 | 2_189653884_C_T | rs7577758 | 3.6E-11 | 0.0606348 |
| GCST90025962 | Mean corpuscular hemoglobin concentration | PMID:34226706 | Barton AR | 2021-07-05 | 443081 | 2_189600010_G_A | rs6733858 | 3.4E-11 | -0.0123119 |
| GCST90002357 | Platelet count | PMID:32888493 | Chen MH | 2020-09-01 | 542827 | 2_190474797_G_C | rs79431248 | 1.23E-10 | 0.012323 |
| GCST012398 | Calcium levels | PMID:33887147 | Young WJ | 2021-04-22 | 305349 | 2_190479037_T_C | rs12613807 | 4E-11 | 0.001483 |
| GCST90018951 | Calcium levels | PMID:34594039 | Sakaue S | 2021-09-30 | 399133 | 2_190492968_A_T | rs35095338 | 3E-11 |  |
| GCST003119 | Urinary metabolites [1.067 ppm/1.049 ppm] | PMID:26352407 | Raffler J | 2015-09-09 | 3861 | 2_190340773_G_C | rs13006833 | 2E-20 |  |
| GCST90025951 | Platelet count | PMID:34226706 | Barton AR | 2021-07-05 | 444866 | 2_190436642_A_G | rs9646748 | 6.9E-09 | -0.0113623 |
| GCST009733_190 | Urinary metabolite levels in chronic kidney disease [methylmalonate (MMA] | PMID:31959995 | Schlosser P | 2020-01-20 | 1221 | 2_190323393_A_G | rs291468 | 2E-14 | 0.38 |
| GCST011427_305 | Protein quantitative trait loci (liver) [HIBCH] | PMID:32778093 | He B | 2020-08-10 | 287 | 2_190362016_A_G | rs2067416 | 3E-14 | -0.1465 |
| GCST011427_305 | Protein quantitative trait loci (liver) [HIBCH] | PMID:32778093 | He B | 2020-08-10 | 287 | 2_190283621_G_A | rs2664252 | 7E-36 | 0.2191 |
| NEALE2_20015_raw | Sitting height |  | UKB Neale v2 | 2018-08-01 | 360066 | 2_189581210_C_T | rs13015236 | 1.5589E-10 | 0.0572015 |
| GCST90025981 | Diastolic blood pressure | PMID:34226706 | Barton AR | 2021-07-05 | 422713 | 2_190514542_G_A | rs4586658 | 5.4E-13 | 0.0151022 |
| GCST90025990 | Calcium levels | PMID:34226706 | Barton AR | 2021-07-05 | 400792 | 2_190479037_T_C | rs12613807 | 1.5E-13 | 0.0158636 |
| GCST90002369 | Red cell distribution width | PMID:32888493 | Chen MH | 2020-09-01 | 531774 | 2_189571446_A_T | rs1371467 | 3.06E-11 | 0.013653 |
| GCST006585_836 | Blood protein levels [HIBCH, 12396_19_3] | PMID:30072576 | Emilsson V | 2018-08-02 | 3200 | 2_190312279_A_C | rs291447 | 3E-152 | 0.6305172 |
| GCST90002369 | Red cell distribution width | PMID:32888493 | Chen MH | 2020-09-01 | 531774 | 2_189647251_T_C | rs62183651 | 1.02E-10 | 0.017578 |
| GCST90086956 | Serum levels of protein HIBCH | PMID:35078996 | Gudjonsson A | 2022-01-25 | 5367 | 2_190311497_T_C | rs291444 | 2E-258 | 0.604929 |
| GCST90002402 | Platelet count | PMID:32888494 | Vuckovic D | 2020-09-01 | 408112 | 2_190474797_G_C | rs79431248 | 7E-10 | 0.0129799 |
| NEALE2_30010_raw | Red blood cell (erythrocyte) count |  | UKB Neale v2 | 2018-08-01 | 350475 | 2_189559059_T_C | rs2352262 | 7.07205E-09 | 0.00503794 |
| GCST011427_305 | Protein quantitative trait loci (liver) [HIBCH] | PMID:32778093 | He B | 2020-08-10 | 287 | 2_190366777_G_A | rs4940 | 2E-16 | -0.1712 |
| GCST011427_305 | Protein quantitative trait loci (liver) [HIBCH] | PMID:32778093 | He B | 2020-08-10 | 287 | 2_190300703_C_T | rs60469543 | 5E-15 | -0.167 |
| GCST011427_305 | Protein quantitative trait loci (liver) [HIBCH] | PMID:32778093 | He B | 2020-08-10 | 287 | 2_190297119_C_T | rs2582743 | 2E-20 | 0.1867 |
| GCST004601 | Red blood cell count | PMID:27863252 | Astle WJ | 2016-11-17 | 172952 | 2_189568259_C_T | rs112257498 | 5.843E-09 | 0.0213889 |
| GCST006866 | Lung cancer (SNP x SNP interaction) | PMID:24325914 | Chu M | 2013-12-09 | 5408 | 2_190248283_T_G | rs2562796 | 1E-13 |  |
| GCST90002386 | High light scatter reticulocyte percentage of red cells | PMID:32888494 | Vuckovic D | 2020-09-01 | 408112 | 2_190195823_G_A | rs58052205 | 7.4E-11 | -0.0499714 |
| GCST90000059 | Diastolic blood pressure | PMID:33230300 | Surendran P | 2020-11-23 | 810865 | 2_189962297_A_G | rs2053163 | 2.783E-10 | -0.010878665 |
| GCST011427_305 | Protein quantitative trait loci (liver) [HIBCH] | PMID:32778093 | He B | 2020-08-10 | 287 | 2_190200027_C_T | rs6753459 | 2E-21 | -0.1928 |
| GCST011427_305 | Protein quantitative trait loci (liver) [HIBCH] | PMID:32778093 | He B | 2020-08-10 | 287 | 2_190248527_T_C | rs7590991 | 4E-15 | 0.148 |
| GCST90025972 | Reticulocyte count | PMID:34226706 | Barton AR | 2021-07-05 | 437291 | 2_190195543_C_T | rs6755406 | 1.3E-08 | -0.0372631 |
| GCST90002385 | High light scatter reticulocyte count | PMID:32888494 | Vuckovic D | 2020-09-01 | 408112 | 2_190184677_G_A | rs191148279 | 3.3E-13 | 0.0703951 |
| GCST90002386 | High light scatter reticulocyte percentage of red cells | PMID:32888494 | Vuckovic D | 2020-09-01 | 408112 | 2_190184677_G_A | rs191148279 | 2.1E-13 | 0.071074 |
| GCST90002405 | Reticulocyte count | PMID:32888494 | Vuckovic D | 2020-09-01 | 408112 | 2_190184677_G_A | rs191148279 | 1.1E-10 | 0.0627395 |
| GCST90002406 | Reticulocyte fraction of red cells | PMID:32888494 | Vuckovic D | 2020-09-01 | 408112 | 2_190184677_G_A | rs191148279 | 1.3E-11 | 0.0659613 |
| NEALE2_30050_raw | Mean corpuscular haemoglobin |  | UKB Neale v2 | 2018-08-01 | 350472 | 2_189568259_C_T | rs112257498 | 2.05127E-08 | -0.0253044 |
| GCST90025970 | High light scatter reticulocyte count | PMID:34226706 | Barton AR | 2021-07-05 | 437723 | 2_190195543_C_T | rs6755406 | 2.2E-11 | -0.0428299 |
| GCST90025974 | Hemoglobin A1c levels | PMID:34226706 | Barton AR | 2021-07-05 | 437749 | 2_190195543_C_T | rs6755406 | 2.5E-09 | 0.0362227 |
| GCST007037 | Educational attainment (years of education) | PMID:30595370 | Kichaev G | 2018-12-27 | 455000 | 2_190142308_G_C | rs11693404 | 2E-08 |  |

**Supplementary Table S10. Associations with *MSTN* variants identified in the OpenTarget Genetics Portal.** A p-value equal to or less than 5*e*−8 was considered statistically significant.

| **rsid** | **hg19_coordinates** | **a1** | **a2** | **trait** | **pmid** | **beta** | **p** | **n** |
| --- | --- | --- | --- | --- | --- | --- | --- | --- |
| rs377503267 | chr2:190920622 | T | C | Cause of death: chronic myeloid leukaemia | UKBB | -0.02066 | 7.029E-07 | 7637 |
| rs3791782 | chr2:190920652 | T | C | Self-reported lichen planus | UKBB | -0.0037 | 1.187E-06 | 337159 |
| rs3791782 | chr2:190920652 | T | C | Cause of death: cardiac | UKBB | -0.08957 | 3.033E-08 | 7637 |
| rs7596995 | chr2:190923344 | T | C | Self-reported lichen planus | UKBB | 0.003607 | 1.449E-06 | 337159 |
| rs7596995 | chr2:190923344 | T | C | Cause of death: cardiac | UKBB | 0.08124 | 1.749E-07 | 7637 |
| rs115760476 | chr2:190923503 | A | G | Home area population density: postcode not linkable | UKBB | 0.000689 | 4.85E-09 | 333997 |
| rs115760476 | chr2:190923503 | A | G | Cause of death: acute lymphoblastic leukaemia | UKBB | 0.02115 | 5.117E-07 | 7637 |
| rs115760476 | chr2:190923503 | A | G | Cause of death: organ-limited amyloidosis | UKBB | 0.02145 | 2.427E-06 | 7637 |
| rs115760476 | chr2:190923503 | A | G | Cause of death: acute and subacute infective endocarditis | UKBB | 0.02187 | 1.311E-08 | 7637 |
| rs62186768 | chr2:190924010 | A | C | Cause of death: pharynx, unspecified | UKBB | -0.01119 | 2.131E-06 | 7637 |
| rs146209367 | chr2:190924130 | T | C | Cause of death: cervix uteri, unspecified | UKBB | -0.01466 | 3.366E-06 | 7637 |
| rs180921345 | chr2:190924506 | T | C | Doctor diagnosed idiopathic pulmonary fibrosis | UKBB | 0.007152 | 2.798E-06 | 83529 |
| rs180921345 | chr2:190924506 | T | C | Cause of death: acute and subacute infective endocarditis | UKBB | 0.02247 | 9.856E-09 | 7637 |
| rs180921345 | chr2:190924506 | T | C | Cause of death: subarachnoid haemorrhage from intracranial artery, unspecified | UKBB | 0.02241 | 1.325E-06 | 7637 |
| rs188760163 | chr2:190926549 | T | C | Self-reported chronic skin ulcers | UKBB | -0.00183 | 9.915E-09 | 337159 |
| rs188760163 | chr2:190926549 | T | C | Treatment with ferrous salt product | UKBB | -0.00147 | 7.024E-06 | 337159 |
| rs188760163 | chr2:190926549 | T | C | Treatment with indivina 1mg or 2.5mg tablet | UKBB | -0.00146 | 4.346E-06 | 337159 |
| rs188760163 | chr2:190926549 | T | C | Systemic sclerosis | UKBB | -0.00111 | 6.903E-07 | 337199 |
| rs138343163 | chr2:190927060 | T | C | Self-reported unclassifiable cancer | UKBB | 0.004537 | 8.535E-06 | 337159 |
| rs138343163 | chr2:190927060 | T | C | Cause of death: acute vascular disorders of intestine | UKBB | 0.04175 | 3.267E-08 | 7637 |

**Supplementary Table S11. Associations with *MSTN* variants identified in PhenoScanner.** A p-value less than 1e-5 was considered statistically significant. A1 = the effect allele; A2 = the non-effect allele.

| **Trait** | **Variant** | **Position** | **-log10(p-value)** |
| --- | --- | --- | --- |
| F33 Recurrent depressive disorder | rs561423669 | 191853391 | 9.272117297 |
| E00-E07 Disorders of thyroid gland | rs3024859 | 191925424 | 16.6500235 |
| hypothyroidism/myxoedema | rs3024859 | 191925424 | 24.49023313 |
| Comparative height size at age 10 | rs12693589 | 191832662 | 9.520568663 |
| Immature reticulocyte fraction | rs11687659 | 191741197 | 22.62172932 |
| Reticulocyte percentage | rs1882398 | 191724460 | 12.98741584 |
| Reticulocyte count | rs1882398 | 191724460 | 15.31606186 |
| High light scatter reticulocyte count | rs1882398 | 191724460 | 24.99567863 |
| High light scatter reticulocyte percentage | rs1882398 | 191724460 | 21.84820128 |
| Mean platelet (thrombocyte) volume | rs12693588 | 191832492 | 26.41668785 |
| Platelet crit | rs34943464 | 191251875 | 11.78096589 |
| Mean corpuscular haemoglobin | rs6718798 | 190452586 | 12.76235574 |
| Red blood cell (erythrocyte) distribution width | rs12478088 | 190423005 | 9.12932109 |
| Red blood cell (erythrocyte) count | rs17271134 | 190389803 | 11.31309573 |
| Trunk predicted mass | rs7561002 | 189982418 | 8.991953694 |
| Trunk fat-free mass | rs7561002 | 189982418 | 9.046452918 |
| E03 Other hypothyroidism | rs3024859 | 191925424 | 15.90486553 |
| Impedance of arm (right) | rs13391332 | 189979720 | 9.469787491 |
| Impedance of whole body | rs2119069 | 190354871 | 13.34005529 |
| Impedance of arm (left) | rs2119069 | 190354871 | 11.91492359 |
| Impedance of leg (left) | rs1973666 | 190062633 | 11.76710549 |
| Impedance of leg (right) | rs1973666 | 190062633 | 11.16337211 |
| Sitting height | rs114885387 | 190146210 | 16.47402279 |
| thyroid problem (not cancer) | rs3024859 | 191925424 | 26.78954771 |
| Standing height | rs35917062 | 191660828 | 19.87582194 |
| Time spent watching television (TV) | rs4577309 | 191288833 | 12.26696332 |

**Supplementary Table S12. Associations with *MSTN* variants identified in GeneATLAS.** Only associations that met the threshold (p < 1e-08) are listed in the table.

| **Trait** | **Avg chi^2^ ratio** | **Avg chi^2^** | **Max chi^2^** | **Z-score** |
| --- | --- | --- | --- | --- |
| Overall health rating | 11.3 | 24.3 | 24.3 | 4.9 |
| Loneliness, isolation | 11.2 | 15.5 | 15.5 | 3.9 |
| Back pain experienced in last month | 11.2 | 16.3 | 16.3 | 4 |
| Smoking Status | 11 | 26.4 | 26.4 | 5.1 |
| Respiratory disease | 10 | 22.2 | 22.2 | -4.7 |
| Neck or shoulder pain in last month | 9 | 12.8 | 12.8 | 3.6 |
| Allergy | 8.6 | 19.8 | 19.8 | 4.5 |
| Hypothyroidism/myxoedema (self-reported) | 8.5 | 16.6 | 16.6 | -4.1 |
| Past tobacco smoking | 8.4 | 15.5 | 15.5 | -3.9 |
| Hypothyroidism (self reported) | 8.2 | 18.8 | 18.8 | -4.3 |
| Asthma (self-reported) | 8.2 | 16.4 | 16.4 | -4 |
| Smoking status: Previous | 8 | 12 | 12 | 3.5 |
| Had other major operations | 7.9 | 9.6 | 9.6 | -3.1 |
| Qualifications: College or University degree | 7.9 | 24.8 | 24.8 | -5 |
| Myopia | 7.5 | 10.8 | 10.8 | 3.3 |
| Pain experienced in last month | 7.4 | 12.4 | 12.4 | -3.5 |
| Asthma | 7.2 | 14.2 | 14.2 | -3.8 |
| Verbal and Numeric Reasoning (VNR) | 6.8 | 16.4 | 16.4 | -4.1 |
| Allergy or Eczema | 6.7 | 17.6 | 17.6 | -4.2 |
| Cardiovascular Disease | 6.5 | 21.6 | 21.6 | 4.6 |
| Vascular/heart problems diagnosed by doctor | 6.4 | 17.3 | 17.3 | -4.2 |
| Age when periods started (menarche) | 6.2 | 14.8 | 14.8 | -3.8 |
| Hypertension (Self-reported) | 6.1 | 17 | 17 | 4.1 |
| High blood pressure | 6 | 16.6 | 16.6 | 4.1 |
| Vitamin and mineral supplements | 5.7 | 7.8 | 7.8 | -2.8 |
| Reproduciblity of spirometry measurement using ERS/ATS criteria | 5.6 | 6.7 | 6.7 | -2.6 |
| Birth weight | 5.5 | 11.8 | 11.8 | -3.4 |
| Hayfever/allergic rhinitis (self-reported) | 5.4 | 7.2 | 7.2 | -2.7 |
| Chronic bronchitis/emphysema (mother) | 5.4 | 6.3 | 6.3 | -2.5 |
| Exposure to tobacco smoke outside home | 5.2 | 6.9 | 6.9 | 2.6 |
| Schizophrenia vs Biploar Disorder | 5.1 | 6.7 | 6.7 | 2.6 |
| Sensitivity / hurt feelings | 5.1 | 8.7 | 8.7 | 3 |
| Medication: Atorvastatin | 5.1 | 6.3 | 6.3 | 2.5 |
| Reason for reducing amount of alcohol drunk: Health precaution | 5 | 5.9 | 5.9 | -2.4 |
| Eye problems/disorders: Diabetes related eye disease | 4.9 | 5.4 | 5.4 | -2.3 |
| Age at last live birth | 4.9 | 7.1 | 7.1 | -2.7 |
| Stomach or abdominal pain in last month | 4.8 | 6 | 6 | 2.4 |
| Qualifications: None of the above | 4.8 | 10.2 | 10.2 | 3.2 |
| Mouth/teeth dental problems: Bleeding gums | 4.6 | 6 | 6 | 2.5 |
| Rheumatoid Arthritis | 4.5 | 6.2 | 6.2 | 2.5 |
| Frequency of unenthusiasm / disinterest in last 2 weeks | 4.5 | 6 | 6 | 2.5 |
| Hayfever, allergic rhinitis or eczema | 4.5 | 9.7 | 9.7 | -3.1 |
| Medication: Levothyroxine sodium | 4.4 | 7.4 | 7.4 | -2.7 |
| Guilty feelings | 4.3 | 6.5 | 6.5 | 2.6 |
| Medication: Paracetamol | 4.3 | 6.6 | 6.6 | 2.6 |
| Fluid intelligence score | 4.2 | 8.8 | 8.8 | -3 |
| Average weekly champagne plus white wine intake | 4.2 | 5.8 | 5.8 | -2.4 |
| Depression (Nagel 2018) | 4 | 6 | 6 | 2.4 |
| Intelligence (Savage-Jansen 2018) | 4 | 12.2 | 12.2 | -3.5 |
| Frequency of tenseness / restlessness in last 2 weeks | 4 | 6.2 | 6.2 | 2.5 |
| Qualifications: A levels/AS levels or equivalent | 4 | 8.9 | 8.9 | -3 |
| Medication: Ventolin 100micrograms inhaler | 4 | 5.1 | 5.1 | -2.3 |
| Mean Putamen Volume | 3.9 | 4.5 | 4.5 | -2.1 |
| Mouth/teeth dental problems: Mouth ulcers | 3.9 | 6.1 | 6.1 | 2.5 |
| Mouth/teeth dental problems | 3.8 | 5.9 | 5.9 | -2.4 |
| Ever smoked | 3.6 | 6.4 | 6.4 | 2.5 |
| Length of working week for main job | 3.5 | 3.9 | 3.9 | 2 |
| Qualifications: CSEs or equivalent | 3.4 | 4.4 | 4.4 | 2.1 |
| Frequency of tiredness / lethargy in last 2 weeks | 3.4 | 5.5 | 5.5 | 2.3 |
| Age at first live birth | 3.4 | 6 | 6 | -2.4 |
| Impedance of arm (left) | 3.3 | 15.1 | 15.1 | -3.9 |
| Birth weight of first child | 3.2 | 5.8 | 5.8 | -2.4 |
| Medication: Paracetamol | 3.2 | 5.2 | 5.2 | 2.3 |
| High cholesterol (Self-reported) | 3.2 | 5.7 | 5.7 | 2.4 |
| Blood Eosinophil Count | 3.1 | 23.7 | 23.7 | -4.9 |
| Knee pain experienced in last month | 3.1 | 4.8 | 4.8 | 2.2 |
| Qualifications: nursing, teaching | 3.1 | 4.9 | 4.9 | -2.2 |
| Heart disease (siblings) | 3.1 | 3.8 | 3.8 | 1.9 |
| Type 2 Diabetes (T2D) (2012) | 3 | 3.4 | 3.4 | 1.8 |
| Lung FEV1/FVC ratio | 3 | 15.4 | 15.4 | 3.9 |
| Impedance of arm (right) | 3 | 13.9 | 13.9 | -3.7 |
| Ever had bowel cancer screening | 2.9 | 3.4 | 3.4 | 1.8 |
| Medication: Ramipril | 2.8 | 3.5 | 3.5 | 1.9 |
| Ever taken oral contraceptive pill | 2.8 | 3.1 | 3.1 | -1.8 |
| BMI | 2.5 | 14.3 | 14.3 | 3.8 |
| Depression (self-reported) | 2.5 | 3 | 3 | 1.7 |
| Smoking status: Current | 2.5 | 4 | 4 | 2 |
| Neuroticism score | 2.4 | 5 | 5 | 2.2 |
| Number of live births | 2.4 | 3.4 | 3.4 | -1.8 |
| High blood pressure (siblings) | 2.4 | 3.5 | 3.5 | 1.9 |
| Impedance of whole body | 2.4 | 12.5 | 12.5 | -3.5 |
| Bipolar Disorder (2011) | 2.3 | 2.2 | 2.2 | -1.5 |
| Illnesses of mother | 2.3 | 2.8 | 2.8 | 1.7 |
| Alcohol intake frequency. | 2.3 | 5.3 | 5.3 | 2.3 |
| Medication: Laxatives (e.g. Dulcolax, Senokot) | 2.2 | 2.6 | 2.6 | 1.6 |
| Age started oral contraceptive pill | 2.2 | 2.7 | 2.7 | -1.6 |
| Neuroticism (Nagel 2018) | 2.1 | 5 | 5 | 2.2 |
| Worry (Nagel 2018) | 2.1 | 4.4 | 4.4 | 2.1 |
| Primary Biliary Cirrhosis | 2.1 | 3 | 3 | 1.7 |
| Medication for pain relief, constipation, heartburn | 2.1 | 3.6 | 3.6 | -1.9 |
| Illnesses of siblings: Diabetes | 2.1 | 2.6 | 2.6 | 1.6 |
| Medication: Blood pressure | 2.1 | 4 | 4 | 2 |
| Unable to work because of sickness or disability | 2.1 | 2.8 | 2.8 | 1.7 |
| Blood Red Count | 2 | 19.4 | 19.4 | 4.4 |
| Frequency of depressed mood in last 2 weeks | 2 | 2.9 | 2.9 | 1.7 |
| Townsend deprivation index at recruitment | 2 | 2.8 | 2.8 | 1.7 |
| Average weekly fortified wine intake | 2 | 2.4 | 2.4 | -1.5 |
| Health satisfaction | 2 | 2.6 | 2.6 | 1.6 |
| Cholesterol lowering medication | 2 | 3 | 3 | 1.7 |
| Current tobacco smoking | 1.9 | 3 | 3 | 1.7 |
| Illnesses of siblings | 1.9 | 2.6 | 2.6 | -1.6 |
| Medication for cholesterol | 1.9 | 2.9 | 2.9 | -1.7 |
| Medication: Aspirin | 1.9 | 2.5 | 2.5 | 1.6 |
| Commuting to job workplace: Public transport | 1.9 | 2.2 | 2.2 | -1.5 |
| Illnesses of father: High blood pressure | 1.9 | 2.3 | 2.3 | 1.5 |
| Major Depression (MDD) | 1.8 | 2.2 | 2.2 | 1.5 |
| Hair/balding pattern: Pattern 4 | 1.8 | 4.4 | 4.4 | -2.1 |
| Pulse wave reflection index | 1.8 | 2.2 | 2.2 | 1.5 |
| Mouth/teeth dental problems: Loose teeth | 1.8 | 2.1 | 2.1 | 1.5 |
| Gastro-oesophageal reflux (gord) / gastric reflux (Self-reported) | 1.8 | 2.1 | 2.1 | 1.4 |
| Neuroticism | 1.7 | 4 | 4 | 2 |
| Other serious medical condition/disability diagnosed by doctor | 1.7 | 2.2 | 2.2 | 1.5 |
| Diverticular disease/diverticulitis (self-reported) | 1.7 | 2 | 2 | 1.4 |
| Nervous feelings | 1.7 | 3.1 | 3.1 | 1.8 |
| Emphysema/chronic bronchitis | 1.7 | 2 | 2 | -1.4 |
| Medication: Aspirin | 1.7 | 2.3 | 2.3 | 1.5 |
| Age at First Birth | 1.6 | 2.4 | 2.4 | -1.5 |
| Hearing difficulty/problems with background noise | 1.6 | 2.5 | 2.5 | 1.6 |
| Worry too long after embarrassment | 1.6 | 2.7 | 2.7 | 1.6 |
| Eye problems/disorders: Glaucoma | 1.6 | 1.9 | 1.9 | -1.4 |
| Number of treatments/medications taken | 1.5 | 2.6 | 2.6 | 1.6 |
| Forced expiratory volume in 1-second (FEV1) | 1.5 | 5.1 | 5.1 | 2.3 |
| Medication: Cholesterol lowering | 1.5 | 2.3 | 2.3 | 1.5 |
| Hand grip strength (right) | 1.4 | 3.8 | 3.8 | -2 |
| Heel bone mineral density (BMD) T-score, automated (left) | 1.4 | 3.1 | 3.1 | 1.8 |
| Forced expiratory volume in 1-second (FEV1), Best measure | 1.4 | 4.6 | 4.6 | 2.1 |
| Medication: Seretide 50 evohaler | 1.4 | 1.7 | 1.7 | -1.3 |
| Supplements: Fish oil (including cod liver oil) | 1.4 | 1.8 | 1.8 | 1.3 |
| Lung FVC | 1.3 | 6.7 | 6.7 | 2.6 |
| Supplements: Vitamin C | 1.3 | 1.6 | 1.6 | 1.3 |
| Illnesses of father: None of the above (group 1) | 1.3 | 1.6 | 1.6 | -1.3 |
| Alzheimer’s Disease (including proxy) | 1.2 | 1.7 | 1.7 | -1.3 |
| Chronic bronchitis/emphysema (father) | 1.2 | 1.5 | 1.5 | -1.2 |
| Medication for cholesterol, blood pressure or diabetes | 1.2 | 2.1 | 2.1 | -1.4 |
| Medication: Blood pressure | 1.2 | 2.2 | 2.2 | 1.5 |
| Arm predicted mass (right) | 1.2 | 6.1 | 6.1 | 2.5 |
| Alzheimer’s Disease (in mother) | 1.1 | 1.3 | 1.3 | -1.1 |
| Ever used hormone-replacement therapy (HRT) | 1.1 | 1.4 | 1.4 | 1.2 |
| Impedance of leg (right) | 1.1 | 5.5 | 5.5 | -2.3 |
| Body mass index (BMI) | 1.1 | 4.7 | 4.7 | 2.2 |
| Waist circumference | 1.1 | 4 | 4 | 2 |
| Tense / ‘highly strung’ | 1.1 | 1.8 | 1.8 | 1.3 |
| Suffer from ‘nerves’ | 1.1 | 1.7 | 1.7 | 1.3 |
| Tinnitus: Yes, now most or all of the time | 1.1 | 1.3 | 1.3 | -1.1 |
| High blood pressure (mother) | 1 | 1.5 | 1.5 | -1.2 |
| Impedance of leg (left) | 1 | 5 | 5 | -2.2 |
| Medication: Amlodipine | 1 | 1.5 | 1.5 | 1.2 |
| Arm fat-free mass (right) | 1 | 5.1 | 5.1 | 2.3 |
| Pulse rate, automated reading | 1 | 3.3 | 3.3 | 1.8 |
| Number of full sisters | 1 | 1.2 | 1.2 | 1.1 |
| Mineral and other dietary supplements | 1 | 1.3 | 1.3 | -1.1 |
| Wears glasses or contact lenses | 1 | 1.1 | 1.1 | 1.1 |
| Ever had prostate specific antigen (PSA) test | 1 | 1.1 | 1.1 | 1.1 |
| Arm predicted mass (left) | 1 | 5 | 5 | 2.2 |
| Depressed Affect (Nagel 2018) | 0.9 | 1.9 | 1.9 | 1.4 |
| HbA1C | 0.9 | 1 | 1 | -1 |
| Worrier / anxious feelings | 0.9 | 1.7 | 1.7 | 1.3 |
| Friendships satisfaction | 0.9 | 1.1 | 1.1 | -1 |
| Arm fat-free mass (left) | 0.9 | 4.8 | 4.8 | 2.2 |
| Waist Hip Ratio (WHR) | 0.8 | 3.6 | 3.6 | 1.9 |
| Relative age of first facial hair | 0.8 | 1.6 | 1.6 | -1.3 |
| Serious illness, injury or assault to yourself | 0.8 | 1 | 1 | 1 |
| Pain all over the body in last month | 0.8 | 0.8 | 0.8 | -0.9 |
| Falls in the last year | 0.8 | 1.1 | 1.1 | 1.1 |
| Pulse wave Arterial Stiffness index | 0.8 | 0.9 | 0.9 | 1 |
| Trunk predicted mass | 0.8 | 4.6 | 4.6 | 2.1 |
| Gout (self-reported) | 0.8 | 1.1 | 1.1 | 1 |
| Pulse wave peak to peak time | 0.8 | 0.9 | 0.9 | -1 |
| Medication: Ibuprofen | 0.8 | 1 | 1 | -1 |
| Ever had stillbirth, spontaneous miscarriage or termination | 0.8 | 0.8 | 0.8 | 0.9 |
| Osteoporosis (self-reported) | 0.8 | 1 | 1 | 1 |
| Blood White Count | 0.7 | 5 | 5 | 2.2 |
| Chronotype (morning person) | 0.7 | 1.7 | 1.7 | -1.3 |
| Systolic blood pressure, automated reading | 0.7 | 2.1 | 2.1 | 1.4 |
| Fed-up feelings | 0.7 | 1.3 | 1.3 | 1.2 |
| Tinnitus: Yes, but not now, but have in the past | 0.7 | 0.8 | 0.8 | -0.9 |
| Trunk fat-free mass | 0.7 | 4.2 | 4.2 | 2.1 |
| Prospective memory result | 0.7 | 0.9 | 0.9 | -0.9 |
| Glaucoma (self-reported) | 0.7 | 0.8 | 0.8 | -0.9 |
| Whole body fat-free mass | 0.7 | 4 | 4 | 2 |
| Whole body water mass | 0.7 | 4 | 4 | 2 |
| Basal metabolic rate | 0.7 | 3.8 | 3.8 | 1.9 |
| Number of operations (self-reported) | 0.7 | 1 | 1 | -1 |
| Diastolic blood pressure, automated reading | 0.7 | 2.3 | 2.3 | 1.5 |
| Alcohol drinker status: Previous | 0.7 | 0.8 | 0.8 | 0.9 |
| Schizophrenia (2014) | 0.6 | 1.3 | 1.3 | 1.2 |
| Hearing difficulty/problems: Yes | 0.6 | 1 | 1 | 1 |
| Medication for hormone replacement therapy | 0.6 | 0.7 | 0.7 | -0.8 |
| Multivitamins +/- minerals | 0.6 | 0.8 | 0.8 | 0.9 |
| Heel bone mineral density (BMD) T-score, automated (right) | 0.6 | 1.5 | 1.5 | 1.2 |
| Financial difficulties in last 2 years | 0.6 | 0.9 | 0.9 | 0.9 |
| Father’s age at death | 0.6 | 0.8 | 0.8 | -0.9 |
| Forced vital capacity (FVC), Best measure | 0.6 | 2 | 2 | 1.4 |
| Comparative body size at age 10 | 0.6 | 1.5 | 1.5 | 1.2 |
| Leg fat mass (right) | 0.6 | 2.4 | 2.4 | 1.5 |
| Chest pain or discomfort | 0.6 | 0.8 | 0.8 | 0.9 |
| Number of self-reported non-cancer illnesses | 0.6 | 1 | 1 | 1 |
| Supplements: Calcium | 0.6 | 0.6 | 0.6 | 0.8 |
| Leg fat mass (left) | 0.6 | 2.4 | 2.4 | 1.6 |
| Ulcerative Colitis | 0.5 | 0.7 | 0.7 | 0.9 |
| Systolic Blood Pressure | 0.5 | 2.7 | 2.7 | 1.6 |
| Type 2 Diabetes (T2D) (2018) | 0.5 | 0.9 | 0.9 | 1 |
| Forced vital capacity (FVC) | 0.5 | 1.9 | 1.9 | 1.4 |
| Medication: Simvastatin | 0.5 | 0.7 | 0.7 | 0.8 |
| Hair/balding pattern: Pattern 3 | 0.5 | 0.7 | 0.7 | -0.9 |
| Medication: Lansoprazole | 0.5 | 0.6 | 0.6 | -0.8 |
| Breastfed as a baby | 0.5 | 0.6 | 0.6 | -0.8 |
| Leg fat-free mass (right) | 0.5 | 2.6 | 2.6 | 1.6 |
| Forced expiratory volume in 1-second (FEV1), predicted percentage | 0.5 | 1.1 | 1.1 | 1 |
| Leg predicted mass (right) | 0.5 | 2.5 | 2.5 | 1.6 |
| Pain type(s) experienced in last month: Hip pain | 0.5 | 0.7 | 0.7 | 0.8 |
| Schizophrenia (2018) | 0.4 | 0.8 | 0.8 | 0.9 |
| Ulcerative Colitis (UC) | 0.4 | 0.7 | 0.7 | -0.8 |
| Reaction Time | 0.4 | 0.7 | 0.7 | 0.8 |
| Triglycerides | 0.4 | 0.7 | 0.7 | 0.8 |
| Leg fat-free mass (left) | 0.4 | 2.2 | 2.2 | 1.5 |
| Supplements: Glucosamine | 0.4 | 0.5 | 0.5 | 0.7 |
| Leg predicted mass (left) | 0.4 | 1.9 | 1.9 | 1.4 |
| Alcohol usually taken with meals | 0.4 | 0.7 | 0.7 | -0.8 |
| Weight | 0.4 | 2 | 2 | 1.4 |
| Time employed in main current job | 0.4 | 0.5 | 0.5 | -0.7 |
| Arm fat mass (right) | 0.4 | 1.7 | 1.7 | 1.3 |
| Seen doctor (GP) for nerves, anxiety, tension or depression | 0.4 | 0.6 | 0.6 | 0.8 |
| Leg fat percentage (right) | 0.4 | 1.4 | 1.4 | 1.2 |
| Qualifications: NVQ or HND or HNC or equivalent | 0.4 | 0.5 | 0.5 | 0.7 |
| Commuting to job workplace: Car/motor vehicle | 0.4 | 0.5 | 0.5 | -0.7 |
| Commuting to job workplace: Walk | 0.4 | 0.4 | 0.4 | 0.6 |
| Financial situation satisfaction | 0.4 | 0.5 | 0.5 | 0.7 |
| Leg fat percentage (left) | 0.4 | 1.4 | 1.4 | 1.2 |
| Risk taking | 0.4 | 0.5 | 0.5 | 0.7 |
| Commuting to job workplace: Cycle | 0.4 | 0.5 | 0.5 | -0.7 |
| Pack years of smoking | 0.4 | 0.6 | 0.6 | -0.8 |
| Crohns Disease (2012) | 0.3 | 0.4 | 0.4 | 0.7 |
| HDL Cholesterol | 0.3 | 0.5 | 0.5 | -0.7 |
| Hair Pigment | 0.3 | 9.8 | 9.8 | 3.1 |
| Angina | 0.3 | 0.4 | 0.4 | 0.6 |
| Supplements: Zinc | 0.3 | 0.3 | 0.3 | -0.6 |
| Medication: Co-codamol | 0.3 | 0.3 | 0.3 | 0.6 |
| Diabetes (father) | 0.3 | 0.4 | 0.4 | 0.6 |
| Pack years adult smoking proportion | 0.3 | 0.4 | 0.4 | -0.7 |
| Bilateral oophorectomy (both ovaries removed) | 0.3 | 0.3 | 0.3 | 0.6 |
| Number of incorrect matches in round | 0.3 | 0.6 | 0.6 | 0.8 |
| Noisy workplace | 0.3 | 0.3 | 0.3 | -0.6 |
| Mouth/teeth dental problems: Dentures | 0.3 | 0.6 | 0.6 | 0.8 |
| Qualifications: O levels/GCSEs or equivalent | 0.3 | 0.5 | 0.5 | -0.7 |
| Number of children fathered | 0.3 | 0.3 | 0.3 | 0.6 |
| Illness, injury, bereavement, stress in last 2 years | 0.3 | 0.3 | 0.3 | -0.6 |
| Handedness (chirality/laterality): Left-handed | 0.3 | 0.3 | 0.3 | 0.6 |
| Medication: Gliclazide | 0.3 | 0.4 | 0.4 | 0.6 |
| Alcohol drinker status: Never | 0.3 | 0.3 | 0.3 | -0.6 |
| Bipolar Disorder or Schizophrenia | 0.2 | 0.4 | 0.4 | 0.6 |
| Crohns Disease (2017) | 0.2 | 0.5 | 0.5 | 0.7 |
| Body Mass Index (BMI) (2010) | 0.2 | 0.3 | 0.3 | 0.6 |
| Tanning | 0.2 | 1.9 | 1.9 | -1.4 |
| Hand grip strength (left) | 0.2 | 0.4 | 0.4 | -0.6 |
| Ever had hysterectomy (womb removed) | 0.2 | 0.2 | 0.2 | 0.4 |
| Severe depression (siblings) | 0.2 | 0.2 | 0.2 | 0.5 |
| Maternal smoking around birth | 0.2 | 0.4 | 0.4 | -0.6 |
| Relative age voice broke | 0.2 | 0.4 | 0.4 | -0.6 |
| Hair/balding pattern: Pattern 2 | 0.2 | 0.2 | 0.2 | 0.5 |
| Happiness | 0.2 | 0.2 | 0.2 | -0.5 |
| Standing height | 0.2 | 1.7 | 1.7 | -1.3 |
| Exposure to tobacco smoke at home | 0.2 | 0.2 | 0.2 | -0.4 |
| Peak expiratory flow (PEF) | 0.2 | 0.4 | 0.4 | -0.7 |
| Loud music exposure frequency | 0.2 | 0.2 | 0.2 | -0.5 |
| Eye problems/disorders: None of the above | 0.2 | 0.2 | 0.2 | 0.5 |
| Leg pain on walking | 0.2 | 0.2 | 0.2 | 0.5 |
| Forced expiratory volume in 1-second (FEV1), predicted | 0.2 | 0.4 | 0.4 | 0.7 |
| Average weekly red wine intake | 0.2 | 0.2 | 0.2 | -0.5 |
| Long-standing illness, disability or infirmity | 0.2 | 0.3 | 0.3 | 0.5 |
| Arm fat mass (left) | 0.2 | 1 | 1 | 1 |
| Mother’s age at death | 0.2 | 0.2 | 0.2 | 0.5 |
| Medication: Omeprazole (e.g. Zanprol) | 0.2 | 0.3 | 0.3 | -0.5 |
| Deep venous thrombosis (DVT) (self-reported) | 0.2 | 0.3 | 0.3 | -0.5 |
| Alzheimer’s Disease (in father) | 0.1 | 0.1 | 0.1 | -0.4 |
| Ovarian Cancer | 0.1 | 0.2 | 0.2 | -0.4 |
| Coronary Artery Disease (CAD) | 0.1 | 0.1 | 0.1 | -0.3 |
| Fasting Glucose | 0.1 | 0.1 | 0.1 | -0.3 |
| Lupus | 0.1 | 0.1 | 0.1 | -0.4 |
| Neuroticism (2016) | 0.1 | 0.2 | 0.2 | 0.4 |
| Average weekly spirits intake | 0.1 | 0.1 | 0.1 | -0.3 |
| Diabetes (mother) | 0.1 | 0.1 | 0.1 | 0.3 |
| Trunk fat percentage | 0.1 | 0.3 | 0.3 | -0.5 |
| Had menopause | 0.1 | 0.2 | 0.2 | 0.4 |
| Arm fat percentage (right) | 0.1 | 0.3 | 0.3 | 0.5 |
| Alcohol intake versus 10 years previously | 0.1 | 0.1 | 0.1 | 0.3 |
| Serious illness, injury or assault of a close relative in last 2 years | 0.1 | 0.1 | 0.1 | 0.3 |
| Ever highly irritable/argumentative for 2 days | 0.1 | 0.2 | 0.2 | 0.4 |
| Blood clot in the leg (DVT) | 0.1 | 0.1 | 0.1 | -0.3 |
| Number of full brothers | 0.1 | 0.2 | 0.2 | 0.4 |
| Wheeze or whistling in the chest in last year | 0.1 | 0.2 | 0.2 | -0.4 |
| Angina (self-reported) | 0.1 | 0.2 | 0.2 | 0.4 |
| Medication: Allopurinol | 0.1 | 0.1 | 0.1 | 0.4 |
| Medication: Omeprazole | 0.1 | 0.1 | 0.1 | 0.3 |
| Heart disease (mother) | 0.1 | 0.1 | 0.1 | 0.3 |
| Whole body fat mass | 0.1 | 0.5 | 0.5 | 0.7 |
| Family relationship satisfaction | 0.1 | 0.1 | 0.1 | 0.4 |
| Illnesses of father: Heart disease | 0.1 | 0.1 | 0.1 | 0.3 |
| Mood swings | 0.1 | 0.2 | 0.2 | 0.4 |
| Diabetes diagnosed by doctor | 0.1 | 0.1 | 0.1 | 0.4 |
| Eye problems/disorders: Cataract | 0.1 | 0.1 | 0.1 | 0.2 |
| Breast cancer (self-reported) | 0.1 | 0.1 | 0.1 | -0.4 |
| Osteoarthritis (self-reported) | 0.1 | 0.1 | 0.1 | 0.3 |
| Medication: Bendroflumethiazide | 0.1 | 0.1 | 0.1 | 0.4 |
| Medication: Lisinopril | 0.1 | 0.2 | 0.2 | 0.4 |
| Average weekly beer plus cider intake | 0.1 | 0.2 | 0.2 | 0.4 |
| Fractured/broken bones in last 5 years | 0.1 | 0.1 | 0.1 | 0.4 |
| Basal cell carcinoma (self-reported) | 0.1 | 0.1 | 0.1 | 0.3 |
| Bipolar Disorder (2018) | 0 | 0 | 0 | -0.1 |
| Irritable Bowel Disease (IBD) | 0 | 0 | 0 | 0.1 |
| Breast Cancer | 0 | 0 | 0 | 0 |
| Prostate Cancer | 0 | 0 | 0 | 0 |
| LDL Cholesterol | 0 | 0 | 0 | -0.1 |
| Blood Platelet Count | 0 | 0.5 | 0.5 | 0.7 |
| Heel T-Score | 0 | 0.3 | 0.3 | 0.5 |
| Height | 0 | 0.6 | 0.6 | -0.8 |
| Medication: Metformin | 0 | 0 | 0 | 0.1 |
| Taking other prescription medications | 0 | 0 | 0 | -0.2 |
| Medication: Atenolol | 0 | 0 | 0 | 0.1 |
| Sitting height | 0 | 0.2 | 0.2 | 0.4 |
| Trunk fat mass | 0 | 0.1 | 0.1 | 0.2 |
| Ever unenthusiastic/disinterested for a whole week | 0 | 0 | 0 | -0.1 |
| Heart attack | 0 | 0.1 | 0.1 | -0.2 |
| Diabetes (self-reported) | 0 | 0 | 0 | 0.2 |
| Hip circumference | 0 | 0.1 | 0.1 | 0.3 |
| Pulse rate | 0 | 0 | 0 | 0 |
| Medication: Ibuprofen (e.g. Nurofen) | 0 | 0.1 | 0.1 | -0.2 |
| Lung cancer (father) | 0 | 0 | 0 | 0.1 |
| Headache pain in last month | 0 | 0.1 | 0.1 | 0.3 |
| Body fat percentage | 0 | 0 | 0 | 0.1 |
| Seen a psychiatrist for nerves, anxiety, tension or depression | 0 | 0 | 0 | 0.2 |
| Shortness of breath walking on level ground | 0 | 0 | 0 | 0.1 |
| Migraine (self-reported) | 0 | 0 | 0 | -0.2 |
| Mean time to correctly identify matches | 0 | 0 | 0 | 0.2 |
| Comparative height size at age 10 | 0 | 0.1 | 0.1 | -0.4 |
| Had major operations | 0 | 0 | 0 | 0.1 |
| Arm fat percentage (left) | 0 | 0.1 | 0.1 | 0.4 |
| Miserableness | 0 | 0 | 0 | 0 |
| Hearing aid user | 0 | 0 | 0 | 0.2 |
| Irritability | 0 | 0 | 0 | -0.1 |
| Ever depressed for a whole week | 0 | 0.1 | 0.1 | -0.2 |
| Heart attack/myocardial infarction (self-reported) | 0 | 0 | 0 | -0.1 |

**Supplementary Table S13. Traits associated with predicted *GDF11* expression in the skin in the TWAS Hub.**

| **Trait** | **Avg chi^2^ ratio** | **Avg chi^2^** | **Max chi^2^** | **Z-score** |
| --- | --- | --- | --- | --- |
| Age at First Birth | 1.7 | 2.5 | 2.5 | 1.6 |
| Age at first live birth | 0 | 0 | 0 | -0.2 |
| Age at last live birth | 0.2 | 0.3 | 0.3 | -0.6 |
| Age started oral contraceptive pill | 0.7 | 0.9 | 0.9 | 0.9 |
| Age when periods started (menarche) | 1.4 | 3.4 | 3.4 | -1.8 |
| Alcohol drinker status: Never | 0.3 | 0.4 | 0.4 | 0.6 |
| Alcohol drinker status: Previous | 5.5 | 6.2 | 6.2 | 2.5 |
| Alcohol intake frequency. | 0.7 | 1.6 | 1.6 | 1.3 |
| Alcohol intake versus 10 years previously | 1.3 | 2 | 2 | 1.4 |
| Alcohol usually taken with meals | 0 | 0 | 0 | 0.2 |
| Allergy | 1.5 | 3.5 | 3.5 | 1.9 |
| Allergy or Eczema | 0.8 | 2.1 | 2.1 | -1.4 |
| Alzheimer’s Disease (in father) | 1.7 | 1.9 | 1.9 | 1.4 |
| Alzheimer’s Disease (in mother) | 0.2 | 0.2 | 0.2 | -0.4 |
| Alzheimer’s Disease (including proxy) | 0 | 0 | 0 | 0.2 |
| Angina | 1.2 | 1.6 | 1.6 | 1.3 |
| Angina (self-reported) | 0.7 | 0.9 | 0.9 | 0.9 |
| Arm fat mass (left) | 0.2 | 0.9 | 0.9 | -0.9 |
| Arm fat mass (right) | 0.3 | 1.1 | 1.1 | -1.1 |
| Arm fat percentage (left) | 0.1 | 0.4 | 0.4 | -0.7 |
| Arm fat percentage (right) | 0.3 | 1 | 1 | -1 |
| Arm fat-free mass (left) | 0.1 | 0.4 | 0.4 | -0.7 |
| Arm fat-free mass (right) | 0 | 0 | 0 | -0.1 |
| Arm predicted mass (left) | 0.1 | 0.4 | 0.4 | -0.6 |
| Arm predicted mass (right) | 0 | 0 | 0 | -0.2 |
| Asthma | 0.1 | 0.1 | 0.1 | -0.3 |
| Asthma (self-reported) | 0.1 | 0.2 | 0.2 | -0.4 |
| Average weekly beer plus cider intake | 0 | 0 | 0 | 0.1 |
| Average weekly champagne plus white wine intake | 2 | 2.8 | 2.8 | 1.7 |
| Average weekly fortified wine intake | 7.2 | 8.4 | 8.4 | 2.9 |
| Average weekly red wine intake | 0.1 | 0.2 | 0.2 | 0.5 |
| Average weekly spirits intake | 0.9 | 1.2 | 1.2 | -1.1 |
| Back pain experienced in last month | 0.4 | 0.6 | 0.6 | -0.8 |
| Basal cell carcinoma (self-reported) | 2.8 | 3.3 | 3.3 | -1.8 |
| Basal metabolic rate | 0.2 | 1.1 | 1.1 | -1.1 |
| Bilateral oophorectomy (both ovaries removed) | 2.5 | 2.9 | 2.9 | 1.7 |
| Bipolar Disorder (2011) | 2.1 | 2 | 2 | -1.4 |
| Bipolar Disorder (2018) | 0 | 0 | 0 | -0.1 |
| Bipolar Disorder or Schizophrenia | 0 | 0 | 0 | 0 |
| Birth weight | 0.9 | 2 | 2 | 1.4 |
| Birth weight of first child | 0 | 0 | 0 | -0.1 |
| Blood clot in the leg (DVT) | 0.2 | 0.2 | 0.2 | 0.5 |
| Blood Eosinophil Count | 0.3 | 2.5 | 2.5 | 1.6 |
| Blood Platelet Count | 0.1 | 0.6 | 0.6 | -0.8 |
| Blood Red Count | 0.5 | 5 | 5 | -2.2 |
| Blood White Count | 0.1 | 1 | 1 | 1 |
| BMI | 0 | 0 | 0 | -0.2 |
| Body fat percentage | 0.3 | 1 | 1 | -1 |
| Body mass index (BMI) | 0.1 | 0.2 | 0.2 | 0.5 |
| Body Mass Index (BMI) (2010) | 0.5 | 0.7 | 0.7 | -0.8 |
| Breast Cancer | 0.3 | 0.7 | 0.7 | -0.8 |
| Breast cancer (self-reported) | 0.3 | 0.4 | 0.4 | -0.6 |
| Breastfed as a baby | 1.7 | 2 | 2 | -1.4 |
| Cardiovascular Disease | 1.7 | 5.5 | 5.5 | -2.3 |
| Chest pain or discomfort | 0.6 | 0.9 | 0.9 | -0.9 |
| Cholesterol lowering medication | 0.1 | 0.1 | 0.1 | 0.3 |
| Chronic bronchitis/emphysema (father) | 0.3 | 0.3 | 0.3 | 0.5 |
| Chronic bronchitis/emphysema (mother) | 2.4 | 2.8 | 2.8 | 1.7 |
| Chronotype (morning person) | 0.2 | 0.4 | 0.4 | -0.6 |
| Commuting to job workplace: Car/motor vehicle | 0.9 | 1.1 | 1.1 | 1 |
| Commuting to job workplace: Cycle | 4.6 | 5.6 | 5.6 | -2.4 |
| Commuting to job workplace: Public transport | 0.2 | 0.3 | 0.3 | 0.5 |
| Commuting to job workplace: Walk | 2.5 | 2.7 | 2.7 | -1.6 |
| Comparative body size at age 10 | 0.1 | 0.2 | 0.2 | 0.4 |
| Comparative height size at age 10 | 0.7 | 4 | 4 | -2 |
| Coronary Artery Disease (CAD) | 0.1 | 0.1 | 0.1 | 0.3 |
| Crohns Disease (2012) | 1.3 | 2 | 2 | -1.4 |
| Crohns Disease (2017) | 0 | 0 | 0 | 0 |
| Current tobacco smoking | 0.3 | 0.4 | 0.4 | 0.7 |
| Deep venous thrombosis (DVT) (self-reported) | 0 | 0 | 0 | 0.2 |
| Depressed Affect (Nagel 2018) | 0.9 | 1.9 | 1.9 | 1.4 |
| Depression (Nagel 2018) | 0.5 | 0.7 | 0.7 | 0.9 |
| Depression (self-reported) | 1.7 | 2 | 2 | 1.4 |
| Diabetes (father) | 0.3 | 0.3 | 0.3 | 0.6 |
| Diabetes (mother) | 0.3 | 0.4 | 0.4 | -0.6 |
| Diabetes (self-reported) | 0.1 | 0.2 | 0.2 | 0.5 |
| Diabetes diagnosed by doctor | 0 | 0.1 | 0.1 | 0.3 |
| Diastolic blood pressure, automated reading | 15.4 | 47.7 | 47.7 | -6.9 |
| Diverticular disease/diverticulitis (self-reported) | 0 | 0 | 0 | -0.2 |
| Emphysema/chronic bronchitis | 1.1 | 1.3 | 1.3 | -1.1 |
| Ever depressed for a whole week | 0.1 | 0.1 | 0.1 | 0.3 |
| Ever had bowel cancer screening | 0 | 0 | 0 | 0.1 |
| Ever had hysterectomy (womb removed) | 3 | 3.4 | 3.4 | 1.8 |
| Ever had prostate specific antigen (PSA) test | 0.8 | 0.9 | 0.9 | -1 |
| Ever had stillbirth, spontaneous miscarriage or termination | 1.5 | 1.6 | 1.6 | 1.3 |
| Ever highly irritable/argumentative for 2 days | 0.4 | 0.4 | 0.4 | 0.7 |
| Ever smoked | 1.6 | 2.8 | 2.8 | 1.7 |
| Ever taken oral contraceptive pill | 0.6 | 0.7 | 0.7 | 0.8 |
| Ever unenthusiastic/disinterested for a whole week | 0 | 0 | 0 | 0 |
| Ever used hormone-replacement therapy (HRT) | 0.1 | 0.2 | 0.2 | -0.4 |
| Exposure to tobacco smoke at home | 1 | 1.2 | 1.2 | 1.1 |
| Exposure to tobacco smoke outside home | 1.8 | 2.4 | 2.4 | 1.5 |
| Eye problems/disorders: Cataract | 0.1 | 0.1 | 0.1 | 0.4 |
| Eye problems/disorders: Diabetes related eye disease | 0.2 | 0.2 | 0.2 | 0.4 |
| Eye problems/disorders: Glaucoma | 2.3 | 2.7 | 2.7 | -1.6 |
| Eye problems/disorders: None of the above | 0 | 0 | 0 | -0.1 |
| Falls in the last year | 1.6 | 2.2 | 2.2 | 1.5 |
| Family relationship satisfaction | 0.7 | 0.9 | 0.9 | 1 |
| Fasting Glucose | 1.3 | 1.5 | 1.5 | 1.2 |
| Father’s age at death | 0.1 | 0.1 | 0.1 | 0.3 |
| Fed-up feelings | 1 | 1.7 | 1.7 | 1.3 |
| Financial difficulties in last 2 years | 1.3 | 1.7 | 1.7 | -1.3 |
| Financial situation satisfaction | 0.7 | 0.8 | 0.8 | 0.9 |
| Fluid intelligence score | 0.9 | 1.8 | 1.8 | -1.4 |
| Forced expiratory volume in 1-second (FEV1) | 4.2 | 14.2 | 14.2 | -3.8 |
| Forced expiratory volume in 1-second (FEV1), Best measure | 4.1 | 13 | 13 | -3.6 |
| Forced expiratory volume in 1-second (FEV1), predicted | 2.6 | 5.3 | 5.3 | -2.3 |
| Forced expiratory volume in 1-second (FEV1), predicted percentage | 0.6 | 1.2 | 1.2 | -1.1 |
| Forced vital capacity (FVC) | 2.8 | 10.8 | 10.8 | -3.3 |
| Forced vital capacity (FVC), Best measure | 2.5 | 9.3 | 9.3 | -3 |
| Fractured/broken bones in last 5 years | 0.1 | 0.1 | 0.1 | -0.3 |
| Frequency of depressed mood in last 2 weeks | 1.8 | 2.5 | 2.5 | 1.6 |
| Frequency of tenseness / restlessness in last 2 weeks | 0.1 | 0.1 | 0.1 | 0.3 |
| Frequency of tiredness / lethargy in last 2 weeks | 1.1 | 1.8 | 1.8 | -1.3 |
| Frequency of unenthusiasm / disinterest in last 2 weeks | 0.1 | 0.1 | 0.1 | 0.3 |
| Friendships satisfaction | 0.1 | 0.2 | 0.2 | -0.4 |
| Gastro-oesophageal reflux (gord) / gastric reflux (Self-reported) | 0.1 | 0.1 | 0.1 | 0.2 |
| Glaucoma (self-reported) | 0.6 | 0.7 | 0.7 | -0.8 |
| Gout (self-reported) | 0.2 | 0.2 | 0.2 | 0.5 |
| Guilty feelings | 0.1 | 0.2 | 0.2 | 0.5 |
| Had major operations | 0.2 | 0.2 | 0.2 | -0.4 |
| Had menopause | 0.2 | 0.2 | 0.2 | -0.4 |
| Had other major operations | 1 | 1.3 | 1.3 | -1.1 |
| Hair Pigment | 0 | 0 | 0 | -0.2 |
| Hair/balding pattern: Pattern 2 | 2.5 | 3.2 | 3.2 | 1.8 |
| Hair/balding pattern: Pattern 3 | 0 | 0 | 0 | -0.1 |
| Hair/balding pattern: Pattern 4 | 1.7 | 4 | 4 | -2 |
| Hand grip strength (left) | 0.7 | 1.8 | 1.8 | -1.3 |
| Hand grip strength (right) | 0 | 0 | 0 | -0.2 |
| Handedness (chirality/laterality): Left-handed | 2.9 | 3.5 | 3.5 | -1.9 |
| Happiness | 1.5 | 1.8 | 1.8 | 1.3 |
| Hayfever, allergic rhinitis or eczema | 0.9 | 1.9 | 1.9 | -1.4 |
| Hayfever/allergic rhinitis (self-reported) | 0.8 | 1 | 1 | -1 |
| HbA1C | 0 | 0 | 0 | -0.2 |
| HDL Cholesterol | 0.7 | 1.1 | 1.1 | -1.1 |
| Headache pain in last month | 0.3 | 0.5 | 0.5 | -0.7 |
| Health satisfaction | 0.5 | 0.6 | 0.6 | 0.8 |
| Hearing aid user | 0.7 | 0.8 | 0.8 | -0.9 |
| Hearing difficulty/problems with background noise | 0 | 0 | 0 | 0 |
| Hearing difficulty/problems: Yes | 4.2 | 6.4 | 6.4 | 2.5 |
| Heart attack | 0 | 0 | 0 | -0.2 |
| Heart attack/myocardial infarction (self-reported) | 0 | 0.1 | 0.1 | -0.2 |
| Heart disease (mother) | 1.5 | 1.8 | 1.8 | -1.3 |
| Heart disease (siblings) | 0.5 | 0.6 | 0.6 | 0.7 |
| Heel bone mineral density (BMD) T-score, automated (left) | 1.4 | 3.1 | 3.1 | 1.8 |
| Heel bone mineral density (BMD) T-score, automated (right) | 1.4 | 3.2 | 3.2 | 1.8 |
| Heel T-Score | 0 | 0.2 | 0.2 | 0.4 |
| Height | 1.1 | 21.6 | 21.6 | -4.7 |
| High blood pressure | 0.2 | 0.7 | 0.7 | -0.8 |
| High blood pressure (mother) | 2.6 | 3.6 | 3.6 | -1.9 |
| High blood pressure (siblings) | 0.7 | 1 | 1 | -1 |
| High cholesterol (Self-reported) | 1.1 | 1.9 | 1.9 | -1.4 |
| Hip circumference | 0.1 | 0.4 | 0.4 | -0.6 |
| Hypertension (Self-reported) | 0.3 | 0.8 | 0.8 | -0.9 |
| Hypothyroidism (self reported) | 2.2 | 5.1 | 5.1 | 2.3 |
| Hypothyroidism/myxoedema (self-reported) | 3.3 | 6.5 | 6.5 | 2.5 |
| Illness, injury, bereavement, stress in last 2 years | 0.5 | 0.6 | 0.6 | 0.8 |
| Illnesses of father: Heart disease | 0.3 | 0.5 | 0.5 | -0.7 |
| Illnesses of father: High blood pressure | 7.1 | 8.8 | 8.8 | -3 |
| Illnesses of father: None of the above (group 1) | 1.8 | 2.2 | 2.2 | 1.5 |
| Illnesses of mother | 4.6 | 5.7 | 5.7 | 2.4 |
| Illnesses of siblings | 0.5 | 0.6 | 0.6 | 0.8 |
| Illnesses of siblings: Diabetes | 0.8 | 1 | 1 | -1 |
| Impedance of arm (left) | 1.5 | 6.8 | 6.8 | -2.6 |
| Impedance of arm (right) | 2.7 | 12.6 | 12.6 | -3.5 |
| Impedance of leg (left) | 0.4 | 1.9 | 1.9 | -1.4 |
| Impedance of leg (right) | 0.3 | 1.6 | 1.6 | -1.2 |
| Impedance of whole body | 0.9 | 4.6 | 4.6 | -2.1 |
| Intelligence (Savage-Jansen 2018) | 0.3 | 0.9 | 0.9 | -1 |
| Irritability | 0.8 | 1.4 | 1.4 | 1.2 |
| Irritable Bowel Disease (IBD) | 0.4 | 0.9 | 0.9 | 0.9 |
| Knee pain experienced in last month | 0.2 | 0.3 | 0.3 | 0.5 |
| LDL Cholesterol | 0.2 | 0.3 | 0.3 | -0.6 |
| Leg fat mass (left) | 0 | 0.1 | 0.1 | -0.3 |
| Leg fat mass (right) | 0.1 | 0.2 | 0.2 | -0.5 |
| Leg fat percentage (left) | 0.1 | 0.2 | 0.2 | 0.5 |
| Leg fat percentage (right) | 0 | 0.1 | 0.1 | 0.3 |
| Leg fat-free mass (left) | 0.2 | 1 | 1 | -1 |
| Leg fat-free mass (right) | 0.2 | 1 | 1 | -1 |
| Leg pain on walking | 1.8 | 2.3 | 2.3 | 1.5 |
| Leg predicted mass (left) | 0.2 | 1.1 | 1.1 | -1 |
| Leg predicted mass (right) | 0.2 | 1.1 | 1.1 | -1.1 |
| Length of working week for main job | 0.6 | 0.6 | 0.6 | -0.8 |
| Loneliness, isolation | 0.4 | 0.6 | 0.6 | 0.7 |
| Long-standing illness, disability or infirmity | 0.5 | 0.8 | 0.8 | 0.9 |
| Loud music exposure frequency | 0.1 | 0.1 | 0.1 | -0.4 |
| Lung cancer (father) | 0.4 | 0.5 | 0.5 | 0.7 |
| Lung FEV1/FVC ratio | 1 | 5 | 5 | -2.2 |
| Lung FVC | 2.7 | 13.6 | 13.6 | -3.7 |
| Lupus | 1.9 | 2.9 | 2.9 | 1.7 |
| Major Depression (MDD) | 0 | 0 | 0 | 0.1 |
| Maternal smoking around birth | 0 | 0 | 0 | 0.1 |
| Mean Putamen Volume | 0 | 0 | 0 | 0.1 |
| Mean time to correctly identify matches | 0.1 | 0.2 | 0.2 | 0.4 |
| Medication for cholesterol | 0.1 | 0.1 | 0.1 | -0.4 |
| Medication for cholesterol, blood pressure or diabetes | 0.3 | 0.4 | 0.4 | 0.7 |
| Medication for hormone replacement therapy | 0.4 | 0.5 | 0.5 | 0.7 |
| Medication for pain relief, constipation, heartburn | 0.2 | 0.4 | 0.4 | 0.6 |
| Medication: Allopurinol | 1.1 | 1.5 | 1.5 | -1.2 |
| Medication: Amlodipine | 0.6 | 0.8 | 0.8 | -0.9 |
| Medication: Aspirin | 0.9 | 1.1 | 1.1 | -1.1 |
| Medication: Aspirin | 0.5 | 0.7 | 0.7 | -0.8 |
| Medication: Atenolol | 0 | 0 | 0 | 0.1 |
| Medication: Atorvastatin | 5.9 | 7.3 | 7.3 | -2.7 |
| Medication: Bendroflumethiazide | 1.3 | 2 | 2 | -1.4 |
| Medication: Blood pressure | 0.3 | 0.5 | 0.5 | -0.7 |
| Medication: Blood pressure | 0.1 | 0.2 | 0.2 | -0.4 |
| Medication: Cholesterol lowering | 0.1 | 0.2 | 0.2 | -0.4 |
| Medication: Co-codamol | 0.1 | 0.1 | 0.1 | -0.3 |
| Medication: Gliclazide | 1.4 | 1.6 | 1.6 | 1.3 |
| Medication: Ibuprofen | 0.6 | 0.7 | 0.7 | -0.8 |
| Medication: Ibuprofen (e.g. Nurofen) | 0.6 | 0.8 | 0.8 | -0.9 |
| Medication: Lansoprazole | 0.2 | 0.2 | 0.2 | 0.4 |
| Medication: Laxatives (e.g. Dulcolax, Senokot) | 0.1 | 0.1 | 0.1 | 0.3 |
| Medication: Levothyroxine sodium | 1.4 | 2.4 | 2.4 | 1.5 |
| Medication: Lisinopril | 0 | 0.1 | 0.1 | -0.2 |
| Medication: Metformin | 0.3 | 0.4 | 0.4 | 0.6 |
| Medication: Omeprazole | 0.5 | 0.6 | 0.6 | 0.8 |
| Medication: Omeprazole (e.g. Zanprol) | 1.9 | 2.3 | 2.3 | 1.5 |
| Medication: Paracetamol | 2.4 | 3.8 | 3.8 | -1.9 |
| Medication: Paracetamol | 2.2 | 3.7 | 3.7 | -1.9 |
| Medication: Ramipril | 0.1 | 0.2 | 0.2 | 0.4 |
| Medication: Seretide 50 evohaler | 0.1 | 0.1 | 0.1 | 0.3 |
| Medication: Simvastatin | 0.5 | 0.7 | 0.7 | 0.8 |
| Medication: Ventolin 100micrograms inhaler | 0.1 | 0.1 | 0.1 | -0.3 |
| Migraine (self-reported) | 0.3 | 0.4 | 0.4 | -0.6 |
| Mineral and other dietary supplements | 2.3 | 3.1 | 3.1 | 1.8 |
| Miserableness | 1.1 | 1.8 | 1.8 | 1.4 |
| Mood swings | 0 | 0 | 0 | 0 |
| Mother’s age at death | 0.9 | 1.1 | 1.1 | 1.1 |
| Mouth/teeth dental problems | 1.1 | 1.6 | 1.6 | 1.3 |
| Mouth/teeth dental problems: Bleeding gums | 0.2 | 0.3 | 0.3 | -0.6 |
| Mouth/teeth dental problems: Dentures | 0 | 0 | 0 | 0.1 |
| Mouth/teeth dental problems: Loose teeth | 0.5 | 0.6 | 0.6 | 0.8 |
| Mouth/teeth dental problems: Mouth ulcers | 1.6 | 2.5 | 2.5 | -1.6 |
| Multivitamins +/- minerals | 0 | 0 | 0 | -0.1 |
| Myopia | 0 | 0 | 0 | 0 |
| Neck or shoulder pain in last month | 1 | 1.4 | 1.4 | -1.2 |
| Nervous feelings | 0.5 | 1 | 1 | -1 |
| Neuroticism | 0.1 | 0.2 | 0.2 | -0.4 |
| Neuroticism (2016) | 0.7 | 1.1 | 1.1 | -1 |
| Neuroticism (Nagel 2018) | 0.3 | 0.7 | 0.7 | 0.9 |
| Neuroticism score | 0 | 0 | 0 | -0.1 |
| Noisy workplace | 4 | 4.9 | 4.9 | 2.2 |
| Number of children fathered | 0.6 | 0.7 | 0.7 | 0.8 |
| Number of full brothers | 1.3 | 1.6 | 1.6 | 1.3 |
| Number of full sisters | 0.6 | 0.8 | 0.8 | -0.9 |
| Number of incorrect matches in round | 3.8 | 6.2 | 6.2 | 2.5 |
| Number of live births | 0.1 | 0.1 | 0.1 | 0.3 |
| Number of operations (self-reported) | 0 | 0 | 0 | -0.2 |
| Number of self-reported non-cancer illnesses | 0.3 | 0.5 | 0.5 | -0.7 |
| Number of treatments/medications taken | 0.2 | 0.4 | 0.4 | -0.6 |
| Osteoarthritis (self-reported) | 0.2 | 0.3 | 0.3 | 0.5 |
| Osteoporosis (self-reported) | 0 | 0 | 0 | -0.1 |
| Other serious medical condition/disability diagnosed by doctor | 0.5 | 0.6 | 0.6 | 0.8 |
| Ovarian Cancer | 0.1 | 0.2 | 0.2 | 0.4 |
| Overall health rating | 0.5 | 1 | 1 | -1 |
| Pack years adult smoking proportion | 2.1 | 3.2 | 3.2 | 1.8 |
| Pack years of smoking | 2.5 | 3.7 | 3.7 | 1.9 |
| Pain all over the body in last month | 0.3 | 0.4 | 0.4 | -0.6 |
| Pain experienced in last month | 1.4 | 2.4 | 2.4 | 1.5 |
| Pain type(s) experienced in last month: Hip pain | 0.1 | 0.2 | 0.2 | -0.4 |
| Past tobacco smoking | 1.3 | 2.3 | 2.3 | -1.5 |
| Peak expiratory flow (PEF) | 6.6 | 15.4 | 15.4 | -3.9 |
| Primary Biliary Cirrhosis | 1.6 | 2.3 | 2.3 | 1.5 |
| Prospective memory result | 0 | 0 | 0 | 0.1 |
| Prostate Cancer | 4 | 5.6 | 5.6 | 2.4 |
| Pulse rate | 1.8 | 3.1 | 3.1 | -1.8 |
| Pulse rate, automated reading | 6 | 19.4 | 19.4 | -4.4 |
| Pulse wave Arterial Stiffness index | 0.2 | 0.3 | 0.3 | 0.5 |
| Pulse wave peak to peak time | 0.5 | 0.5 | 0.5 | -0.7 |
| Pulse wave reflection index | 0 | 0.1 | 0.1 | -0.2 |
| Qualifications: A levels/AS levels or equivalent | 2.2 | 4.9 | 4.9 | -2.2 |
| Qualifications: College or University degree | 0.1 | 0.3 | 0.3 | -0.6 |
| Qualifications: CSEs or equivalent | 4.6 | 6.1 | 6.1 | -2.5 |
| Qualifications: None of the above | 1 | 2.2 | 2.2 | 1.5 |
| Qualifications: nursing, teaching | 0.9 | 1.4 | 1.4 | -1.2 |
| Qualifications: NVQ or HND or HNC or equivalent | 0.2 | 0.2 | 0.2 | 0.5 |
| Qualifications: O levels/GCSEs or equivalent | 1.7 | 2.8 | 2.8 | -1.7 |
| Reaction Time | 0 | 0 | 0 | -0.2 |
| Reason for reducing amount of alcohol drunk: Health precaution | 0 | 0 | 0 | 0 |
| Relative age of first facial hair | 0 | 0 | 0 | 0 |
| Relative age voice broke | 0.2 | 0.4 | 0.4 | 0.6 |
| Reproduciblity of spirometry measurement using ERS/ATS criteria | 0 | 0.1 | 0.1 | -0.2 |
| Respiratory disease | 0.8 | 1.7 | 1.7 | -1.3 |
| Rheumatoid Arthritis | 2.4 | 3.3 | 3.3 | 1.8 |
| Risk taking | 2.5 | 3.6 | 3.6 | 1.9 |
| Schizophrenia (2014) | 0.3 | 0.6 | 0.6 | 0.8 |
| Schizophrenia (2018) | 0 | 0 | 0 | 0.2 |
| Schizophrenia vs Biploar Disorder | 0.9 | 1.1 | 1.1 | 1.1 |
| Seen a psychiatrist for nerves, anxiety, tension or depression | 0 | 0 | 0 | 0.2 |
| Seen doctor (GP) for nerves, anxiety, tension or depression | 0 | 0 | 0 | 0.2 |
| Sensitivity / hurt feelings | 0.4 | 0.7 | 0.7 | 0.8 |
| Serious illness, injury or assault of a close relative in last 2 years | 2 | 2.3 | 2.3 | -1.5 |
| Serious illness, injury or assault to yourself | 0.6 | 0.7 | 0.7 | 0.9 |
| Severe depression (siblings) | 0.8 | 0.9 | 0.9 | -0.9 |
| Shortness of breath walking on level ground | 0 | 0 | 0 | -0.2 |
| Sitting height | 1.1 | 8 | 8 | -2.8 |
| Smoking Status | 1 | 2.5 | 2.5 | 1.6 |
| Smoking status: Current | 0.7 | 1.1 | 1.1 | 1.1 |
| Smoking status: Previous | 1.2 | 1.8 | 1.8 | 1.3 |
| Standing height | 1.4 | 14.2 | 14.2 | -3.8 |
| Stomach or abdominal pain in last month | 1.1 | 1.4 | 1.4 | -1.2 |
| Suffer from ‘nerves’ | 1 | 1.6 | 1.6 | -1.3 |
| Supplements: Calcium | 1.3 | 1.4 | 1.4 | -1.2 |
| Supplements: Fish oil (including cod liver oil) | 2.4 | 3.2 | 3.2 | -1.8 |
| Supplements: Glucosamine | 0 | 0.1 | 0.1 | -0.3 |
| Supplements: Vitamin C | 0.2 | 0.2 | 0.2 | 0.4 |
| Supplements: Zinc | 1.2 | 1.3 | 1.3 | -1.2 |
| Systolic Blood Pressure | 5.7 | 28.5 | 28.5 | -5.3 |
| Systolic blood pressure, automated reading | 8.7 | 25.4 | 25.4 | -5 |
| Taking other prescription medications | 0.2 | 0.3 | 0.3 | -0.6 |
| Tanning | 0 | 0.1 | 0.1 | -0.4 |
| Tense / ‘highly strung’ | 0 | 0.1 | 0.1 | -0.3 |
| Time employed in main current job | 1.2 | 1.4 | 1.4 | -1.2 |
| Tinnitus: Yes, but not now, but have in the past | 0.1 | 0.1 | 0.1 | 0.3 |
| Tinnitus: Yes, now most or all of the time | 1.1 | 1.3 | 1.3 | 1.1 |
| Townsend deprivation index at recruitment | 0.5 | 0.7 | 0.7 | -0.8 |
| Triglycerides | 0.5 | 0.8 | 0.8 | 0.9 |
| Trunk fat mass | 0.5 | 2.2 | 2.2 | -1.5 |
| Trunk fat percentage | 0.5 | 2.2 | 2.2 | -1.5 |
| Trunk fat-free mass | 0.2 | 0.9 | 0.9 | -1 |
| Trunk predicted mass | 0.2 | 1.1 | 1.1 | -1.1 |
| Type 2 Diabetes (T2D) (2012) | 3.1 | 3.5 | 3.5 | 1.9 |
| Type 2 Diabetes (T2D) (2018) | 0.2 | 0.3 | 0.3 | 0.5 |
| Ulcerative Colitis | 0.4 | 0.6 | 0.6 | 0.7 |
| Ulcerative Colitis (UC) | 0.7 | 1.3 | 1.3 | 1.1 |
| Unable to work because of sickness or disability | 0.5 | 0.6 | 0.6 | 0.8 |
| Vascular/heart problems diagnosed by doctor | 0.3 | 0.7 | 0.7 | 0.8 |
| Verbal and Numeric Reasoning (VNR) | 0.1 | 0.3 | 0.3 | 0.5 |
| Vitamin and mineral supplements | 0.3 | 0.4 | 0.4 | 0.6 |
| Waist circumference | 0.1 | 0.4 | 0.4 | -0.6 |
| Waist Hip Ratio (WHR) | 0.4 | 2 | 2 | -1.4 |
| Wears glasses or contact lenses | 0.1 | 0.1 | 0.1 | -0.3 |
| Weight | 0.3 | 1.5 | 1.5 | -1.2 |
| Wheeze or whistling in the chest in last year | 0.1 | 0.2 | 0.2 | 0.4 |
| Whole body fat mass | 0.4 | 1.7 | 1.7 | -1.3 |
| Whole body fat-free mass | 0.1 | 0.8 | 0.8 | -0.9 |
| Whole body water mass | 0.1 | 0.8 | 0.8 | -0.9 |
| Worrier / anxious feelings | 0.1 | 0.3 | 0.3 | 0.5 |
| Worry (Nagel 2018) | 0.4 | 0.9 | 0.9 | -1 |
| Worry too long after embarrassment | 0.8 | 1.4 | 1.4 | -1.2 |

**Supplementary Table S14. Associations with predicted tissue-specific *MSTN* expression in the TWAS Hub.** Associations were identified in the hypothalamus.

| **Trait** | **Variant** | **Position** | **-log10(p-value)** |
| --- | --- | --- | --- |
| J40-J47 Chronic lower respiratory diseases | rs4739738 | 81291645 | 10.41995362 |
| asthma | rs4739738 | 81291645 | 26.26378682 |
| J45 Asthma | rs13263709 | 81287175 | 13.47616252 |
| hypertension | rs72688070 | 81393697 | 11.13654628 |
| Reticulocyte count | rs72688070 | 81393697 | 10.19827395 |
| Nucleated red blood cell count | rs190281522 | 80422591 | 9.923359556 |
| Lymphocyte count | rs3888020 | 79716536 | 57.74446217 |
| Eosinophill count | rs13263709 | 81287175 | 13.37219393 |
| Lymphocyte percentage | rs3888020 | 79716536 | 47.07223967 |
| Monocyte percentage | rs3888020 | 79716536 | 9.292370219 |
| Neutrophill percentage | rs3888020 | 79716536 | 25.35031473 |
| Eosinophill percentage | rs13263709 | 81287175 | 14.33486009 |
| Mean platelet (thrombocyte) volume | rs182120 | 81418642 | 14.8194154 |
| Platelet distribution width | rs182120 | 81418642 | 8.849274253 |
| White blood cell (leukocyte) count | rs12677936 | 79747724 | 10.23633246 |
| Red blood cell (erythrocyte) count | rs111897958 | 81641637 | 8.402063606 |
| Haemoglobin concentration | rs575610 | 81393649 | 12.30830862 |
| Haematocrit percentage | rs61557287 | 81441012 | 11.12905795 |
| Impedance of arm (right) | rs400824 | 81357702 | 10.94119451 |
| Impedance of whole body | rs1065238 | 81397105 | 11.00283213 |
| Impedance of arm (left) | rs406629 | 81458387 | 10.68296097 |
| Impedance of leg (left) | rs1065238 | 81397105 | 9.129812711 |
| Impedance of leg (right) | rs1065238 | 81397105 | 9.112118378 |
| C43-C44 Melanoma and other malignant neoplasms of skin | rs7818943 | 81392215 | 8.723676089 |
| C44 Other malignant neoplasms of skin | rs575610 | 81393649 | 10.8293863 |
| M80 Osteoporosis with pathological fracture | rs181281104 | 80375335 | 8.162537829 |
| rosacea | rs113932653 | 80788573 | 9.424523491 |

**Supplementary Table S15. Traits associated with *HEY1* variants in GeneATLAS.** Only associations that met the threshold (p < 1e-08) are listed in the table.

| **Phenotype** | **Tissue** | **p-value** | **z-score** | **effect size** |
| --- | --- | --- | --- | --- |
| Alcohol usually taken with meals | Brain_Putamen_basal_ganglia | 0.0000228 | 4.2354 | 0.202 |
| Alcohol usually taken with meals | Pituitary | 0.0000228 | 4.2354 | 0.44817 |
| Alcohol usually taken with meals | Brain_Caudate_basal_ganglia | 0.0000228 | 4.2354 | 0.39419 |
| Alcohol usually taken with meals | Brain_Nucleus_accumbens_basal_ganglia | 0.0000228 | 4.2354 | 0.26271 |
| Job coding: senior officer in fire service including captain, commander, controller, firemaster, inspector, salvage officer, station officer, superintendent (22601_11733439) | Muscle_Skeletal | 0.0000282 | 4.1876 | 0.02163 |
| Mean platelet (thrombocyte) volume | Minor_Salivary_Gland | 0.0000442 | -4.0846 | -0.44467 |
| Job SOC coding: Senior officers in fire, ambulance, prison and related services | Muscle_Skeletal | 0.0000642 | 3.9969 | 0.027301 |
| Heel bone mineral density (BMD) | Brain_Hypothalamus | 0.0000685 | -3.9813 | -0.027887 |
| Mean platelet (thrombocyte) volume | Brain_Caudate_basal_ganglia | 0.0000731 | 3.9659 | 0.64083 |
| Mean platelet (thrombocyte) volume | Pituitary | 0.0000731 | 3.9659 | 0.72857 |
| Mean platelet (thrombocyte) volume | Brain_Putamen_basal_ganglia | 0.0000731 | 3.9659 | 0.32839 |
| Mean platelet (thrombocyte) volume | Brain_Nucleus_accumbens_basal_ganglia | 0.0000731 | 3.9659 | 0.42708 |
| Heel quantitative ultrasound index (QUI), direct entry | Brain_Hypothalamus | 0.0000965 | -3.8993 | -4.3371 |
| Heel bone mineral density (BMD) T-score, automated | Brain_Hypothalamus | 0.0000965 | -3.8991 | -0.24484 |
| Non-cancer illness code, self-reported: hypertension | Brain_Caudate_basal_ganglia | 0.000104 | 3.8819 | 0.24422 |
| Non-cancer illness code, self-reported: hypertension | Pituitary | 0.000104 | 3.8819 | 0.27766 |
| Non-cancer illness code, self-reported: hypertension | Brain_Nucleus_accumbens_basal_ganglia | 0.000104 | 3.8819 | 0.16276 |
| Non-cancer illness code, self-reported: hypertension | Brain_Putamen_basal_ganglia | 0.000104 | 3.8819 | 0.12515 |
| Seen doctor (GP) for nerves, anxiety, tension or depression | Muscle_Skeletal | 0.000106 | -3.877 | -0.12325 |
| Job SOC coding: Electrical/electronics technicians | Whole_Blood | 0.000122 | 3.8411 | 0.0060996 |
| Vitamin E | Brain_Hypothalamus | 0.000137 | 3.8144 | 2.2331 |
| Non-cancer illness code, self-reported: liver failure/cirrhosis | Muscle_Skeletal | 0.00015 | -3.7912 | -0.006931 |
| Vascular/heart problems diagnosed by doctor: None of the above | Brain_Nucleus_accumbens_basal_ganglia | 0.000151 | -3.7889 | -0.16446 |
| Vascular/heart problems diagnosed by doctor: None of the above | Pituitary | 0.000151 | -3.7889 | -0.28055 |
| Vascular/heart problems diagnosed by doctor: None of the above | Brain_Putamen_basal_ganglia | 0.000151 | -3.7889 | -0.12645 |
| Vascular/heart problems diagnosed by doctor: None of the above | Brain_Caudate_basal_ganglia | 0.000151 | -3.7889 | -0.24677 |
| Vascular/heart problems diagnosed by doctor: High blood pressure | Brain_Nucleus_accumbens_basal_ganglia | 0.000168 | 3.763 | 0.16025 |
| Vascular/heart problems diagnosed by doctor: High blood pressure | Brain_Putamen_basal_ganglia | 0.000168 | 3.763 | 0.12322 |
| Vascular/heart problems diagnosed by doctor: High blood pressure | Pituitary | 0.000168 | 3.763 | 0.27337 |
| Vascular/heart problems diagnosed by doctor: High blood pressure | Brain_Caudate_basal_ganglia | 0.000168 | 3.763 | 0.24045 |
| GI-bleeding | Brain_Hypothalamus | 0.000181 | -3.7444 | -0.017998 |
| Easily tired during worst period of anxiety | Whole_Blood | 0.000194 | 3.7266 | 0.042842 |
| Diagnoses - main ICD10: J39 Other diseases of upper respiratory tract | Minor_Salivary_Gland | 0.000235 | -3.6776 | -0.012296 |
| Speech-reception-threshold (SRT) estimate (right) | Minor_Salivary_Gland | 0.000264 | 3.6482 | 1.2461 |
| Job coding: draughtsperson, cartographer, designer-detailer/draughtsperson, drawing office checker, engineering draughtsperson (22601_31223293) | Testis | 0.000283 | 3.6302 | 0.0075874 |
| Non-cancer illness code, self-reported: pneumonia | Whole_Blood | 0.000308 | 3.6083 | 0.0033902 |
| Alcohol usually taken with meals | Minor_Salivary_Gland | 0.00032 | -3.5985 | -0.22568 |
| Speech-reception-threshold (SRT) estimate (right) | Brain_Caudate_basal_ganglia | 0.000335 | -3.5867 | -1.8179 |
| Speech-reception-threshold (SRT) estimate (right) | Brain_Putamen_basal_ganglia | 0.000335 | -3.5867 | -0.93156 |
| Speech-reception-threshold (SRT) estimate (right) | Brain_Nucleus_accumbens_basal_ganglia | 0.000335 | -3.5867 | -1.2115 |
| Speech-reception-threshold (SRT) estimate (right) | Pituitary | 0.000335 | -3.5867 | -2.0668 |
| Treatment/medication code: spironolactone (20003_1140866236) | Muscle_Skeletal | 0.000341 | -3.5822 | -0.010362 |
| Treatment/medication code: loperamide (20003_1140879464) | Minor_Salivary_Gland | 0.000348 | 3.5765 | 0.020535 |
| Diagnoses - main ICD10: K92 Other diseases of digestive system | Brain_Hypothalamus | 0.000351 | -3.5748 | -0.017459 |
| Job SOC coding: Draughtspersons | Testis | 0.000366 | 3.5632 | 0.0074345 |
| Job SOC coding: Careers advisers and vocational guidance specialists | Muscle_Skeletal | 0.000468 | -3.4984 | -0.030718 |
| Schizophrenia | Muscle_Skeletal | 0.000479 | -3.492 |  |
| Job coding: travel agency manager/owner, tourist manager (22601_12263368) | Muscle_Skeletal | 0.000485 | 3.489 | 0.020781 |
| Treatment/medication code: loperamide (20003_1140879464) | Pituitary | 0.000501 | -3.4804 | -0.033719 |
| Treatment/medication code: loperamide (20003_1140879464) | Brain_Nucleus_accumbens_basal_ganglia | 0.000501 | -3.4804 | -0.019766 |
| Treatment/medication code: loperamide (20003_1140879464) | Brain_Caudate_basal_ganglia | 0.000501 | -3.4804 | -0.029658 |
| Treatment/medication code: loperamide (20003_1140879464) | Brain_Putamen_basal_ganglia | 0.000501 | -3.4804 | -0.015198 |
| Past tobacco smoking | Muscle_Skeletal | 0.000528 | 3.4662 | 0.3022 |
| Job SOC coding: Legal secretaries | Testis | 0.000538 | 3.461 | 0.013615 |
| Treatment/medication code: vitamin b12 preparation (20003_1140870570) | Whole_Blood | 0.000549 | -3.4558 | -0.0015159 |
| Tobacco smoking: Never smoked | Muscle_Skeletal | 0.000572 | 3.4446 | 0.22448 |
| Reason for reducing amount of alcohol drunk: Financial reasons | Minor_Salivary_Gland | 0.000578 | 3.4418 | 0.12513 |
| Injury, poisoning and certain other consequences of external causes | Whole_Blood | 0.000684 | -3.3959 | -0.008816 |
| Job SOC coding: Travel agency managers | Muscle_Skeletal | 0.000688 | 3.3942 | 0.020053 |
| Treatment/medication code: diclofenac (20003_1140884488) | Brain_Hypothalamus | 0.000779 | 3.3602 | 0.019328 |
| Distance between home and job workplace | Whole_Blood | 0.000788 | 3.3569 | 3.3326 |
| Number of days/week of vigorous physical activity 10+ minutes | Muscle_Skeletal | 0.000803 | -3.3518 | -0.44906 |
| Ever had known person concerned about, or recommend reduction of, alcohol consumption: Yes, but not in the last year | Brain_Hypothalamus | 0.000838 | 3.3398 | 0.049039 |
| Diagnoses - main ICD10: C22 Malignant neoplasm of liver and intrahepatic bile ducts | Brain_Hypothalamus | 0.000855 | -3.3345 | -0.0034428 |
| Smoking status: Previous | Muscle_Skeletal | 0.000897 | -3.321 | -0.10607 |
| Diagnoses - main ICD10: J39 Other diseases of upper respiratory tract | Brain_Nucleus_accumbens_basal_ganglia | 0.000908 | 3.3177 | 0.010972 |
| Diagnoses - main ICD10: J39 Other diseases of upper respiratory tract | Brain_Putamen_basal_ganglia | 0.000908 | 3.3177 | 0.0084366 |
| Diagnoses - main ICD10: J39 Other diseases of upper respiratory tract | Pituitary | 0.000908 | 3.3177 | 0.018717 |
| Diagnoses - main ICD10: J39 Other diseases of upper respiratory tract | Brain_Caudate_basal_ganglia | 0.000908 | 3.3177 | 0.016463 |
| Job coding: aircraft engineer (22601_52232942) | Muscle_Skeletal | 0.000943 | 3.3071 | 0.017402 |
| How are people in household related to participant: Grandchild | Brain_Hypothalamus | 0.000992 | -3.2929 | -0.011913 |
| Tea intake | Pituitary | 0.000995 | -3.2919 | -1.6217 |
| Tea intake | Brain_Caudate_basal_ganglia | 0.000995 | -3.2919 | -1.4264 |
| Tea intake | Brain_Putamen_basal_ganglia | 0.000995 | -3.2919 | -0.73096 |
| Tea intake | Brain_Nucleus_accumbens_basal_ganglia | 0.000995 | -3.2919 | -0.95063 |
| Frequency of unusual or psychotic experiences in past year | Muscle_Skeletal | 0.00102 | 3.2853 | 1.5705 |
| Heart failure | Muscle_Skeletal | 0.00115 | -3.2513 | -0.013664 |
| Heart failure,strict | Muscle_Skeletal | 0.00115 | -3.2513 | -0.013664 |
| Heart failure, not strict | Muscle_Skeletal | 0.00115 | -3.2513 | -0.013664 |
| Diagnoses - main ICD10: H80 Otosclerosis | Whole_Blood | 0.0012 | 3.2379 | 0.00063486 |
| Total traffic load on major roads | Pituitary | 0.00124 | -3.2291 | -0.29913 |
| Total traffic load on major roads | Brain_Nucleus_accumbens_basal_ganglia | 0.00124 | -3.2291 | -0.17535 |
| Total traffic load on major roads | Brain_Putamen_basal_ganglia | 0.00124 | -3.2291 | -0.13483 |
| Total traffic load on major roads | Brain_Caudate_basal_ganglia | 0.00124 | -3.2291 | -0.26311 |
| Platelet distribution width | Minor_Salivary_Gland | 0.00138 | -3.1988 | -0.16572 |
| Cancer code, self-reported: non-hodgkins lymphoma | Brain_Nucleus_accumbens_basal_ganglia | 0.00143 | -3.1893 | -0.013669 |
| Cancer code, self-reported: non-hodgkins lymphoma | Brain_Putamen_basal_ganglia | 0.00143 | -3.1893 | -0.01051 |
| Cancer code, self-reported: non-hodgkins lymphoma | Pituitary | 0.00143 | -3.1893 | -0.023318 |
| Cancer code, self-reported: non-hodgkins lymphoma | Brain_Caudate_basal_ganglia | 0.00143 | -3.1893 | -0.02051 |
| Mature T/NK-cell lymphomas | Whole_Blood | 0.00147 | -3.1807 | -0.00048401 |
| Type of fat/oil used in cooking: Rapeseed oil | Brain_Putamen_basal_ganglia | 0.00148 | -3.1789 | -0.096501 |
| Type of fat/oil used in cooking: Rapeseed oil | Brain_Nucleus_accumbens_basal_ganglia | 0.00148 | -3.1789 | -0.1255 |
| Type of fat/oil used in cooking: Rapeseed oil | Brain_Caudate_basal_ganglia | 0.00148 | -3.1789 | -0.18831 |
| Type of fat/oil used in cooking: Rapeseed oil | Pituitary | 0.00148 | -3.1789 | -0.2141 |
| Average evening sound level of noise pollution | Brain_Caudate_basal_ganglia | 0.00149 | -3.1757 | -1.9935 |
| Average evening sound level of noise pollution | Brain_Putamen_basal_ganglia | 0.00149 | -3.1757 | -1.0216 |
| Average evening sound level of noise pollution | Brain_Nucleus_accumbens_basal_ganglia | 0.00149 | -3.1757 | -1.3286 |
| Average evening sound level of noise pollution | Pituitary | 0.00149 | -3.1757 | -2.2665 |
| Average 16-hour sound level of noise pollution | Brain_Putamen_basal_ganglia | 0.0015 | -3.1752 | -1.0214 |
| Average 16-hour sound level of noise pollution | Pituitary | 0.0015 | -3.1752 | -2.2661 |
| Average 16-hour sound level of noise pollution | Brain_Nucleus_accumbens_basal_ganglia | 0.0015 | -3.1752 | -1.3284 |
| Average daytime sound level of noise pollution | Brain_Nucleus_accumbens_basal_ganglia | 0.0015 | -3.1752 | -1.3284 |
| Average daytime sound level of noise pollution | Brain_Caudate_basal_ganglia | 0.0015 | -3.1752 | -1.9932 |
| Average daytime sound level of noise pollution | Brain_Putamen_basal_ganglia | 0.0015 | -3.1752 | -1.0214 |
| Average daytime sound level of noise pollution | Pituitary | 0.0015 | -3.1752 | -2.2661 |
| Average 16-hour sound level of noise pollution | Brain_Caudate_basal_ganglia | 0.0015 | -3.1752 | -1.9932 |
| Average night-time sound level of noise pollution | Brain_Nucleus_accumbens_basal_ganglia | 0.0015 | -3.175 | -1.3283 |
| Average night-time sound level of noise pollution | Brain_Putamen_basal_ganglia | 0.0015 | -3.175 | -1.0214 |
| Average night-time sound level of noise pollution | Pituitary | 0.0015 | -3.175 | -2.266 |
| Average night-time sound level of noise pollution | Brain_Caudate_basal_ganglia | 0.0015 | -3.175 | -1.9931 |
| Average 24-hour sound level of noise pollution | Brain_Nucleus_accumbens_basal_ganglia | 0.0015 | -3.175 | -1.3283 |
| Average 24-hour sound level of noise pollution | Brain_Putamen_basal_ganglia | 0.0015 | -3.175 | -1.0214 |
| Average 24-hour sound level of noise pollution | Pituitary | 0.0015 | -3.175 | -2.266 |
| Average 24-hour sound level of noise pollution | Brain_Caudate_basal_ganglia | 0.0015 | -3.175 | -1.9931 |
| Treatment/medication code: aloe vera product (20003_1203) | Testis | 0.00159 | 3.157 | 0.0018599 |
| Treatment/medication code: paracetamol (20003_2038460150) | Brain_Hypothalamus | 0.0016 | 3.1561 | 0.048837 |
| Treatment/medication code: omeprazole (20003_1140865634) | Muscle_Skeletal | 0.00169 | -3.1396 | -0.049571 |
| Job coding: nurse (of any kind, at any level) (22601_32113072) | Whole_Blood | 0.0017 | 3.1387 | 0.011053 |
| Pain type(s) experienced in last month: None of the above | Minor_Salivary_Gland | 0.00172 | 3.134 | 0.15324 |
| Job coding: electrical or electronics technician, signals officer (22601_31123049) | Whole_Blood | 0.00175 | 3.1289 | 0.0037669 |
| Tea intake | Minor_Salivary_Gland | 0.00176 | 3.1286 | 0.91322 |
| Type of fat/oil used in cooking: Rapeseed oil | Minor_Salivary_Gland | 0.00176 | 3.1272 | 0.12496 |
| Impaction of intestines | Whole_Blood | 0.00186 | -3.1117 | -0.00045784 |
| Treatment/medication code: detrusitol 1mg tablet (20003_1141162824) | Whole_Blood | 0.00189 | -3.1075 | -0.00096136 |
| Job coding: quality assurance manager, quality control manager (22601_11412694) | Muscle_Skeletal | 0.00189 | 3.1075 | 0.023985 |
| Mental health problems ever diagnosed by a professional: Psychological over-eating or binge-eating | Brain_Putamen_basal_ganglia | 0.00199 | -3.0922 | -0.026794 |
| Mental health problems ever diagnosed by a professional: Psychological over-eating or binge-eating | Brain_Nucleus_accumbens_basal_ganglia | 0.00199 | -3.0922 | -0.034846 |
| Mental health problems ever diagnosed by a professional: Psychological over-eating or binge-eating | Pituitary | 0.00199 | -3.0922 | -0.059444 |
| Mental health problems ever diagnosed by a professional: Psychological over-eating or binge-eating | Brain_Caudate_basal_ganglia | 0.00199 | -3.0922 | -0.052286 |
| Endocarditis | Brain_Putamen_basal_ganglia | 0.00203 | -3.0862 | -0.0057051 |
| Endocarditis | Brain_Nucleus_accumbens_basal_ganglia | 0.00203 | -3.0862 | -0.0074197 |
| Endocarditis | Pituitary | 0.00203 | -3.0862 | -0.012657 |
| Endocarditis | Brain_Caudate_basal_ganglia | 0.00203 | -3.0862 | -0.011133 |
| Non-cancer illness code, self-reported: brain haemorrhage | Muscle_Skeletal | 0.00206 | 3.0821 | 0.0036382 |
| Treatment/medication code: pariet 10mg e/c tablet (20003_1141168590) | Whole_Blood | 0.00212 | -3.0735 | -0.00062497 |
| Cancer code, self-reported: multiple myeloma | Minor_Salivary_Gland | 0.00212 | 3.0731 | 0.0056974 |
| Pulse rate, automated reading | Muscle_Skeletal | 0.00215 | -3.0693 | -2.4033 |
| Diagnoses - main ICD10: I33 Acute and subacute endocarditis | Muscle_Skeletal | 0.00216 | -3.0669 | -0.0043375 |
| Non-cancer illness code, self-reported: iron deficiency anaemia | Minor_Salivary_Gland | 0.00218 | -3.064 | -0.022566 |
| Job SOC coding: Chartered and certified accountants | Whole_Blood | 0.00221 | -3.0605 | -0.0069813 |
| Use of sun/uv protection | Brain_Putamen_basal_ganglia | 0.00224 | 3.0569 | 0.20035 |
| Use of sun/uv protection | Brain_Caudate_basal_ganglia | 0.00224 | 3.0569 | 0.39096 |
| Use of sun/uv protection | Brain_Nucleus_accumbens_basal_ganglia | 0.00224 | 3.0569 | 0.26056 |
| Use of sun/uv protection | Pituitary | 0.00224 | 3.0569 | 0.44449 |
| Close to major road | Pituitary | 0.00224 | -3.0569 | -0.13114 |
| Close to major road | Brain_Caudate_basal_ganglia | 0.00224 | -3.0569 | -0.11534 |
| Close to major road | Brain_Putamen_basal_ganglia | 0.00224 | -3.0569 | -0.059107 |
| Close to major road | Brain_Nucleus_accumbens_basal_ganglia | 0.00224 | -3.0569 | -0.076871 |
| Reason for reducing amount of alcohol drunk: Financial reasons | Brain_Caudate_basal_ganglia | 0.00226 | -3.0532 | -0.16473 |
| Reason for reducing amount of alcohol drunk: Financial reasons | Brain_Nucleus_accumbens_basal_ganglia | 0.00226 | -3.0532 | -0.10978 |
| Reason for reducing amount of alcohol drunk: Financial reasons | Brain_Putamen_basal_ganglia | 0.00226 | -3.0532 | -0.084414 |
| Reason for reducing amount of alcohol drunk: Financial reasons | Pituitary | 0.00226 | -3.0532 | -0.18728 |
| Job coding: health visitor (22601_32113077) | Brain_Hypothalamus | 0.00236 | 3.0409 | 0.016328 |
| Ever unenthusiastic/disinterested for a whole week | Muscle_Skeletal | 0.00237 | -3.04 | -0.17449 |
| Treatment/medication code: sertraline (20003_1140867878) | Minor_Salivary_Gland | 0.00237 | 3.0398 | 0.019682 |
| Anxiety disorders | Muscle_Skeletal | 0.00239 | -3.0371 | -0.011278 |
| Diagnoses - main ICD10: I50 Heart failure | Muscle_Skeletal | 0.00239 | -3.0366 | -0.012566 |
| Own or rent accommodation lived in: Own with a mortgage | Whole_Blood | 0.00241 | -3.034 | -0.010137 |
| Total traffic load on major roads | Minor_Salivary_Gland | 0.00243 | 3.0315 | 0.16645 |
| How are people in household related to participant: Husband, wife or partner | Minor_Salivary_Gland | 0.00244 | -3.0305 | -0.13214 |
| Age of stopping smoking | Brain_Caudate_basal_ganglia | 0.00246 | -3.0283 | -17.514 |
| Age of stopping smoking | Brain_Nucleus_accumbens_basal_ganglia | 0.00246 | -3.0283 | -11.672 |
| Age of stopping smoking | Brain_Putamen_basal_ganglia | 0.00246 | -3.0283 | -8.9751 |
| Age of stopping smoking | Pituitary | 0.00246 | -3.0283 | -19.912 |
| Medication for pain relief, constipation, heartburn: Omeprazole (e.g. Zanprol) | Muscle_Skeletal | 0.00253 | -3.0197 | -0.048565 |
| Diagnoses - main ICD10: B18 Chronic viral hepatitis | Brain_Hypothalamus | 0.00254 | 3.0186 | 0.0024331 |
| Treatment/medication code: oxytetracycline (20003_1140873548) | Testis | 0.00255 | 3.0171 | 0.0030599 |
| Workplace had a lot of diesel exhaust: Sometimes | Whole_Blood | 0.00255 | -3.017 | -0.016316 |
| Workplace very cold: Often | Brain_Hypothalamus | 0.00267 | 3.0036 | 0.067539 |
| Non-cancer illness code, self-reported: rosacea | Muscle_Skeletal | 0.00282 | -2.9866 | -0.0081244 |
| Oily fish intake | Whole_Blood | 0.00287 | 2.9817 | 0.021413 |
| Endocrine, nutritional and metabolic diseases | Minor_Salivary_Gland | 0.0029 | 2.9786 | 0.041527 |
| Cancer code, self-reported: leukaemia | Pituitary | 0.00292 | 2.9762 | 0.010825 |
| Cancer code, self-reported: leukaemia | Brain_Nucleus_accumbens_basal_ganglia | 0.00292 | 2.9762 | 0.0063457 |
| Cancer code, self-reported: leukaemia | Brain_Putamen_basal_ganglia | 0.00292 | 2.9762 | 0.0048793 |
| Cancer code, self-reported: leukaemia | Brain_Caudate_basal_ganglia | 0.00292 | 2.9762 | 0.0095216 |
| Destinations on discharge from hospital (recoded): Usual Place of residence: Living with relatives | Brain_Putamen_basal_ganglia | 0.00298 | 2.9695 | 0.035812 |
| Destinations on discharge from hospital (recoded): Usual Place of residence: Living with relatives | Brain_Nucleus_accumbens_basal_ganglia | 0.00298 | 2.9695 | 0.046574 |
| Destinations on discharge from hospital (recoded): Usual Place of residence: Living with relatives | Pituitary | 0.00298 | 2.9695 | 0.079452 |
| Destinations on discharge from hospital (recoded): Usual Place of residence: Living with relatives | Brain_Caudate_basal_ganglia | 0.00298 | 2.9695 | 0.069884 |
| Pork intake | Brain_Hypothalamus | 0.00299 | -2.9686 | -0.083993 |
| Tobacco smoking: Ex-smoker | Muscle_Skeletal | 0.003 | -2.9681 | -0.1894 |
| Close to major road | Minor_Salivary_Gland | 0.00301 | 2.9667 | 0.075431 |
| Treatment/medication code: gaviscon liquid (20003_1140865354) | Muscle_Skeletal | 0.00308 | -2.9594 | -0.014418 |
| Diagnoses - main ICD10: Z12 Special screening examination for neoplasms | Minor_Salivary_Gland | 0.00311 | -2.9569 | -0.031708 |
| Job SOC coding: Nurses | Whole_Blood | 0.00312 | 2.9559 | 0.010463 |
| Treatment/medication code: ibandronic acid (20003_1141180314) | Whole_Blood | 0.00312 | 2.9556 | 0.00070812 |
| Non-cancer illness code, self-reported: neurological injury/trauma | Brain_Hypothalamus | 0.00313 | 2.9546 | 0.0020174 |
| Cancer code, self-reported: non-hodgkins lymphoma | Minor_Salivary_Gland | 0.00313 | 2.9546 | 0.012802 |
| Job coding: legal secretary (22601_42123299) | Testis | 0.00316 | 2.9521 | 0.0090928 |
| Job coding: chartered/certified accountant, auditor, company accountant, articled/audit clerk, official receiver (22601_24213281) | Whole_Blood | 0.00326 | -2.9426 | -0.0067145 |
| Non-cancer illness code, self-reported: gall bladder disease | Brain_Caudate_basal_ganglia | 0.00326 | -2.9425 | -0.010292 |
| Non-cancer illness code, self-reported: gall bladder disease | Brain_Putamen_basal_ganglia | 0.00326 | -2.9425 | -0.0052739 |
| Non-cancer illness code, self-reported: gall bladder disease | Brain_Nucleus_accumbens_basal_ganglia | 0.00326 | -2.9425 | -0.0068588 |
| Non-cancer illness code, self-reported: gall bladder disease | Pituitary | 0.00326 | -2.9425 | -0.011701 |
| Cancer code, self-reported: multiple myeloma | Brain_Nucleus_accumbens_basal_ganglia | 0.00333 | -2.9352 | -0.0053826 |
| Cancer code, self-reported: multiple myeloma | Brain_Putamen_basal_ganglia | 0.00333 | -2.9352 | -0.0041387 |
| Cancer code, self-reported: multiple myeloma | Pituitary | 0.00333 | -2.9352 | -0.0091822 |
| Diagnoses - main ICD10: C91 Lymphoid leukaemia | Whole_Blood | 0.00343 | -2.9261 | -0.00075575 |
| Diagnoses - main ICD10: K70 Alcoholic liver disease | Muscle_Skeletal | 0.00344 | -2.9251 | -0.006462 |
| Treatment/medication code: ibandronic acid (20003_1141180314) | Testis | 0.00346 | -2.9234 | -0.0016485 |
| Medication for cholesterol, blood pressure, diabetes, or take exogenous hormones: Blood pressure medication | Brain_Putamen_basal_ganglia | 0.0035 | 2.9197 | 0.11116 |
| Medication for cholesterol, blood pressure, diabetes, or take exogenous hormones: Blood pressure medication | Brain_Caudate_basal_ganglia | 0.0035 | 2.9197 | 0.21693 |
| Medication for cholesterol, blood pressure, diabetes, or take exogenous hormones: Blood pressure medication | Pituitary | 0.0035 | 2.9197 | 0.24663 |
| Medication for cholesterol, blood pressure, diabetes, or take exogenous hormones: Blood pressure medication | Brain_Nucleus_accumbens_basal_ganglia | 0.0035 | 2.9197 | 0.14457 |
| Diagnoses - main ICD10: C82 Follicular [nodular] non-Hodgkin's lymphoma | Brain_Hypothalamus | 0.00351 | 2.9189 | 0.003706 |
| Diagnoses - main ICD10: I85 Oesophageal varices | Muscle_Skeletal | 0.00352 | -2.9179 | -0.0061488 |
| Treatment/medication code: adalate 10mg capsule (20003_1140881702) | Muscle_Skeletal | 0.00365 | 2.9066 | 0.0054124 |
| Number of vehicles in household | Brain_Hypothalamus | 0.00374 | -2.8996 | -0.098762 |
| Non-cancer illness code, self-reported: anal fissure | Testis | 0.0039 | 2.8865 | 0.0012259 |
| Astigmatism angle (right) | Muscle_Skeletal | 0.00391 | 2.8855 | 21.371 |
| Diagnoses - main ICD10: J38 Diseases of vocal cords and larynx, not elsewhere classified | Brain_Putamen_basal_ganglia | 0.00393 | -2.8841 | -0.012835 |
| Diagnoses - main ICD10: J38 Diseases of vocal cords and larynx, not elsewhere classified | Pituitary | 0.00393 | -2.8841 | -0.028476 |
| Diagnoses - main ICD10: J38 Diseases of vocal cords and larynx, not elsewhere classified | Brain_Caudate_basal_ganglia | 0.00393 | -2.8841 | -0.025046 |
| Diagnoses - main ICD10: J38 Diseases of vocal cords and larynx, not elsewhere classified | Brain_Nucleus_accumbens_basal_ganglia | 0.00393 | -2.8841 | -0.016692 |
| Current employment status: None of the above | Whole_Blood | 0.00396 | -2.881 | -0.001577 |
| Smoking status: Never | Muscle_Skeletal | 0.004 | 2.8785 | 0.096051 |
| Illnesses of adopted siblings: Severe depression | Brain_Hypothalamus | 0.00405 | 2.8741 | 0.44652 |
| Non-cancer illness code, self-reported: gall bladder disease | Minor_Salivary_Gland | 0.00409 | 2.8714 | 0.0067667 |
| How are people in household related to participant: Brother and/or sister | Muscle_Skeletal | 0.00411 | -2.8698 | -0.013182 |
| Viral hepatitis, IBD co-morbidity | Brain_Hypothalamus | 0.00412 | 2.8686 | 0.0028735 |
| Job coding: medical secretary (22601_42113298) | Whole_Blood | 0.00414 | -2.8676 | -0.0044174 |
| Blood clot, DVT, bronchitis, emphysema, asthma, rhinitis, eczema, allergy diagnosed by doctor: Emphysema/chronic bronchitis | Muscle_Skeletal | 0.00416 | -2.8658 | -0.025047 |
| Diagnoses - main ICD10: I74 Arterial embolism and thrombosis | Brain_Hypothalamus | 0.00416 | -2.8657 | -0.0045986 |
| Pain type(s) experienced in last month: None of the above | Brain_Putamen_basal_ganglia | 0.00423 | -2.8601 | -0.10637 |
| Pain type(s) experienced in last month: None of the above | Pituitary | 0.00423 | -2.8601 | -0.23599 |
| Pain type(s) experienced in last month: None of the above | Brain_Nucleus_accumbens_basal_ganglia | 0.00423 | -2.8601 | -0.13834 |
| Pain type(s) experienced in last month: None of the above | Brain_Caudate_basal_ganglia | 0.00423 | -2.8601 | -0.20757 |
| Ever stopped smoking for 6+ months | Testis | 0.00424 | 2.8594 | 0.053277 |
| Diagnoses - main ICD10: D11 Benign neoplasm of major salivary glands | Whole_Blood | 0.00425 | 2.859 | 0.00075119 |
| Diagnoses - main ICD10: R73 Elevated blood glucose level | Whole_Blood | 0.0043 | 2.8556 | 0.00049142 |
| Treatment/medication code: brimonidine tartrate (20003_1141150750) | Minor_Salivary_Gland | 0.0043 | -2.8556 | -0.0048057 |
| Ptosis of eyelid | Minor_Salivary_Gland | 0.00433 | -2.8533 | -0.014319 |
| Non-cancer illness code, self-reported: non-infective hepatitis | Brain_Hypothalamus | 0.00436 | -2.8509 | -0.0036902 |
| Non-cancer illness code, self-reported: muscle or soft tissue injuries | Testis | 0.00439 | -2.8485 | -0.0045261 |
| Treatment/medication code: qvar 50 inhaler (20003_1141167594) | Minor_Salivary_Gland | 0.00441 | -2.8476 | -0.017392 |
| Own or rent accommodation lived in: Pay part rent and part mortgage (shared ownership) | Muscle_Skeletal | 0.00441 | 2.8474 | 0.0098359 |
| Non-cancer illness code, self-reported: hypertension | Minor_Salivary_Gland | 0.00443 | -2.8458 | -0.12063 |
| Arterial embolism and thrombosis | Brain_Hypothalamus | 0.00447 | -2.843 | -0.0045702 |
| Other/unspecified synovitis and tenosynovitis | Brain_Hypothalamus | 0.0045 | 2.8409 | 0.0047664 |
| Peak expiratory flow (PEF) | Muscle_Skeletal | 0.0045 | 2.8407 | 20.604 |
| Amount of alcohol drunk on a typical drinking day | Muscle_Skeletal | 0.00451 | -2.8403 | -0.35172 |
| Job coding: it operations technician, computer operator or technician, database manager, network or web technician, web master, systems administrator or officer (22601_31313060) | Pituitary | 0.00451 | -2.8398 | -0.10404 |
| Job coding: it operations technician, computer operator or technician, database manager, network or web technician, web master, systems administrator or officer (22601_31313060) | Brain_Nucleus_accumbens_basal_ganglia | 0.00451 | -2.8398 | -0.06099 |
| Job coding: it operations technician, computer operator or technician, database manager, network or web technician, web master, systems administrator or officer (22601_31313060) | Brain_Caudate_basal_ganglia | 0.00451 | -2.8398 | -0.091515 |
| Job coding: it operations technician, computer operator or technician, database manager, network or web technician, web master, systems administrator or officer (22601_31313060) | Brain_Putamen_basal_ganglia | 0.00451 | -2.8398 | -0.046896 |
| Non-cancer illness code, self-reported: infective/viral hepatitis | Testis | 0.00454 | 2.8377 | 0.0024339 |
| Other follicular disorders | Brain_Hypothalamus | 0.00463 | -2.8319 | -0.0029968 |
| Diagnoses - main ICD10: L73 Other follicular disorders | Brain_Hypothalamus | 0.00463 | -2.8319 | -0.0029968 |
| Job code - deduced: Production, works and maintenance managers | Minor_Salivary_Gland | 0.00466 | 2.83 | 0.26926 |
| Tea intake | Brain_Hypothalamus | 0.0047 | 2.8266 | 0.3332 |
| Diagnoses - main ICD10: R17 Unspecified jaundice | Muscle_Skeletal | 0.00474 | 2.8239 | 0.0058226 |
| Job SOC coding: Motor mechanics, auto engineers | Muscle_Skeletal | 0.0048 | 2.8204 | 0.034767 |
| Transport type for commuting to job workplace: Cycle | Testis | 0.0048 | -2.8203 | -0.019124 |
| Treatment/medication code: brimonidine tartrate (20003_1141150750) | Pituitary | 0.00484 | 2.8174 | 0.0080006 |
| Treatment/medication code: brimonidine tartrate (20003_1141150750) | Brain_Putamen_basal_ganglia | 0.00484 | 2.8174 | 0.0036061 |
| Treatment/medication code: brimonidine tartrate (20003_1141150750) | Brain_Nucleus_accumbens_basal_ganglia | 0.00484 | 2.8174 | 0.0046899 |
| Treatment/medication code: brimonidine tartrate (20003_1141150750) | Brain_Caudate_basal_ganglia | 0.00484 | 2.8174 | 0.0070371 |
| Treatment/medication code: quinine (20003_1140874420) | Whole_Blood | 0.00499 | 2.8074 | 0.0018028 |
| Diagnoses - main ICD10: H80 Otosclerosis | Testis | 0.00513 | -2.7986 | -0.0012943 |
| Mood swings | Muscle_Skeletal | 0.00515 | -2.7973 | -0.094431 |
| Job SOC coding: Bus and coach drivers | Testis | 0.00519 | 2.7947 | 0.0073017 |
| Diagnoses - main ICD10: J93 Pneumothorax | Minor_Salivary_Gland | 0.00527 | -2.7899 | -0.0082846 |
| Morning/evening person (chronotype) | Brain_Nucleus_accumbens_basal_ganglia | 0.00528 | -2.7895 | -0.26933 |
| Morning/evening person (chronotype) | Pituitary | 0.00528 | -2.7895 | -0.45945 |
| Morning/evening person (chronotype) | Brain_Putamen_basal_ganglia | 0.00528 | -2.7895 | -0.20709 |
| Morning/evening person (chronotype) | Brain_Caudate_basal_ganglia | 0.00528 | -2.7895 | -0.40412 |
| Ever been injured or injured someone else through drinking alcohol: Yes, during the last year | Brain_Hypothalamus | 0.00532 | 2.7871 | 0.013325 |
| Diagnoses - main ICD10: M51 Other intervertebral disk disorders | Brain_Putamen_basal_ganglia | 0.00549 | -2.7769 | -0.024433 |
| Diagnoses - main ICD10: M51 Other intervertebral disk disorders | Brain_Nucleus_accumbens_basal_ganglia | 0.00549 | -2.7769 | -0.031776 |
| Diagnoses - main ICD10: M51 Other intervertebral disk disorders | Brain_Caudate_basal_ganglia | 0.00549 | -2.7769 | -0.04768 |
| Diagnoses - main ICD10: M51 Other intervertebral disk disorders | Pituitary | 0.00549 | -2.7769 | -0.054208 |
| Birth weight | Brain_Hypothalamus | 0.00549 | -2.7767 | -0.097531 |
| Heating type(s) in home: Open fire without central heating | Brain_Putamen_basal_ganglia | 0.00552 | -2.7749 | -0.022546 |
| Heating type(s) in home: Open fire without central heating | Brain_Nucleus_accumbens_basal_ganglia | 0.00552 | -2.7749 | -0.029322 |
| Heating type(s) in home: Open fire without central heating | Brain_Caudate_basal_ganglia | 0.00552 | -2.7749 | -0.043997 |
| Heating type(s) in home: Open fire without central heating | Pituitary | 0.00552 | -2.7749 | -0.050021 |
| Viral hepatitis | Brain_Hypothalamus | 0.00553 | 2.7743 | 0.0028945 |
| Own or rent accommodation lived in: Own with a mortgage | Testis | 0.00558 | 2.7717 | 0.021901 |
| Ever taken cannabis | Muscle_Skeletal | 0.0056 | -2.7706 | -0.30133 |
| Ohter specific/unspecified arthritis | Brain_Hypothalamus | 0.00564 | 2.7678 | 0.0060853 |
| Illnesses of father: Severe depression | Brain_Hypothalamus | 0.0057 | 2.7647 | 0.022381 |
| Hidradenitis suppurativa | Testis | 0.00572 | 2.7632 | 0.00099433 |
| Red blood cell (erythrocyte) distribution width | Muscle_Skeletal | 0.00576 | -2.7611 | -0.17907 |
| Recent medication for asthma | Muscle_Skeletal | 0.00579 | 2.7597 | 0.50479 |
| Job SOC coding: Plumbers, heating and ventilating engineers | Muscle_Skeletal | 0.00582 | -2.7577 | -0.025305 |
| Job SOC coding: Further education teaching professionals | Testis | 0.00584 | -2.7568 | -0.016725 |
| Diagnoses - main ICD10: C61 Malignant neoplasm of prostate | Whole_Blood | 0.00586 | -2.7555 | -0.0023958 |
| Diagnoses - main ICD10: F52 Sexual dysfunction, not caused by organic disorder or disease | Muscle_Skeletal | 0.00591 | 2.7527 | 0.0035847 |
| Time to complete round | Testis | 0.00593 | 2.7518 | 5.4685 |
| Destinations on discharge from hospital (recoded): Usual Place of residence: Living with relatives | Minor_Salivary_Gland | 0.006 | -2.7475 | -0.043565 |
| Workplace very noisy: Sometimes | Whole_Blood | 0.00602 | 2.7469 | 0.021474 |
| Job SOC coding: Metal working production and maintenance fitters | Muscle_Skeletal | 0.00603 | 2.746 | 0.041895 |
| Frequency of memory loss due to drinking alcohol in last year | Brain_Hypothalamus | 0.00604 | 2.7458 | 0.12866 |
| Diagnoses - main ICD10: C61 Malignant neoplasm of prostate | Testis | 0.00604 | 2.7457 | 0.0056281 |
| Pulse rate, automated reading | Whole_Blood | 0.00605 | 2.7451 | 0.25017 |
| Amount of alcohol drunk on a typical drinking day | Whole_Blood | 0.00612 | 2.7414 | 0.039675 |
| How are people in household related to participant: Husband, wife or partner | Pituitary | 0.00644 | 2.7247 | 0.20048 |
| How are people in household related to participant: Husband, wife or partner | Brain_Putamen_basal_ganglia | 0.00644 | 2.7247 | 0.090361 |
| How are people in household related to participant: Husband, wife or partner | Brain_Nucleus_accumbens_basal_ganglia | 0.00644 | 2.7247 | 0.11752 |
| How are people in household related to participant: Husband, wife or partner | Brain_Caudate_basal_ganglia | 0.00644 | 2.7247 | 0.17633 |
| Job SOC coding: Further education teaching professionals | Muscle_Skeletal | 0.0066 | -2.7163 | -0.059835 |
| Diagnoses - main ICD10: M18 Arthrosis of first carpometacarpal joint | Brain_Hypothalamus | 0.00661 | 2.716 | 0.00565 |
| Non-cancer illness code, self-reported: tuberculosis (tb) | Muscle_Skeletal | 0.00664 | -2.7145 | -0.012801 |
| Food weight | Muscle_Skeletal | 0.00664 | -2.7143 | -437.88 |
| Vascular/heart problems diagnosed by doctor: High blood pressure | Minor_Salivary_Gland | 0.00677 | -2.7082 | -0.11659 |
| Type of meals eaten: Ready meals | Testis | 0.00681 | 2.706 | 0.036064 |
| Endocrine, nutritional and metabolic diseases | Pituitary | 0.00682 | -2.7055 | -0.063649 |
| Endocrine, nutritional and metabolic diseases | Brain_Nucleus_accumbens_basal_ganglia | 0.00682 | -2.7055 | -0.03731 |
| Endocrine, nutritional and metabolic diseases | Brain_Caudate_basal_ganglia | 0.00682 | -2.7055 | -0.055983 |
| Endocrine, nutritional and metabolic diseases | Brain_Putamen_basal_ganglia | 0.00682 | -2.7055 | -0.028688 |
| Diagnoses - main ICD10: Z40 Prophylactic surgery | Whole_Blood | 0.00688 | -2.7027 | -0.00081407 |
| Non-cancer illness code, self-reported: rheumatic fever | Minor_Salivary_Gland | 0.00694 | 2.6999 | 0.014557 |
| Other diseases of the digestive system | Brain_Hypothalamus | 0.00702 | -2.6957 | -0.015013 |
| Sum of road length of major roads within 100m | Brain_Nucleus_accumbens_basal_ganglia | 0.00704 | -2.6949 | -0.15055 |
| Sum of road length of major roads within 100m | Brain_Putamen_basal_ganglia | 0.00704 | -2.6949 | -0.11576 |
| Sum of road length of major roads within 100m | Pituitary | 0.00704 | -2.6949 | -0.25682 |
| Sum of road length of major roads within 100m | Brain_Caudate_basal_ganglia | 0.00704 | -2.6949 | -0.22589 |
| Treatment/medication code: synalar 1:10 cream (20003_1140913292) | Muscle_Skeletal | 0.00708 | 2.6929 | 0.003046 |
| Diagnoses - main ICD10: M51 Other intervertebral disk disorders | Minor_Salivary_Gland | 0.00708 | 2.6929 | 0.031153 |
| Other or ill-defined heart diseases | Minor_Salivary_Gland | 0.00709 | 2.6925 | 0.0081673 |
| Job coding: school secretary (22601_42133300) | Muscle_Skeletal | 0.00714 | -2.6902 | -0.023165 |
| Vascular/heart problems diagnosed by doctor: None of the above | Minor_Salivary_Gland | 0.00737 | 2.6796 | 0.11759 |
| Severity of problems due to mania or irritability | Brain_Hypothalamus | 0.00738 | -2.6793 | -0.18071 |
| Comparative height size at age 10 | Muscle_Skeletal | 0.00745 | 2.6758 | 0.12322 |
| Diagnoses - main ICD10: J22 Unspecified acute lower respiratory infection | Testis | 0.00757 | 2.6707 | 0.0049812 |
| Treatment/medication code: serc-8 tablet (20003_1140868064) | Minor_Salivary_Gland | 0.0076 | 2.6694 | 0.0046427 |
| Non-cancer illness code, self-reported: iron deficiency anaemia | Brain_Nucleus_accumbens_basal_ganglia | 0.0076 | 2.6691 | 0.019444 |
| Non-cancer illness code, self-reported: iron deficiency anaemia | Brain_Caudate_basal_ganglia | 0.0076 | 2.6691 | 0.029175 |
| Non-cancer illness code, self-reported: iron deficiency anaemia | Pituitary | 0.0076 | 2.6691 | 0.03317 |
| Non-cancer illness code, self-reported: iron deficiency anaemia | Brain_Putamen_basal_ganglia | 0.0076 | 2.6691 | 0.014951 |
| Job coding: advertising or public relations manager, media/publicity manager, campaign/fundraising manager (22601_11343224) | Muscle_Skeletal | 0.00762 | -2.6684 | -0.027382 |
| Illnesses of adopted siblings: Heart disease | Minor_Salivary_Gland | 0.0077 | 2.6649 | 1.0634 |
| Job SOC coding: Vocational and industrial trainers and instructors | Whole_Blood | 0.00774 | -2.6634 | -0.0043584 |
| Frequency of consuming six or more units of alcohol | Muscle_Skeletal | 0.00774 | -2.6631 | -0.36917 |
| Fractured bone site(s): Arm | Brain_Hypothalamus | 0.00775 | 2.6629 | 0.010808 |
| Ptosis of eyelid | Pituitary | 0.00782 | 2.6599 | 0.022524 |
| Ptosis of eyelid | Brain_Putamen_basal_ganglia | 0.00782 | 2.6599 | 0.010152 |
| Ptosis of eyelid | Brain_Nucleus_accumbens_basal_ganglia | 0.00782 | 2.6599 | 0.013203 |
| Ptosis of eyelid | Brain_Caudate_basal_ganglia | 0.00782 | 2.6599 | 0.019811 |
| Hearing difficulty/problems: Yes | Brain_Hypothalamus | 0.00788 | -2.6571 | -0.047087 |
| Diagnoses - main ICD10: D16 Benign neoplasm of bone and articular cartilage | Muscle_Skeletal | 0.00795 | -2.6544 | -0.0044897 |
| Blood clot, DVT, bronchitis, emphysema, asthma, rhinitis, eczema, allergy diagnosed by doctor: Hayfever, allergic rhinitis or eczema | Brain_Hypothalamus | 0.00795 | -2.6543 | -0.044761 |
| Type of fat/oil used in cooking: Polyunsaturated margarine | Minor_Salivary_Gland | 0.00796 | 2.6536 | 0.19289 |
| Diagnoses - main ICD10: S02 Fracture of skull and facial bones | Minor_Salivary_Gland | 0.00798 | 2.6527 | 0.016223 |
| Frequency of inability to cease drinking in last year | Brain_Hypothalamus | 0.008 | 2.6519 | 0.16188 |
| Job coding: draughtsperson, cartographer, designer-detailer/draughtsperson, drawing office checker, engineering draughtsperson (22601_31223293) | Whole_Blood | 0.00804 | -2.6505 | -0.0023527 |
| Polyneuropathies and other disorders of the peripheral nervous system | Testis | 0.00806 | -2.6496 | -0.0019406 |
| Long-standing illness, disability or infirmity | Brain_Hypothalamus | 0.00808 | 2.6486 | 0.049992 |
| Behavioural syndromes associated with physiological disturbances and physical factors | Muscle_Skeletal | 0.0081 | 2.6477 | 0.0040295 |
| Diagnoses - main ICD10: Z12 Special screening examination for neoplasms | Brain_Putamen_basal_ganglia | 0.00813 | 2.6465 | 0.021585 |
| Diagnoses - main ICD10: Z12 Special screening examination for neoplasms | Brain_Nucleus_accumbens_basal_ganglia | 0.00813 | 2.6465 | 0.028071 |
| Diagnoses - main ICD10: Z12 Special screening examination for neoplasms | Brain_Caudate_basal_ganglia | 0.00813 | 2.6465 | 0.042121 |
| Diagnoses - main ICD10: Z12 Special screening examination for neoplasms | Pituitary | 0.00813 | 2.6465 | 0.047888 |
| Drinking water intake | Muscle_Skeletal | 0.00825 | -2.6416 | -0.92833 |
| Inflammatory diseases of the central nervous system | Whole_Blood | 0.00826 | 2.6415 | 0.00060283 |
| Frequency of tenseness / restlessness in last 2 weeks | Testis | 0.00826 | 2.6412 | 0.030285 |
| Mental health problems ever diagnosed by a professional: Psychological over-eating or binge-eating | Minor_Salivary_Gland | 0.00829 | 2.64 | 0.030087 |
| Birth Weight | Brain_Hypothalamus | 0.0083 | -2.6396 |  |
| Age of stopping smoking | Minor_Salivary_Gland | 0.00831 | 2.639 | 10.281 |
| Signal-to-noise-ratio (SNR) of triplet (right) | Pituitary | 0.00834 | -2.638 | -1.4931 |
| Signal-to-noise-ratio (SNR) of triplet (right) | Brain_Nucleus_accumbens_basal_ganglia | 0.00834 | -2.638 | -0.87526 |
| Signal-to-noise-ratio (SNR) of triplet (right) | Brain_Caudate_basal_ganglia | 0.00834 | -2.638 | -1.3133 |
| Signal-to-noise-ratio (SNR) of triplet (right) | Brain_Putamen_basal_ganglia | 0.00834 | -2.638 | -0.67301 |
| Years of cough on most days | Minor_Salivary_Gland | 0.0084 | 2.6354 | 20.856 |
| 3mm regularity index (left) | Muscle_Skeletal | 0.00847 | 2.6326 | 0.55312 |
| Diagnoses - main ICD10: R52 Pain, not elsewhere classified | Brain_Hypothalamus | 0.00853 | 2.6302 | 0.003188 |
| Hearing difficulty/problems: No | Brain_Hypothalamus | 0.00856 | 2.629 | 0.046604 |
| Treatment/medication code: tetralysal 300 capsule (20003_1140873476) | Testis | 0.00857 | -2.6287 | -0.00098391 |
| Illnesses of adopted father: None of the above (group 1) | Testis | 0.00859 | -2.6279 | -0.28081 |
| Job coding: senior official in central government including members of the senior civil service and the equivalent in the diplomatic service, mps, meps (22601_11113200) | Minor_Salivary_Gland | 0.00861 | 2.6272 | 0.050802 |
| Type of tobacco previously smoked: Manufactured cigarettes | Muscle_Skeletal | 0.00862 | 2.6268 | 0.11125 |
| Job SOC coding: IT operations technicians | Brain_Putamen_basal_ganglia | 0.00869 | -2.6241 | -0.043344 |
| Job SOC coding: IT operations technicians | Brain_Nucleus_accumbens_basal_ganglia | 0.00869 | -2.6241 | -0.05637 |
| Job SOC coding: IT operations technicians | Pituitary | 0.00869 | -2.6241 | -0.096164 |
| Job SOC coding: IT operations technicians | Brain_Caudate_basal_ganglia | 0.00869 | -2.6241 | -0.084583 |
| Birth weight of first child | Whole_Blood | 0.00875 | -2.6216 | -0.024523 |
| Primary coxarthrosis, bilateral | Brain_Hypothalamus | 0.00876 | 2.6213 | 0.0042181 |
| Waking too early | Testis | 0.00884 | -2.6181 | -0.05864 |
| Blood clot, DVT, bronchitis, emphysema, asthma, rhinitis, eczema, allergy diagnosed by doctor: None of the above | Brain_Hypothalamus | 0.00886 | 2.6173 | 0.049085 |
| Malignant neoplasm of liver and intrahepatic bile ducts | Brain_Putamen_basal_ganglia | 0.00889 | -2.6163 | -0.0047112 |
| Malignant neoplasm of liver and intrahepatic bile ducts | Brain_Nucleus_accumbens_basal_ganglia | 0.00889 | -2.6163 | -0.0061271 |
| Malignant neoplasm of liver and intrahepatic bile ducts | Pituitary | 0.00889 | -2.6163 | -0.010452 |
| Malignant neoplasm of liver and intrahepatic bile ducts | Brain_Caudate_basal_ganglia | 0.00889 | -2.6163 | -0.0091935 |
| 6mm asymmetry angle (left) | Whole_Blood | 0.00904 | -2.6107 | -3.9314 |
| Non-cancer illness code, self-reported: thyroid problem (not cancer) | Minor_Salivary_Gland | 0.00906 | -2.6099 | -0.013378 |
| Other or ill-defined heart diseases | Brain_Caudate_basal_ganglia | 0.00914 | -2.6068 | -0.011736 |
| Other or ill-defined heart diseases | Brain_Nucleus_accumbens_basal_ganglia | 0.00914 | -2.6068 | -0.0078215 |
| Other or ill-defined heart diseases | Brain_Putamen_basal_ganglia | 0.00914 | -2.6068 | -0.0060141 |
| Other or ill-defined heart diseases | Pituitary | 0.00914 | -2.6068 | -0.013343 |
| Diagnoses - main ICD10: I86 Varicose veins of other sites | Muscle_Skeletal | 0.00917 | -2.6055 | -0.0045403 |
| Type milk consumed: wholemilk | Pituitary | 0.00918 | -2.6051 | -0.26973 |
| Type milk consumed: wholemilk | Brain_Putamen_basal_ganglia | 0.00918 | -2.6051 | -0.12158 |
| Type milk consumed: wholemilk | Brain_Nucleus_accumbens_basal_ganglia | 0.00918 | -2.6051 | -0.15811 |
| Type milk consumed: wholemilk | Brain_Caudate_basal_ganglia | 0.00918 | -2.6051 | -0.23724 |
| Type milk consumed: powdered milk | Testis | 0.00919 | 2.6048 | 0.008547 |

**Supplementary Table S16. Traits associated with predicted tissue-specific *HEY1* expression in PhenomeXcan.** Only associations that met the threshold (p < 1e-08) are listed in the table.

| **PUBMEDID** | **FIRST AUTHOR** | **DATE** | **JOURNAL** | **DISEASE/TRAIT** | **INITIAL SAMPLE SIZE** | **REGION** | **SNPS** | **P-VALUE** | **OR or BETA** |
| --- | --- | --- | --- | --- | --- | --- | --- | --- | --- |
| 23823483 | Rhee EP | 2013-07-02 | Cell Metab | Metabolite levels | 2,076 | 8q21.13 | rs6473177 | 8E-06 | 0.3819534 |
| 32958699 | Innocenti F | 2020-09-21 | Clin Cancer Res | Metastatic colorectal cancer survival in treatment with chemotherapy plus biologics | 613 | 8q21.13 | rs2461035 | 5E-06 | 0.72 |
| 35361970 | Okbay A | 2022-03-31 | Nat Genet | Educational attainment | 3,037,499 | 8q21.13 | rs2461063 | 3E-11 | 0.0074074 |

**Supplementary Table S17. Associations with *HEY1* variants in the GWAS Catalog.** Only associations that met the threshold (p < 1e-05) are listed in the table.

| **HEY2** | | | | |
| --- | --- | --- | --- | --- |
| **PheWAS** | | | | |
| **GeneAtlas** | **Respiratory-related Phenotype** | **Variant** | **P-value** | **GWAS** |
|  | J39 Other diseases of upper respiratory tract | rs188787682 | 2.86E-10 | |
| **PhenoScanner** | **Thyroid-related Phenotype** | **Variant** | **P-value** | **GWAS** |
|  | Cause of death: malignant neoplasm of thyroid gland | rs151283443 | 0.000001282 | UKBB |
| **HEYL** | | | | |
| **PheWAS** | | | | |
| **PhenoScanner** | **Respiratory-related Phenotype** | **Variant** | **P-value** | **GWAS** |
|  | Cause of death: other specified respiratory disorders | rs551113969 | 3.077E-44 | UKBB |
|  | Cause of death: asthma, unspecified | rs41264503 | 5.985E-08 | UKBB |
|  | Cause of death: other specified respiratory disorders | rs563028798 | 2.369E-07 | UKBB |
|  | **Thyroid-related Phenotype** |  |  |  |
|  | Cause of death: malignant neoplasm of thyroid gland | rs185638484 | 8.291E-10 | UKBB |
|  | Cause of death: malignant neoplasm of thyroid gland | rs41264497 | 6.666E-07 | UKBB |
| **TWAS** | | | | |
| **PhenomeXcan** | **Respiratory-related Phenotype** | **Tissue** | **P-value** | **Z-score** |
|  | Non-cancer illness code, self-reported: nasal polyps | Testis | 0.0000359 | 4.1322 |
|  | Non-cancer illness code, self-reported: nasal polyps | Muscle_skeletal | 0.000129 | 3.828 |
|  | Underlying (primary) cause of death: ICD10: J84.1 Other interstitial pulmonary diseases with fibrosis | Pituitary; Brain_Cerebellar_Hemisphere; Heart_Atrial_Appendage; Brain_Nucleus_accumbens_basal_ganglia; Breast_Mammary_Tissue; Brain_Cerebellum; Spleen; Lung | 0.00195 | 3.0979 |
|  | Non-cancer illness code, self-reported: emphysema/chronic bronchitis | Colon_transverse | 0.00252 | -3.0206 |
|  | Doctor diagnosed asthma | Cell_cultured_fibroblasts | 0.00279 | -2.9901 |
| **TWAS Hub** | **Respiratory-related Phenotype** | **Tissue** | **P-value** | **Z-score** |
|  | Lung FEV1/FVC ratio | Adipose | 6E-10 | 6.2 |
|  | Peak expiratory flow | Adipose | 0.000966965 | 3.3 |

**Supplementary Table S18. PheWAS and TWAS respiratory and thyroid associations with *HEY* family genes.** Associations are listed in the table by analysis type and database. No relevant TWAS associations were identified for *HEY2.*

| **Trait** | **Variant** | **Position** | **-log10(p-value)** |
| --- | --- | --- | --- |
| nervous breakdown | rs60196933 | 41553370 | 8.192241794 |
| asthma | rs9943 | 40326282 | 8.493684421 |
| hypertension | rs112684153 | 41849731 | 9.877849681 |
| Comparative body size at age 10 | rs61954177 | 40787036 | 18.59250712 |
| Comparative height size at age 10 | rs9315784 | 41289166 | 13.11332319 |
| Mean reticulocyte volume | rs9549243 | 41217205 | 23.837346 |
| Immature reticulocyte fraction | rs9594607 | 42240641 | 8.595576834 |
| Reticulocyte percentage | rs9566791 | 42144480 | 9.434400083 |
| Reticulocyte count | rs9566791 | 42144480 | 9.31999101 |
| High light scatter reticulocyte count | rs1023243 | 42151623 | 13.30493881 |
| Mean sphered cell volume | rs9549243 | 41217205 | 28.64977137 |
| High light scatter reticulocyte percentage | rs1023243 | 42151623 | 13.30340774 |
| Nucleated red blood cell count | rs150057490 | 41111178 | 14.40344689 |
| Lymphocyte count | rs9532679 | 41522338 | 21.34806329 |
| Monocyte count | rs1892548 | 41002641 | 147.6412469 |
| Eosinophill count | rs12875311 | 40342557 | 36.94931097 |
| Lymphocyte percentage | rs9532679 | 41522338 | 28.66735959 |
| Monocyte percentage | rs1892548 | 41002641 | 151.2719873 |
| Neutrophill percentage | rs7323267 | 41204015 | 22.61332272 |
| Eosinophill percentage | rs12875311 | 40342557 | 37.59689665 |
| Platelet crit | rs9590569 | 41584690 | 29.36813026 |
| Mean corpuscular haemoglobin | rs9532563 | 41160270 | 24.3074527 |
| Platelet count | rs9532580 | 41244260 | 27.84639853 |
| Mean corpuscular volume | rs7323267 | 41204015 | 27.4997498 |
| Red blood cell (erythrocyte) count | rs9549260 | 41254104 | 23.01040337 |
| Whole body fat mass | rs1336486 | 40784814 | 10.88219859 |
| Trunk predicted mass | rs9532583 | 41256473 | 14.91599601 |
| Trunk fat-free mass | rs9532583 | 41256473 | 15.42131496 |
| Trunk fat mass | rs1336486 | 40784814 | 12.05094682 |
| Trunk fat percentage | rs1336486 | 40784814 | 8.064336577 |
| Arm predicted mass (left) | rs9532583 | 41256473 | 12.57045765 |
| Arm fat-free mass (left) | rs9532583 | 41256473 | 12.03016025 |
| Arm fat mass (left) | rs1336486 | 40784814 | 10.75023926 |
| Arm fat percentage (left) | rs1336486 | 40784814 | 9.556956171 |
| Arm predicted mass (right) | rs9532583 | 41256473 | 11.38018692 |
| Arm fat-free mass (right) | rs9532583 | 41256473 | 11.41184038 |
| Arm fat mass (right) | rs1336486 | 40784814 | 11.10368326 |
| Arm fat percentage (right) | rs1336486 | 40784814 | 8.773812677 |
| Leg predicted mass (left) | rs9532583 | 41256473 | 17.44784302 |
| Leg fat-free mass (left) | rs9532583 | 41256473 | 17.25527685 |
| Leg fat mass (left) | rs1336486 | 40784814 | 8.880546149 |
| Leg predicted mass (right) | rs9532583 | 41256473 | 16.69671752 |
| Leg fat-free mass (right) | rs9532583 | 41256473 | 16.8305901 |
| Whole body fat-free mass | rs9532583 | 41256473 | 17.51270604 |
| Leg fat mass (right) | rs1336486 | 40784814 | 9.275920837 |
| Impedance of leg (left) | rs1336486 | 40784814 | 10.12426177 |
| Impedance of leg (right) | rs1336486 | 40784814 | 9.064281181 |
| Basal metabolic rate | rs9532583 | 41256473 | 16.79841172 |
| Body mass index (BMI) | rs1336486 | 40784814 | 11.08982933 |
| Whole body water mass | rs9532583 | 41256473 | 17.14455959 |
| Weight | rs1336486 | 40784814 | 14.25473778 |
| Sitting height | rs2755237 | 41109429 | 18.59875459 |
| Body mass index (BMI) | rs61954177 | 40787036 | 11.09539264 |
| Weight | rs1336486 | 40784814 | 13.79274274 |
| Standing height | rs7996228 | 41281731 | 22.77062537 |
| Hip circumference | rs9532583 | 41256473 | 10.15336604 |
| Waist circumference | rs1336486 | 40784814 | 9.947229131 |
| sarcoidosis | rs201909382 | 42149949 | 9.795201962 |
| Frequency of solarium/sunlamp use | rs9549143 | 40851361 | 10.5729523 |

**Supplementary Table S19. PheWAS associations with *FOXO1* in GeneATLAS.** Only associations that met the threshold (p < 1e-08) are listed in the table.

| **PUBMEDID** | **FIRST AUTHOR** | **DATE** | **DISEASE/TRAIT** | **INITIAL SAMPLE SIZE** | **SNP** | **P-VALUE** | **ODDS RATIO (OR) or BETA** |
| --- | --- | --- | --- | --- | --- | --- | --- |
| 30598549 | Morris JA | 2018-12-31 | Heel bone mineral density | 426,824 British ancestry individuals | rs76983463 | 2E-19 | 0.0423875 |
| 32888493 | Chen MH | 2020-09-01 | Basophil count | 474,001 European ancestry individuals | rs2701863 | 2E-13 | 0.052069 |
| 32888493 | Chen MH | 2020-09-01 | Basophil count | 577,663 African American or Afro-Caribbean, African ancestry, European ancestry, East Asian ancestry, Hispanic or Latin American and South Asian ancestry individuals | rs2701863 | 4E-12 |  |
| 31361310 | Johansson A | 2019-07-30 | Allergic disease (asthma, hay fever or eczema) | 106,772 European ancestry cases, 239,773 European ancestry controls | rs9549238 | 1E-11 | 1.046 |
| 31361310 | Johansson A | 2019-07-30 | Hay fever and/or eczema | 84,034 European ancestry cases, 239,773 European ancestry controls | rs113377887 | 2E-12 | 1.052 |
| 32888493 | Chen MH | 2020-09-01 | Eosinophil counts | 583,850 African American or Afro-Caribbean, African ancestry, European ancestry, East Asian ancestry, Hispanic or Latin American and South Asian ancestry individuals | rs7985364 | 5E-59 |  |
| 29083406 | Ferreira MA | 2017-10-30 | Allergic disease (asthma, hay fever or eczema) | 180,129 European ancestry cases, 180,709 European ancestry controls | rs4943794 | 7E-12 | 1.043 |
| 28552196 | Tachmazidou I | 2017-06-01 | Weight | 1,249 whole genome sequenced European ancestry individuals, 3,559 whole genome sequenced individuals, 46,819 European ancestry individuals, 472 Carlantino (founder/genetic isolate) individuals, 1,172 Friuli Venezia Giulia (founder/genetic isolate) individuals, 1,051 Mylopotamos (founder/genetic isolate) individuals, 942 Pomak (founder/genetic isolate) individuals, 1,779 Val Borbera (founder/genetic isolate) individuals | rs3841231 | 6E-06 | 0.035 |
| 32888493 | Chen MH | 2020-09-01 | Mean corpuscular volume | 696,882 African American or Afro-Caribbean, African ancestry, European ancestry, East Asian ancestry, Hispanic or Latin American and South Asian ancestry individuals | rs9549238 | 4E-42 |  |
| 32888494 | Vuckovic D | 2020-09-01 | Mean corpuscular volume | 408,112 British individuals | rs9532562 | 7E-25 | 0.0312824 |
| 32888494 | Vuckovic D | 2020-09-01 | Mean corpuscular hemoglobin | 408,112 British individuals | rs9532563 | 4E-18 | 0.023734156 |
| 32888493 | Chen MH | 2020-09-01 | Mean corpuscular hemoglobin | 486,823 European ancestry individuals | rs9532563 | 5E-29 | 0.026091 |
| 32888493 | Chen MH | 2020-09-01 | Mean corpuscular volume | 544,127 European ancestry individuals | rs9532563 | 6E-42 | 0.02991 |
| 32888494 | Vuckovic D | 2020-09-01 | High light scatter reticulocyte count | 408,112 British individuals | rs11459608 | 5E-10 | 0.015127829 |
| 32888494 | Vuckovic D | 2020-09-01 | Monocyte count | 408,112 British individuals | rs4325427 | 2E-32 | 0.028901674 |
| 32888494 | Vuckovic D | 2020-09-01 | Monocyte percentage of white cells | 408,112 British individuals | rs4325427 | 3E-43 | 0.033687208 |
| 27863252 | Astle WJ | 2016-11-17 | Granulocyte percentage of myeloid white cells | 169,545 European ancestry individuals | rs4325427 | 3E-20 | 0.03641795 |
| 27863252 | Astle WJ | 2016-11-17 | Mean corpuscular volume | 172,433 European ancestry individuals | rs9532563 | 7E-12 | 0.02931834 |
| 27863252 | Astle WJ | 2016-11-17 | Monocyte percentage of white cells | 170,494 European ancestry individuals | rs4325427 | 1E-16 | 0.03253815 |
| 30595370 | Kichaev G | 2018-12-27 | Mean corpuscular hemoglobin | approximately 443,000 European ancestry individuals | rs9532563 | 2E-31 |  |
| 30595370 | Kichaev G | 2018-12-27 | Eczema | approximately 459,000 European ancestry individuals | rs9549243 | 1E-13 |  |
| 32888494 | Vuckovic D | 2020-09-01 | Mean reticulocyte volume | 408,112 British individuals | rs9532564 | 6E-20 | 0.025922857 |
| 32888494 | Vuckovic D | 2020-09-01 | Mean spheric corpuscular volume | 408,112 British individuals | rs9532562 | 2E-26 | 0.029418586 |
| 32888494 | Vuckovic D | 2020-09-01 | Plateletcrit | 408,112 British individuals | rs3900833 | 1E-15 | 0.020105602 |
| 34104963 | Thompson A | 2021-06-08 | Mean corpuscular volume | 362,595 British ancestry individuals | rs9532563 | 5E-22 | 0.13 |
| 34469753 | Kachuri L | 2021-08-26 | Platelet-to-lymphocyte ratio | 234,552 European ancestry individuals | rs1986649 | 1E-19 |  |
| 34469753 | Kachuri L | 2021-08-26 | Monocyte count | 234,690 European ancestry individuals | rs1986649 | 9E-12 |  |
| 35446358 | He W | 2022-04-21 | Corneal resistance factor (MTAG) | 123,734 European ancestry individuals | rs10507486 | 4E-09 | 0.028106 |
| 27863252 | Astle WJ | 2016-11-17 | Monocyte count | 170,721 European ancestry individuals | rs4325427 | 4E-15 | 0.03100491 |
| 31649266 | Gallagher CS | 2019-10-24 | Uterine fibroids | 20,406 European ancestry cases, 223,918 European ancestry controls | rs7986407 | 2E-13 | 0.064 |
| 30595370 | Kichaev G | 2018-12-27 | Heel bone mineral density | approximately 446,000 European ancestry individuals | rs76983463 | 7E-16 |  |
| 33311554 | Jiang X | 2020-12-11 | Corneal resistance factor | 76,029 white-British ancestry individuals | rs9532564 | 5E-09 | 0.0615037 |
| 30194396 | Rafnar T | 2018-09-07 | Uterine fibroids | 16,595 European ancestry cases, 523,330 European ancestry controls | rs7986407 | 7E-08 | 1.07 |
| 31070453 | Zekavat SM | 2019-04-11 | Arterial stiffness | 131,686 British ancestry individuals | rs7331212 | 9E-09 | 0.024 |
| 31235810 | Fung K | 2019-06-24 | Arterial stiffness index | 127,121 European ancestry individuals | rs7331212 | 2E-11 | 0.03 |
| 36329257 | Lee CJ | 2022-11-03 | Red blood cell count | 76,086 Taiwanese ancestry individuals | rs9549254 | 6E-06 |  |
| 34648354 | Pietzner M | 2021-11-12 | Forkhead box protein M1 levels | 10,708 European ancestry individuals | rs117828578 | 2E-25 | 0.338 |
| 30048462 | Kim SK | 2018-07-26 | Heel bone mineral density | 394,929 European ancestry individuals | rs17061453 | 4E-15 | 0.0426284 |
| 32888494 | Vuckovic D | 2020-09-01 | Platelet count | 408,112 British individuals | rs3900833 | 7E-13 | 0.018042846 |
| 35935937 | Hoglund J | 2022-07-22 | Eosinophil counts | 365,954 British ancestry individuals | rs9577090 | 1E-19 | 0.036 |
| 36726022 | Sliz E | 2023-02-01 | Uterine leiomyomata | 38,466 European ancestry cases, 329,437 European ancestry controls | rs7986407 | 2E-18 | 0.93 |
| 36653354 | Saarentaus EC | 2023-01-18 | Chronic inflammatory sinonasal disease | 19,901 Finnish ancestry cases, 199,208 Finnish ancestry controls | rs2701859 | 7E-09 | 1.08 |
| 36653354 | Saarentaus EC | 2023-01-18 | Sinonasal disease | 25,235 Finnish ancestry cases, 199,208 Finnish ancestry controls | rs2701859 | 4E-07 | 1.06 |
| 38191017 | Xiao C | 2024-01-06 | Age at natural menopause or uterine leiomyomata (pleiotropy) | 35,474 European ancestry uterine leiomyomata cases, 267,505 European ancestry controls, 201,323 European ancestry individuals with age at natural menopause measurements | rs7986407 | 4E-19 |  |
| 38538606 | Harris BHL | 2024-03-27 | Whole body fat free mass (UKB data field 23101) | 337,739 European ancestry individuals | rs2253001 | 6E-10 | 0.00883715 |
| 37684235 | Brown DW | 2023-09-08 | Myeloproliferative neoplasms (MTAG) | at least 1,427 cases, at least 480,951 controls (MTAG boosted by telomere length and loss of chromosome Y samples) | rs7323267 | 6E-13 | 0.012 |
| 36224396 | Yengo L | 2022-10-12 | Height | 455,180 Hispanic or Latin American individuals | rs2755209 | 2E-09 | 0.013 |
| 30595370 | Kichaev G | 2018-12-27 | Height | approximately 458,000 European ancestry individuals | rs7996228 | 8E-22 |  |
| 32888493 | Chen MH | 2020-09-01 | Hematocrit | 562,259 European ancestry individuals | rs9549260 | 2E-11 | 0.015152 |
| 32888494 | Vuckovic D | 2020-09-01 | Eosinophil percentage of white cells | 408,112 British individuals | rs2065633 | 6E-42 | 0.033996202 |
| 32888493 | Chen MH | 2020-09-01 | Hemoglobin concentration | 563,946 European ancestry individuals | rs9549260 | 7E-11 | 0.014695 |
| 31798171 | Bonnemaijer PWM | 2019-11-27 | Central corneal thickness | 16,204 European ancestry individuals | rs2721051 | 4E-20 | 5.836 |
| 30595370 | Kichaev G | 2018-12-27 | Eosinophil counts | approximately 440,000 European ancestry individuals | rs17061503 | 2E-52 |  |
| 30595370 | Kichaev G | 2018-12-27 | Red blood cell count | approximately 445,000 European ancestry individuals | rs9549260 | 4E-26 |  |
| 32888493 | Chen MH | 2020-09-01 | Eosinophil counts | 474,237 European ancestry individuals | rs6563842 | 3E-61 | 0.035704 |
| 20485516 | Lu Y | 2010-05-13 | Central corneal thickness | 3,473 European ancestry twins in 1,905 families, 301 European ancestry thin CCT cases, 301 European ancestry thick CCT controls | rs2721051 | 5E-10 | 0.24 |
| 23291589 | Lu Y | 2013-01-06 | Corneal structure | 13,057 European ancestry individuals, 2,538 Indian ancestry individuals, 2,542 Malay ancestry individuals, 1,883 Chinese ancestry individuals | rs2721051 | 4E-14 | 0.17 |
| 32888493 | Chen MH | 2020-09-01 | Neutrophil count | 519,288 European ancestry individuals | rs9532580 | 2E-10 | 0.013957 |
| 32888493 | Chen MH | 2020-09-01 | Mean corpuscular volume | 121,047 East Asian ancestry individuals | rs114224075 | 2E-29 | 0.056816 |
| 32888493 | Chen MH | 2020-09-01 | Red blood cell count | 545,203 European ancestry individuals | rs9549260 | 4E-36 | 0.028439 |
| 32888493 | Chen MH | 2020-09-01 | Red blood cell count | 150,708 East Asian ancestry individuals | rs60333767 | 4E-18 | 0.040106 |
| 32888493 | Chen MH | 2020-09-01 | Red blood cell count | 727,624 African American or Afro-Caribbean, African ancestry, European ancestry, East Asian ancestry, Hispanic or Latin American and South Asian ancestry individuals | rs9549260 | 3E-33 |  |
| 29403010 | Kanai M | 2018-02-05 | Mean corpuscular hemoglobin | 108,054 Japanese ancestry individuals | rs148041301 | 7E-23 | 0.05384 |
| 29235454 | Choquet H | 2017-12-13 | Intraocular pressure | 56,819 European ancestry individuals, 5,748 Hispanic/Latino individuals, 5,119 East Asian ancestry individuals, 2,070 African American individuals | rs11616662 | 8E-18 | 0.19 |
| 29403010 | Kanai M | 2018-02-05 | Mean corpuscular volume | 108,256 Japanese ancestry individuals | rs148041301 | 2E-25 | 0.05715 |
| 29403010 | Kanai M | 2018-02-05 | Red blood cell count | 108,794 Japanese ancestry individuals | rs111437842 | 1E-14 | 0.04176 |
| 27863252 | Astle WJ | 2016-11-17 | Eosinophil percentage of white cells | 172,378 European ancestry individuals | rs6563842 | 2E-15 | 0.0310048 |
| 27863252 | Astle WJ | 2016-11-17 | Eosinophil percentage of granulocytes | 170,536 European ancestry individuals | rs6563842 | 2E-10 | 0.02494378 |
| 30595370 | Kichaev G | 2018-12-27 | Lung function (FEV1/FVC) | approximately 370,000 European ancestry individuals | rs9532580 | 8E-10 |  |
| 30894546 | Ivarsdottir EV | 2019-03-20 | Corneal hysteresis | 6,125 Icelandic ancestry individuals | rs2721051 | 7E-07 | 0.17 |
| 30894546 | Ivarsdottir EV | 2019-03-20 | Corneal resistance factor | 6,125 Icelandic ancestry individuals | rs2721051 | 1E-09 | 0.21 |
| 35446358 | He W | 2022-04-21 | Central corneal thickness (MTAG) | 17,803 European ancestry individuals | rs2755238 | 7E-80 | 7.231712 |
| 35446358 | He W | 2022-04-21 | Corneal resistance factor (MTAG) | 123,734 European ancestry individuals | rs2755238 | 1E-96 | 0.135336 |
| 27863252 | Astle WJ | 2016-11-17 | Red blood cell count | 172,952 European ancestry individuals | rs9549260 | 2E-09 | 0.02604552 |
| 27863252 | Astle WJ | 2016-11-17 | Mean corpuscular hemoglobin | 172,332 European ancestry individuals | rs9532580 | 7E-09 | 0.02357476 |
| 27863252 | Astle WJ | 2016-11-17 | Sum eosinophil basophil counts | 171,771 European ancestry individuals | rs6563842 | 3E-13 | 0.0286538 |
| 31246245 | Khawaja AP | 2019-06-27 | Corneal resistance factor | 6,645 individuals | rs2721051 | 5E-11 | 0.27 |
| 31246245 | Khawaja AP | 2019-06-27 | Corneal hysteresis | 6,645 individuals | rs2721051 | 5E-11 | 0.25 |
| 33311554 | Jiang X | 2020-12-11 | Corneal resistance factor | 76,029 white-British ancestry individuals | rs11616662 | 5E-60 | 0.240534 |
| 33311554 | Jiang X | 2020-12-11 | Corneal resistance factor | 72,301 European ancestry individuals | rs11616662 | 7E-50 |  |
| 34594039 | Sakaue S | 2021-09-30 | Weight | 360,116 European ancestry individuals, 165,419 East Asian ancestry individuals | rs9532583 | 3E-13 | 0.0132 |
| 34594039 | Sakaue S | 2021-09-30 | Eosinophil counts | 349,856 European ancestry individuals, 93,063 East Asian ancestry individuals | rs2065633 | 1E-45 | 0.0276 |
| 33649486 | Hardcastle AJ | 2021-03-01 | Keratoconus | 2,116 European ancestry cases, 24,626 European ancestry controls | rs2721051 | 6E-35 | 0.452 |
| 36329257 | Lee CJ | 2022-11-03 | Red blood cell count | 76,086 Taiwanese ancestry individuals | rs115535790 | 1E-09 | 0.0140911 |
| 32716492 | Simcoe MJ | 2020-07-27 | Corneal hysteresis | 106,041 European ancestry individuals | rs2755238 | 7E-75 | 0.205 |
| 32716492 | Simcoe MJ | 2020-07-27 | Corneal resistance factor | 106,030 European ancestry individuals | rs11616662 | 4E-84 | 0.232 |
| 32888494 | Vuckovic D | 2020-09-01 | Red blood cell count | 408,112 British individuals | rs9549260 | 3E-26 | 0.028503282 |
| 32888494 | Vuckovic D | 2020-09-01 | Reticulocyte count | 408,112 British individuals | rs17061503 | 9E-12 | 0.01662681 |
| 36502284 | Yu XH | 2022-12-10 | Body surface area | 337,198 British ancestry individuals | rs9549263 | 2E-12 | 0.00310958 |
| 29617998 | Gao XR | 2018-03-28 | Intraocular pressure | 115,486 European ancestry individuals | rs11616662 | 5E-17 | 0.1962 |
| 29617998 | Gao XR | 2018-03-28 | Intraocular pressure | 115,486 European ancestry individuals | rs2721051 | 2E-16 | 0.1917 |
| 34594039 | Sakaue S | 2021-09-30 | Height | 360,388 European ancestry individuals, 165,056 East Asian ancestry individuals | rs9577111 | 8E-18 | 0.0121 |
| 34594039 | Sakaue S | 2021-09-30 | Mean corpuscular hemoglobin | 350,472 European ancestry individuals, 128,028 East Asian ancestry individuals | rs116541893 | 1E-29 | 0.0499 |
| 34594039 | Sakaue S | 2021-09-30 | Mean corpuscular volume | 350,473 European ancestry individuals, 129,832 East Asian ancestry individuals | rs60333767 | 2E-29 | 0.0495 |
| 34594039 | Sakaue S | 2021-09-30 | Red blood cell count | 350,475 European ancestry individuals, 153,512 East Asian ancestry individuals | rs9549258 | 8E-23 | 0.0235 |
| 34594039 | Sakaue S | 2021-09-30 | Mean corpuscular hemoglobin | 128,028 East Asian ancestry individuals | rs115535790 | 1E-29 | 0.049816 |
[truncated: 54,182 more chars]
